# Synthesis of Primary Thioamides by the Insertion of CS_2_ into Aldoximes

**DOI:** 10.1021/acs.orglett.6c01816

**Published:** 2026-06-08

**Authors:** Marcos López-Aguilar, Nicolás Ríos-Lombardía, Daniel Barrena-Espés, Miguel Gallegos, Joaquín García-Álvarez, Carmen Concellón, Vicente del Amo

**Affiliations:** ‡ Laboratorio de Química Sintética Sostenible (QuimSinSos), Organic and Inorganic Chemistry Department, Instituto de Química Organometálica Enrique Moles, 201467 Universidad de Oviedo, Avenida Julián Clavería 8, 33006 Oviedo, Asturias, Spain; § Department of Physics and Material Sciences, University of Luxembourg, L-1511 Luxembourg, Luxembourg; ⊥ Physical and Analytical Chemistry Department, Universidad de Oviedo, Avenida Julián Clavería 8, 33006 Oviedo, Asturias, Spain

## Abstract

Aldoximes react with CS_2_ in the presence of DBU and TBACl, providing access to primary thioamides. This unprecedented reaction proceeds under metal-free and mild conditions and tolerates a broad range of substrates. Its robustness has been demonstrated on the synthesis of active drug Febuxostat (gout) and an advanced intermediate in the synthesis of SRT2104 (type 2 diabetes). Computational studies, based on DFT calculations, have revealed the roles of both the base and TBACl in the reaction and have elucidated the reaction mechanism, which differs significantly from those displayed for other CS_2_ insertions.

## Graphical abstract

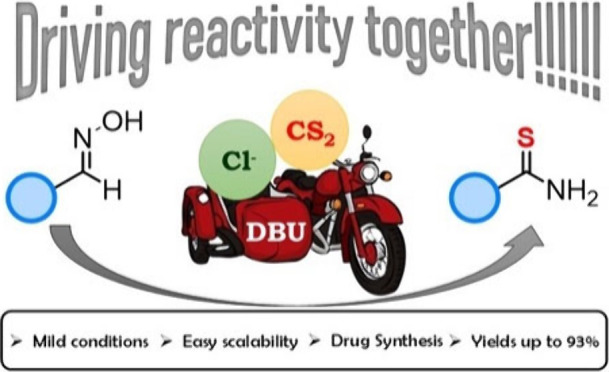

Thioamides constitute a fundamental class of sulfur-containing compounds that are broadly significant in biology,^1–3^ organocatalysis,^4,5^ and materials science.^6^ Their structural resemblance to amides, coupled with the unique influence of the sulfur atom, imparts distinct physicochemical and biological properties that make them attractive in diverse fields. For instance, thioamides are present in bioactive natural products and ribosomally synthesized peptides, where they modulate hydrogen-bonding patterns and confer enhanced metabolic stability.^7^ In medicinal chemistry, amide-to-thioamide substitution is often exploited to tune lipophilicity, proteolytic resistance, and binding affinity, thereby improving the pharmacokinetic profile of peptide-based therapeutics and small molecules.^8,9^

Primary thioamides, of the general form R–C­(=S)­NH_2_, are particularly relevant, representing the sulfur analogues of primary amides in which the carbonyl oxygen is replaced by sulfur. This substitution produces profound effects on the electronic distribution, resonance stabilization, and acid–base behavior. A distinctive feature of primary thioamides is the markedly increased acidity of the N–H bond relative to their oxygen analogues. In fact, experimental measurements indicate that the p*K*_a_ values of primary thioamides generally fall in the range of 8–10, depending on the electronic and steric environment, whereas amides typically display much higher p*K*_a_ values around 14–16 in aqueous solution.^10^ This enhanced acidity reflects both the weakened resonance stabilization of the thioamide ground state and the stronger stabilization of the conjugate base through sulfur’s diffuse orbitals,^10,11^ which has several important implications for the reactivity and synthetic utility of thioamides. First, thioamide deprotonation under mild conditions enables their use as versatile nucleophilic partners in bond-forming processes in the synthesis of heterocycles such as thiazoles, thiadiazoles, and related sulfur- and nitrogen-containing scaffolds.^12^ It expands their synthetic utility beyond classical amide analogues, positioning thioamides as privileged intermediates in modern synthetic organic chemistry. Second, in coordination chemistry, deprotonated thioamides act as ambidentate ligands, offering both S- and N-donor sites for transition-metal binding, thereby expanding the structural diversity and reactivity of metal complexes.^11^

Despite their aforementioned utility, conventional syntheses of primary thioamides are limited by severe drawbacks. In this sense, classical methods often rely on treating primary amides with harsh thionating agents such as phosphorus sulfides of various stoichiometries or Lawesson’s reagent (Scheme 1a).^13^ Other classical methods rely on the use of nitriles, which can be converted into primary thioamides upon vigorous treatment with sulfide derivatives (Scheme 1b).^14,15^ While effective in general, these reagents typically require severe reaction conditions, suffer from intrinsically poor chemoselectivity when some functional groups (e.g., ketones, aldehydes, esters, or amides) are present within the same molecular framework, and generate problematic byproducts.

**Scheme 1:**
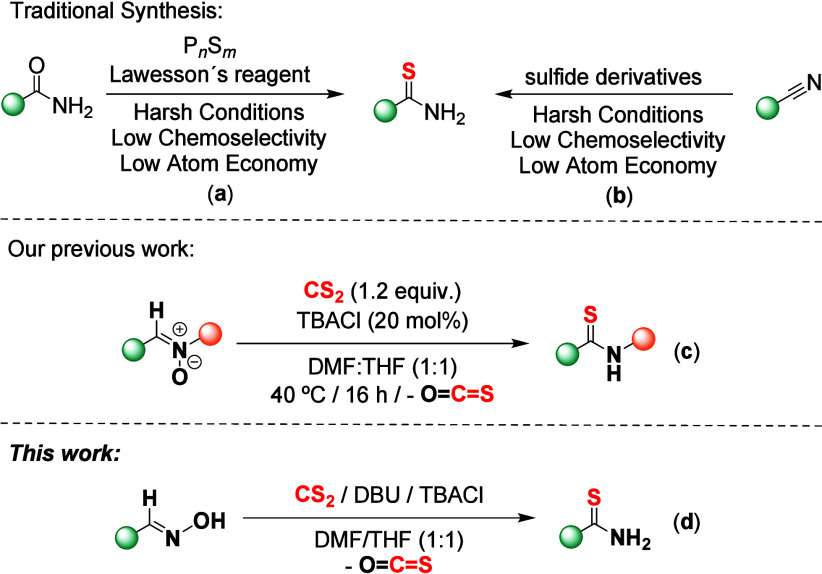
Summary of Methods for the Synthesis of Primary Thioamides

Considering our previous understanding of the chemistry of CS_2_ (Scheme 1c)^16^ and the pressing demand for the development of mild, selective, and sustainable strategies for the synthesis of primary thioamides, herein we present the design and implementation of an unprecedented strategy for the synthesis of these compounds using aldoximes as substrates (Scheme 1d). It is worth noting that our previously reported nitrone-based approach is intrinsically limited to the synthesis of secondary N-substituted thioamides.^16^ The possibility of preparing primary thioamides, offered by the addition of CS_2_ to aldoximes, addresses a previously unmet synthetic need and, hence, constitutes an orthogonal strategy. Importantly, and to the best of our knowledge, this work discloses the first documented example of reactivity between aldoximes and CS_2_.^17^ Moreover, the aldoximes used as substrates can be readily assembled from the corresponding aldehydes and *N*-hydroxylamine (see the Supporting Information), thus providing a modular, mild, and chemoselective entry to primary thioamides from readily accessible starting materials.

To our delight, upon optimization of the experimental reaction parameters (see section III of the Supporting Information), we found that benzaldehyde oxime (**1a**) and CS_2_ (1.2 equiv), dissolved in an anhydrous DMF/THF mixture, reacted in the presence of DBU (1.2 equiv)^18^ and TBACl (20 mol %) to afford primary benzothioamide **3a** in 82% isolated yield (Table 1, entry 1), working under mild reaction conditions (40 °C). A control experiment, conducted in the absence of a base, resulted in quantitative recovery of the starting material (Table 1, entry 2). In contrast, while not essential for the reaction to occur, the addition of the chloride salt enhanced the reaction efficiency (Table 1, entry 3), pointing to a synergistic effect, which is in good agreement with the proposed mechanism discussed below (Figure 1). Using off-the-bench solvent slightly erodes the reaction yield (Table 1, entry 4), while does not guarantee reproducibility.

**Table 1:**
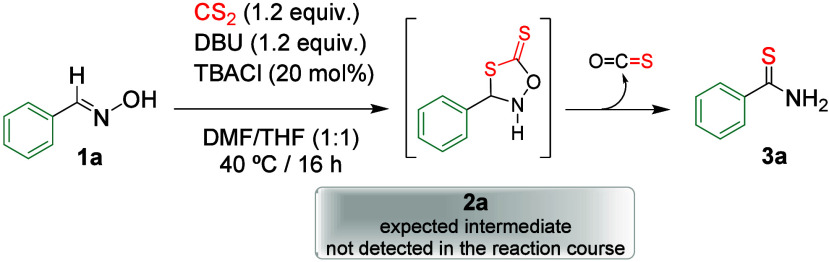
Synthesis of Benzothioamide (**3a**) from CS_2_ and Aldoxime **1a**, Promoted by DBU and TBACl^a^

entry	DBU (equiv)	TBACl (equiv)	yield (%)^b^
1	1.2	0.2	82
2	0	0.2	0
3	1.2	0	67
4^c^	1.2	0.2	76

a To a solution of benzaldehyde oxime **1a** (48 mg, 0.4 mmol) and the indicated amount of tetrabutylammonium chloride in 2 mL of a 1:1 anhydrous DMF/THF mixture were sequentially added the indicated amount of 1,8-diazabicyclo[5.4.0]­undec-7-ene (DBU) and CS_2_ (29 μL, 0.48 mmol, 1.2 equiv). The resulting reaction mixture was stirred at 40 °C (oil bath) for 16 h under an argon atmosphere.

b Isolated yield of analytically pure benzothioamide **3a** after flash chromatography.

c Reaction carried out using off-the-bench (not anhydrous) solvents.

**Figure 1:**
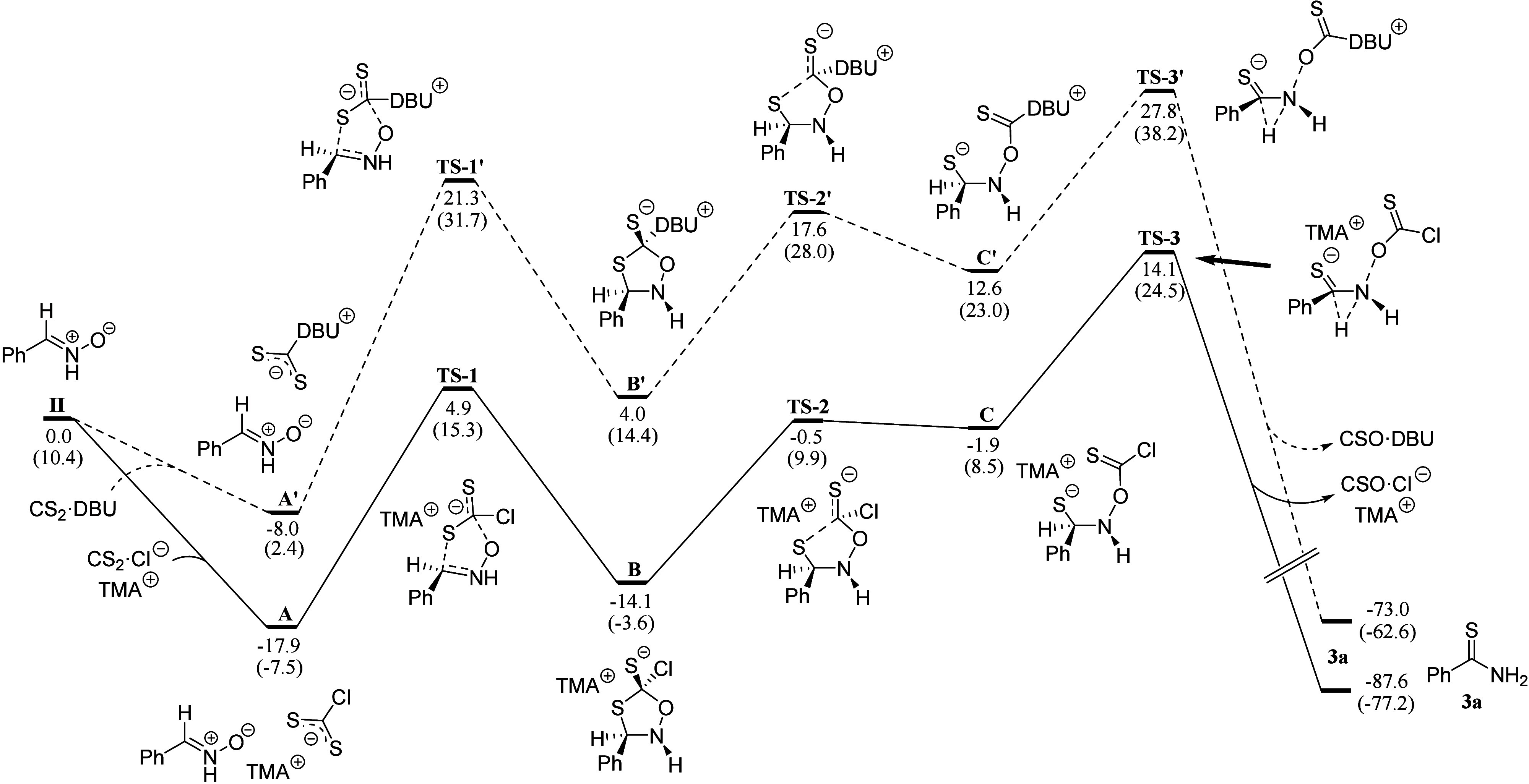
Reaction energy profile from the active species of the oxime (**II**) to **3a**. Note that the solid lines follow the path for the reaction with the [CS_2_Cl]^−^ adduct and dashed lines that when the [CS_2_·DBU] adduct is considered. Energy values correspond to the corrected Gibbs energies according to the Morokuma scheme relative to species **II** and with respect to oxime **1a** (in parentheses).

Although heterocycle **2a** could not be characterized or detected during the reaction outcome, we believe that it acts as an intermediate in the **1a** → **3a** transformation, evolving into the final product by the extrusion of CSO, as we have previously observed in related cases.^16,19^ To corroborate this hypothesis, we next investigated the reaction pathway connecting aldoxime **1a** to benzothioamide **3a** through computational studies following the procedure outlined in the Supporting Information. The reaction is initiated by the deprotonation of aldoxime **1a** by DBU, completing the tautomerism when the DBUH^+^ returns the proton to the nitrogen atom in the oxime (see Figures CS1 and CS2 for the detailed pathway followed). Tautomerism without an explicit DBU molecule implies an insurmountable 52.0 kcal/mol barrier (see Figure CS2). After tautomerization, aldoxime is transformed into a nitrone-like species, but it is never a true nitrone.^16^

As shown in Figure 1, the formation of **II** enables the insertion of a [CS_2_Cl]^−^ adduct by means of a concerted cycloaddition. This [CS_2_Cl]^−^ adduct is formed in the course of the reaction in the presence of chloride, as previously demonstrated in depth by our group.^16^ In the first stage of the reaction, the oxygen atom of the activated oxime attacks the carbon atom in the [CS_2_Cl]^−^ adduct, while one of its sulfur atoms simultaneously attacks the α-carbon of **II**, affording five-membered heterocyclic intermediate **B**. The energy barrier imposed by the cycloaddition is 22.8 kcal/mol at the DFT level. The newly formed heterocycle can evolve through C–S bond cleavage, with an energy requirement of 14.6 kcal/mol. Finally, opened intermediate **C** can evolve through **TS-3** via extrusion of the [CSOCl]^−^ adduct, which triggers the simultaneous hydrogen migration from the α-carbon to the nitrogen, which could also be mediated by DBU (see Figure CS7). The activation energy of this last step shows a value of 16.0 kcal/mol. The process yields **3a**, which displays a stability that is nearly 90 kcal/mol lower than that of activated oxime **II**. TMA (tetramethylammonium) was used as the counterion instead of TBA to facilitate the calculations, their role being analogous.

In order to clarify the relevance of TBACl (TMACl in the calculations), the utilization of the [CS_2_Cl]^−^ adduct was omitted. Instead, the activation of CS_2_ with DBU was considered. The DBU molecule mimics the role of the Cl^–^ anion. Its activation, thermodynamically feasible, imposes an energy barrier of 11.5 kcal/mol (see Figure CS4), with the formed adduct being 0.5 kcal/mol more stable than the distinct CS_2_ and DBU molecules. The process followed by the insertion of [CS_2_·DBU] is shown in Figure 1 with dashed lines. In all cases, intermediates **B′** and **C′**, which are in fact equivalent to those appearing in the case of insertion of [CSOCl]^−^, are energetically less stable. The main difference is detected in the cycloaddition, which is considered the rate-limiting step, showing an activation energy of 29.3 kcal/mol. The remaining steps display nearly identical energy barriers for both adduct insertions. Therefore, in the absence of TBACl (TMACl), the reaction may occur but with a lower yield given the higher energy requirements, being fully consistent with the concomitant and synergistic effect of DBU and TBACl observed experimentally (see Table 1).

Five-membered heterocyclic intermediate **2a** appears along the reaction pathway but in combination with the Cl^–^ anion or DBU (**B** or **B′**, respectively, in Figure 1). When bearing chloride, the intermediate naturally evolves toward ring opening and [CSO·Cl]^−^[TMA]^+^ extrusion. On the contrary, the release of DBU from **B′** and insertion are possible. The release imposes an energy barrier of less than 1 kcal/mol, which can be reversed by the same transition state (details in Figure CS5). The release, which explicitly yields **2a**, can also evolve in the presence of chloride ions, again via direct ring opening. The latter step is dominated by a high activation energy of 33.9 kcal/mol (details in the Supporting Information).

Altogether, it is worth noting that the present mechanism differs significantly from that of our previously reported chloride-catalyzed insertion of CS_2_ into nitrones.^16^ In that system, the 1,3-dipole is preformed, no base is required, and chloride acts as the sole and governing catalyst by generating the [CS_2_·Cl]^−^ adduct. Here, DBU is indispensable for generation of reactive dipole **II** via base-assisted deprotonation/tautomerization, while chloride plays a merely synergistic role, lowering the cycloaddition barrier from 29.3 to 22.8 kcal/mol relative to the [CS_2_·BDU] activaction mode. Furthermore, the product-forming events follow a different mechanistic logic. The aldoxime pathway proceeds through a concerted extrusion of [CSOCl]^−^ coupled to a C_α_ → N hydrogen migration (TS-3), whereas the nitrone pathway involves a stepwise C_α_ → S hydrogen shift followed by a separate S → N tautomerization. These distinctions define two independent mechanistic manifolds rather than a single evolving transformation, based on two distinctive substrates.

Subsequently, and to establish the scope of our transformation, a diverse set of aldoximes (**1b**–**u**) featuring various functional groups and substitution patterns were reacted with CS_2_ under the previously optimized conditions (Scheme 2). In this sense, we were pleased to find that the reactions proceeded smoothly, tolerating halide-containing substrates (**1b**–**e**), with steric effects being negligible, as illustrated by substrate **1d**. Along the same lines, strongly electron-donating groups, such as methoxy (**1g**), were likewise compatible and did not affect the reaction outcome. A similar trend was observed for strong electron-withdrawing substituents, including trifluoromethyl (**1m**), which also had no detrimental impact on reactivity.

**Scheme 2:**
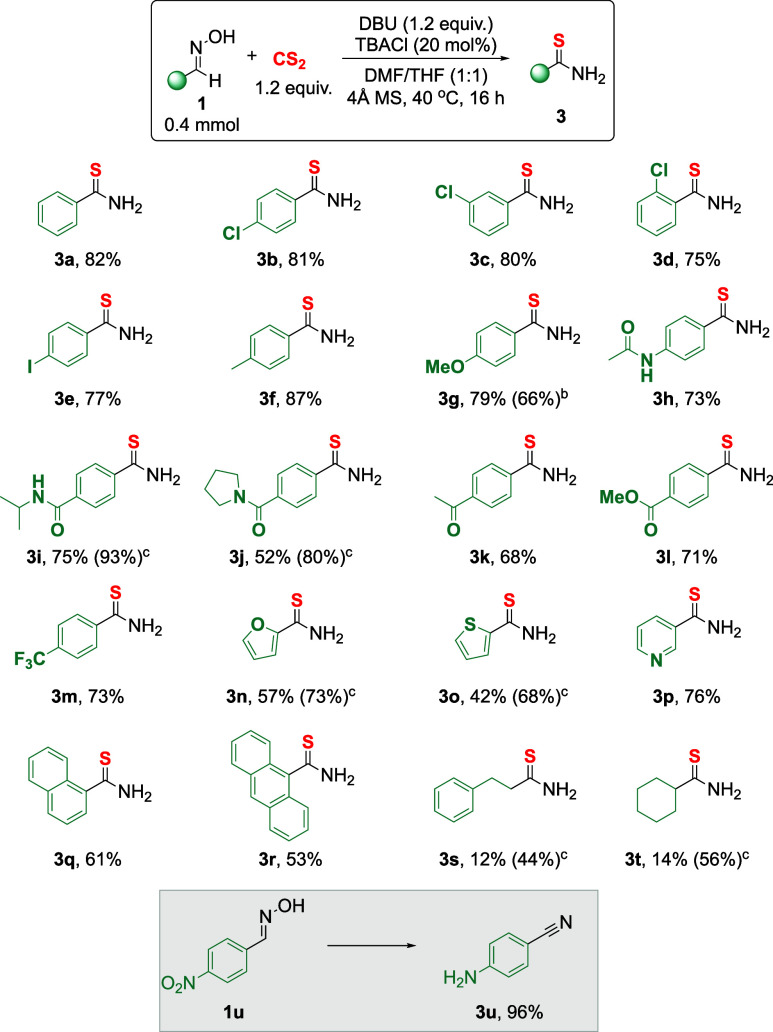
Synthesis of Primary Thioamides **3a**–**t** by Insertion of CS_2_ into Aldoximes **1a**–**t**, Respectively^a^

a To a solution of aldoxime **1a**–**u** (0.4 mmol) and TBACl (22 mg, 0.08 mmol, 0.2 equiv) in 2 mL of a 1:1 anhydrous DMF/THF mixture were sequentially added DBU (73 mg, 72 μL, 0.48 mmol, 1.2 equiv) and CS_2_ (29 μL, 0.48 mmol, 1.2 equiv). The resulting reaction mixtures were stirred at 40 °C (oil bath) for 16 h under an argon atmosphere. The isolated yields of analytically pure products, after flash chromatography, are given.
b Reaction carried out using off-the-bench (no anhydrous) solvents.
c Reaction mixture heated at 80 °C.

Importantly, the formation of primary thioamides can be accomplished with total chemoselectivity, even in the presence of other carbonyl functionalities, potentially reactive toward thionation. This is demonstrated by the successful conversion of substrates **1h**–**j** (amides), **1k** (ketone), and **1l** (ester). Notably, such substrates are typically incompatible with classical thionation methodologies, often leading to complex reaction mixtures due to the lack of carbonyl discrimination. Moreover, our methodology can cope with aldoximes containing heterocyclic scaffolds (**1n**–**p**), polyaromatic systems (**1q** and **1r**), or even aliphatic frameworks (**1s** and **1t**). This broad and structurally diverse collection of examples demonstrates the robustness, versatility, and synthetic potential of our unprecedented transformation.

Interestingly, substrate **1u** (bearing a nitro group) underwent a remarkable disproportionation reaction under our conditions, delivering nitrile **3u** in almost quantitative yield (96%). In this process, the nitro group was selectively reduced to the corresponding primary amine, while the aldoxime moiety was concomitantly oxidized to the nitrile, highlighting the complementary redox events occurring within the same molecular framework.^20^

Furthermore, to validate the robustness of our strategy, a gram-scale reaction between CS_2_ and nitrone **1a** was set up, yielding thioamide **3a** with slightly better efficiency (86% (Scheme 3a)). Also, to showcase the synthetic utility and demonstrate that our methodology can be readily applied to the synthesis of highly substituted pharmacophore scaffolds, we applied it to the synthesis of thiazole **4** from aldoxime **1p**, which was obtained in 56% overall yield (Scheme 3b). Remarkably, this transformation can be executed either stepwise or in a streamlined sequential one-pot fashion without any erosion of the overall yield. Thiazole **4** is an advanced intermediate in the synthesis of SRT2104, a selective regulator of energy homeostasis investigated for type 2 diabetes.^21^ Last but not least, we applied our methodology to the preparation of thioamide **3v** from aldoxime **1v**, a particularly challenging substrate for traditional thionation reagents, as they often struggle to achieve chemoselective conversion in the presence of nitrile groups. Resulting thioamide **3v** was subsequently further elaborated into Febuxostat (**8** (Scheme 3c)), a marketed drug used to inhibit uric acid production for the treatment of gout in adults.^22^

**Scheme 3:**
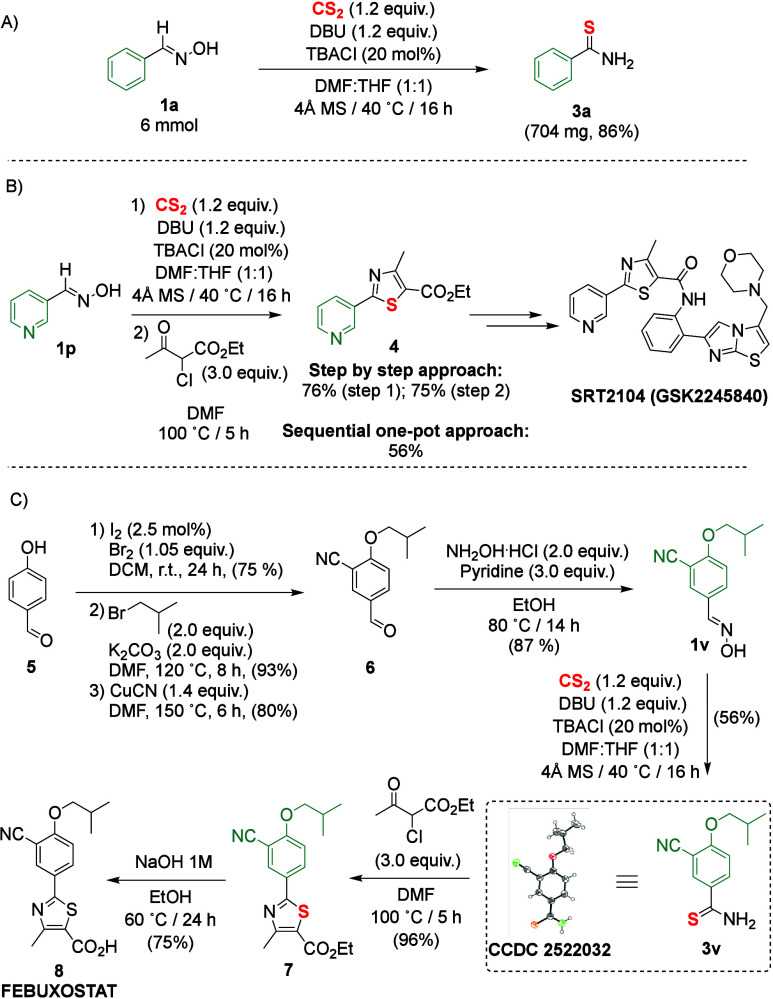
Scalability and Synthetic Application of the Insertion of CS_2_ into Aldoximes toward Pharmacophore Scaffolds

In summary, we have developed an unprecedented strategy for the synthesis of primary thioamides from aldoximes and CS_2_, promoted by DBU and improved by TBACl. Our transformation is rather straightforward, as it proceeds under mild conditions in the absence of metals or sophisticated chemicals, exhibiting high chemoselectivity and a broad tolerance for substrates. Importantly, it enables the synthesis of primary thioamides in the presence of functional groups that are sensitive to traditional thionating agents, thus implying an advantage over most previously reported methods. The synthetic utility and robustness of this method have been demonstrated in the preparation of advanced intermediates of drugs SRT2104 and Febuxostat (**8**).

Moreover, insights gained through DFT calculations have revealed the mechanism of our transformation, which involves an unprecedented and formal [1,3]-cycloaddition process between a dipole generated from the aldoximes and CS_2_. It is a reaction mode distinct from other additions of CS_2_ previously described by our group.^16,19^ More broadly, the insertion of CS_2_ into diverse organic frameworks, an area that has recently attracted our attention, is opening up uncharted avenues in synthetic organic chemistry that will be further explored by us and others in the future.

## Supplementary Material



## Data Availability

The data underlying this study are available in the published article and its Supporting Information.

## Notes

Experimental procedures, NMR data and spectra, and computational information (PDF)
