# Supplementary material for: Synthesis of Primary Thioamides by the Insertion of CS2 into Aldoximes
Source: Org Lett. 2026 Jun 8;28(24):7667–72. doi: 10.1021/acs.orglett.6c01816 (PMC13288929; doi:10.1021/acs.orglett.6c01816)
Supplement: Supplementary file 1 [file ol6c01816_si_001.pdf]

SUPPORTING INFORMATION

Synthesis of Primary Thioamides by the Insertion of CS_2 in Aldoximes

Marcos López-Aguilar, Nicolás Ríos-Lombardía, Daniel Barrena-Espés,† Miguel Gallegos,†
Joaquín García-Álvarez,* Carmen Concellón,* and Vicente del Amo*.

Laboratorio de Química Sintética Sostenible (QuimSinSos), Organic and Inorganic Chemistry Department, Instituto de Química Organometálica Enrique Moles, Universidad de Oviedo. Avenida Julián Clavería 8, 33006 Oviedo, Asturias (Spain).

Email addresses: garciajoaquin@uniovi.es; ccf@uniovi.es; vdelamo@uniovi.es

†Physical and Analytical Chemistry Department, Universidad de Oviedo. Avenida Julián Clavería 8, 33006 Oviedo, Asturias (Spain).

Table of Contents

**I. Compounds described in this contribution**	**SI_3**
**II.- General information**	**SI_5**
**III.- Optimization of the experimental procedures**	**SI_7**
**IV.- Description of aldoximes 1e, 1i-k**	**SI_17**
**V.- General procedures for the synthesis of products 3a-3u**	**SI_19**
V.1.- Standard procedure for the synthesis of thioamides **3a-3t** and spectroscopic data	SI_19
V.2.- Synthesis of 4-aminobenzonitrile **3u** and spectroscopic data	SI_28
V.3- Scale-up of the synthesis of thioamide **3a**	SI_29
**VI.- Synthesis of thiazole 4 and spectroscopic data**	**SI_30**
VI.1.- Synthesis of thiazole **4** from pyridine-3-carbothioamide **3p**	SI_30
VI.2.- One-pot sequential synthesis of thiazole **4** from 3-pyridinealdoxime **1p**	SI_31
**VII.- Total synthesis of Febuxostat**	**SI_32**
VII.1.- Synthesis of 3-bromo-4-hydroxybenzaldehyde	SI_33
VII.2.- Synthesis of 3-bromo-4-isobutoxybenzaldehyde	SI_33

VII.3.- Synthesis of 3-cyano-4-isobutoxybenzaldehyde **6**	SI_34
VII.4.- Synthesis of 3-cyano-4-isobutoxybenzaldoxime **1v**	SI_35
VII.5.- Synthesis of 3-cyano-4-isobutoxybenzothioamide **3v**	SI_36
VII.6.- Synthesis of thiazole **7**	SI_37
VII.7.- Synthesis of Febuxostat **8**	SI_38
**VIII.- X-Ray Data**	**SI_39**
**IX.- References**	**SI_42**
**X.- Copy of NMR and HRMS spectra**	**SI_44**
**XI.- Computational Supporting Information**	**SI_101**
**XII.- Cartesian coordinates of optimized structures**	**SI_108**

I.- Compounds described in this contribution.

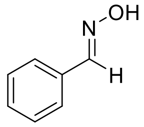

1a

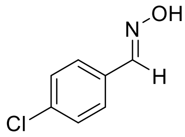

1b

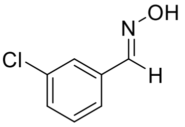

1c

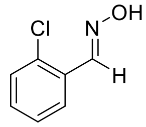

1d

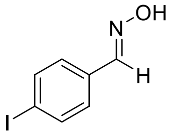

1e

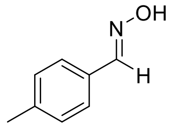

1f

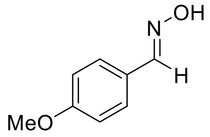

1g

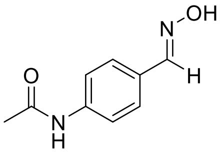

1h

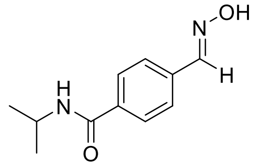

1i

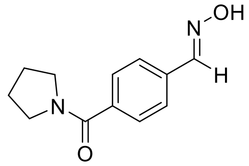

1j

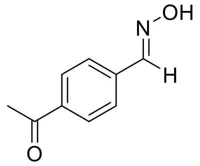

1k

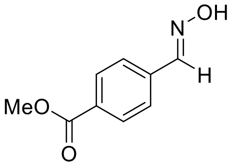

1l

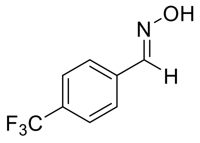

1m

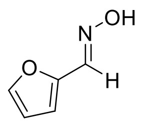

1n

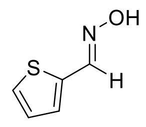

1o

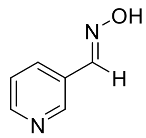

1p

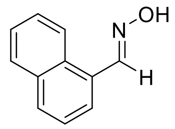

1q

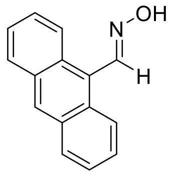

1r

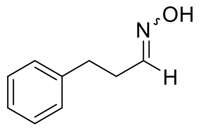

1s

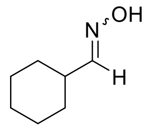

1t

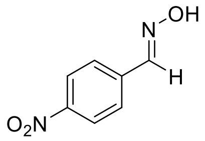

1u

Figure SI_1: Chemical structure of aldoximes studied in this contribution.

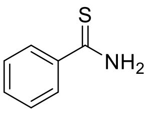

3a

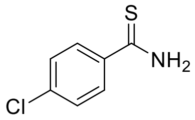

3b

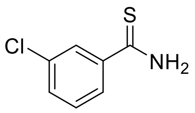

3c

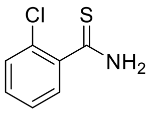

3d

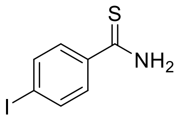

3e

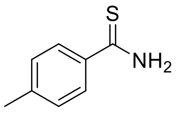

3f

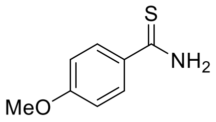

3g

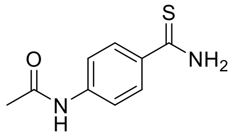

3h

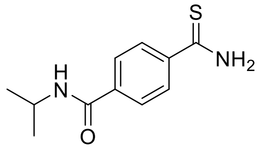

3i

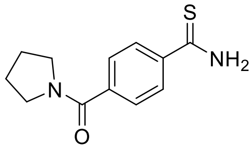

3j

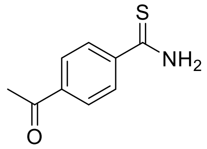

3k

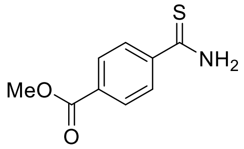

3l

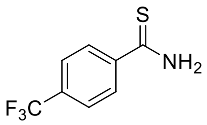

3m

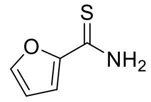

3n

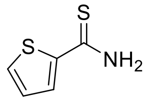

3o

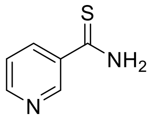

3p

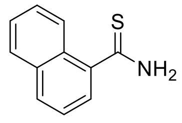

3q

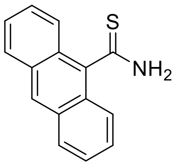

3r

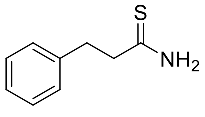

3s

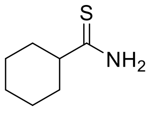

3t

Figure SI_2: Chemical structure of thioamides studied in this contribution.

II.- General information.

All commercially available reagents and solvents were used without further purification, unless otherwise noted. Commercially available tetrahydrofuran (THF) was dried by distilling from sodium/benzophenone. Dry N,N-Dimethylformamide (DMF) was purchased from Sigma-Aldrich (biotech. grade, ≥99.9% purity, <0.005% water by *Karl-Fischer* tritation) and stored under an argon atmosphere. Aldoximes **1a** and **1p** were purchased from commercially available sources. Aldoximes **1b-j**, **1m-o**, **1r** and **1u** were synthesized by refluxing (oil bath) the corresponding aldehyde (1 equiv.), hydroxylamine hydrochloride ($NH_2OH{\cdot}HCl$, 2 equiv.) and pyridine (3 equiv.) in ethanol (0.2 M) for 2 – 16 hours, (TLC monitored).[1] Aldoximes **1k-l** were synthesized by stirring the corresponding aldehyde (1 equiv.), hydroxylamine hydrochloride (1 equiv.) and pyridine (2 equiv.) in ethanol (0.2 M) for 4 days at room temperature. Aldoximes **1q** and **1s-t** were synthesized by stirring the corresponding aldehyde (1 equiv.) and hydroxylamine hydrochloride (1.6 equiv.) in a mixture methanol – pyridine (2:1, 1.3 M) for 24 hours at room temperature.[2] Aldoximes **1b-d**, **1f-h**, **1l-o** and **1q-u** are known compounds and the spectroscopic data obtained were in good agreement with those reported in the literature.[3] Aldoximes **1e** and **1i-k** are described in Section IV. Furfural was distilled under reduced pressure before used. 4-Formyl-*N*-isopropylbenzamide and 4-(pyrrolidine-1-carbonyl)benzaldehyde were respectively obtained by condensation of isopropylamine and pyrrolidine with 4-formylbenzoic acid using *N*,*N*'-dicyclohexylcarbodiimide (DCC) as a reagent. 1,8-Diazabicyclo[5.4.0]undec-7-ene (DBU) used in the synthesis of thioamides (Section V) was distilled under argon and stored over molecular sieves. Tetrabutylammonium chloride (TBACl) was carefully dried under vacuum before used and stored under argon.

Flash chromatography of reaction products was carried out using Silica gel 60, particle size 400-630 micron (VWR). Analytical thin layer chromatography (TLC) was performed on DC-Alufolien Kieselgel Silica Gel 60 F254 0.2 mm plates (Merck) and compounds were visualized by UV fluorescence or using either $KMnO_4$ or vanillin stains followed by heating.

^{1}H, ^{13}C{^{1}H}, DEPT and ^{19}F{^{1}H}-NMR spectra ($CDCl_3$, DMSO-d^6) were obtained using a Bruker NAV-300 (^{1}H, 300 MHz; ^{13}C, 75 MHz; ^{19}F, 282 MHz). All chemical shifts (δ) are given in parts per million (ppm). Calibration was made on the signal of the solvent ($CDCl_3$ ^{1}H: 7.26, ^{13}C: 77.16, DMSO-d^6 ^{1}H: 2.50; ^{13}C: 39.52).[4] Coupling constants (*J*-values) are given in hertzs (Hz). Chemical shifts are reported as follows: value (description of absorption, coupling constant(s) where applicable, number of protons). The ^{1}H-NMR spectra are presented over the 0-10 ppm region, unless significant signals of the compound are observed outside this range.

High resolution mass spectra (HRMS) experiments were carried out by ESI^+ (electrospray ionization) using a Micro TOF Q spectrometer. Melting points were taken on samples in open capillary tubes and are uncorrected.

X-Ray diffraction data were collected on an Oxford Diffraction Xcalibur Onyx Nova Geminy single crystal diffractometer (details in Section IX). Crystals were obtained by dissolving **3v** (51 mg) and **10** (25 mg) in a 5 mL of a mixture CH_2Cl_2/$CDCl_3$ (9:1) in a 6 mL vial and allowing solvents to slowly evaporate at room temperature.

III.- Optimization of the experimental procedures.

Table SI_1: Initial results in the reaction of CS_2 with (*E*)-benzaldehyde oxime **1a**.[a]

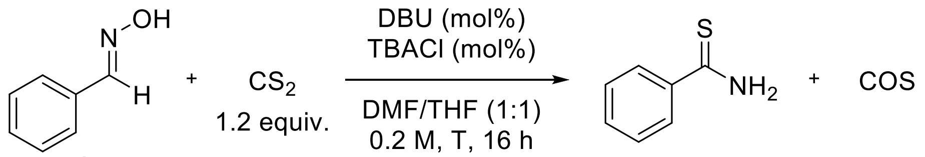

entry	T (°C)	DBU (mol%)	TBACl (mol%)	Yield (%)[b]
**1**	40	-	20	0
**2**	80	-	20	0
**3**	40	20	20	11
**4**	40	120	20	82
**5**	40	120	-	67

[a]General conditions: To a solution of (*E*)-benzaldehyde oxime **1a** (48 mg, 0.4 mmol, 1 equiv.) and the indicated amount of tetrabutylammonium chloride (TBACl) in 2 mL of a mixture of anhydrous DMF/THF (1:1), the indicated amount of 1,8-diazabicyclo[5.4.0]undec-7-ene (DBU) and CS_2 (29 µL, 0.48 mmol, 1.2 equiv.) were sequentially added and the reaction mixture was stirred at the indicated temperature (oil bath) for 16 hours under argon atmosphere. [b]Yield of pure analytical product benzothioamide **3a** isolated by flash chromatography is given.

Table SI_2: Use of different bases in the reaction of CS_2 with (*E*)-benzaldehyde oxime **1a**.[a]

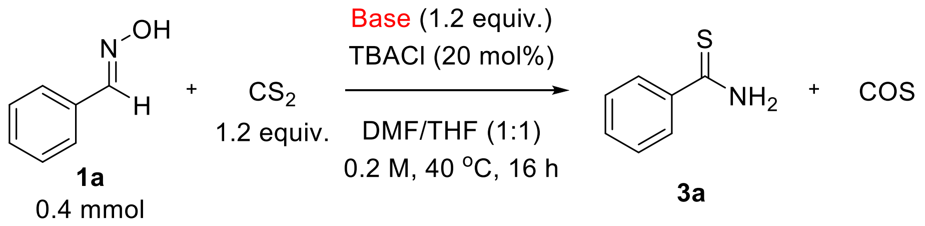

entry	Base (1.2 equiv.)	Yield (%)[b]
**1**	$NaHCO_3$	0
**2**	DMAP	0
**3**	Cs_2CO_3	20
**4**	NEt_3	Traces
**5**	DIPEA	Traces
**6**	DBU	82
**7**	TBD	60
**8**	t-BuOK	44
**9**	NaH	33

[a]General conditions: To a solution of (*E*)-benzaldehyde oxime **1a** (48 mg, 0.4 mmol, 1 equiv.) and tetrabutylammonium chloride (TBACl, 22 mg, 0.08 mmol) in 2 mL of a mixture of anhydrous DMF/THF (1:1), the indicated base (1.2 equiv.) and CS_2 (29 µL, 0.48 mmol, 1.2 equiv.) were sequentially added and the reaction mixture was stirred at 40 °C (oil bath) for 16 hours under argon atmosphere. [b]Yield of pure analytical product benzothioamide **3a** isolated by flash chromatography is given.

Table SI_3: Influence of the halogen of the tetrabutylammonium salt in the reaction of CS_2 with (*E*)-benzaldehyde oxime **1a**.[a]

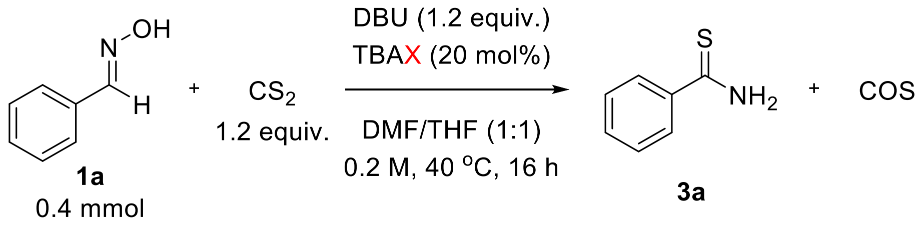

entry	TBAX (20 mol%)	Yield (%)[b]
**1**	TBAF	77
**2**	TBACl	82
**3**	TBABr	78
**4**	TBAI	65

[a]General conditions: To a solution of (*E*)-benzaldehyde oxime **1a** (48 mg, 0.4 mmol, 1 equiv.) and the indicated tetrabutylammonium salt (0.08 mmol) in 2 mL of a mixture anhydrous DMF/THF (1:1), 1,8-diazabicyclo[5.4.0]undec-7-ene (DBU, 72 µL, 0.48 mmol, 1.2 equiv.) and CS_2 (29 µL, 0.48 mmol, 1.2 equiv.) were sequentially added and the reaction mixture was stirred at 40 °C (oil bath) for 16 hours under argon atmosphere. [b]Yield of pure analytical product benzothioamide **3a** isolated by flash chromatography is given.

Table SI_4: Influence of the chloride salt in the reaction of CS_2 with (*E*)-benzaldehyde oxime **1a**.[a]

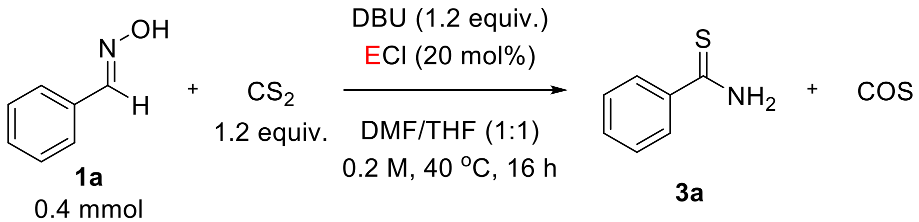

entry	ECl (20 mol%)	Yield (%)[b]
**1**	NaCl	71
**2**	LiCl	72
**3**	CsCl	72
**4**	TMACl	58
**5**	TBACl	82
**6**	TPSCl	65
**7**	PPNCl	73

[a]General conditions: To a solution of (*E*)-benzaldehyde oxime **1a** (48 mg, 0.4 mmol, 1 equiv.) and the indicated chloride salt (0.08 mmol) in 2 mL of a mixture of anhydrous DMF/THF, 1,8-diazabicyclo[5.4.0]undec-7-ene (DBU, 72 µL, 0.48 mmol, 1.2 equiv.) and CS_2 (29 µL, 0.48 mmol, 1.2 equiv.) were sequentially added and the reaction mixture was stirred at 40 °C (oil bath) for 16 hours under argon atmosphere. [b]Yield of pure analytical product benzothioamide **3a** isolated by flash chromatography is given.

Table SI_5: Influence of chloride charge in the reaction of CS_2 with (*E*)-benzaldehyde oxime **1a**.[a]

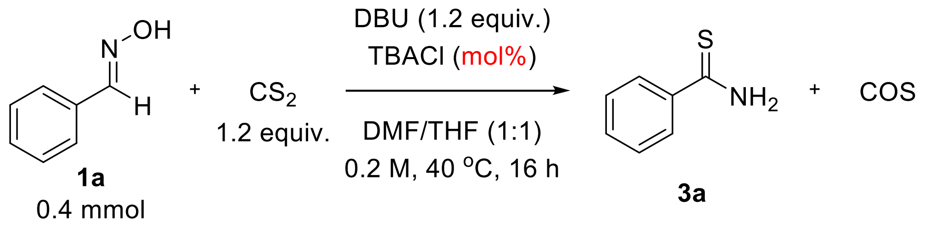

entry	TBACl (mol%)	Yield (%)[b]
**1**	10	75
**2**	20	82
**3**	120	69

[a]General conditions: To a solution of (*E*)-benzaldehyde oxime **1a** (48 mg, 0.4 mmol, 1 equiv.) and the indicated amount of tetrabutylammonium chloride in 2 mL of a mixture of anhydrous DMF/THF (1:1), 1,8-diazabicyclo[5.4.0]undec-7-ene (DBU, 72 µL, 0.48 mmol, 1.2 equiv.) and CS_2 (29 µL, 0.48 mmol, 1.2 equiv.) were sequentially added and the reaction mixture was stirred at 40 °C (oil bath) for 16 hours under argon atmosphere. [b]Yield of pure analytical product benzothioamide **3a** isolated by flash chromatography is given.

Table SI_6: Influence of equivalents of CS_2 in its reaction with (*E*)-benzaldehyde oxime **1a**.[a]

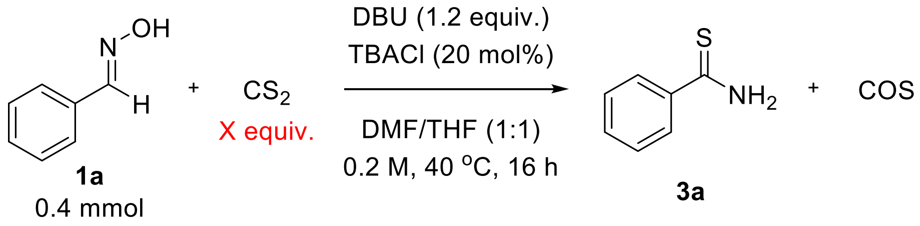

entry	CS_2 (equiv.)	Yield (%)[b]
**1**	1.2	82
**2**	2	50

[a]General conditions: To a solution of (*E*)-benzaldehyde oxime **1a** (48 mg, 0.4 mmol, 1 equiv.) and tetrabutylammonium chloride (TBACl, 22 mg, 0.08 mmol) in 2 mL of a mixture of anhydrous DMF/THF (1:1), 1,8-diazabicyclo[5.4.0]undec-7-ene (DBU, 72 µL, 0.48 mmol, 1.2 equiv.) and the indicated amount of CS_2 were sequentially added and the reaction mixture was stirred at 40 °C (oil bath) for 16 hours under argon atmosphere. [b]Yield of pure analytical product benzothioamide **3a** isolated by flash chromatography is given.

Table SI_7: Influence of equivalents of DBU in the reaction of CS_2 with (*E*)-benzaldehyde oxime **1a**.[a]

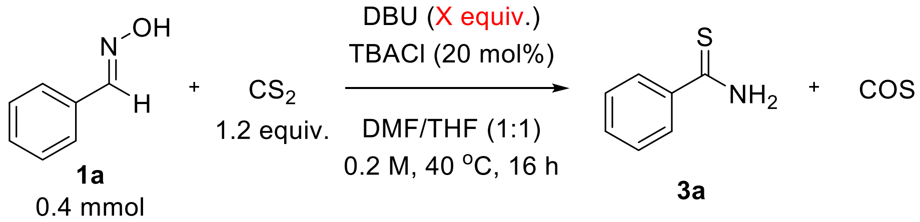

entry	DBU (equiv.)	Yield (%)[b]
**1**	0.2	11
**2**	1.2	82
**3**	2	80

[a]General conditions: To a solution of (*E*)-benzaldehyde oxime **1a** (48 mg, 0.4 mmol, 1 equiv.) and tetrabutylammonium chloride (TBACl, 22 mg, 0.08 mmol) in 2 mL of a mixture of anhydrous DMF/THF (1:1), the indicated amount of 1,8-diazabicyclo[5.4.0]undec-7-ene (DBU) and CS_2 (29 µL, 0.48 mmol, 1.2 equiv.) were sequentially added and the reaction mixture was stirred at 40 °C (oil bath) for 16 hours under argon atmosphere. [b]Yield of pure analytical product benzothioamide **3a** isolated by flash chromatography is given.

Table SI_8: Influence of solvent in the reaction of CS_2 with (*E*)-benzaldehyde oxime **1a**.[a]

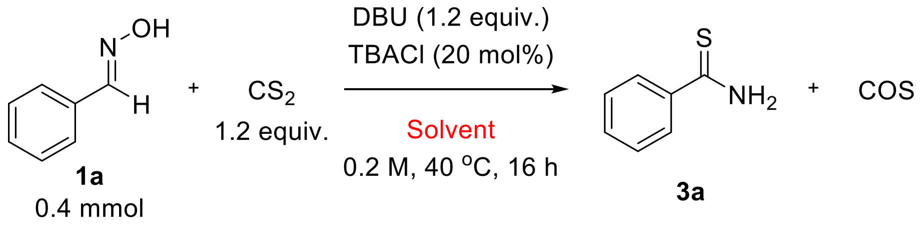

entry	Solvent	Yield (%)[b]
**1**	DMF/THF (1:1)	82
**2**	DMF	77
**3**	THF	46
**4**	2-MeTHF	31
**5**	MeCN	13
**6**	$CHCl_3$	28
**7**	Toluene	45

[a]General conditions: To a solution of (*E*)-benzaldehyde oxime **1a** (48 mg, 0.4 mmol, 1 equiv.) and tetrabutylammonium chloride (TBACl, 22 mg, 0.08 mmol) in 2 mL of the indicated anhydrous, 1,8-diazabicyclo[5.4.0]undec-7-ene (DBU, 72 µL, 0.48 mmol, 1.2 equiv.) and CS_2 (29 µL, 0.48 mmol, 1.2 equiv.) were sequentially added and the reaction mixture was stirred at 40 °C (oil bath) for 16 hours under argon atmosphere. [b]Yield of pure analytical product benzothioamide **3a** isolated by flash chromatography is given.

Table SI_9: Influence of the temperature in the reaction of CS_2 with (*E*)-benzaldehyde oxime **1a**.[a]

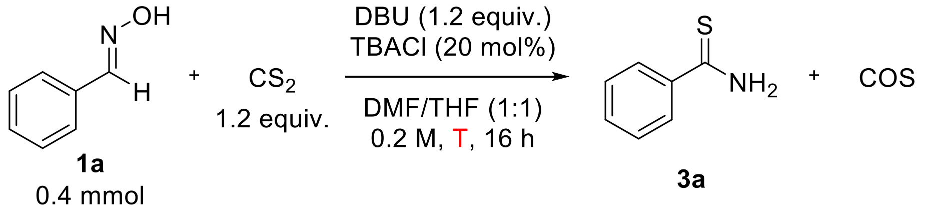

entry	T (°C)	Yield (%)[b]
**1**	25	21
**2**	40	82
**3**	60	73

[a]General conditions: To a solution of (*E*)-benzaldehyde oxime **1a** (48 mg, 0.4 mmol, 1 equiv.) and tetrabutylammonium chloride (TBACl, 22 mg, 0.08 mmol) in 2 mL of a mixture of anhydrous DMF/THF (1:1), 1,8-diazabicyclo[5.4.0]undec-7-ene (DBU, 72 µL, 0.48 mmol, 1.2 equiv.) and CS_2 (29 µL, 0.48 mmol, 1.2 equiv.) were sequentially added and the reaction mixture was stirred at the indicated temperature (oil bath) for 16 hours under argon atmosphere. [b]Yield of pure analytical product benzothioamide **3a** isolated by flash chromatography is given.

Table SI_10: Influence of concentration in the reaction of CS_2 with (*E*)-benzaldehyde oxime **1a**.[a]

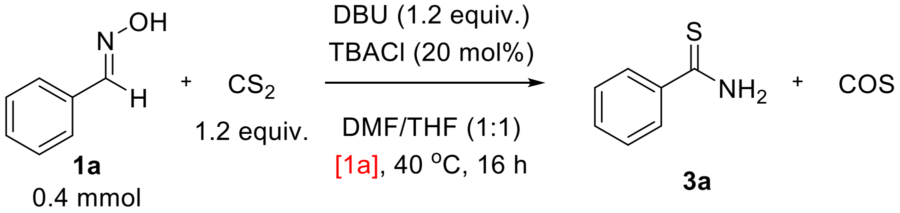

entry	[1a] (M)	Yield (%)[b]
**1**	0.1	75
**2**	0.2	82
**3**	0.4	74

[a]General conditions: To a solution of (*E*)-benzaldehyde oxime **1a** (48 mg, 0.4 mmol, 1 equiv.) and tetrabutylammonium chloride (TBACl, 22 mg, 0.08 mmol) in the appropriate volume of a mixture of anhydrous DMF/THF (1:1), 1,8-diazabicyclo[5.4.0]undec-7-ene (DBU, 72 µL, 0.48 mmol, 1.2 equiv.) and CS_2 (29 µL, 0.48 mmol, 1.2 equiv.) were sequentially added and the reaction mixture was stirred at 40 °C (oil bath) for 16 hours under argon atmosphere. [b]Yield of pure analytical product benzothioamide **3a** isolated by flash chromatography is given.

IV.- Description of aldoximes 1e, 1i-k.

4-Iodobenzaldoxime (1e)

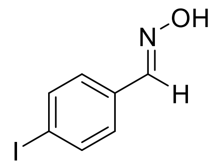

White solid (699 mg, 94% yield, reaction refluxed for 3 h, purified by flash chromatography in *n*-Hexane/EtOAc 8:1). Melting point: 127 – 129 °C. R_f (*n*-Hexane/EtOAc 6:1) = 0.35. ^{1}H NMR (300 MHz, $CDCl_3$): δ (ppm) = 8.45 (s, 1H), 8.08 (s, 1H), 7.75 – 7.70 (m, 2H), 7.32 – 7.28 (m, 2H). ^{13}C NMR (75 MHz, $CDCl_3$): 149.6 (CH), 138.0 (2CH), 131.4 (C), 128.5 (2CH), 96.2 (C). HMRS (ESI^+, m/z) calculated for $(C_7H_7INO)^+$ $[(M+H)^+]$: 247.9567; found: 247.9556.

4-(Isopropylcarbamoyl)benzaldoxime (1i)

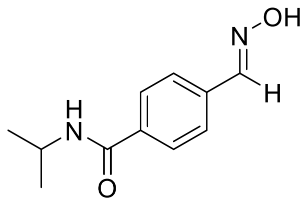

White solid (290 mg, 47% yield, reaction refluxed overnight, purified by flash chromatography in *n*-Hexane/EtOAc 2:1). Melting point: 182 – 184 °C. R_f (*n*-Hexane/EtOAc 1:1) = 0.35. ^{1}H NMR (300 MHz, DMSO-d^6): δ (ppm) = 11.41 (s, 1H), 8.22 (d, J = 7.8 Hz, 1H), 8.17 (s, 1H), 7.84 (d, J = 8.4 Hz, 2H), 7.63 (d, J = 8.4 Hz, 2H), 4.16 – 4.00 (m, 1H), 1.14 (d, J = 6.6 Hz, 6H). ^{13}C NMR (75 MHz, DMSO-d^6): 165.3 (C), 148.1 (CH), 135.9 (C), 135.7 (C), 128.1 (2CH), 126.5 (2CH), 41.5 (CH), 22.8 ($2CH_3$). HMRS (ESI^+, m/z) calculated for $(C_{11}H_{15}N_2O_2)^+$ $[(M+H)^+]$: 207.1128; found: 207.1131.

4-(Pyrrolidine-1-carbonyl)benzaldoxime (1j)

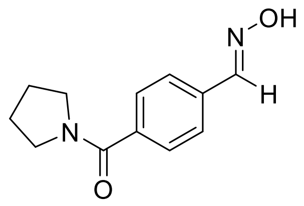

White solid (536 mg, 82% yield, reaction refluxed overnight, purified by flash chromatography in *n*-Hexane/EtOAc 1:3). Melting point: 169 – 171 °C. R_f (*n*-Hexane/EtOAc 1:3) = 0.15. ^{1}H NMR (300 MHz, DMSO-*d*^{6}): δ (ppm) = 11.38 (s, 1H), 8.16 (s, 1H), 7.62 (d, *J* = 8.3 Hz, 2H), 7.51 (d, *J* = 8.3 Hz, 2H), 3.44 (t, *J* = 6.8 Hz, 2H), 3.35 (t, *J* = 6.8 Hz, 2H), 1.89 – 1.72 (m, 4H). ^{13}C NMR (75 MHz, DMSO-*d*^{6}): 168.2 (C), 148.1 (CH), 138.2 (C), 134.7 (C), 128.0 (2CH), 126.6 (2CH), 49.3 (CH_2), 46.4 (CH_2), 26.4 (CH_2), 24.4 (CH_2). HMRS (ESI$^+$, m/z) calculated for $(C_{12}H_{15}N_2O_2)^+$ [(M+H)$^+$]: 219.1128; found: 219.1131.

4-Acetylbenzothioamide (1k)

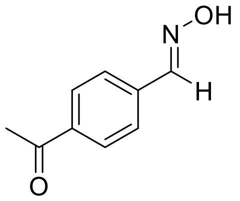

White solid (120 mg, 25% yield, purified by flash chromatography in *n*-Hexane/EtOAc 6:1 to 3:1). Melting point: 119 – 121 °C. R_f (*n*-Hexane/EtOAc 2:1) = 0.33. ^{1}H NMR (300 MHz, DMSO-*d*^{6}): δ (ppm) = 11.59 (s, 1H), 8.21 (s, 1H), 7.95 (d, *J* = 8.4 Hz, 2H), 7.70 (d, *J* = 8.4 Hz, 2H), 2.56 (s, 3H). ^{13}C NMR (75 MHz, DMSO-*d*^{6}): 197.9 (C), 148.0 (CH), 137.9 (C), 137.4 (C), 129.1 (2CH), 127.0 (2CH), 27.2 (CH_3). HMRS (ESI$^+$, m/z) calculated for $(C_9H_{10}NO_2)^+$ [(M+H)$^+$]: 164.0706; found: 164.0705.

V.- General procedures for the synthesis of products 3a-3u.

V.1.- Standard procedure for the synthesis of thioamides 3a-3t and spectroscopic data.

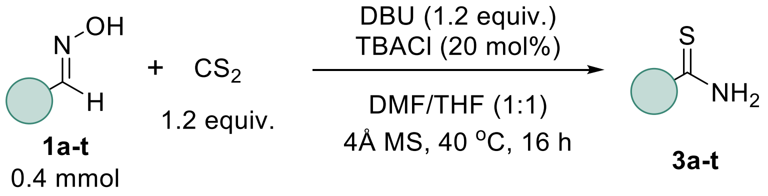

Scheme SI_1: Synthesis of thioamides **3a-t.**

To a solution of tetrabutylammonium chloride (22 mg, 0.08 mmol, 0.2 equiv.) and the corresponding aldoxime **1a-t** (0.4 mmol, 1 equiv.) in 2 mL of suspension of 4Å molecular sieves (5 pellets) in a mixture of anhydrous DMF/THF (1:1) in a 10 mL high-pressure close-capped tube, distilled 1,8-diazabicyclo[5.4.0]undec-7-ene (DBU, 72 µL, 0.48 mmol, 1.2 equiv.) and CS_2 (29 µL, 0.48 mmol, 1.2 equiv.) were sequentially added under argon and the resulting mixture was stirred for 16 hours at 40 °C (oil bath). Then, the mixture was quenched with a saturated solution of NH_4Cl (3 mL), poured in a separator funnel and extracted with EtOAc (20 mL) and distilled water (20 mL). The aqueous phase was washed twice with EtOAc (20 mL), the organic layers combined, dried with $MgSO_4$, filtered and solvent and volatiles were removed under vacuum. Finally, the crude was purified by flash chromatography on silica gel to afford primary thioamides **3a-t** (12-87% yield).

In the case of aldoximes **1i-j**, **1n-o** and **1s-t** an analogous procedure was followed stirring the reaction mixture at 80 °C (oil bath) to afford thioamides **3i-j**, **3n-o** and **3s-t** in higher yields (44-93%).

Benzothioamide (3a)

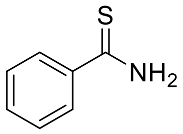

Yellow solid (45 mg, 82% yield, purified by flash chromatography in *n*-Hexane/EtOAc 3:1). ^{1}H NMR (300 MHz, $CDCl_3$): δ (ppm) = 7.96 (*br* s, 1H), 7.88 – 7.84 (m, 2H), 7.53 – 7.48 (m, 1H), 7.42 – 7.36 (m, 2H), 7.31 (*br* s, 1H). ^{13}C NMR (75 MHz, $CDCl_3$): δ (ppm) = 202.9 (C), 139.1 (C), 132.1 (CH), 128.5 (2CH), 126.9 (2CH). The spectroscopic data were matched with the one reported in the literature.[5]

4-Chlorobenzothioamide (3b)

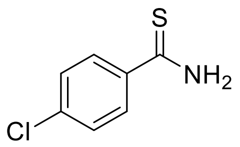

Yellow solid (56 mg, 81% yield, purified by flash chromatography in *n*-Hexane/EtOAc 3:1). ^{1}H NMR (300 MHz, DMSO-d^6): δ (ppm) = 9.92 (*br* s, 1H), 9.54 (*br* s, 1H), 7.88 (d, *J* = 8.7 Hz, 2H), 7.47 (d, *J* = 8.7 Hz, 2H). ^{13}C NMR (75 MHz, DMSO-d^6): δ (ppm) = 199.1 (C), 138.6 (C), 136.5 (C), 129.6 (2CH), 128.4 (2CH). The spectroscopic data were matched with the one reported in the literature.[6]

3-Chlorobenzothioamide (3c)

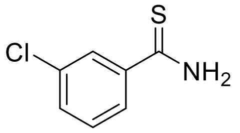

Yellow solid (55 mg, 80% yield, purified by flash chromatography in *n*-Hexane/EtOAc 3:1). ^{1}H NMR (300 MHz, $CDCl_3$): δ (ppm) = 7.86 (t, *J* = 2.0 Hz, 1H), 7.80 (*br* s, 1H), 7.71 (ddd, *J* = 7.8, 1.9, 1.1 Hz, 1H), 7.48 (ddd, *J* = 8.0, 2.1, 1.1 Hz, 1H), 7.34 (t, *J* = 7.9 Hz, 1H), 7.21 (*br* s, 1H). ^{13}C NMR (75 MHz, $CDCl_3$): δ (ppm) = 201.2 (C), 140.8 (C), 134.6 (C), 131.8 (CH), 129.8 (CH), 127.3 (CH), 124.8 (CH). The spectroscopic data were matched with the one reported in the literature.[5]

2-Chlorobenzothioamide (3d)

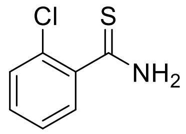

Yellow oil (52 mg, 75% yield, purified by flash chromatography in *n*-Hexane/EtOAc 3:1). ^{1}H NMR (300 MHz, $CDCl_3$): δ (ppm) = 8.01 (*br* s, 1H), 7.73 – 7.67 (m, 1H), 7.39 – 7.27 (m, 3H), 7.22 (*br* s, 1H). ^{13}C NMR (75 MHz, $CDCl_3$): δ (ppm) = 201.6 (C), 140.4 (C), 131.0 (CH), 130.5 (CH), 130.1 (CH), 128.2 (C), 127.1 (CH). The spectroscopic data were matched with the one reported in the literature.[5]

4-Iodobenzothioamide (3e)

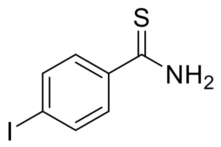

Yellow solid (81 mg, 77% yield, purified by flash chromatography in *n*-Hexane/EtOAc 3:1). ^{1}H NMR (300 MHz, DMSO-*d*^{6}): δ (ppm) = 9.89 (*br* s, 1H), 9.52 (*br* s, 1H), 7.78 (d, *J* = 8.6 Hz, 2H), 7.63 (d, *J* = 8.6 Hz, 2H). ^{13}C NMR (75 MHz, DMSO-*d*^{6}): δ (ppm) = 199.5 (C), 139.3 (C), 137.2 (2CH), 129.6 (2CH), 99.3 (C). The spectroscopic data were matched with the one reported in the literature.[6]

4-Methylbenzothioamide (3f)

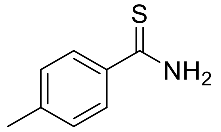

Yellow solid (52 mg, 87% yield, purified by flash chromatography in *n*-Hexane/EtOAc 3:1). ^{1}H NMR (300 MHz, $CDCl_3$): δ (ppm) = 7.81 – 7.77 (m, 2H), 7.62 (*br* s, 1H), 7.21 (d, *J* = 7.9 Hz, 2H), 7.16 (*br* s, 1H), 2.39 (s, 3H). ^{13}C NMR (75 MHz, $CDCl_3$): δ (ppm) = 202.5 (C), 142.9 (C), 136.2 (C), 129.1 (2CH), 127.0 (2CH), 21.4 (CH_3). The spectroscopic data were matched with the one reported in the literature.[5]

4-Methoxybenzothioamide (3g)

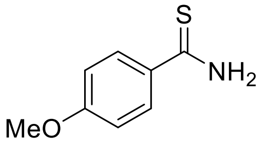

Yellow solid (53 mg, 79% yield, purified by flash chromatography in *n*-Hexane/EtOAc 2:1). ^{1}H NMR (300 MHz, $CDCl_3$): δ (ppm) = 7.92 – 7.87 (m, 2H), 7.60 (*br* s, 1H), 7.15 (*br* s, 1H), 6.92 – 6.86 (m, 2H), 3.85 (s, 3H). ^{13}C NMR (75 MHz, $CDCl_3$): δ (ppm) = 201.4 (C), 163.0 (C), 131.2 (C), 129.1 (2CH), 113.6 (2CH), 55.6 (OCH_3). The spectroscopic data were matched with the one reported in the literature.[7]

4-Acetamidobenzothioamide (3h)

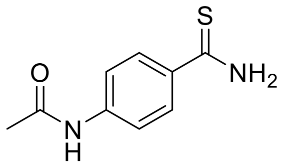

Yellow solid (57 mg, 73% yield, purified by flash chromatography in *n*-Hexane/EtOAc 1:3 to 1:5). Melting point: 240 – 242 °C. R_f (*n*-Hexane/EtOAc 1:3) = 0.14. ^{1}H NMR (300 MHz, DMSO-d^6): δ (ppm) = 10.17 (*br* s, 1H), 9.66 (*br* s, 1H), 9.32 (*br* s, 1H), 7.88 (d, *J* = 8.8 Hz, 2H), 7.57 (d, *J* = 8.8 Hz, 2H), 2.05 (s, 3H). ^{13}C NMR (75 MHz, DMSO-d^6): δ (ppm) = 199.2 (C), 169.2 (C), 142.6 (C), 133.8 (C), 128.8 (2CH), 118.1 (2CH), 24.6 (CH_3). HMRS (ESI$^+$, m/z) calculated for $(C_9H_{11}N_2OS)^+$ [$(M+H)^+$]: 195.0587; found: 195.0588.

4-(Isopropylcarbamoyl)benzothioamide (3i)

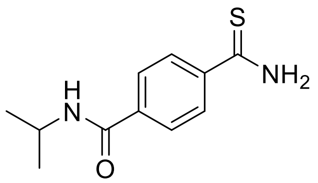

Yellow solid (67 mg, 75% yield for reaction at 40 °C; 83 mg, 93% yield for reaction at 80 °C, purified by flash chromatography in *n*-Hexane/EtOAc 1:2). Melting point: 191 – 193 °C. R_f (*n*-Hexane/EtOAc 1:2) = 0.27. ^{1}H NMR (300 MHz, DMSO-d^6): δ (ppm) = 9.95 (*br* s, 1H), 9.57 (*br* s, 1H), 8.29 (*br* d, *J* = 7.9 Hz, 1H), 7.90 (d, *J* = 8.7 Hz, 2H), 7.83 (d, *J* = 8.7 Hz, 2H), 4.16 – 4.00 (m, 1H), 1.15 (d, *J* = 6.6 Hz, 6H). ^{13}C NMR (75 MHz, DMSO-d^6): δ (ppm) = 199.8 (C), 165.1 (C), 141.8 (C), 137.3 (C), 127.5 (2CH), 127.3 (2CH), 41.6 (CH), 22.7 ($2CH_3$). HMRS (ESI$^+$, m/z) calculated for $(C_{11}H_{15}N_2OS)^+$ [$(M+H)^+$]: 223.0900; found: 223.0901.

4-(Pyrrolidine-1-carbonyl)benzothioamide (3j)

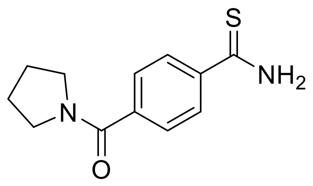

Yellow solid (49 mg, 52% yield for reaction at 40 °C; 75 mg, 80% yield for reaction at 80 °C, purified by flash chromatography in *n*-Hexane/CH_2Cl_2/EtOAc 1:3:3 to *n*-Hexane/EtOAc 1:5). Melting point: 209 – 211 °C. R_f (*n*-Hexane/EtOAc 1:5) = 0.09. ^{1}H NMR (300 MHz, DMSO-d^6): δ (ppm) = 9.94 (*br* s, 1H), 9.56 (*br* s, 1H), 7.89 (d, *J* = 8.5 Hz, 2H), 7.51 (d, *J* = 8.5 Hz, 2H), 3.45 (t, *J* = 6.8 Hz, 2H), 3.34 (t, *J* = 6.8 Hz, 2H), 1.90 – 1.75 (m, 4H). ^{13}C NMR (75 MHz, DMSO-d^6): δ (ppm) = 199.9 (C), 168.0 (C), 140.7 (C), 139.9 (C), 127.6 (2CH), 127.1 (2CH), 49.2 (CH_2), 46.4 (CH_2), 26.4 (CH_2), 24.4 (CH_2). HMRS (ESI$^+$, m/z) calculated for $(C_{12}H_{15}N_2OS)^+$ [$(M+H)^+$]: 235.0900; found: 235.0899.

4-Acetylbenzothioamide (3k)

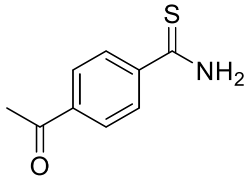

Yellow solid (49 mg, 68% yield, purified by flash chromatography in *n*-Hexane/EtOAc 3:2). Melting point: 177 – 179 °C. R_f (*n*-Hexane/EtOAc 2:1) = 0.12. ^{1}H NMR (300 MHz, DMSO-d^6): δ (ppm) = 10.03 (*br* s, 1H), 9.64 (*br* s, 1H), 7.97 – 7.90 (m, 4H), 2.59 (s, 3H). ^{13}C NMR (75 MHz, DMSO-d^6): δ (ppm) = 199.8 (C), 198.1 (C), 143.7 (C), 138.7 (C), 128.3 (2CH), 127.9 (2CH), 27.4 (CH_3). HMRS (ESI$^+$, m/z) calculated for $(C_9H_{10}NOS)^+$ [$(M+H)^+$]: 180.0478; found: 180.0477.

4-(Methoxycarbonyl)benzothioamide (3l)

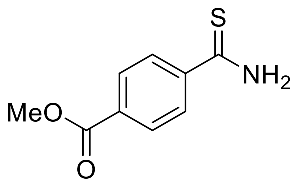

Yellow solid (55 mg, 71% yield, purified by flash chromatography in *n*-Hexane/EtOAc 2:1). ^{1}H NMR (300 MHz, DMSO-*d*^{6}): δ (ppm) = 10.05 (*br* s, 1H), 9.66 (*br* s, 1H), 7.98 – 7.89 (m, 4H), 3.85 (s, 3H). ^{13}C NMR (75 MHz, DMSO-*d*^{6}): δ (ppm) = 199.8 (C), 166.2 (C), 144.0 (C), 131.8 (C), 129.3 (2CH), 127.9 (2CH), 52.8 (OCH_3). The spectroscopic data were matched with the one reported in the literature.[8]

4-(Trifluoromethyl)benzothioamide (3m)

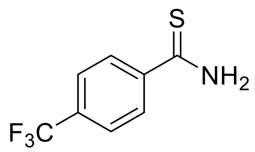

Yellow solid (60 mg, 73% yield, purified by flash chromatography in *n*-Hexane/EtOAc 3:1). ^{1}H NMR (300 MHz, DMSO-*d*^{6}): δ (ppm) = 10.10 (*br* s, 1H), 9.70 (*br* s, 1H), 7.99 (d, *J* = 8.2 Hz, 2H), 7.77 (d, *J* = 8.3 Hz, 2H). ^{19}F NMR (282 MHz, DMSO-*d*^{6}): δ (ppm) = - 61.3. ^{13}C NMR (75 MHz, DMSO-*d*^{6}): δ (ppm) = 199.5 (C), 143.8 (q, *J* = 1.4 Hz, C), 131.1 (q, *J* = 31.8 Hz, C), 128.4 (2CH), 125.4 (q, *J* = 4.0 Hz, 2CH), 124.4 (q, *J* = 272.4 Hz, CF_3). The spectroscopic data were matched with the one reported in the literature.[6]

Furan-2-carbothioamide (3n)

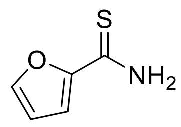

Brownish solid (29 mg, 57% yield for reaction at 40 °C; 37 mg, 73% yield for reaction at 80 °C, purified by flash chromatography in *n*-Hexane/EtOAc 3:1). Melting point: 139 – 141 °C. R_f (*n*-Hexane/EtOAc 3:1) = 0.24. ^{1}H NMR (300 MHz, DMSO-*d*^{6}): δ (ppm) = 9.56 (*br* s, 1H), 9.27 (*br* s, 1H), 7.85 (dd, *J* = 1.8, 0.8 Hz, 1H), 7.23 (dd, *J* = 3.6, 0.8 Hz, 1H), 6.60 (dd, *J* = 3.6, 1.8 Hz, 1H). ^{13}C NMR (75 MHz, DMSO-*d*^{6}): δ (ppm) = 184.8 (C), 152.3 (C), 146.2 (CH), 117.3 (CH), 113.4 (CH). HMRS (ESI^+, m/z) calculated for $(C_5H_6NOS)^+$ [$(M+H)^+$]: 128.0165; found: 128.0164.

Thiophene-2-carbothioamide (3o)

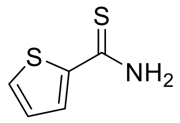

Yellow solid (24 mg, 42% yield for reaction at 40 °C; 39 mg, 68% yield for reaction at 80 °C, purified by flash chromatography in *n*-Hexane/EtOAc 3:1). Melting point: 110 – 112 °C. R_f (*n*-Hexane/EtOAc 3:1) = 0.17. ^{1}H NMR (300 MHz, DMSO-*d*^{6}): δ (ppm) = 9.59 (*br* s, 1H), 9.41 (*br* s, 1H), 7.76 (dd, *J* = 5.1, 1.2 Hz, 1H), 7.67 (dd, *J* = 3.8, 1.2 Hz, 1H), 7.13 (dd, *J* = 5.1, 3.8 Hz, 1H). ^{13}C NMR (75 MHz, DMSO-*d*^{6}): δ (ppm) = 190.2 (C), 147.0 (C), 135.3 (CH), 128.8 (CH), 126.0 (CH). HMRS (ESI$^+$, m/z) calculated for $(C_5H_6NS2)^+$ [(M+H)$^+$]: 143.9936; found: 143.9935.

Pyridine-3-carbothioamide (3p)

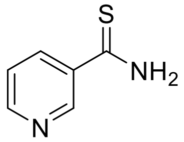

Yellow solid (42 mg, 76% yield, purified by flash chromatography in *n*-Hexane/EtOAc 1:2). ^{1}H NMR (300 MHz, DMSO-*d*^{6}): δ (ppm) = 10.05 (*br* s, 1H), 9.69 (*br* s, 1H), 8.99 – 8.96 (m, 1H), 8.64 (dd, *J* = 4.8, 2.0 Hz, 1H), 8.17 (dt, *J* = 8.0, 2.0 Hz, 1H), 7.43 (dd, *J* = 8.0, 4.8 Hz, 1H). ^{13}C NMR (75 MHz, DMSO-*d*^{6}): δ (ppm) = 198.3 (C), 152.0 (CH), 148.0 (CH), 135.6 (C), 135.3 (CH), 123.5 (CH). The spectroscopic data were matched with the one reported in the literature.[6]

Naphthalene-1-carbothioamide (3q)

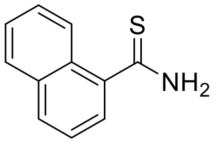

Yellow solid (46 mg, 61% yield, purified by flash chromatography in *n*-Hexane/EtOAc 3:1). ^{1}H NMR (300 MHz, DMSO-*d*^{6}): δ (ppm) = 10.21 (*br* s, 1H), 9.75 (*br* s, 1H), 8.14 – 8.07 (m, 1H), 7.97 – 7.88 (m, 2H), 7.59 – 7.39 (m, 4H). ^{13}C NMR (75 MHz, DMSO-*d*^{6}): δ (ppm) = 202.9 (C), 142.1 (C), 133.4 (C), 128.9 (CH), 128.7 (C), 128.5 (CH), 126.9 (CH), 126.6 (CH), 125.5 (2CH), 123.7 (CH). The spectroscopic data were matched with the one reported in the literature.[6]

Anthracene-9-carbothioamide (3r)

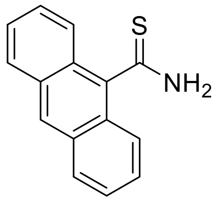

Yellow solid (50 mg, 53% yield, purified by flash chromatography in *n*-Hexane/EtOAc 4:1). Melting point: 224 – 226 °C. R_f (*n*-Hexane/EtOAc 3:1) = 0.36. ^{1}H NMR (300 MHz, DMSO-d^6): δ (ppm) = 10.60 (*br* s, 1H), 10.05 (*br* s, 1H), 8.57 (s, 1H), 8.09 – 8.05 (m, 4H), 7.60 – 7.47 (m, 4H). ^{13}C NMR (75 MHz, DMSO-d^6): δ (ppm) = 202.5 (C), 138.5 (C), 131.2 (2C), 128.6 (2CH), 126.8 (CH), 126.7 (2CH), 126.0 (2CH), 125.8 (2C), 125.8 (2CH). HMRS (ESI$^+$, m/z) calculated for $(C_{15}H_{12}NS)^+$ [$(M+H)^+$]: 238.0685; found: 238.0685.

3-Phenylpropanethioamide (3s)

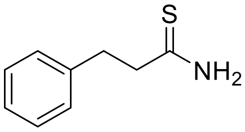

Pale yellow solid (8 mg, 12% yield for reaction at 40 °C; 29 mg, 44% yield for reaction at 80 °C, purified by flash chromatography in *n*-Hexane/EtOAc 3:1). ^{1}H NMR (300 MHz, $CDCl_3$): δ (ppm) = 7.43 (*br* s, 1H), 7.33 – 7.27 (m, 2H), 7.25 – 7.19 (m, 3H), 6.65 (*br* s, 1H), 3.12 (t, J = 7.2 Hz, 2H), 2.94 (t, J = 7.2 Hz, 2H). ^{13}C NMR (75 MHz, $CDCl_3$): 209.7 (C), 140.1 (C), 128.7 (2CH), 128.4 (2CH), 126.6 (CH), 47.0 (CH_2), 35.2 (CH_2). The spectroscopic data were matched with the one reported in the literature.[5]

Cyclohexanecarbothioamide (3t)

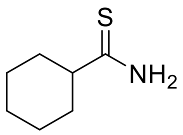

White solid (8 mg, 14% yield for reaction at 40 °C; 32 mg, 56% yield for reaction at 80 °C, purified by flash chromatography in *n*-Hexane/EtOAc 3:1). ^{1}H NMR (300 MHz, $CDCl_3$): δ (ppm) = 7.57 (*br* s, 1H), 6.85 (*br* s, 1H), 2.55 (tt, *J* = 11.7, 3.4 Hz, 1H), 1.99 – 1.91 (m, 2H), 1.87 – 1.79 (m, 2H), 1.74 – 1.67 (m, 1H), 1.51 (qd, *J* = 12.0, 2.9 Hz, 2H), 1.39 – 1.18 (m, 3H). ^{13}C NMR (75 MHz, $CDCl_3$): δ (ppm) = 215.7 (C), 53.4 (CH), 32.9 ($2CH_2$), 25.8 ($2CH_2$), 25.6 (CH_2). The spectroscopic data were matched one reported in the literature.[7]

V.2.- Synthesis of 4-aminobenzonitrile 3u and spectroscopic data.

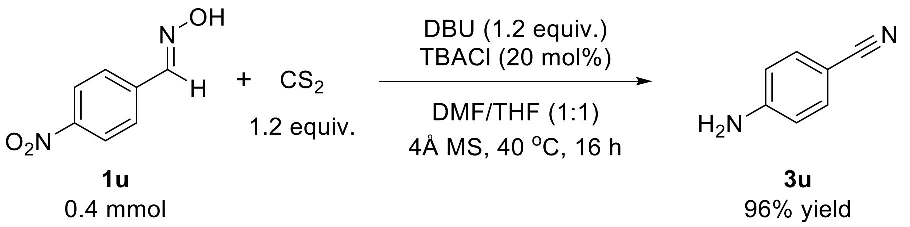

Scheme SI_2: Synthesis of 4-aminobenzonitrile **3u**.

By applying the standard procedure for the synthesis of primary thioamides (Section V.1) to 4-nitrobenzaldoxime **1u**, 4-aminobenzonitrile **3u** was obtained. Yellow solid (45 mg, 96% yield, purified by flash chromatography in *n*-Hexane/EtOAc 3:1). ^{1}H NMR (300 MHz, $CDCl_3$): δ (ppm) = 7.43 – 7.39 (m, 2H), 6.66 – 6.62 (m, 2H), 4.16 (*br* s, 2H). ^{13}C NMR (75 MHz, $CDCl_3$): 150.4 (C), 133.8 (2CH), 120.2 (C), 114.4 (2CH), 100.2 (C). The spectroscopic data were matched with the one reported in the literature.[9] Compound **3u** was not detected when the reaction was carried out in absence of CS_2.

V.3- Scale-up of the synthesis of thioamide 3a.

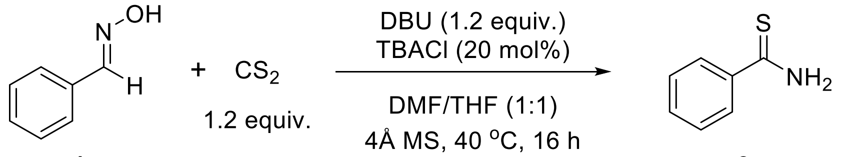

1a 6 mmol — **3a** 0.704 g, 86% yield

Scheme SI_3: Scale-up procedure for the synthesis of **3a**.

To a solution of tetrabutylammonium chloride (334 mg, 1.2 mmol, 0.2 equiv.) and benzaldoxime **1a** (727 mg, 6 mmol, 1 equiv.) in 30 mL of suspension of 4Å molecular sieves (*ca.* 30 pellets) in a mixture of anhydrous DMF/THF (1:1) in a 45 mL high-pressure close-capped tube, distilled 1,8-diazabicyclo[5.4.0]undec-7-ene (DBU, 1.075 mL, 7.2 mmol, 1.2 equiv.) and CS_2 (433 µL, 7.2 mmol, 1.2 equiv.) were sequentially added under argon and the resulting mixture was stirred for 16 hours at 40 °C (oil bath). Then, the mixture was filtered, quenched with a saturated solution of NH_4Cl (30 mL), poured in a separator funnel and extracted with EtOAc (200 mL) and distilled water (200 mL). The aqueous phase was washed twice with EtOAc (200 mL), the organic layers combined, washed with distilled water (200 mL), dried with $MgSO_4$, filtered and solvent and volatiles were removed under vacuum. Finally, the crude was purified by flash chromatography on silica gel (*n*-Hexane/EtOAc 3:1) to obtain benzothioamide **3a** as a yellow solid (0.704 g, 86% yield).

VI.- Synthesis of thiazole 4 and spectroscopic data.

VI.1.- Synthesis of thiazole 4 from pyridine-3-carbothioamide 3p.

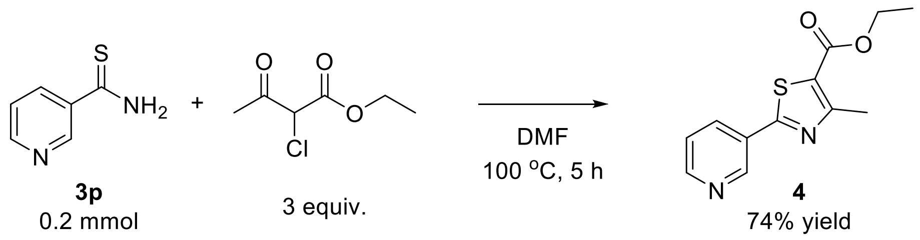

Scheme SI_4: Synthesis of thiazole **4** from thioamide **3p**.

In a 10 mL high-pressure close-capped tube, pyridine-3-carbothioamide **3p** (28 mg, 0.2 mmol, 1 equiv.) was disolved in dry DMF (2mL) and ethyl 2-chloroacetoacetate (84 µL, 0.6 mmol, 3 equiv.) was added under argon and the reaction mixture was stirred at 100 °C (oil bath) for 5 hours. Then, the mixture was allowed to reach room temperature and extracted with EtOAc (30 mL) and distilled water (30 mL). The aqueous phase was washed with EtOAc (30 mL), the organic layers combined, dried with $MgSO_4$, filtered and solvent and volatiles were removed under vacuum. Finally, the crude was purified by flash chromatography on silica gel (*n*-Hexane/EtOAc 4:1) to obtain ethyl 4-methyl-2-(pyridin-3-yl)thiazole-5-carboxylate **4** as a pale yellow solid (37 mg, 74% yield). ^{1}H NMR (300 MHz, $CDCl_3$): δ (ppm) = 9.16 (*br* s, 1H), 8.68 (*br* s, 1H), 8.24 (d, *J* = 8.1 Hz, 1H), 7.38 (dd, *J* = 8.1, 4.7 Hz, 1H), 4.35 (q, *J* = 7.2 Hz, 2H), 2.78 (s, 3H), 1.38 (t, *J* = 7.2 Hz, 3H). ^{13}C NMR (75 MHz, $CDCl_3$): 166.3 (C), 162.0 (C), 161.2 (C), 151.5 (CH), 147.9 (CH), 133.9 (CH), 129.1 (C), 123.8 (CH), 122.7 (C), 61.4 (CH_2), 17.4 (CH_3), 14.3 (CH_3). The spectroscopic data were matched with the one reported in the literature.[10]

VI.2.- One-pot sequential synthesis of thiazole 4 from 3-pyridinealdoxime 1p.

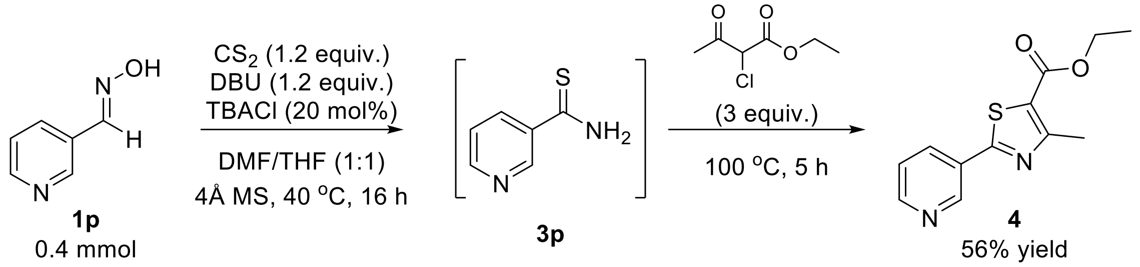

Scheme SI_5: One-pot sequential synthesis of thiazole **4** from 3-pyridinealdoxime **1p**.

To a solution of tetrabutylammonium chloride (22 mg, 0.08 mmol, 0.2 equiv.) and 3-pyridinealdoxime **1p** (49 mg, 0.4 mmol, 1 equiv.) in 2 mL of suspension of 4Å molecular sieves (5 pellets) in a mixture of anhydrous DMF/THF (1:1) in a 10 mL high-pressure close-capped tube, distilled 1,8-diazabicyclo[5.4.0]undec-7-ene (DBU, 72 µL, 0.48 mmol, 1.2 equiv.) and CS_2 (29 µL, 0.48 mmol, 1.2 equiv.) were sequentially added under argon and the resulting mixture was stirred for 16 hours at 40 °C (oil bath). After that time, ethyl 2-chloroacetoacetate (168 µL, 1.2 mmol, 3 equiv.) was added and the mixture stirred at 100 °C (oil bath) for 5 hours. Then, the crude mixture was allowed to reach room temperature, quenched with a saturated solution of NH_4Cl (3 mL), poured in a separator funnel and extracted with EtOAc (30 mL) and distilled water (30 mL). The aqueous phase was washed with EtOAc (30 mL), the organic layers combined, dried with $MgSO_4$, filtered and solvent and volatiles were removed under vacuum. Finally, the crude was purified by flash chromatography on silica gel (*n*-Hexane/EtOAc 4:1) to obtain 56 mg (56% yield) of ethyl 4-methyl-2-(pyridin-3-yl)thiazole-5-carboxylate **4**.

VII.- Total synthesis of Febuxostat.

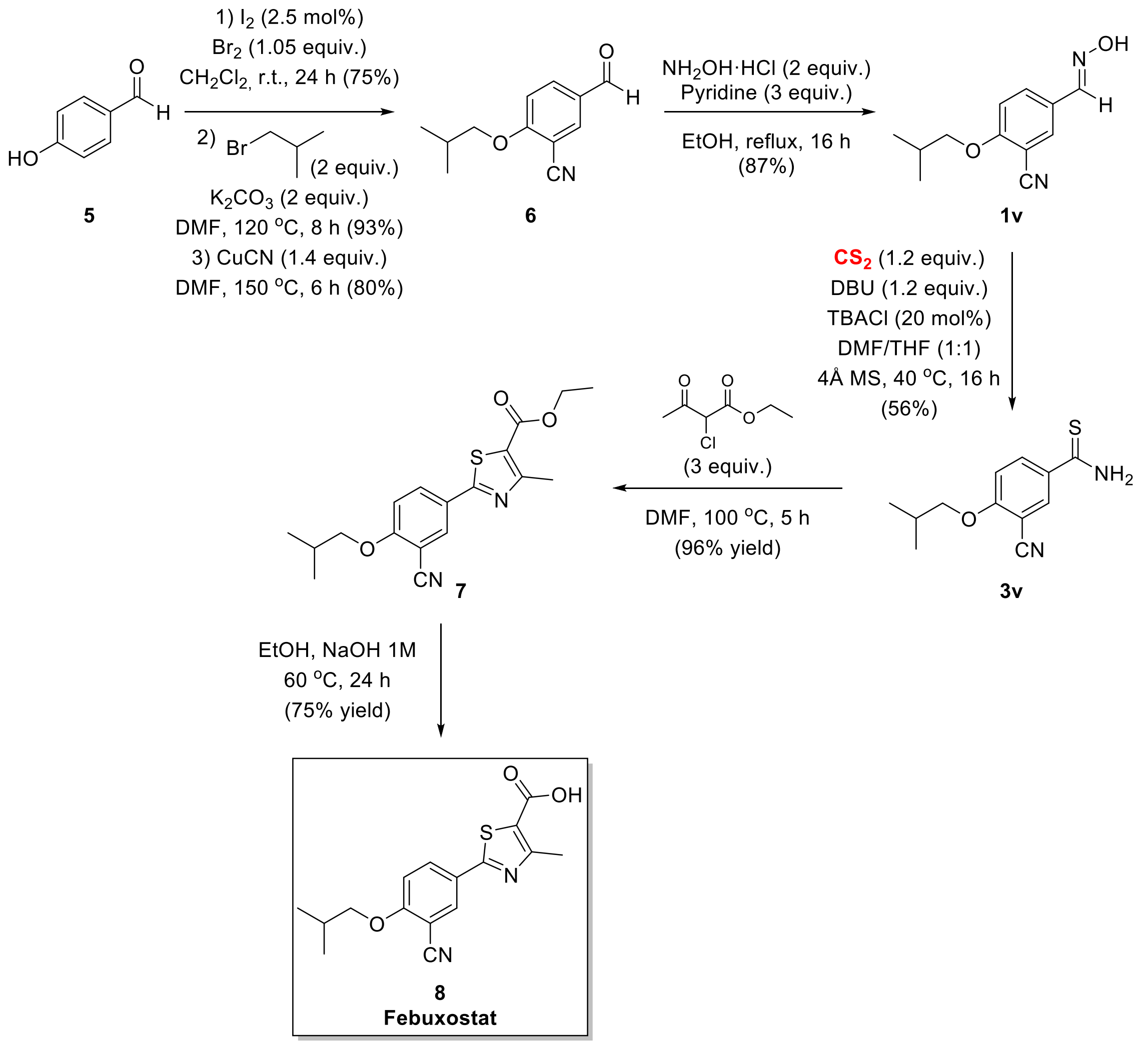

Scheme SI_6: Total synthesis of Febuxostat **8** from 4-hydroxybenzaldehyde **5**.

VII.1.- Synthesis of 3-bromo-4-hydroxybenzaldehyde.

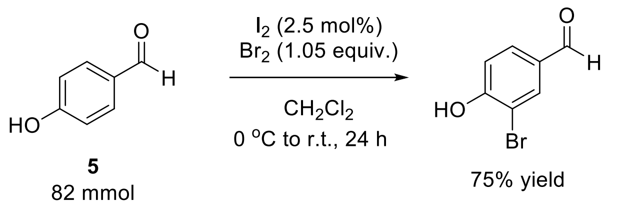

Scheme SI_7: Synthesis of 3-bromo-4-hydroxybenzaldehyde.

3-Bromo-4-hydroxybenzaldehyde was obtained by strictly following the procedure described in literature (12.30 g, 75% yield; reported 81%[11]). ^{1}H NMR (300 MHz, DMSO-*d*^{6}): δ (ppm) = 11.50 (*br* s, 1H), 9.75 (s, 1H), 8.03 – 8.01 (m, 1H), 7.73 (dd, *J* = 8.4, 2.1 Hz, 1H), 7.09 (d, *J* = 8.4 Hz, 1H). ^{13}C NMR (75 MHz, DMSO-*d*^{6}): 190.7 (CHO), 160.2 (C), 135.5 (CH), 131.0 (CH), 130.1 (C), 117.0 (CH), 110.5 (C). The spectroscopic data were matched with the one reported in the literature.[11]

VII.2.- Synthesis of 3-bromo-4-isobutoxybenzaldehyde.

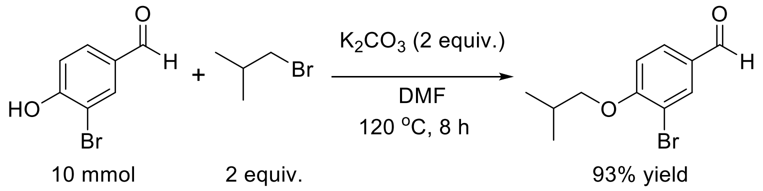

Scheme SI_8: Synthesis of 3-bromo-4-isobutoxybenzaldehyde.

A mixture of 3-bromo-4-hydroxybenzaldehyde (2.01 g, 10 mmol, 1 equiv.), 1-bromo-2-methyl-propane (2.18 mL, 20 mmol, 2 equiv.) and potassium carbonate (2.76 g, 20 mmol, 2 equiv.) in dry DMF (10 mL) in a 45 mL high-pressure close-capped tube was stirred at 120 °C (oil bath) for 8 hours under argon. Upon completion of the reaction, the mixture was diluted with EtOAc (100 mL) and filtered. The organic liquor was washed with distilled water (2 x 100 mL) and brine (100 mL), dried with $MgSO_4$, filtered and solvent and volatiles were removed under vacuum affording 3-bromo-4-isobutoxybenzaldehyde as a pale yellow solid (2.38 g, 93% yield). The product was used in the next step without further purification. ^{1}H NMR (300 MHz, $CDCl_3$): δ (ppm) = 9.82 (s, 1H), 8.06 (d, *J* = 2.1 Hz, 1H), 7.77 (dd, *J* = 8.5, 2.1 Hz, 1H), 6.95 (d, *J* = 8.5 Hz, 1H), 3.86 (d, *J* = 6.4 Hz, 2H), 2.25 – 2.12 (m, 1H), 1.08 (d, *J* = 6.7 Hz, 6H). ^{13}C NMR (75 MHz, $CDCl_3$): 189.6 (CHO), 160.3 (C), 134.5 (CH), 131.1 (CH), 130.5 (C), 113.1 (C), 112.3 (CH), 75.7 (CH_2), 28.2 (CH), 19.1 ($2CH_3$). The spectroscopic data were matched with the one reported in the literature.[12]

VII.3.- Synthesis of 3-cyano-4-isobutoxybenzaldehyde 6.

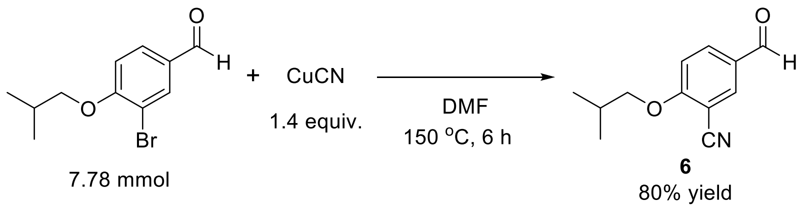

Scheme SI_9: Synthesis of 3-cyano-4-isobutoxybenzaldehyde **6**.

A mixture of 3-bromo-4-isobutoxybenzaldehyde (2.00 g, 7.78 mmol, 1 equiv.), cuprous cyanide (976 mg, 10.89 mmol, 1.4 equiv.) in dry DMF (15 mL) in a 45 mL high-pressure close-capped tube was stirred at 150 °C (oil bath) for 6 hours under argon. Upon completion of the reaction, CH_2Cl_2 (50 mL) and ammonia (aqueous solution, 28-30%, 30 mL) were added, the organic phase extracted, solvent removed under vacuum, crude dissolved in EtOAc (50 mL) and washed with distilled water (50 mL). The organic liquor was dried with $MgSO_4$, filtered and solvent and volatiles were removed under vacuum. Finally, the crude was purified by flash chromatography on silica gel (*n*-Hexane/CH_2Cl_2 1:1) to obtain 3-cyano-4-isobutoxybenzaldehyde **6** as a pale yellow solid (1.26 g, 80% yield). ^{1}H NMR (300 MHz, $CDCl_3$): δ (ppm) = 9.87 (s, 1H), 8.08 – 8.01 (m, 2H), 7.08 (d, *J* = 8.7 Hz, 1H), 3.93 (d, *J* = 6.4 Hz, 2H), 2.26 – 2.13 (m, 1H), 1.07 (d, *J* = 6.7 Hz, 6H). ^{13}C NMR (75 MHz, $CDCl_3$): 188.9 (CHO), 164.9 (C), 135.9 (CH), 135.5 (CH), 129.5 (C), 115.1 (C), 112.5 (CH), 103.1 (C), 76.0 (CH_2), 28.1 (CH), 19.0 ($2CH_3$). The spectroscopic data were matched with the one reported in the literature.[12]

VII.4.- Synthesis of 3-cyano-4-isobutoxybenzaldoxime 1v.

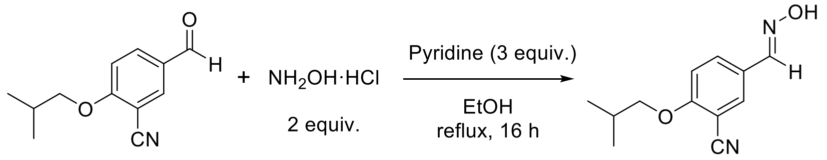

6
3 mmol

1v
87% yield

Scheme SI_10: Synthesis of 3-cyano-4-isobutoxybenzaldoxime **1v**.

To a solution of 3-cyano-4-isobutoxybenzaldehyde **6** (610 mg, 3 mmol, 1 equiv.) in ethanol (15 mL), hydroxylamine hydrochloride (417 mg, 6 mmol, 2 equiv.) and pyridine (728 µL, 9 mmol, 3 equiv.) were sequentially added and the reaction mixture stirred under reflux (heating block) for 16 hours. Then, solvent and volatiles were removed under vacuum and the crude purified by flash chromatography on silica gel (*n*-Hexane/EtOAc 4:1) to obtain 3-cyano-4-isobutoxybenzaldoxime **1v** as white solid (567 mg, 87% yield). Melting point: 92 – 94ºC. R_f (*n*-Hexane/EtOAc 6:1) = 0.18. ^{1}H NMR (300 MHz, $CDCl_3$): δ (ppm) = 8.51 (*br* s, 1H), 8.06 (s, 1H), 7.76 – 7.70 (m, 2H), 6.95 (d, *J* = 8.8 Hz, 1H), 3.85 (d, *J* = 6.5 Hz, 2H), 2.23 – 2.10 (m, 1H), 1.06 (d, *J* = 6.7 Hz, 6H). ^{13}C NMR (75 MHz, $CDCl_3$): δ (ppm) = 161.8 (C), 148.1 (CH), 132.8 (CH), 132.2 (CH), 125.0 (C), 115.8 (C), 112.6 (CH), 102.6 (C), 75.5 (CH_2), 28.1 (CH), 19.0 ($2CH_3$). HMRS (ESI^+, m/z) calculated for $(C_{12}H_{15}N_2O_2)^+$ [$(M+H)^+$]: 219.1128; found: 219.1132.

VII.5.- Synthesis of 3-cyano-4-isobutoxybenzothioamide 3v.

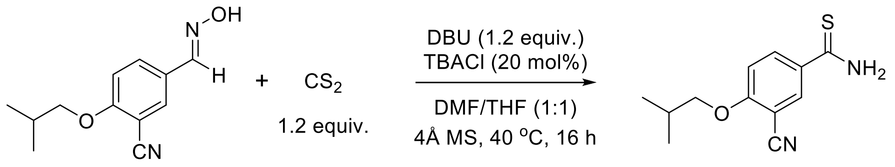

1v
0.4 mmol

3v
56% yield

Scheme SI_11: Synthesis of 3-cyano-4-isobutoxybenzothioamide **3v**.

3-Cyano-4-isobutoxybenzothioamide **3v** was obtained by strictly following the procedure for the synthesis of primary thioamides from aldoximes described in Section V.1. Yellow solid (53 mg, 56% yield, purified by flash chromatography in *n*-Hexane/EtOAc 3:1). Melting point: 133 – 135 °C. R_f (*n*-Hexane/EtOAc 3:1) = 0.14. ^{1}H NMR (300 MHz, DMSO-d^6): δ (ppm) = 9.89 (*br* s, 1H), 9.49 (*br* s, 1H), 8.26 – 8.20 (m, 2H), 7.29 – 7.26 (m, 1H), 3.97 (d, *J* = 6.5 Hz, 2H), 2.12 – 1.99 (m, 1H), 0.99 (d, *J* = 6.8 Hz, 6H). ^{13}C NMR (75 MHz, DMSO-d^6): δ (ppm) = 197.0 (C), 162.7 (C), 135.3 (CH), 132.8 (CH), 132.0 (C), 116.2 (C), 112.9 (CH), 100.3 (C), 75.6 (CH_2), 28.0 (CH), 19.5 ($2CH_3$). HMRS (ESI^+, m/z) calculated for $(C_{12}H_{15}N_2OS)^+$ $[(M+H)^+]$: 235.0900; found: 235.0901. The structure of the compound has been unambiguously determined through single-crystal X-ray diffraction analysis (see Section IX).

VII.6.- Synthesis of thiazole 7.

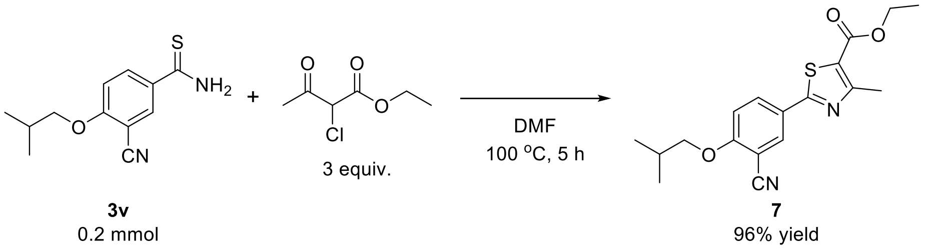

Scheme SI_12: Synthesis of thiazole **7**.

Ethyl 2-(3-cyano-4-isobutoxyphenyl)-4-methylthiazole-5-carboxylate **7** was obtained following the analogous procedure described for the synthesis of thiazole **4** (Section VI.1). White solid (66 mg, 96% yield, purified by flash chromatography in *n*-Hexane/EtOAc 10:1 to 6:1). ^{1}H NMR (300 MHz, $CDCl_3$): δ (ppm) = 8.15 (d, *J* = 2.3 Hz, 1H), 8.07 (dd, *J* = 8.8, 2.3 Hz, 1H), 7.00 (d, *J* = 8.8 Hz, 1H), 4.34 (q, *J* = 7.1 Hz, 2H), 3.89 (d, *J* = 6.5 Hz, 2H), 2.75 (s, 3H), 2.26 – 2.13 (m, 1H), 1.38 (t, *J* = 7.1 Hz, 3H), 1.08 (d, *J* = 6.7 Hz, 6H). ^{13}C NMR (75 MHz, $CDCl_3$): 167.1 (C), 162.5 (C), 162.0 (C), 161.1 (C), 132.5 (CH), 132.1 (CH), 126.1 (C), 121.9 (C), 115.4 (C), 112.6 (CH), 103.0 (C), 75.7 (CH_2), 61.3 (CH_2), 28.2 (CH), 19.0 ($2CH_3$), 17.4 (CH_3), 14.3 (CH_3). The spectroscopic data were matched with the one reported in the literature.[13]

VII.7.- Synthesis of Febuxostat 8.

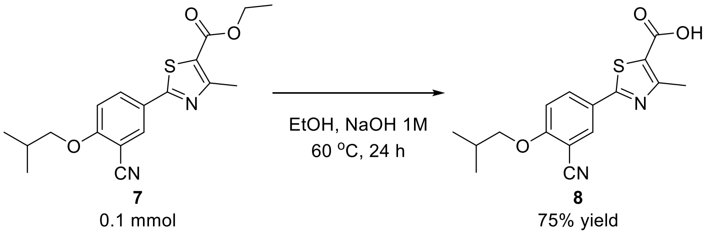

Scheme SI_13: Final step in the synthesis of Febuxostat **8**.

A suspension of ethyl 2-(3-cyano-4-isobutoxyphenyl)-4-methylthiazole-5-carboxylate **7** (34 mg, 0.1 mmol) in EtOH (1 mL) in a 10 mL high-pressure close-capped tube was stirred at 60 °C (oil bath) for 10 minutes before a 1M aqueous solution of NaOH (1 mL) was added dropwise at the same temperature. Then, the reaction mixture was vigorously stirred at 60 °C (oil bath) for 24 hours. After that time, the mixture was allowed to reach room temperature and quenched dropwise with a 1M aqueous solution of HCl (1 mL), leading to the precipitation of a solid. Then, CH_2Cl_2 (30 mL) and distilled water (30 mL) were added, the aqueous phase saturated with brine, the organic phase extracted, and the aqueous phase washed with CH_2Cl_2 (30 mL). Subsequently, the organic liquors were combined, dried with $MgSO_4$, filtered and solvent and volatiles were removed under vacuum. Finally, the crude was purified by flash chromatography on silica gel (CH_2Cl_2/MeOH 97:3) to obtain 2-(3-cyano-4-isobutoxyphenyl)-4-methylthiazole-5-carboxylic acid (Febuxostat, **8**) as a pale yellow solid (24 mg, 75% yield). ^1H NMR (300 MHz, $CDCl_3$): δ (ppm) = 8.18 (d, *J* = 2.4 Hz, 1H), 8.08 (dd, *J* = 8.9, 2.4 Hz, 1H), 7.01 (d, *J* = 8.9 Hz, 1H), 3.90 (d, *J* = 6.5 Hz, 2H), 2.78 (s, 3H), 2.27 – 2.13 (m, 1H), 1.09 (d, *J* = 6.7 Hz, 6H). ^{13}C NMR (75 MHz, $CDCl_3$): 168.5 (C), 166.9 (C), 162.8 (C), 162.7 (C), 132.7 (CH), 132.2 (CH), 125.8 (C), 115.3 (C), 112.7 (CH), 103.1 (C), 75.8 (CH_2), 28.2 (CH), 19.0 ($2CH_3$), 17.6 (CH_3). The spectroscopic data were matched with the one reported in the literature.[14]

VIII.- X-Ray Data.

A crystal of **3v** (Figure SI_ 3) was analysed by X-ray diffraction. A selection of crystal, measurement and refinement data is given in Table SI_11. Diffraction data were collected on an Oxford Diffraction Xcalibur Onyx Nova Geminy single crystal diffractometer. Empirical absorption corrections were applied using the SCALE3 ABSPACK algorithm as implemented in Chrysalis RED.[15] The structure was solved with SIR-2019.[16] Isotropic and full matrix anisotropic least square refinements were carried out using SHELXL-2018/3.[17] All non-H atoms were refined anisotropically. All H atoms were set in calculated positions and were refined riding on their parent atoms except for those of the NH_2 group**.** The nitrogen atom of the nitrile group was disordered over two positions with a 63:37 occupancy ratio. The WINGX program system (version 2021.1)[18] was used throughout the structure determination. The molecular plots were made with ORTEP.[18] The X-ray crystallographic coordinates for the structure of **3v** reported in this study have been deposited at the Cambridge Crystallographic Data Centre (CCDC) under the deposition number 2522032. These data can be obtained free of charge via http://www.ccdc.cam.ac.uk/data_request/cif.

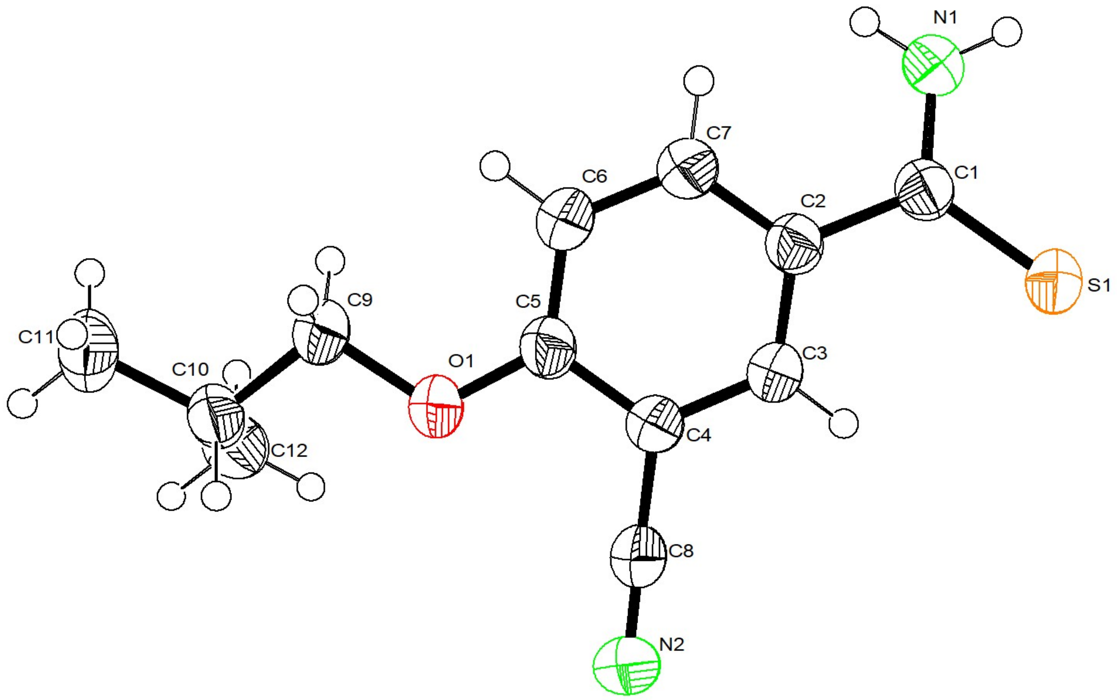

Figure SI_3: SCXRD molecular structure of **3v** (50% displacement ellipsoids).

Table SI_11. Crystal, measurement and refinement data for the compound **3v** studied by X-ray diffraction.

	**3v**
formula	$C_{12}H_{14}N_2OS$
fw	234.31
cryst syst	monoclinic
space group	P21/c
a, Å	5.76540(10)
b, Å	13.30730(10)
c, Å	16.5185(2)
α, deg	90
β, deg	99.8080(10)
γ, deg	90
V, Å^3	1248.81(3)
Z	4
F(000)	496
D_{calcd}, g cm^{-3}	1.246
μ, mm^{-1} (CuKα)	2.149
cryst size, mm	0.38 x 0.29 x 0.17
T, K	298(2)
ϑ range, deg	4.26 to 69.36
min./max. h, k, l	-5/7, -15/16, -20/19
no. collected reflns	13328
no. unique reflns	2331
no. reflns with $I > 2\sigma(I)$	2183
no. params/restraints	164/0
GOF (on F^2)	1.075
R_1 (on F, $I > 2\sigma(I)$)	0.035
wR_2 (on F^2, all data)	0.104
min./max. $\Delta\rho$, e Å^{-3}	-0.269/0.146
CCDC dep. no.	2522032

IX.- References.

[1] Correia Cordeiro, R. S.; Ríos-Lombardía, N.; Morís, F.; Kourist, R.; González-Sabín, J. One-pot transformation of ketoximes into optically active alcohols and amines by sequential action of laccases and ketoreductases or ω-transaminases. *ChemCatChem* **2019**, *11*, 1272-1277. DOI: 10.1002/cctc.201801900.

[2] González-Liste, P. J.; Cadierno, V.; García-Garrido, S. E. Catalytic rearrangement of aldoximes to primary amides in environmentally friendly media under thermal and microwave heating: another application of the bis(allyl)-ruthenium(iv) dimer [{RuCl(μ-Cl)(η^3:η^3-$C_{10}H_{16}$)}$_2$]. *ACS Sustainable Chem. Eng.* **2015**, *3*, 3004–3011. DOI: 10.1021/acssuschemeng.5b01107.

[3] a) Aldoximes **1b-d**, **1g**, **1m**, **1o**, **1s** described in Hyodo, K.; Togashi, K.; Oishi, N.; Hasegawa, G.; Uchida, K. Brønsted acid catalyzed transoximation reaction: synthesis of aldoximes and ketoximes without use of hydroxylamine salts. *Green Chem.* **2016**, *18*, 5788-5793. DOI: 10.1039/c6gc02156e. b) Aldoximes **1f**, **1t-u** described in Minakata, S.; Okumura, S.; Nagamachi, T.; Takeda, Y. Generation of nitrile oxides from oximes using *t*-BuOI and their cycloaddition. *Org. Lett.* **2011**, *13*, 2966–2969. DOI: 10.1021/ol2010616. c) Aldoximes **1h**, **1l** described in Yang, S.; Wang, Y.; Xu, W.; Tian, X.; Bao, M.; Yu, X. Visible-light-driven iron-catalyzed decarboxylative C–N coupling reaction of alkyl carboxylic acids with $NaNO_2$. *Org. Lett.* **2023**, *25*, 8834–8838. DOI: 10.1021/acs.orglett.3c03526. d) Aldoximes **1n**, **1q-r** described in Yu, J.; Jin, Y.; Lu, M. 3-Methyl-4-oxa-5-azahomoadamantane as an organocatalyst for the aerobic oxidation of primary amines to oximes in water. *Adv. Synth. Catal.* **2015**, *357*, 1175-1180. DOI: 10.1002/adsc.201400601.

[4] Gottlieb, H. E.; Kotlyar, V.; Nudelman, A. NMR chemical shifts of common laboratory solvents as trace impurities. *J. Org. Chem.* **1997,** *62,* 7512-7515. DOI: 10.1021/jo971176v.

[5] Kaboudin, B.; Yarahmadi, V.; Kato, J.; Yokomatsu, T. A simple and novel method for the direct conversion of carboxylic acids into thioamides. *RSC Adv.* **2013**, *3*, 6435-6441. DOI: 10.1039/c3ra23414b.

[6] Cao, X.-T.; Qiao, L.; Zheng, H.; Yang, H.-Y.; Zhang, P.-F. A efficient protocol for the synthesis of thioamides in [DBUH][OAc] at room temperature. *RSC Adv.* **2018**, *8*, 170-175. DOI: 10.1039/c7ra11259a.

[7] Orr, D.; Tolfrey, A.; Percy, J. M.; Frieman, J.; Harrison, Z. A.; Campbell-Crawford, M.; Patel, V. K. Single-step microwave-mediated synthesis of oxazoles and thiazoles from 3-oxetanone: a synthetic and computational study. *Chem. Eur. J.* **2013**, *19*, 9655-9662. DOI: 10.1002/chem.201301011.

[8] Chegaev, K.; Rolando, B.; Cortese, D.; Gazzano, E.; Buondonno, I.; Lazzarato, L.; Fanelli, M.; Hattinger, C. M.; Serra, M.; Riganti, C.; Fruttero, R.; Ghigo, D.; Gasco, A. H_2S-donating doxorubicins may overcome cardiotoxicity and multidrug resistance. *J. Med. Chem.* **2016**, *59*, 4881-4889. DOI: 10.1021/acs.jmedchem.6b00184.

[9] Zhang, L.; Ping, L.; Wang, Y. $Cu(NO_3)_2 \cdot 3H_2O$-mediated cyanation of aryl iodides and bromides using DMF as a single surrogate of cyanide. *Chem. Commun.* **2015**, *51*, 2840-2843. DOI: 10.1039/c4cc08579e.

[10] Dhumal, S. T.; Deshmukh, A. R.; Bhosle, M. R.; Khedkar, V. M.; Nawale, L. U.; Sarkar, D.; Mane, R. A. Synthesis and antitubercular activity of new 1,3,4-oxadiazoles bearing pyridyl and thiazolyl scaffolds. *Bioorg. Med. Chem. Lett.* **2016**, *26*, 3646-3651. DOI: 10.1016/j.bmcl.2016.05.093.

[11] Zhang, T.; Lv, Y.; Lei, Y.; Liu, D.; Feng, Y.; Zhao, J.; Chen, S.; Meng, F.; Wang, S. Design, synthesis and biological evaluation of 1-hydroxy-2-phenyl-4-pyridyl-1*H*-imidazole derivatives as xanthine oxidase inhibitors. *Eur. J. Med. Chem.* **2018**, *146*, 668-677. DOI: 10.1016/j.ejmech.2018.01.060.

[12] Chen, S.; Zhang, T.; Wang, J.; Wang, F.; Niu, H.; Wu, C.; Wang, S. Synthesis and evaluation of 1-hydroxy/methoxy-4-methyl-2-phenyl-1*H*-imidazole-5-carboxylic acid derivatives as non-purine xanthine oxidase inhibitors. *Eur. J. Med. Chem.* **2015**, *103*, 343-353. DOI: 10.1016/j.ejmech.2015.08.056.

[13] Komiyama, M.; Tsuchiya, H.; Teramoto, M.; Yajima, N.; Kurokawa, M.; Minamizono, K.; Tsuchiya, N.; Kato, Y.; Sato, Y.; Dohi, M. Process development of febuxostat using palladium- and copper-catalyzed C–H arylation. *Org. Process Res. Dev.* **2018**, *22*, 1306-1311. DOI: 10.1021/acs.oprd.8b00164

[14] Cavinet, J.; Yamaguchi, J.; Ban, I.; Itami, K. Nickel-catalyzed biaryl coupling of heteroarenes and aryl halides/triflates. *Org. Lett.* **2009**, *11*, 1733-1736. DOI: 10.1021/ol9001587.

[15] *CrysAlisPro RED, version 1.171.38.46: Oxford Diffraction Ltd, Oxford, U.K., 2015.*

[16] Burla, M. C.; Caliandro, R.; Carrozzini, B.; Cascarano, G. L.; Cuocci, C.; Giacovazzo, C.; Mallamo, M.; Mazzone, A.; Polidori, G. Crystal structure determination and refinement via *SIR2014*. *J. Appl. Crystallogr.* **2015**, *48*, 306-309. DOI: 10.1107/S1600576715001132.

[17] Sheldrick, G. M. Crystal structure refinement with *SHELXL*. *Acta Cryst.* **2015**, *C71*, 3-8. DOI: 10.1107/S2053229614024218.

[18] Farrugia, L. J. *WinGX* and *ORTEP* for Windows: an update. *J. Appl. Cryst.* **2012**, *45*, 849-854. DOI: 10.1107/S0021889812029111.

X.- Copy of NMR and HRMS spectra.

4-Iodobenzaldoxime (1e)

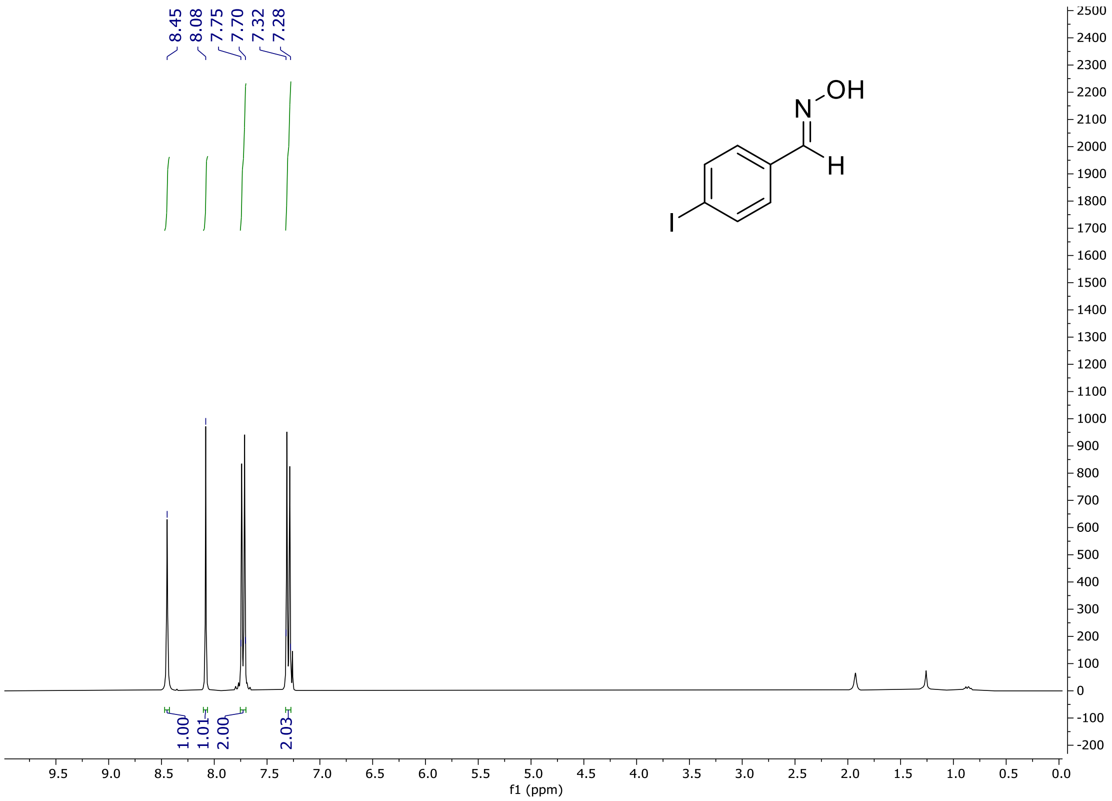

Figure SI_4: ^{1}H-NMR for **1e** in $CDCl_3$ (300 MHz).

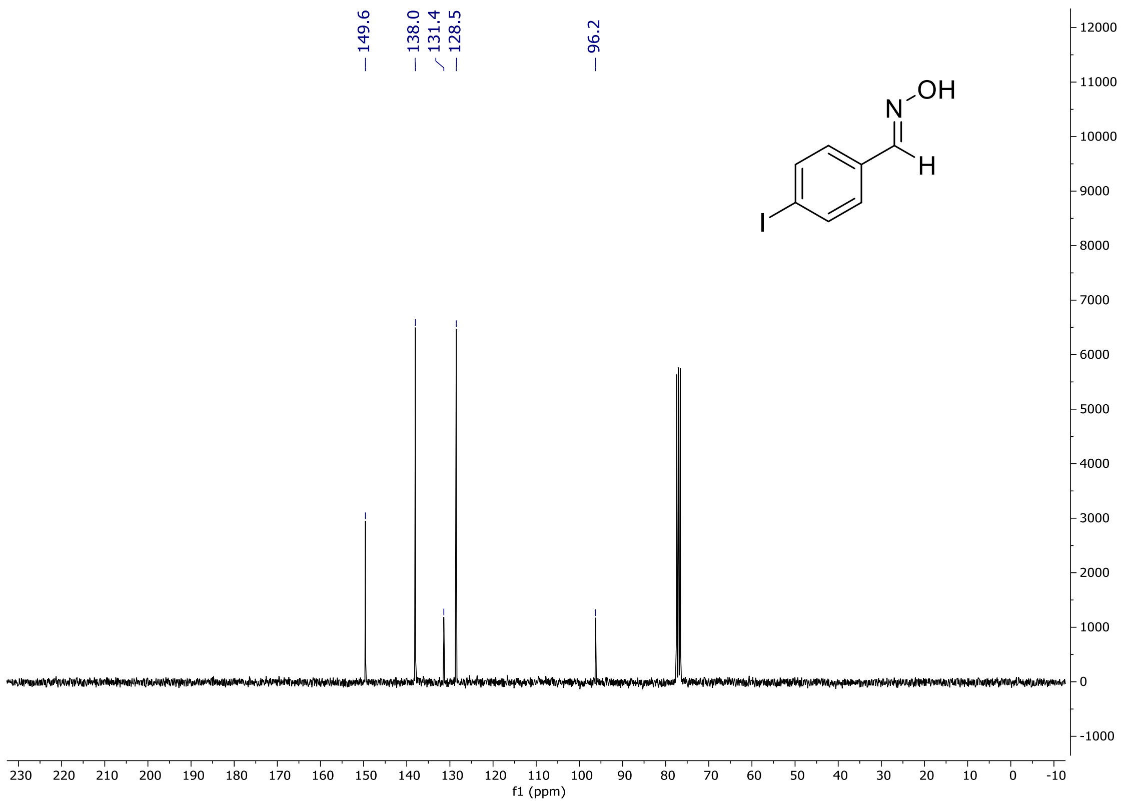

Figure SI_5: ^{13}C-NMR for **1e** in $CDCl_3$ (75 MHz).

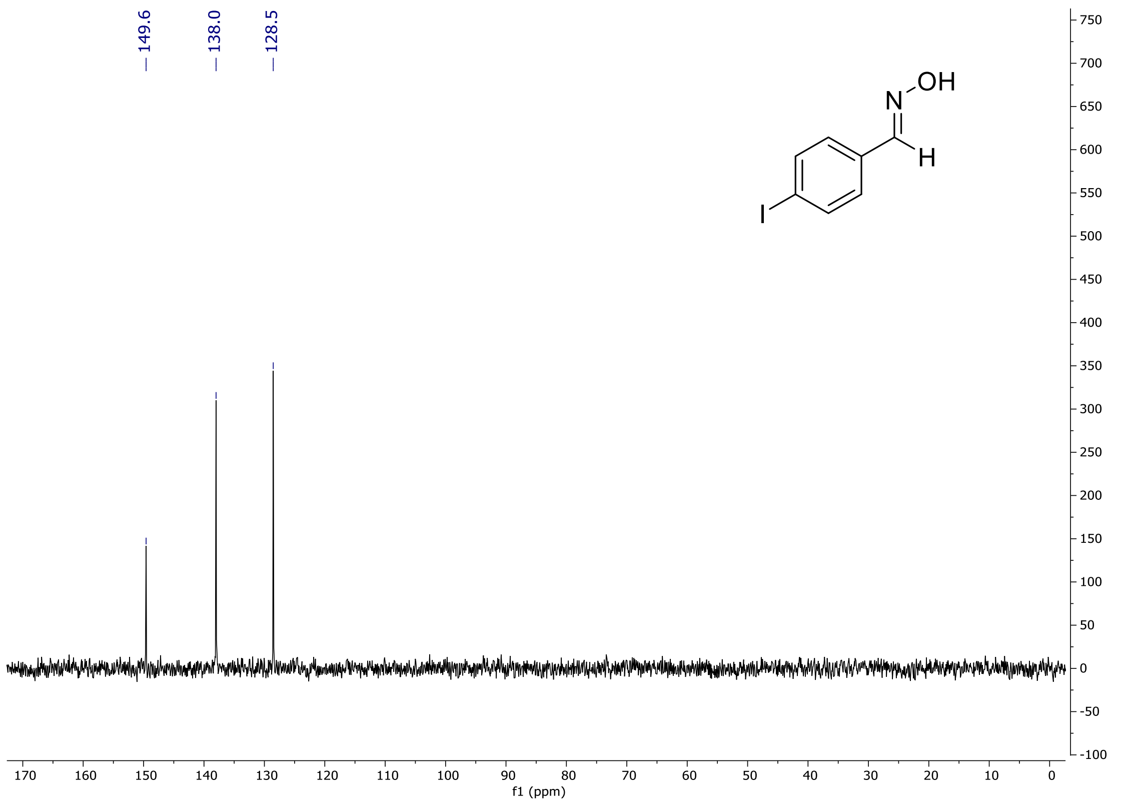

Figure SI_6: DEPT 135-NMR for **1e** in $CDCl_3$ (75 MHz).

Acquisition Parameter

Source Type	ESI	Ion Polarity	Positive	Set Nebulizer	2.0 Bar
Focus	Active	Set Capillary	3500 V	Set Dry Heater	250 °C
Scan Begin	50 m/z	Set End Plate Offset	-500 V	Set Dry Gas	5.0 l/min
Scan End	1500 m/z	Set Charging Voltage	2000 V	Set Divert Valve	Source
		Set Corona	0 nA	Set APCI Heater	0 °C

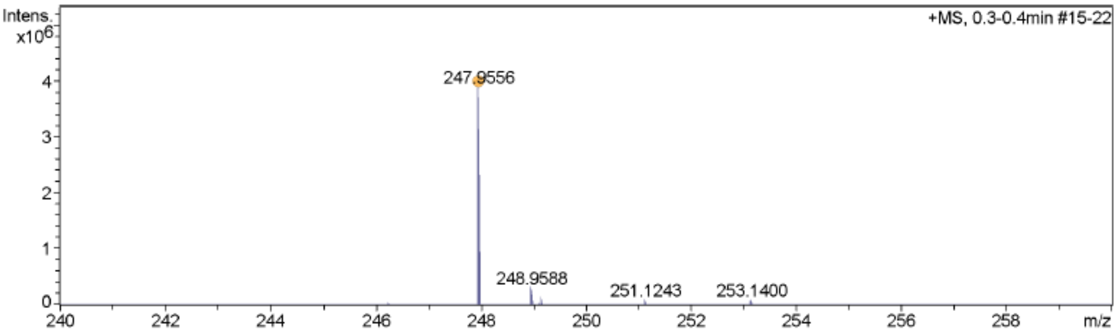

Meas. m/z	#	Ion Formula	m/z	err [ppm]	mSigma	# mSigma	Score	rdb	e⁻ Conf	N-Rule	Adduct
247.9556	1	C7H7INO	247.9567	4.6	4.4	2	67.67	5.0	even	ok	M+H

Figure SI_7: HRMS (ESI⁺, m/z) analysis of **1e**.

4-(Isopropylcarbamoyl)benzaldoxime (1i)

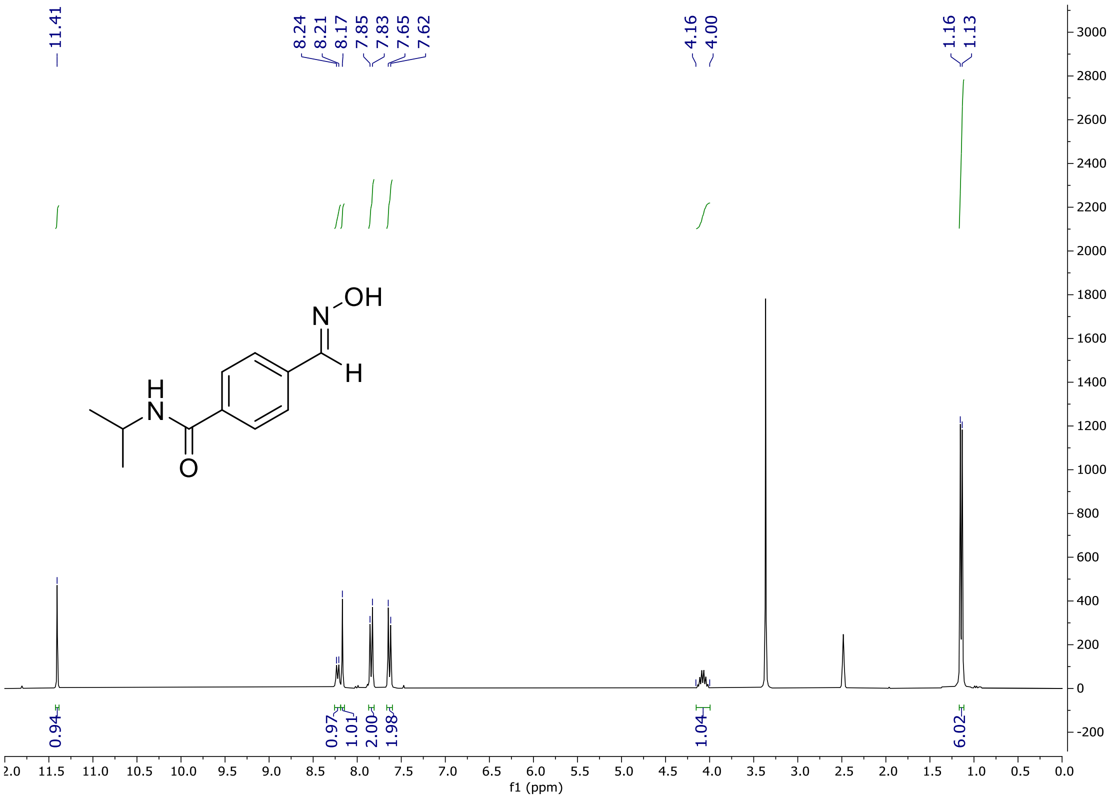

Figure SI_8: ^{1}H-NMR for **1i** in DMSO-d^6 (300 MHz).

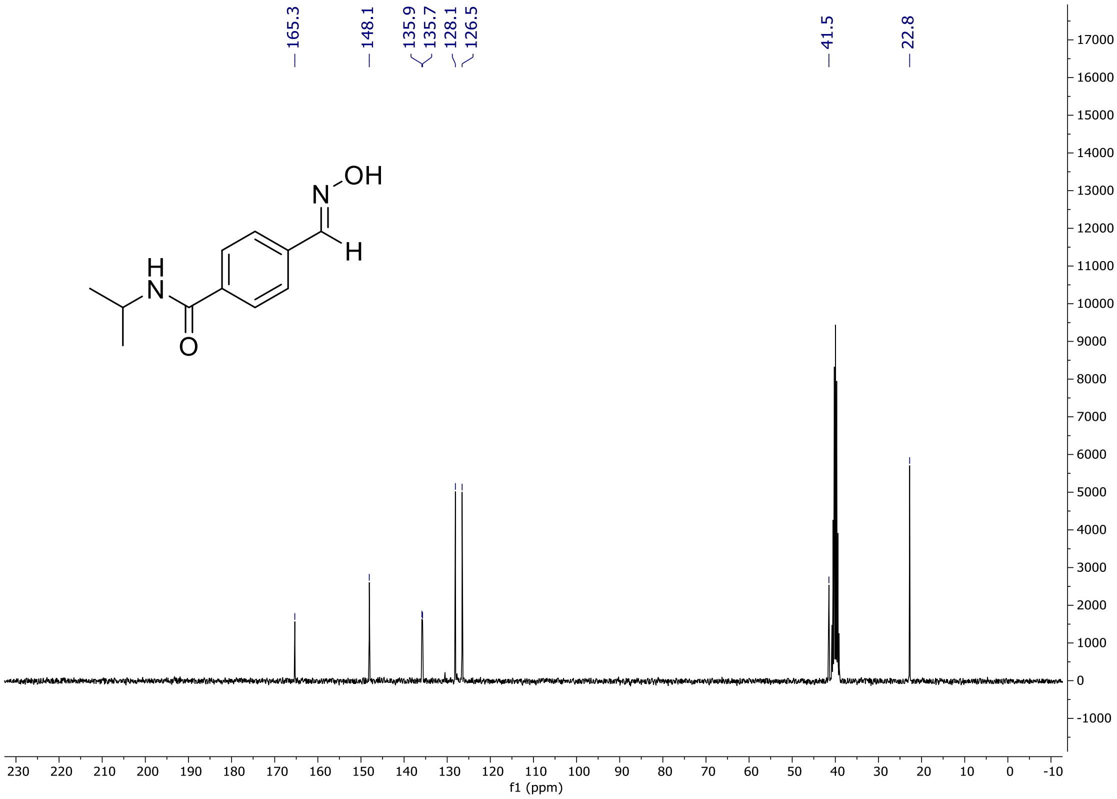

Figure SI_9: ^{13}C-NMR for **1i** in DMSO-d^6 (75 MHz).

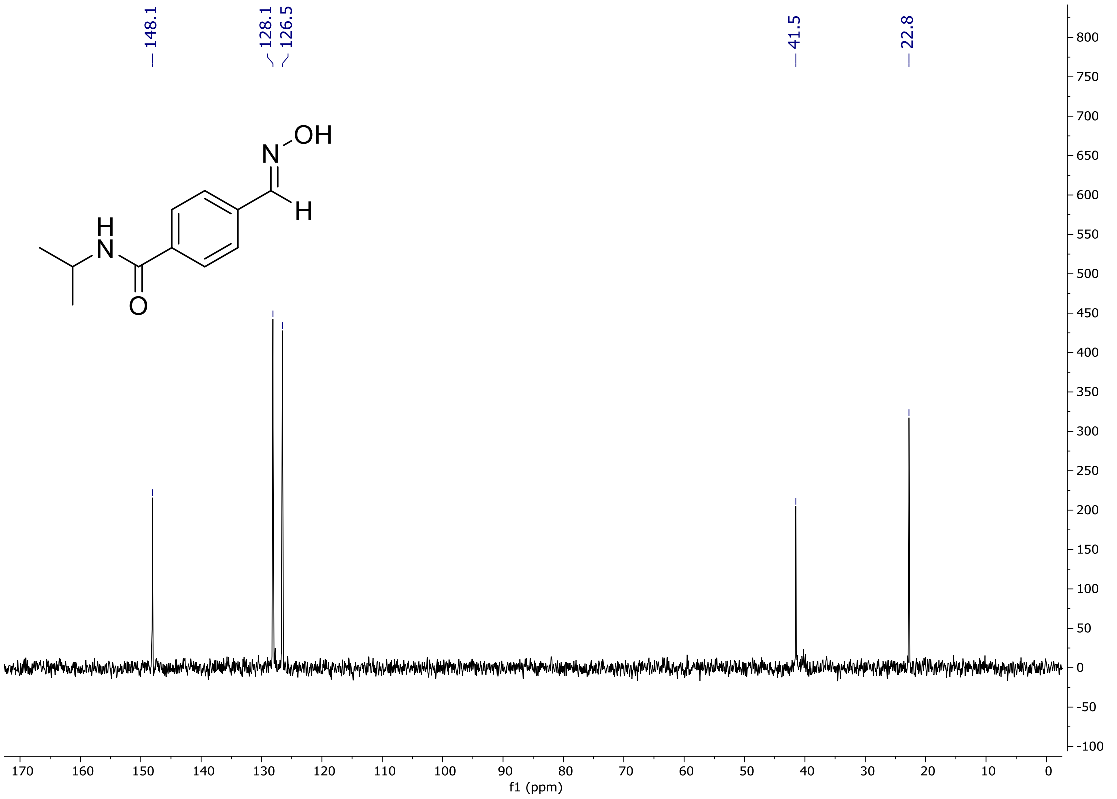

Figure SI_10: DEPT 135-NMR for **1i** in DMSO-d^6 (75 MHz).

Acquisition Parameter

Source Type	ESI	Ion Polarity	Positive	Set Nebulizer	2.0 Bar
Focus	Active	Set Capillary	4000 V	Set Dry Heater	250 °C
Scan Begin	50 m/z	Set End Plate Offset	-500 V	Set Dry Gas	5.0 l/min
Scan End	1500 m/z	Set Charging Voltage	2000 V	Set Divert Valve	Source
		Set Corona	0 nA	Set APCI Heater	0 °C

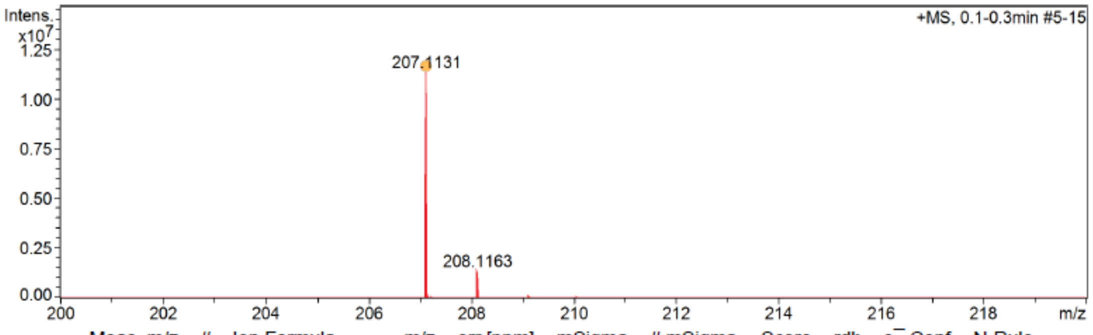

Meas. m/z	#	Ion Formula	m/z	err [ppm]	mSigma	# mSigma	Score	rdb	e⁻ Conf	N-Rule
207.1131	1	C11H15N2O2	207.1128	-1.6	5.2	2	100.00	6.0	even	ok

Figure SI_11: HRMS (ESI^+, m/z) analysis of **1i**.

4-(Pyrrolidine-1-carbonyl)benzaldoxime (1j)

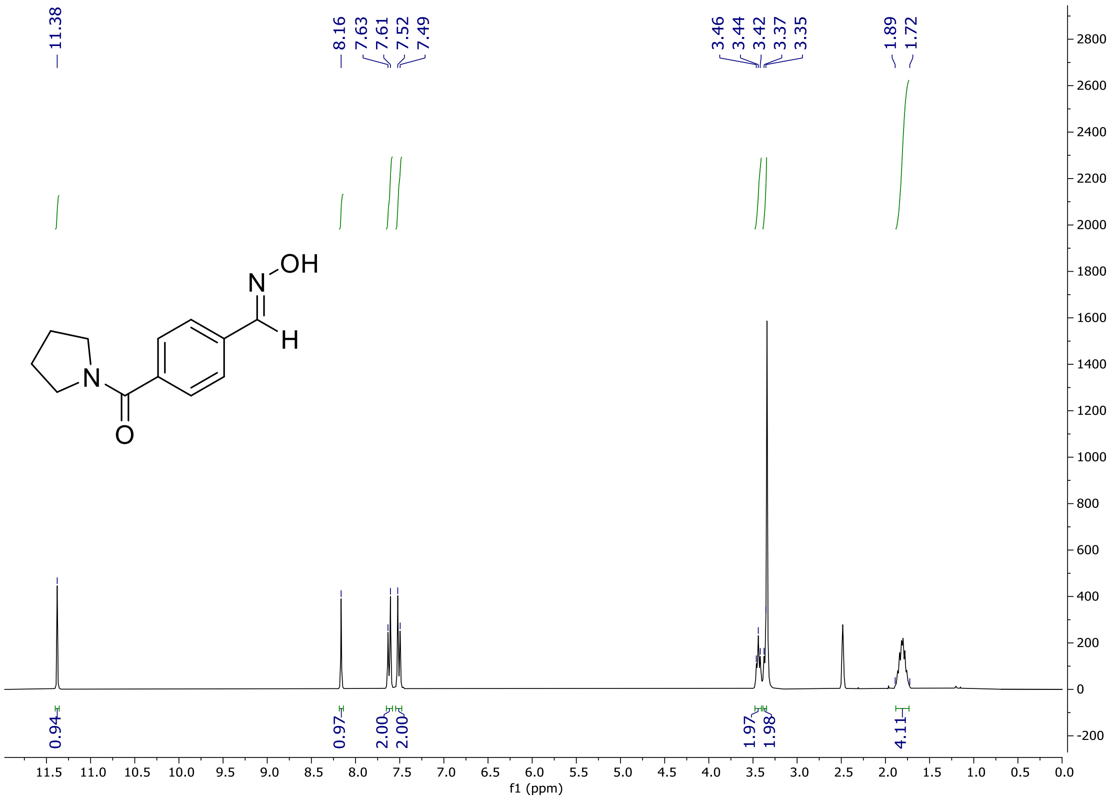

Figure SI_12: ^{1}H-NMR for **1j** in DMSO-d^6 (300 MHz).

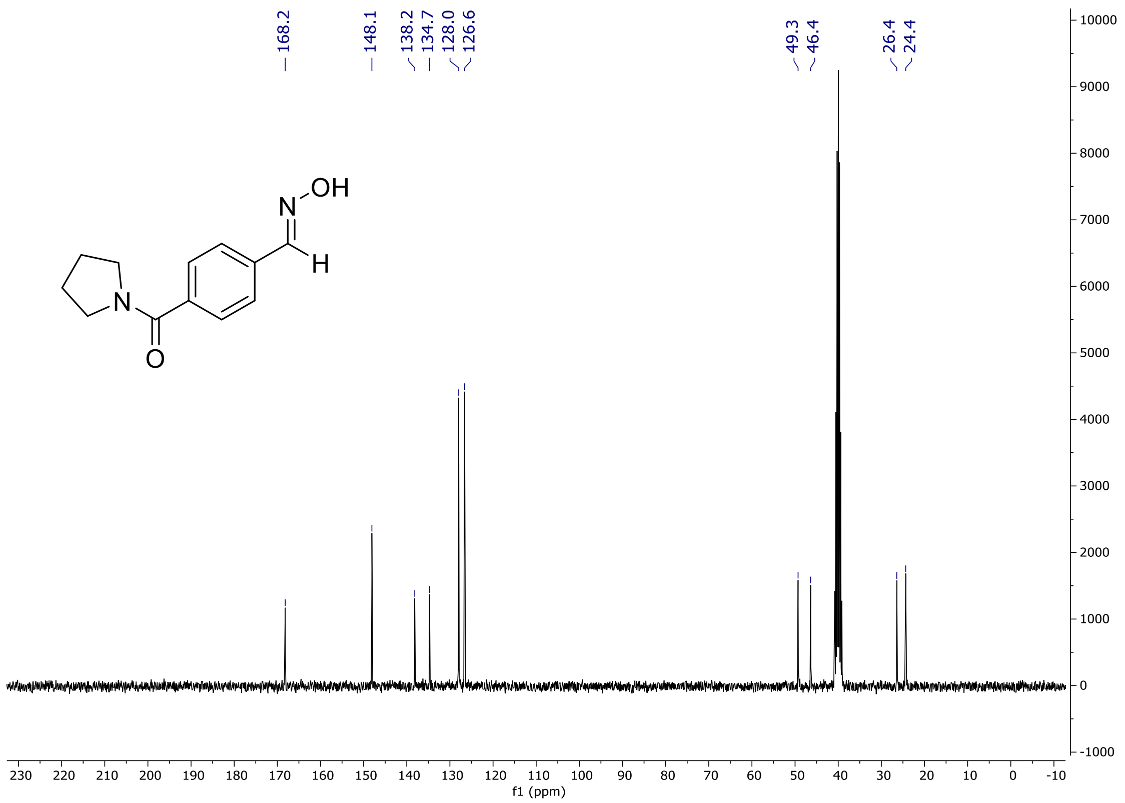

Figure SI_13: ^{13}C-NMR for **1j** in DMSO-d^6 (75 MHz).

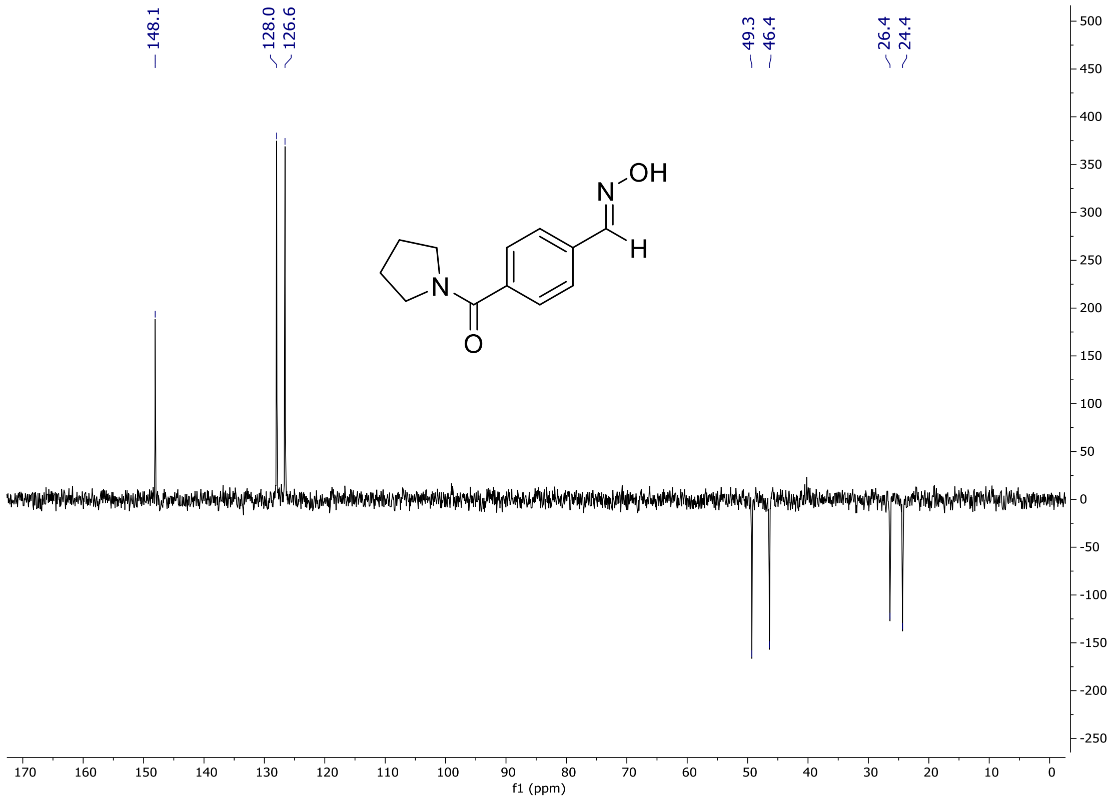

Figure SI_14: DEPT 135-NMR for **1j** in DMSO-d^6 (75 MHz).

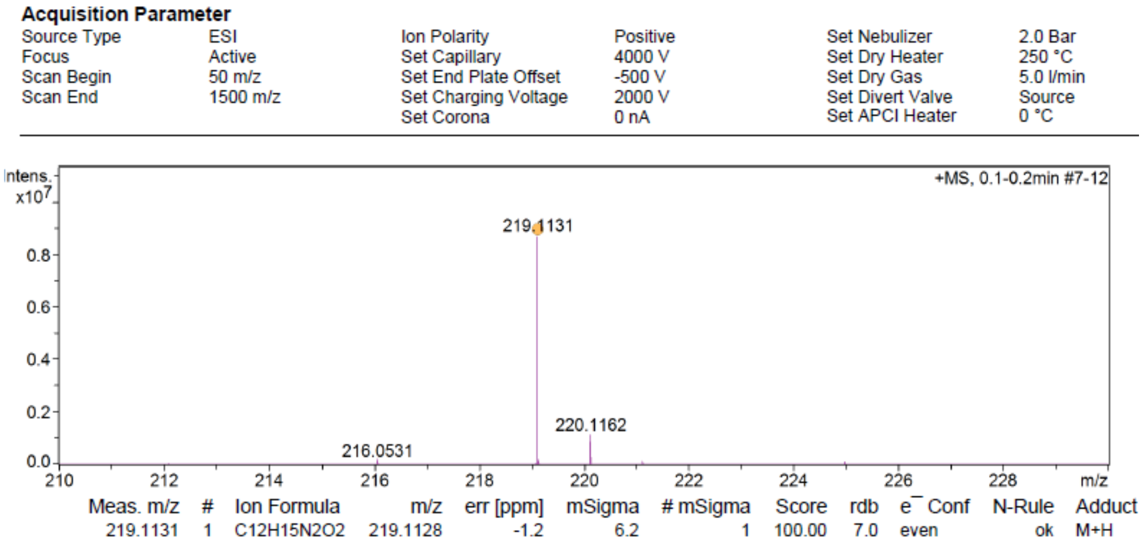

Figure SI_15: HRMS (ESI^+, m/z) analysis of **1j**.

4-Acetylbenzothioamide (1k)

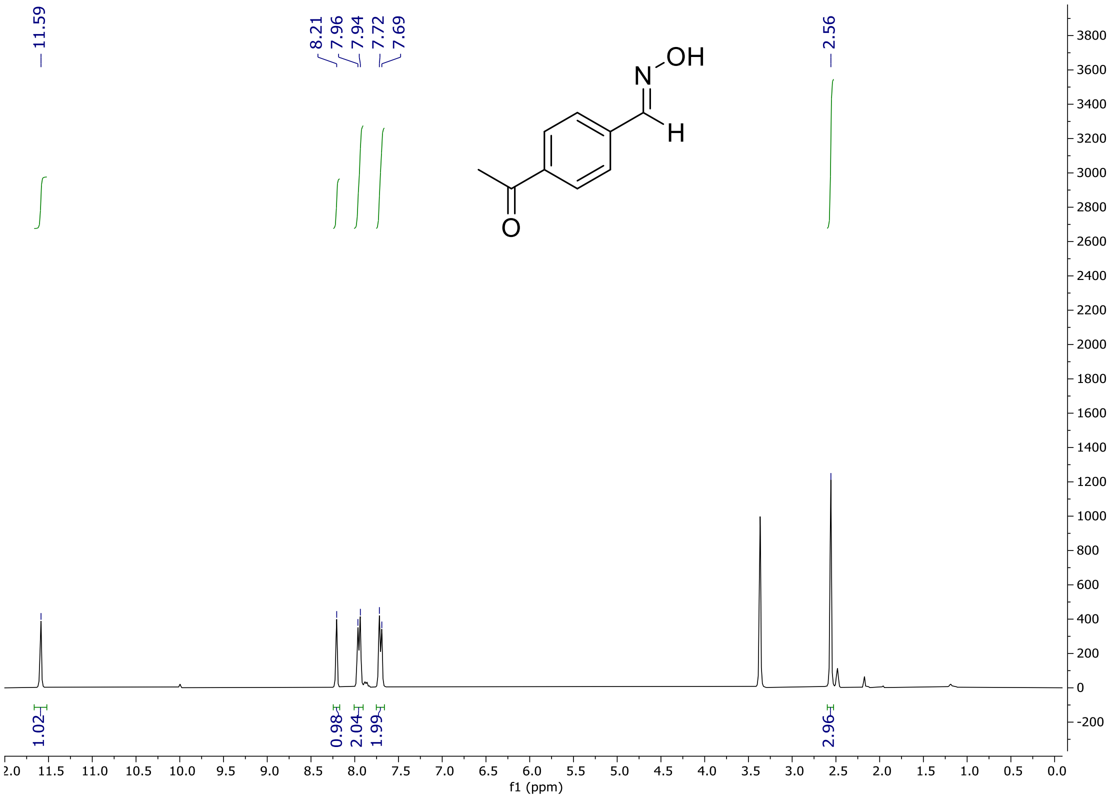

Figure SI_16: ^{1}H-NMR for **1k** in DMSO-d^6 (300 MHz).

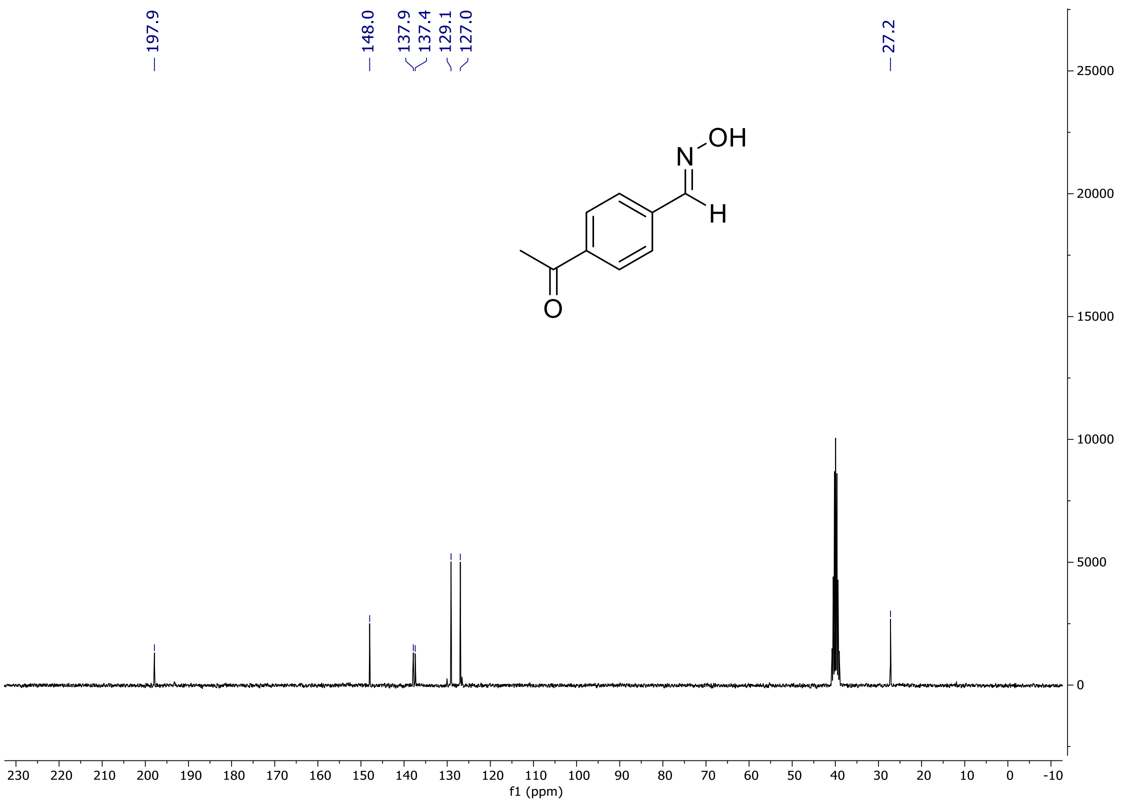

Figure SI_17: ^{13}C-NMR for **1k** in DMSO-d^6 (75 MHz).

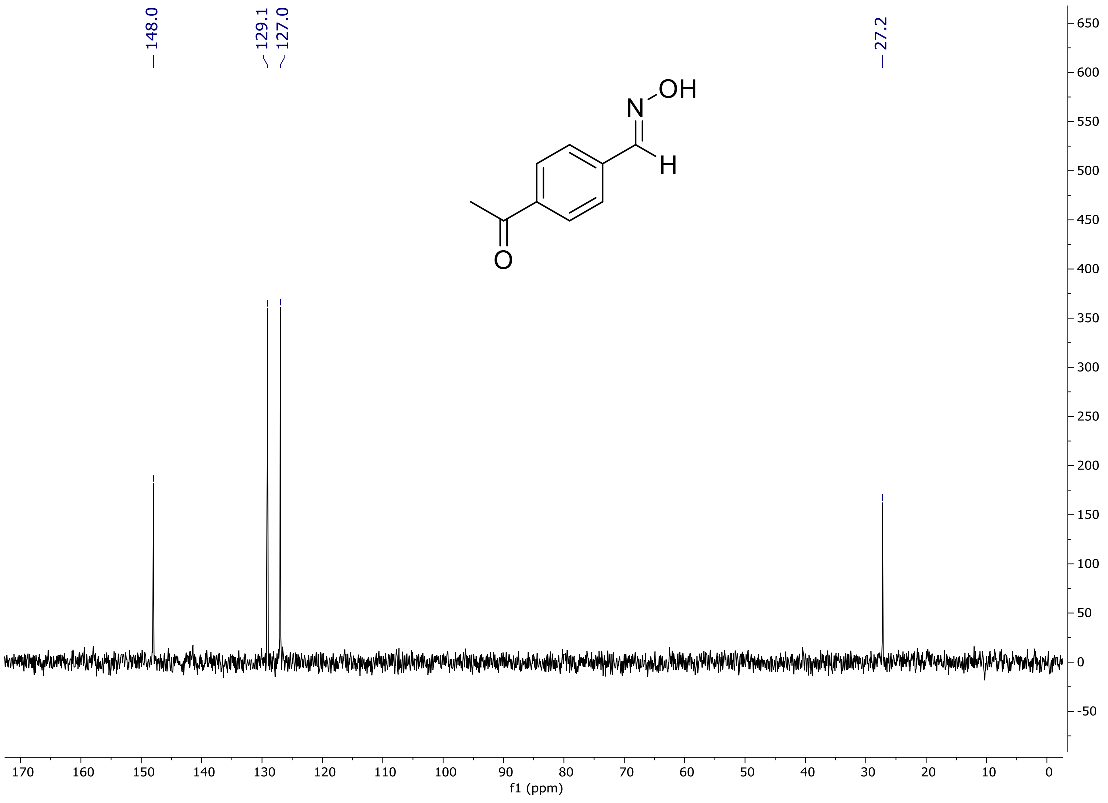

Figure SI_18: DEPT 135-NMR for **1k** in DMSO-d^6 (75 MHz).

Acquisition Parameter

Source Type	ESI	Ion Polarity	Positive	Set Nebulizer	2.0 Bar
Focus	Active	Set Capillary	3500 V	Set Dry Heater	250 °C
Scan Begin	50 m/z	Set End Plate Offset	-500 V	Set Dry Gas	5.0 l/min
Scan End	1500 m/z	Set Charging Voltage	2000 V	Set Divert Valve	Source
		Set Corona	0 nA	Set APCI Heater	0 °C

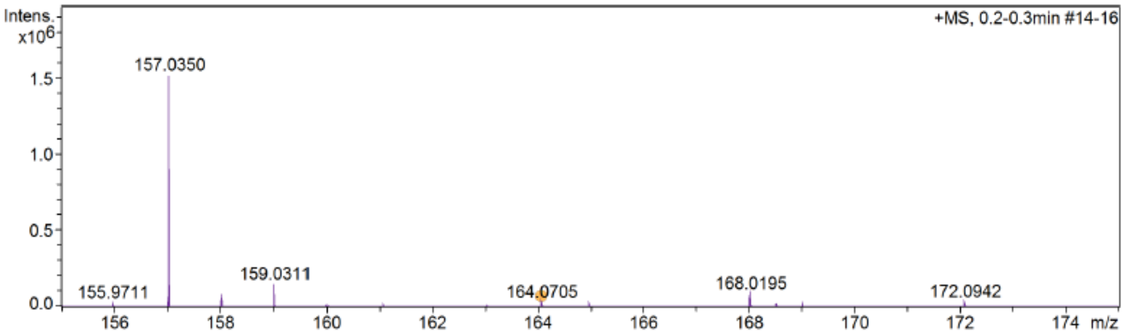

Meas. m/z	#	Ion Formula	m/z	err [ppm]	mSigma	# mSigma	Score	rdb	e¯ Conf	N-Rule
164.0705	1	C9H10NO2	164.0706	0.8	114.1	1	100.00	6.0	even	ok

Figure SI_19: HRMS (ESI$^+$, m/z) analysis of **1k**.

Benzothioamide (3a)

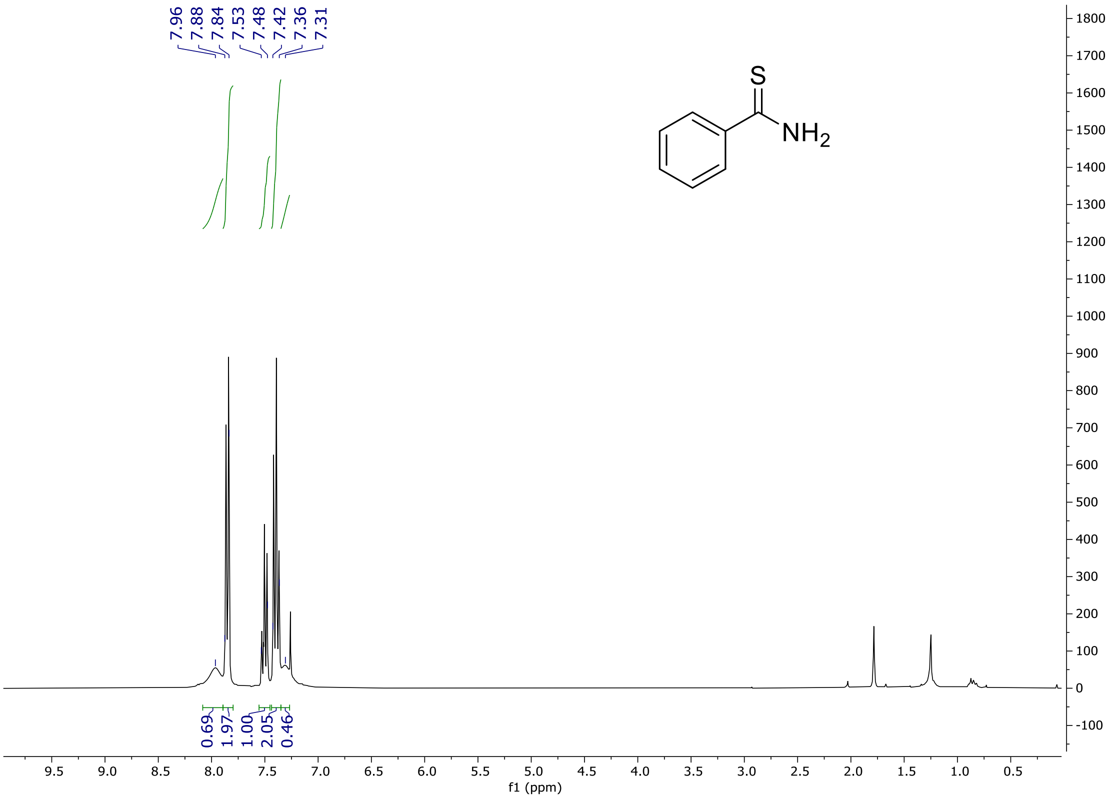

Figure SI_20: ^{1}H-NMR for **3a** in $CDCl_3$ (300 MHz).

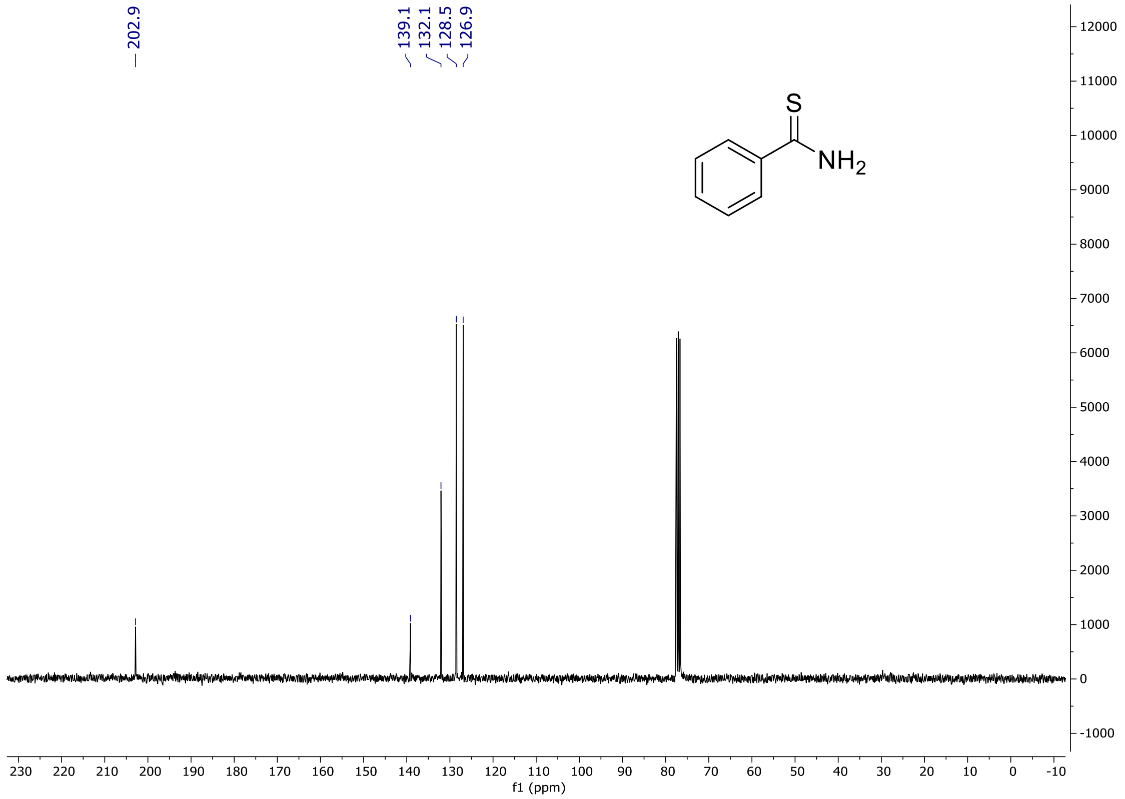

Figure SI_21: ^{13}C-NMR for **3a** in $CDCl_3$ (75 MHz).

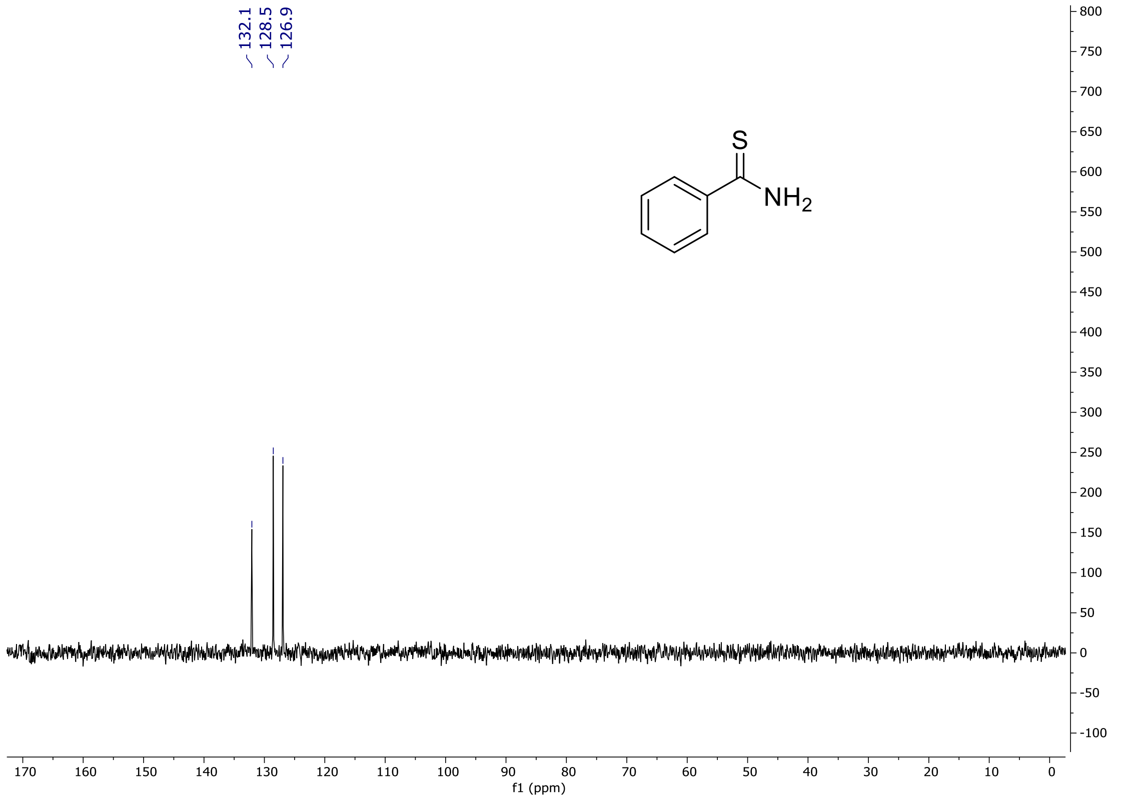

Figure SI_22: DEPT 135-NMR for **3a** in $CDCl_3$ (75 MHz).

4-Chlorobenzothioamide (3b)

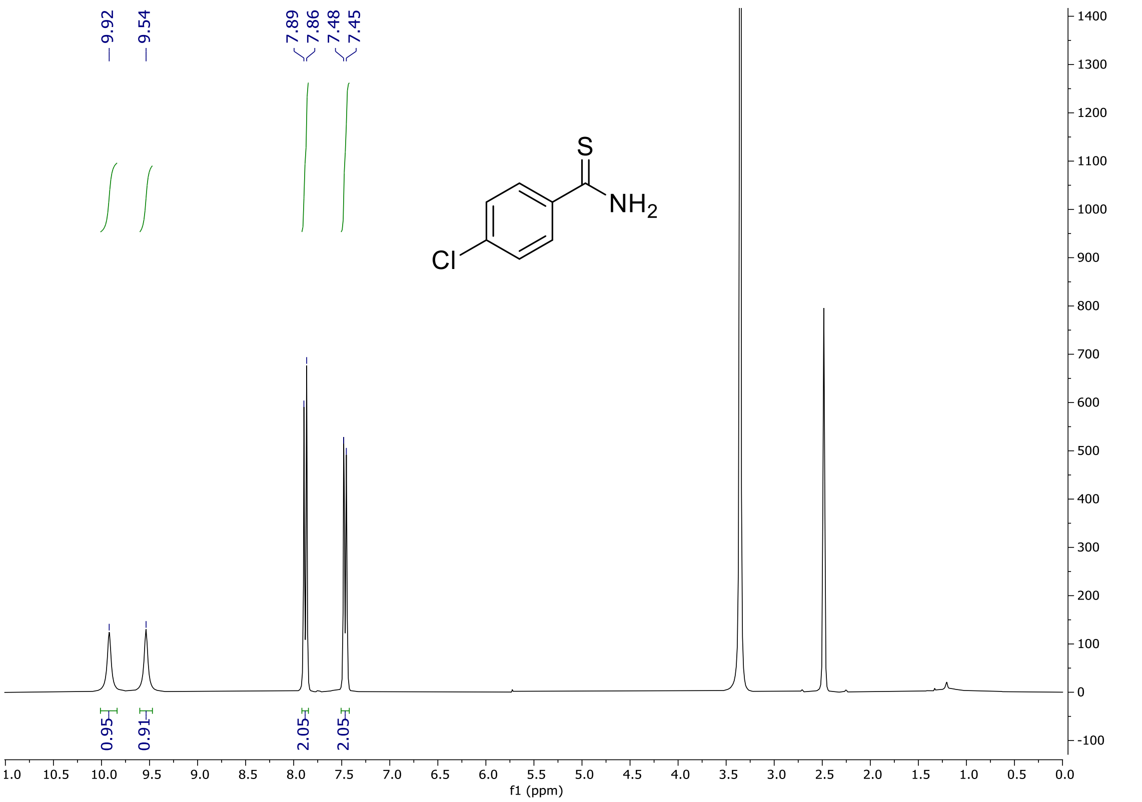

Figure SI_23: ^{1}H-NMR for **3b** in DMSO-d^{6} (300 MHz).

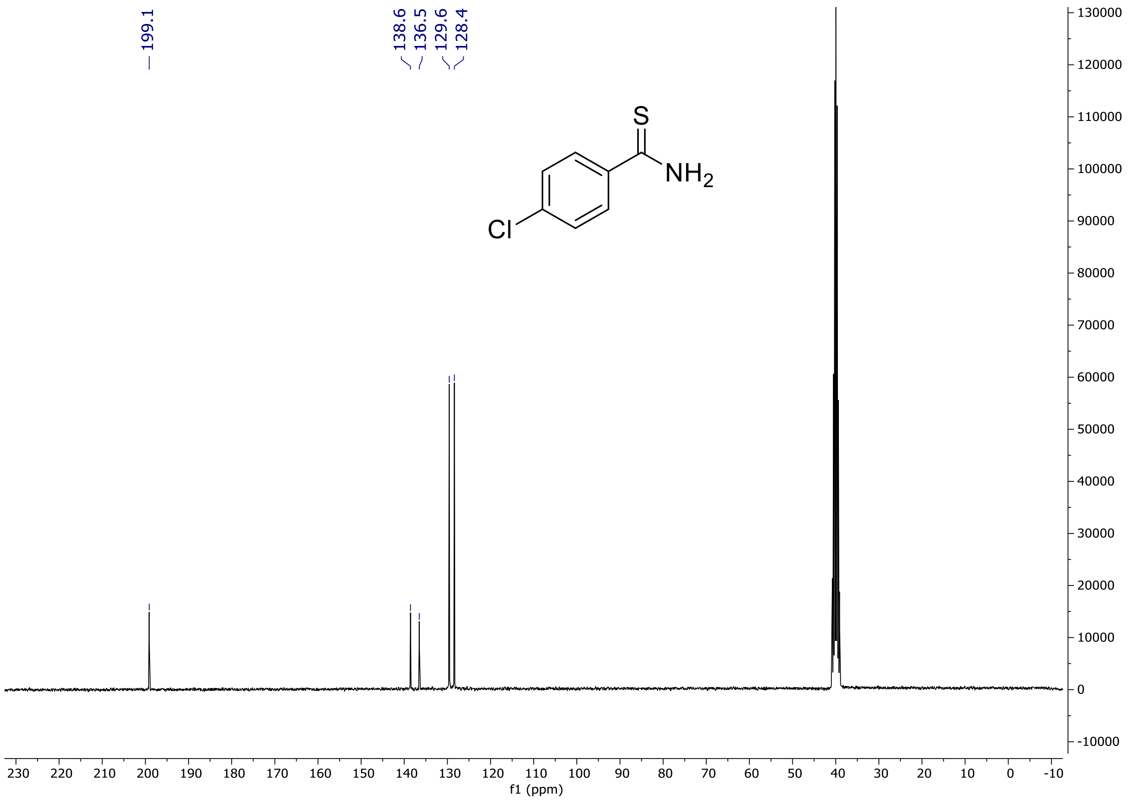

Figure SI_24: ^{13}C-NMR for **3b** in DMSO-d^{6} (75 MHz).

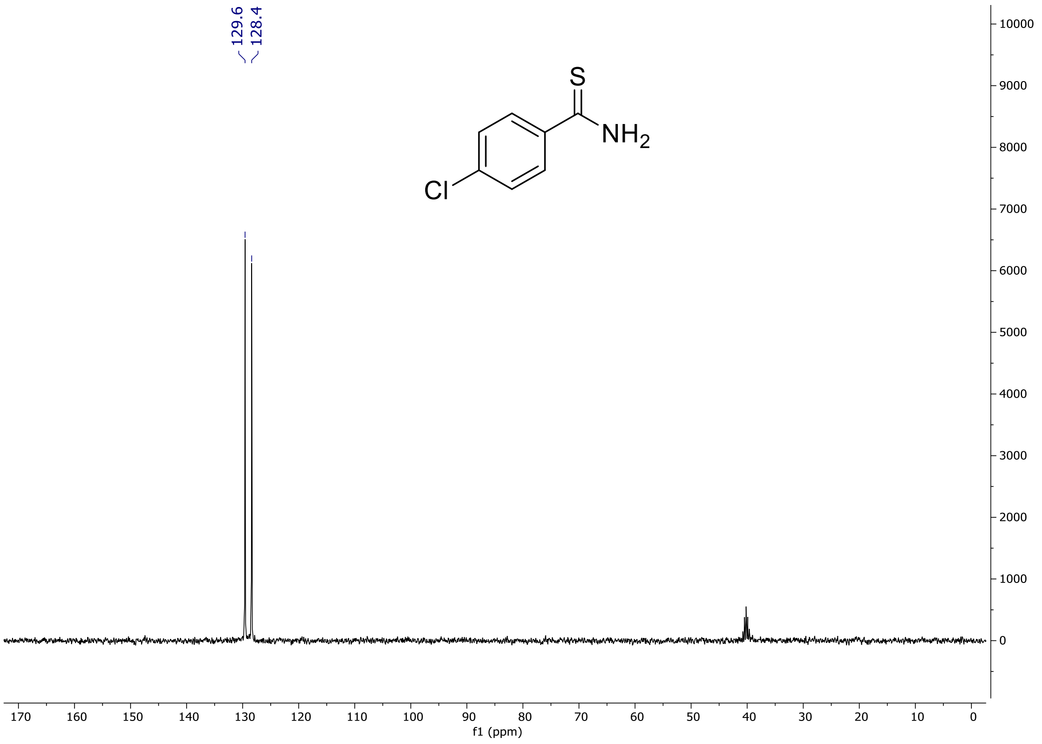

Figure SI_25: DEPT 135-NMR for **3b** in DMSO-d^6 (75 MHz).

3-Chlorobenzothioamide (3c)

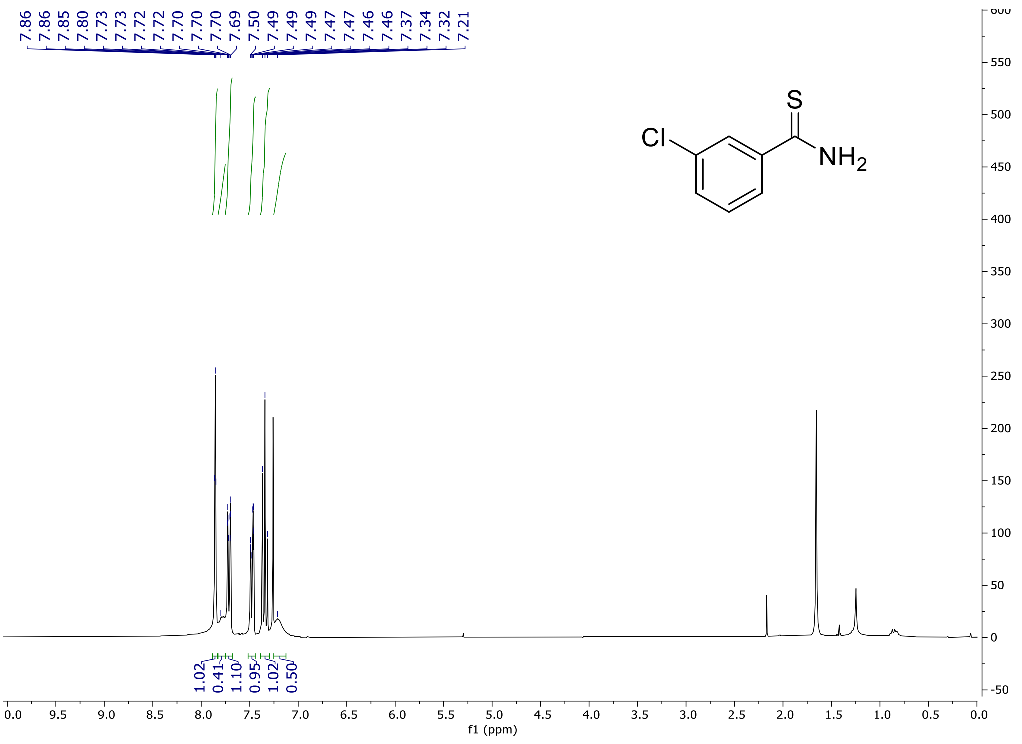

Figure SI_26: ^{1}H-NMR for **3c** in $CDCl_3$ (300 MHz).

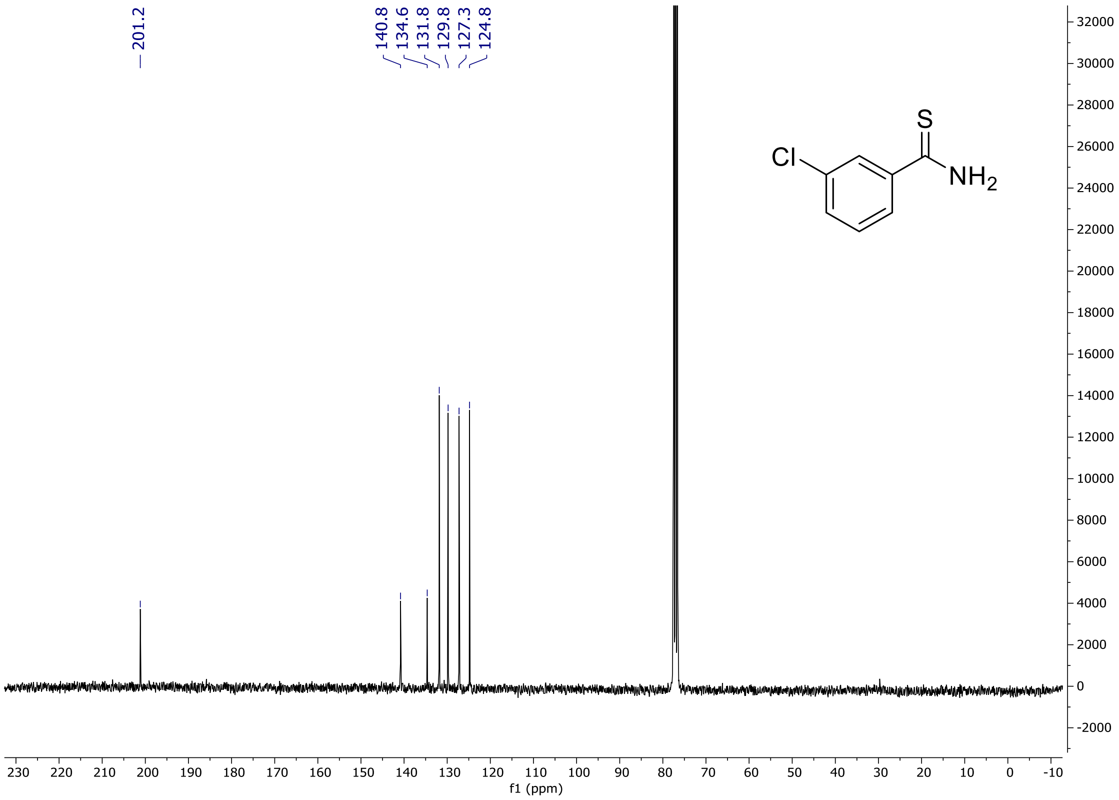

Figure SI_27: ^{13}C-NMR for **3c** in $CDCl_3$ (75 MHz).

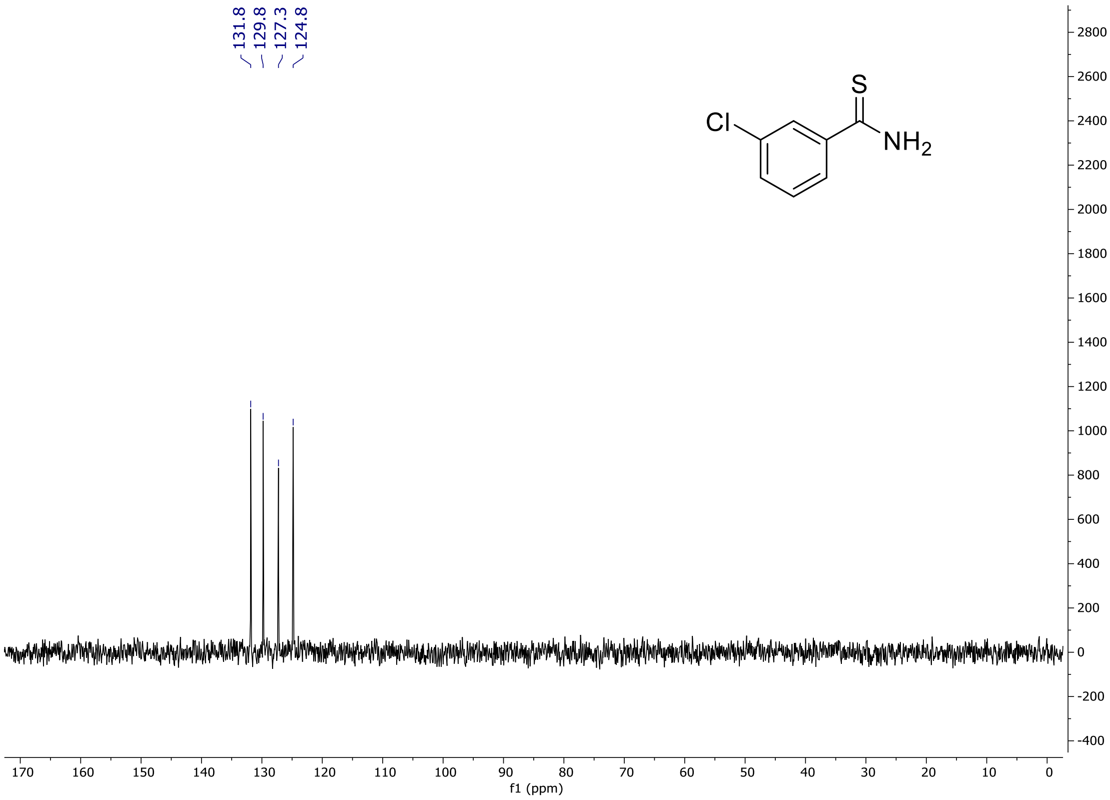

Figure SI_28: DEPT 135-NMR for **3c** in $CDCl_3$ (75 MHz).

2-Chlorobenzothioamide (3d)

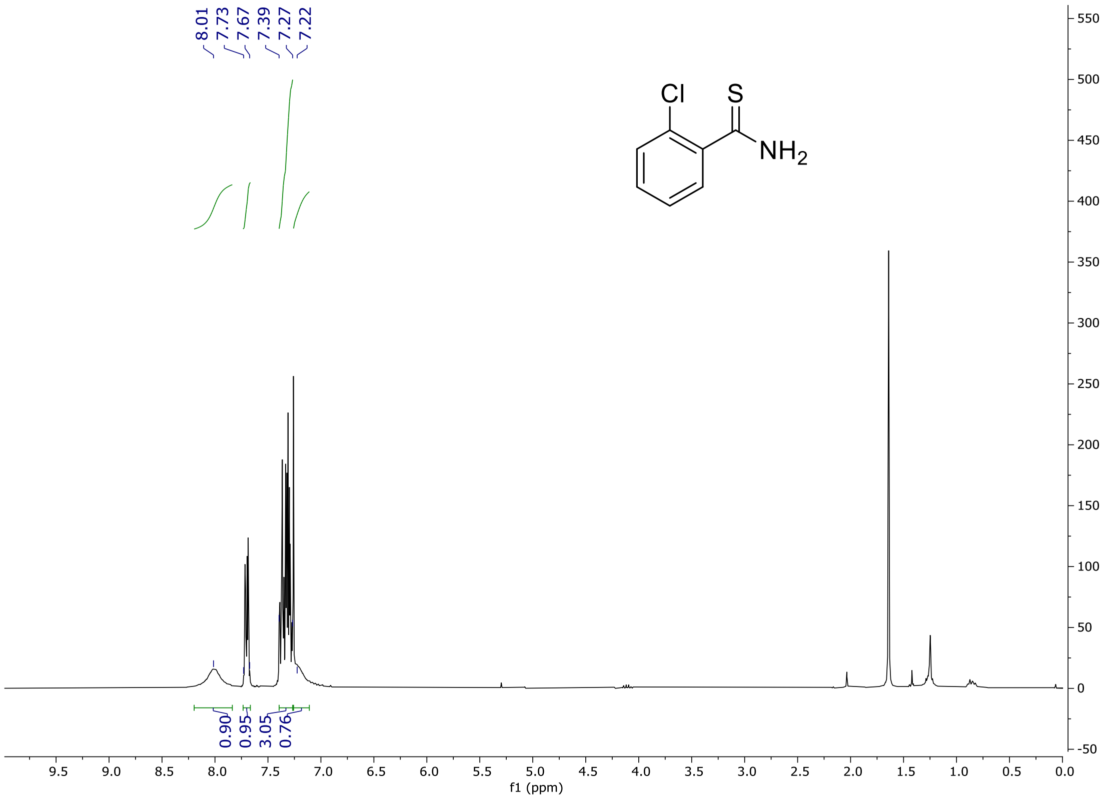

Figure SI_29: ^{1}H-NMR for **3d** in $CDCl_3$ (300 MHz).

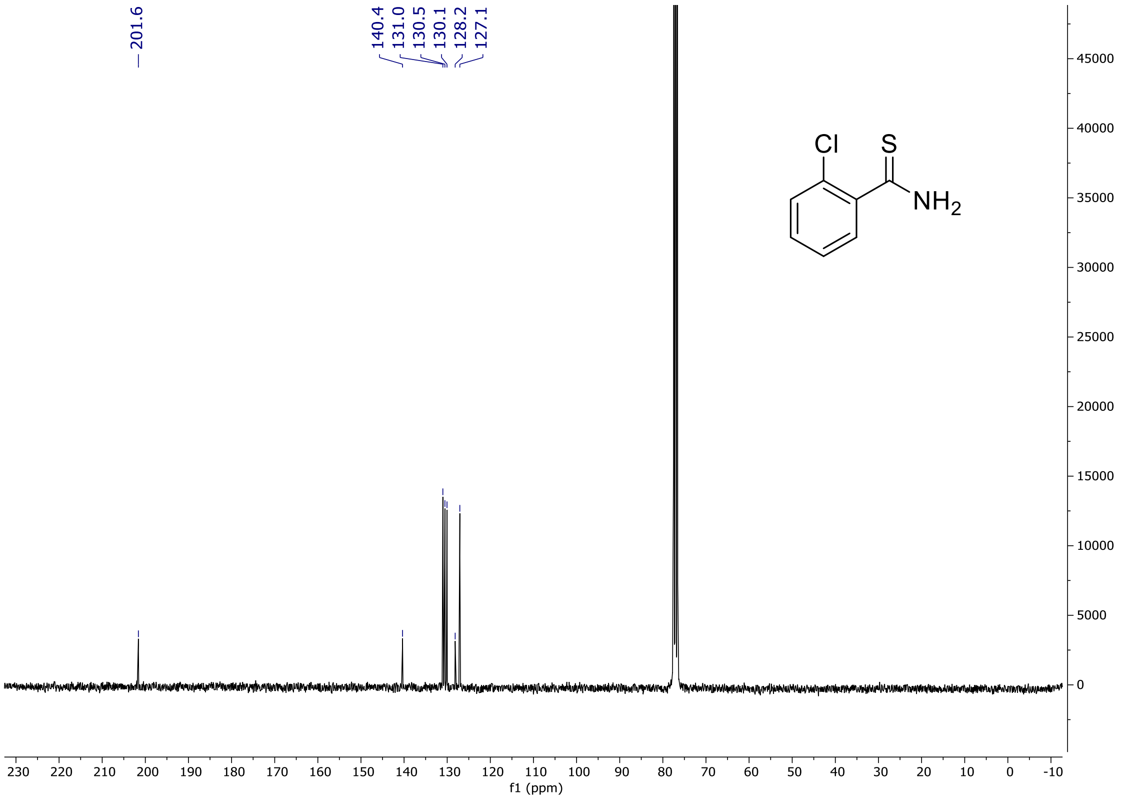

Figure SI_30: ^{13}C-NMR for **3d** in $CDCl_3$ (75 MHz).

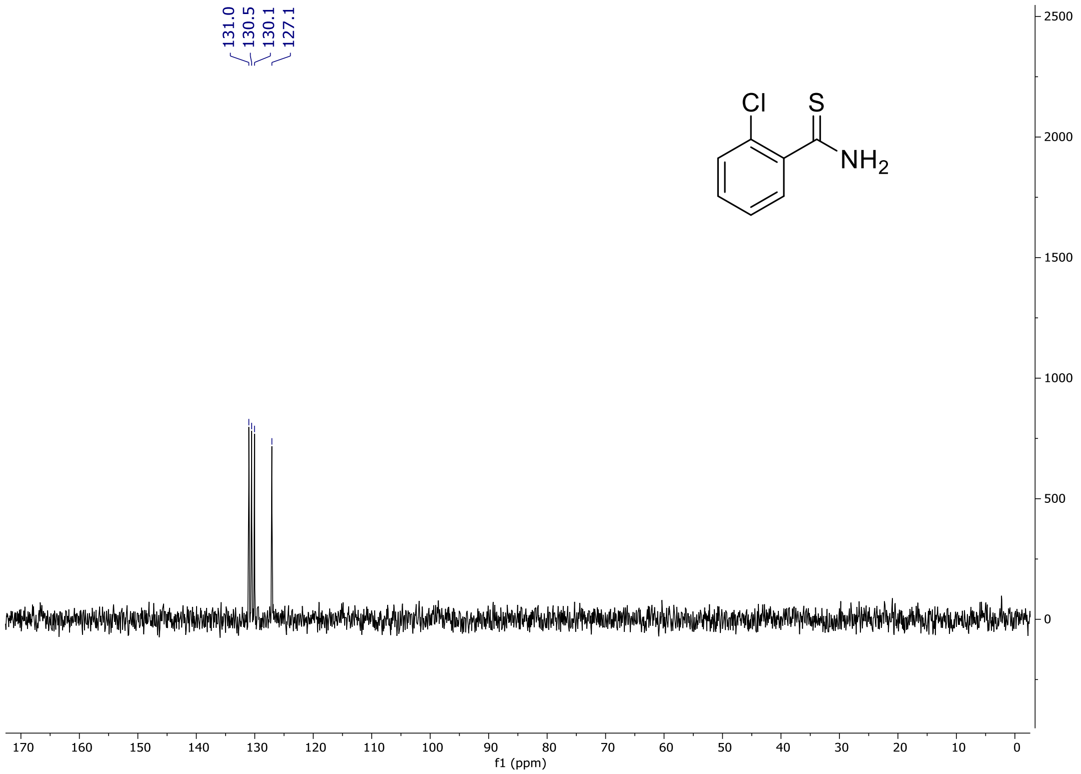

Figure SI_31: DEPT 135-NMR for **3d** in $CDCl_3$ (75 MHz).

4-Iodobenzothioamide (3e)

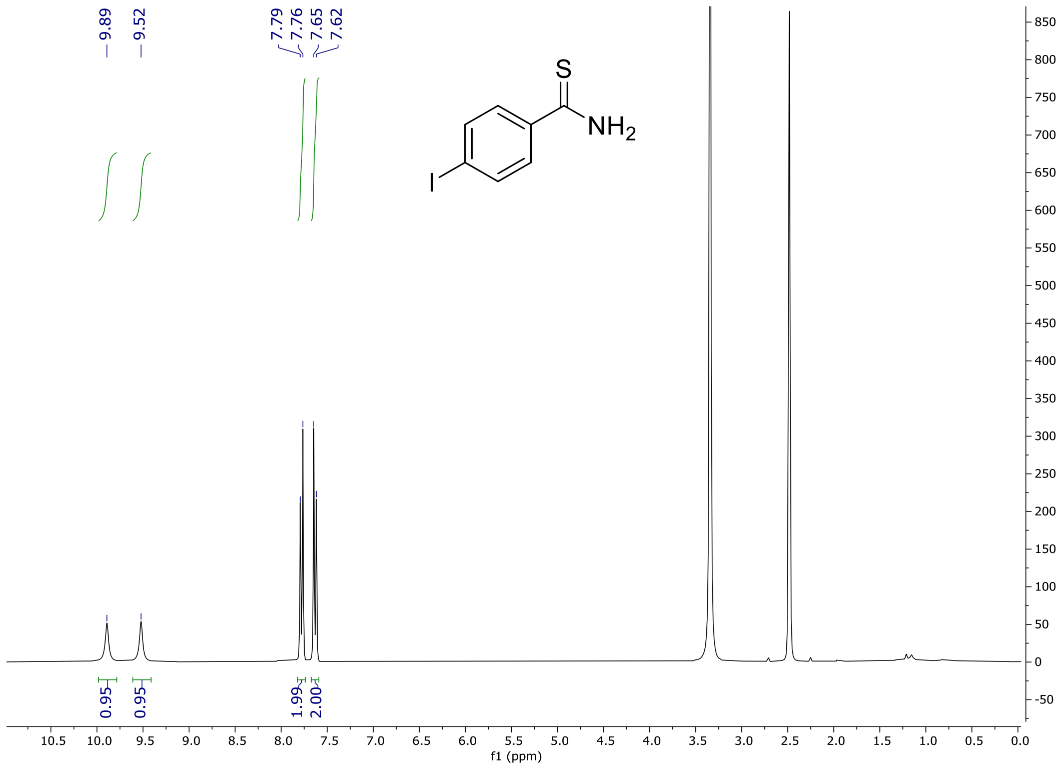

Figure SI_32: ^{1}H-NMR for **3e** in DMSO-d^6 (300 MHz).

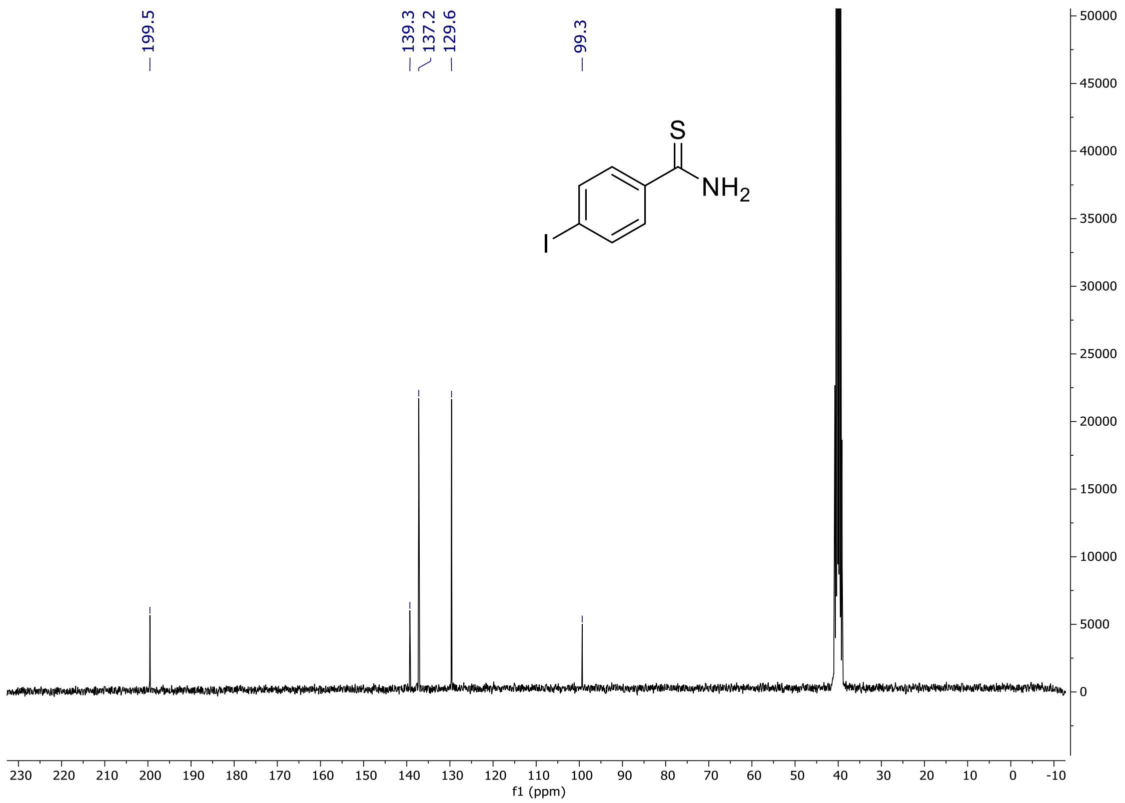

Figure SI_33: ^{13}C-NMR for **3e** in DMSO-d^6 (75 MHz).

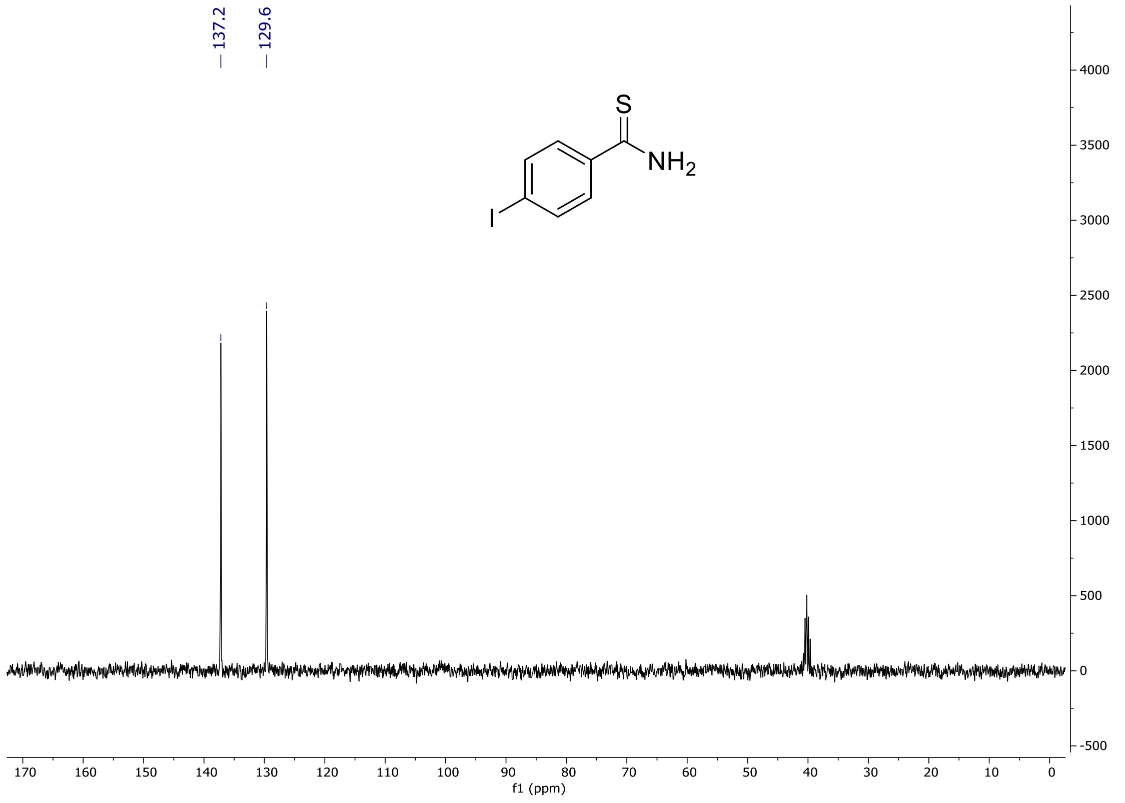

Figure SI_34: DEPT 135-NMR for **3e** in DMSO-d^6 (75 MHz).

4-Methylbenzothioamide (3f)

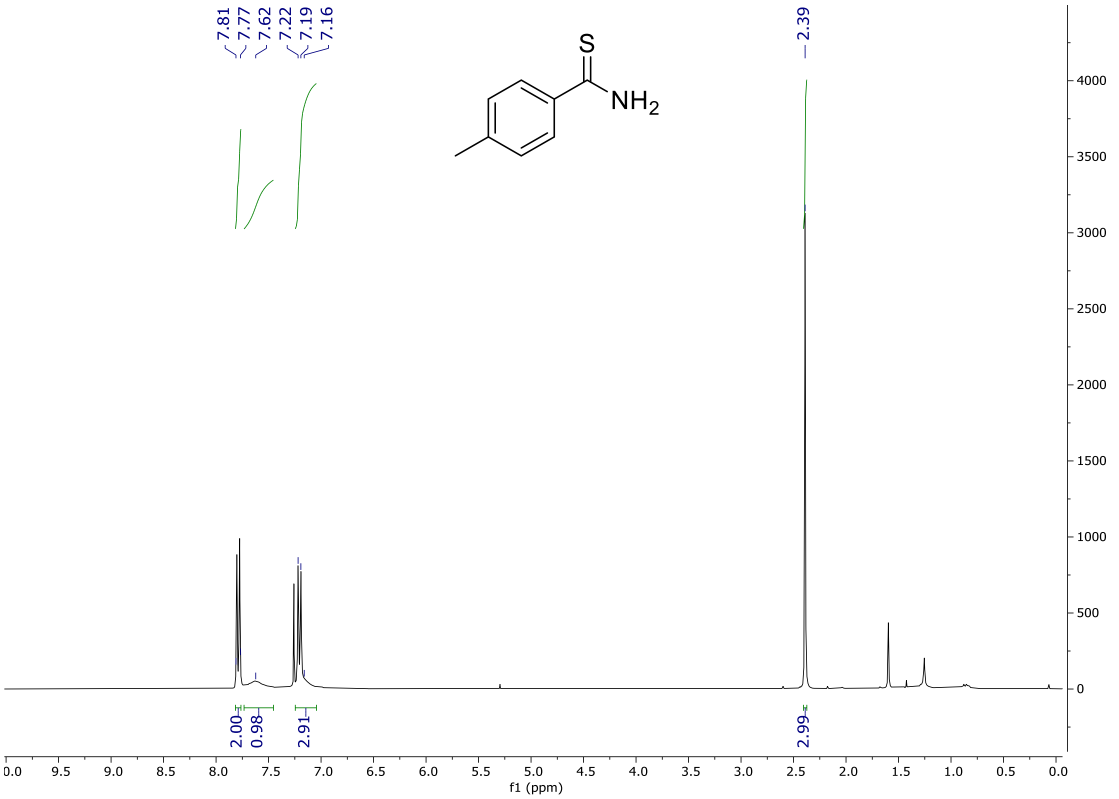

Figure SI_35: ^{1}H-NMR for **3f** in $CDCl_3$ (300 MHz).

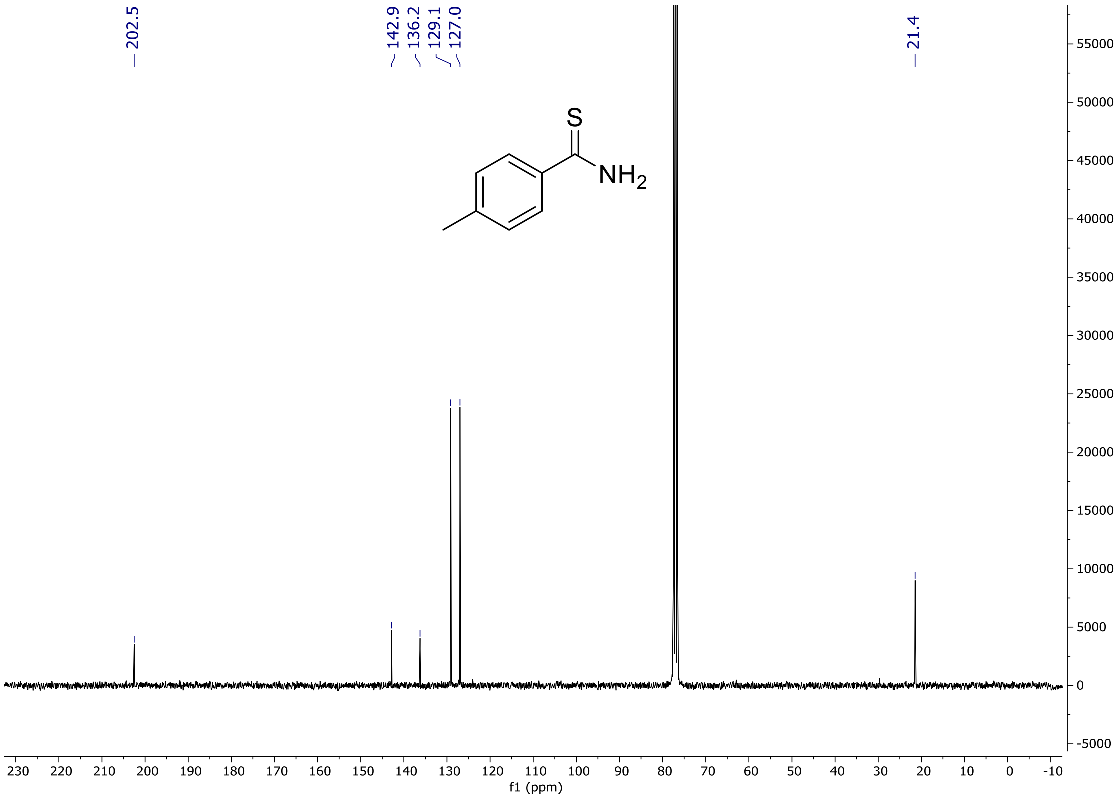

Figure SI_36: ^{13}C-NMR for **3f** in $CDCl_3$ (75 MHz).

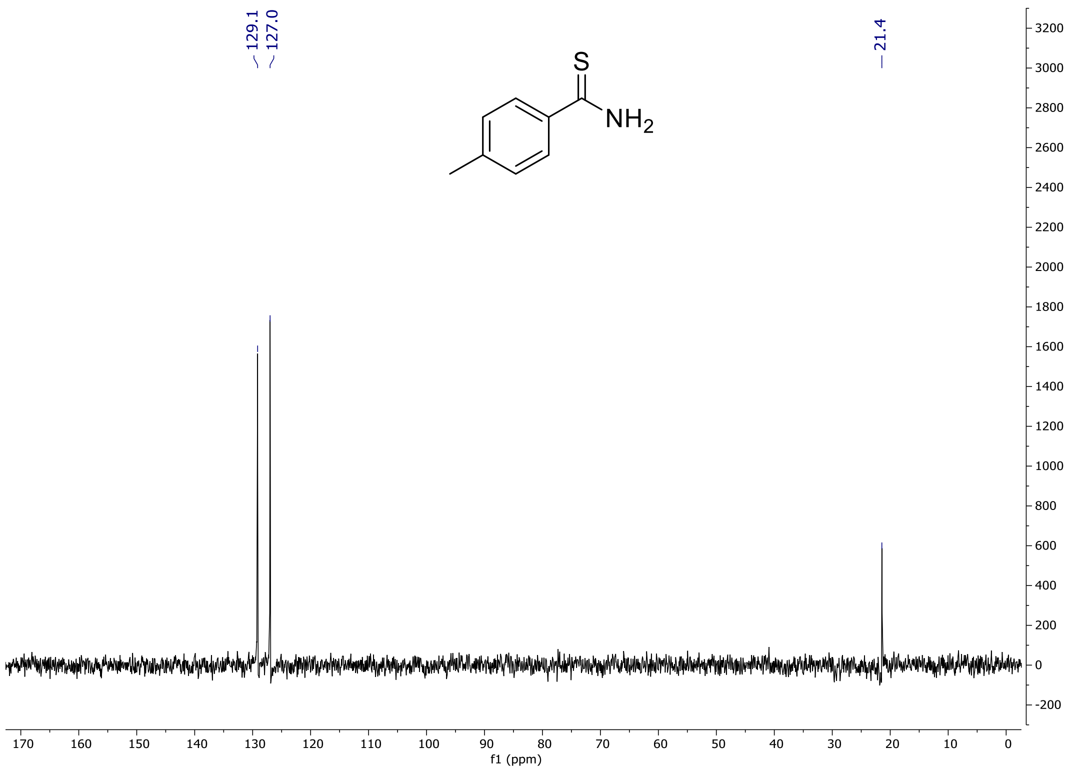

Figure SI_37: DEPT 135-NMR for **3f** in $CDCl_3$ (75 MHz).

4-Methoxybenzothioamide (3g)

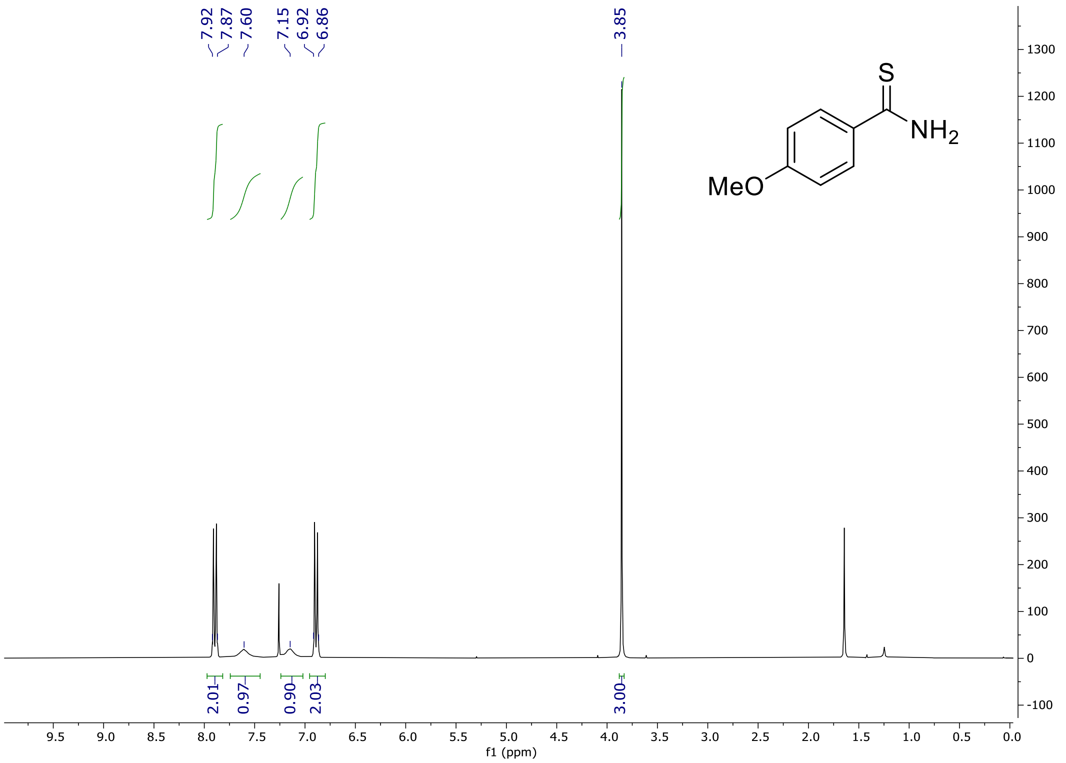

Figure SI_38: ^{1}H-NMR for **3g** in $CDCl_3$ (300 MHz).

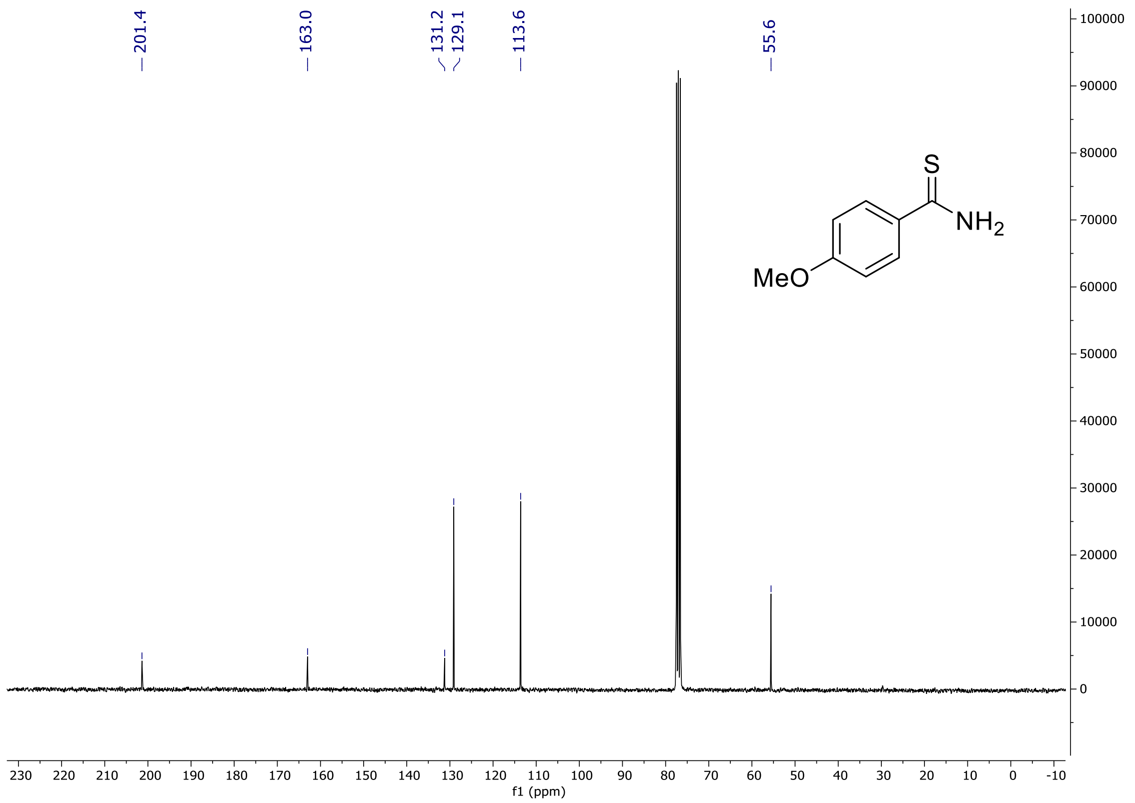

Figure SI_39: ^{13}C-NMR for **3g** in $CDCl_3$ (75 MHz).

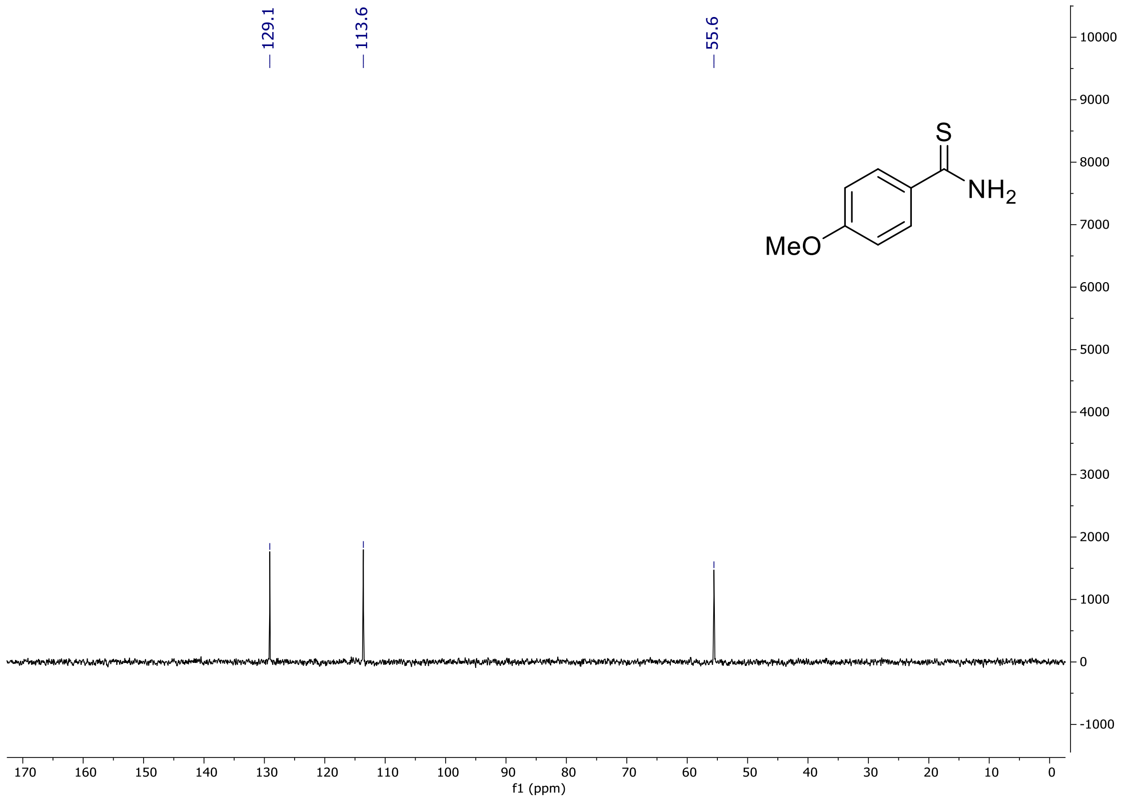

Figure SI_40: DEPT 135-NMR for **3g** in $CDCl_3$ (75 MHz).

4-Acetamidobenzothioamide (3h)

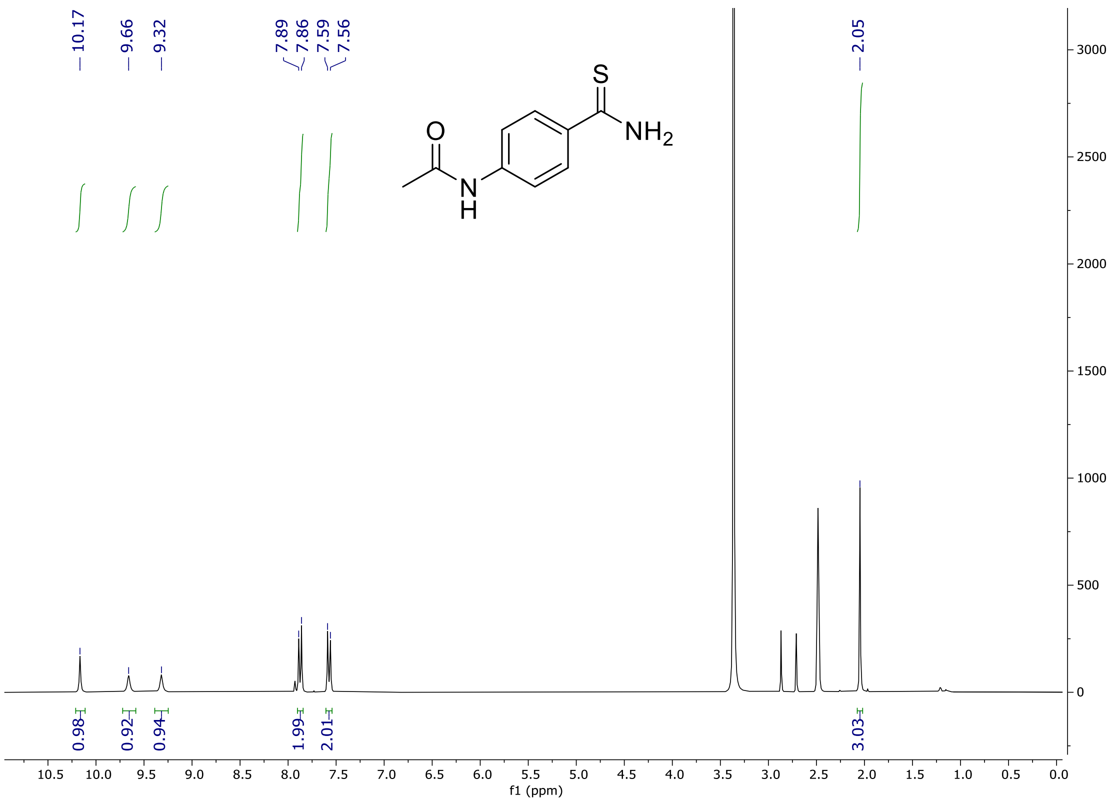

Figure SI_41: ^{1}H-NMR for **3h** in DMSO-d^{6} (300 MHz).

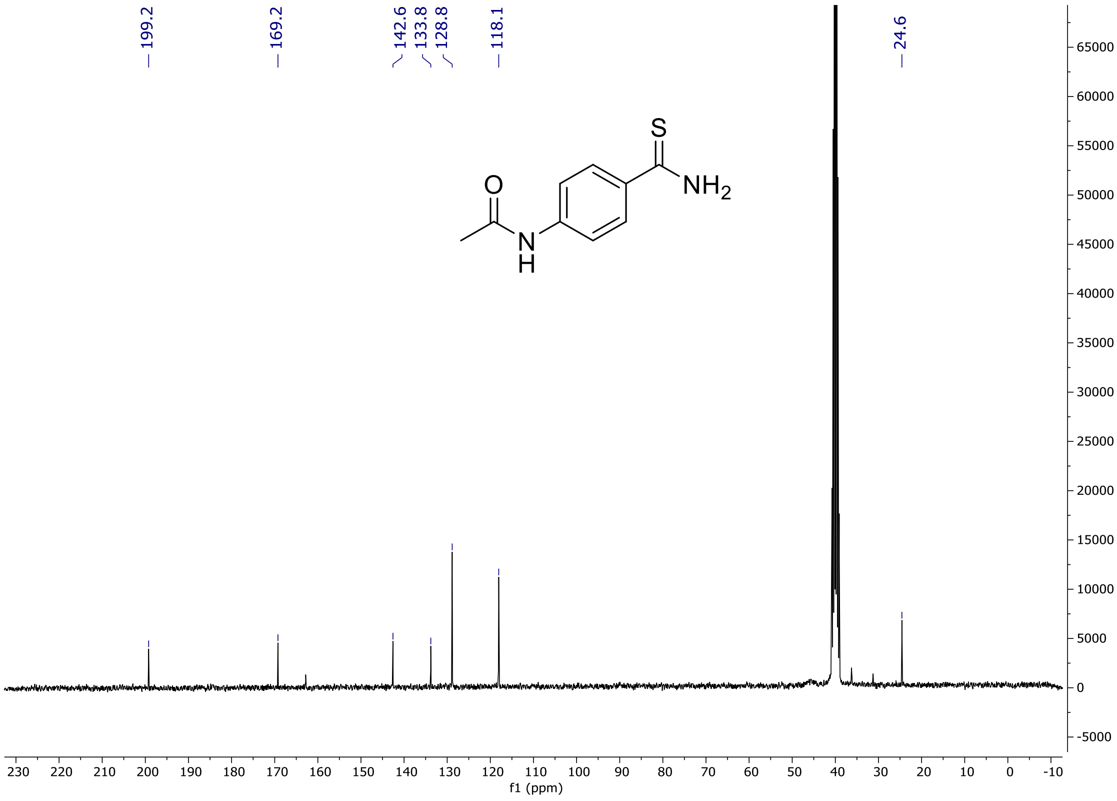

Figure SI_42: ^{13}C-NMR for **3h** in DMSO-d^{6} (75 MHz).

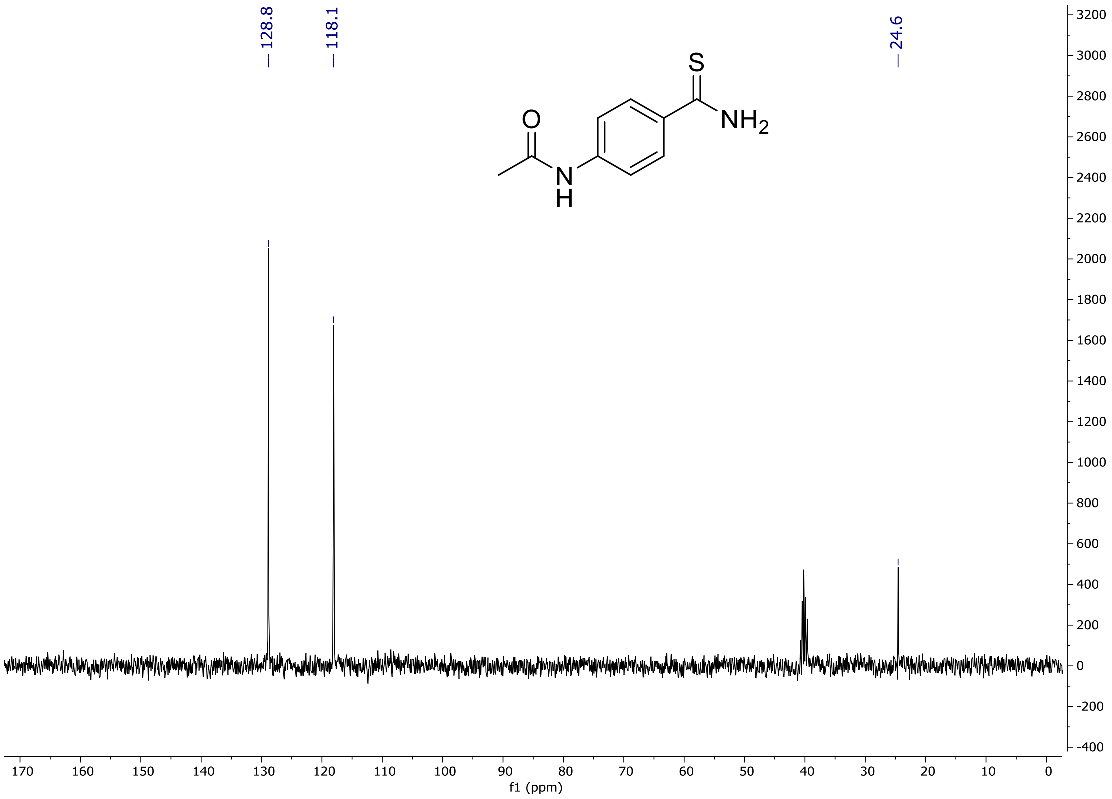

Figure SI_43: DEPT 135-NMR for **3h** in DMSO-d^6 (75 MHz).

Acquisition Parameter

Source Type	ESI	Ion Polarity	Positive	Set Nebulizer	2.0 Bar
Focus	Active	Set Capillary	4000 V	Set Dry Heater	250 °C
Scan Begin	50 m/z	Set End Plate Offset	-500 V	Set Dry Gas	5.0 l/min
Scan End	1500 m/z	Set Charging Voltage	2000 V	Set Divert Valve	Source
		Set Corona	0 nA	Set APCI Heater	0 °C

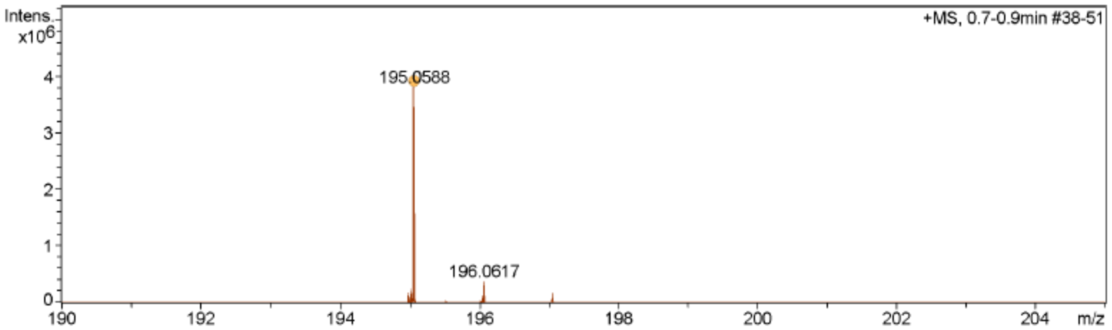

Meas. m/z	#	Ion Formula	m/z	err [ppm]	mSigma	# mSigma	Score	rdb	e⁻ Conf	N-Rule	Adduct
195.0588	1	C9H11N2OS	195.0587	-0.9	10.6	1	100.00	6.0	even	ok	M+H

Figure SI_44: HRMS (ESI$^+$, m/z) analysis of **3h**.

4-(Isopropylcarbamoyl)benzothioamide (3i)

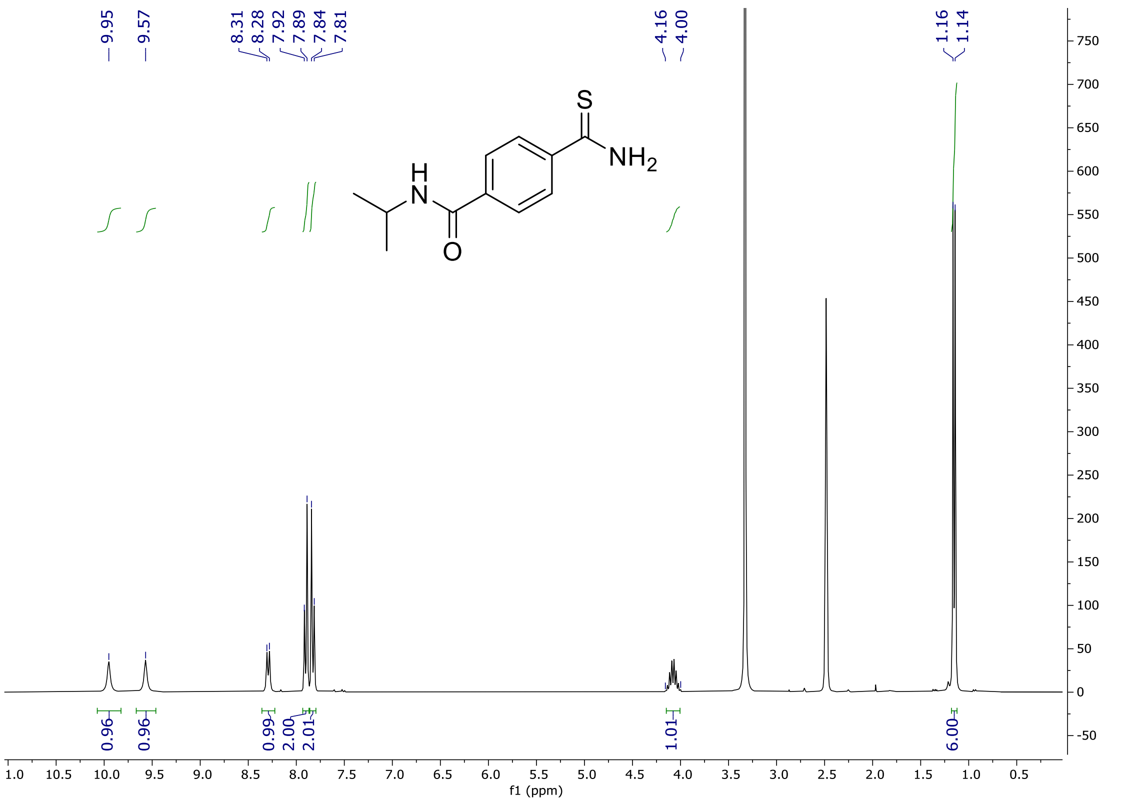

Figure SI_45: ^{1}H-NMR for **3i** in DMSO-*d*^{6} (300 MHz).

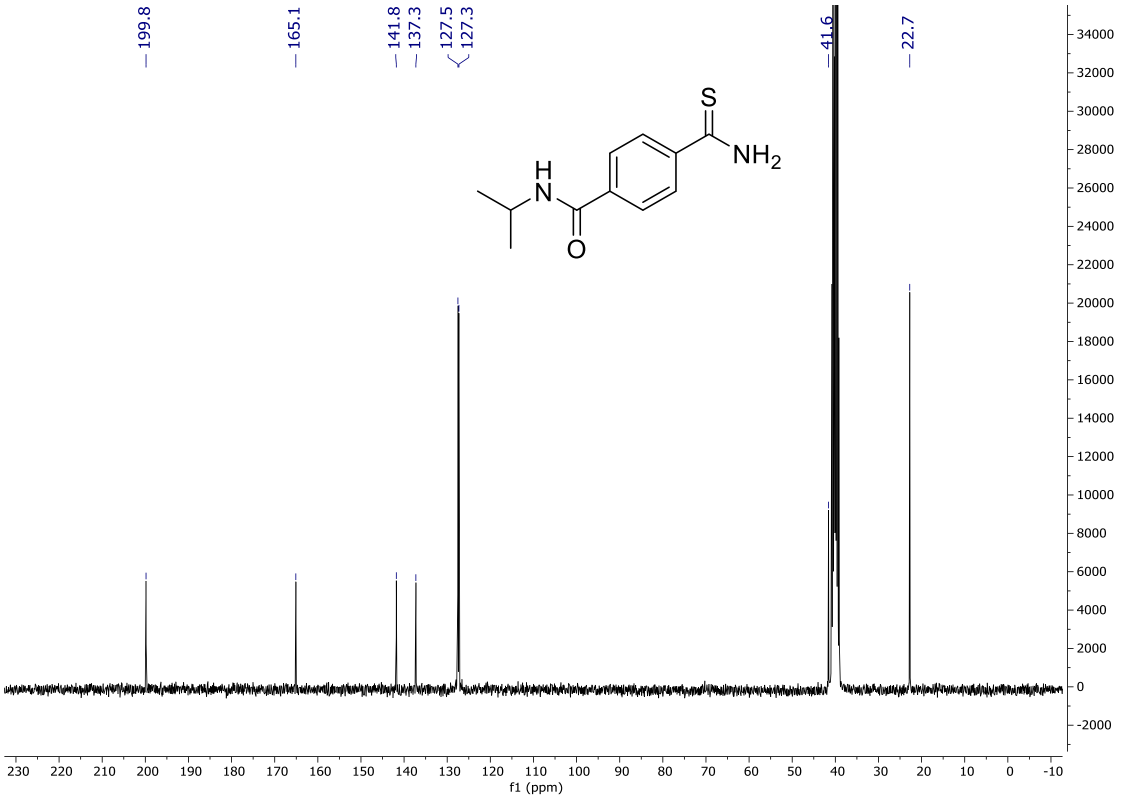

Figure SI_46: ^{13}C-NMR for **3i** in DMSO-*d*^{6} (75 MHz).

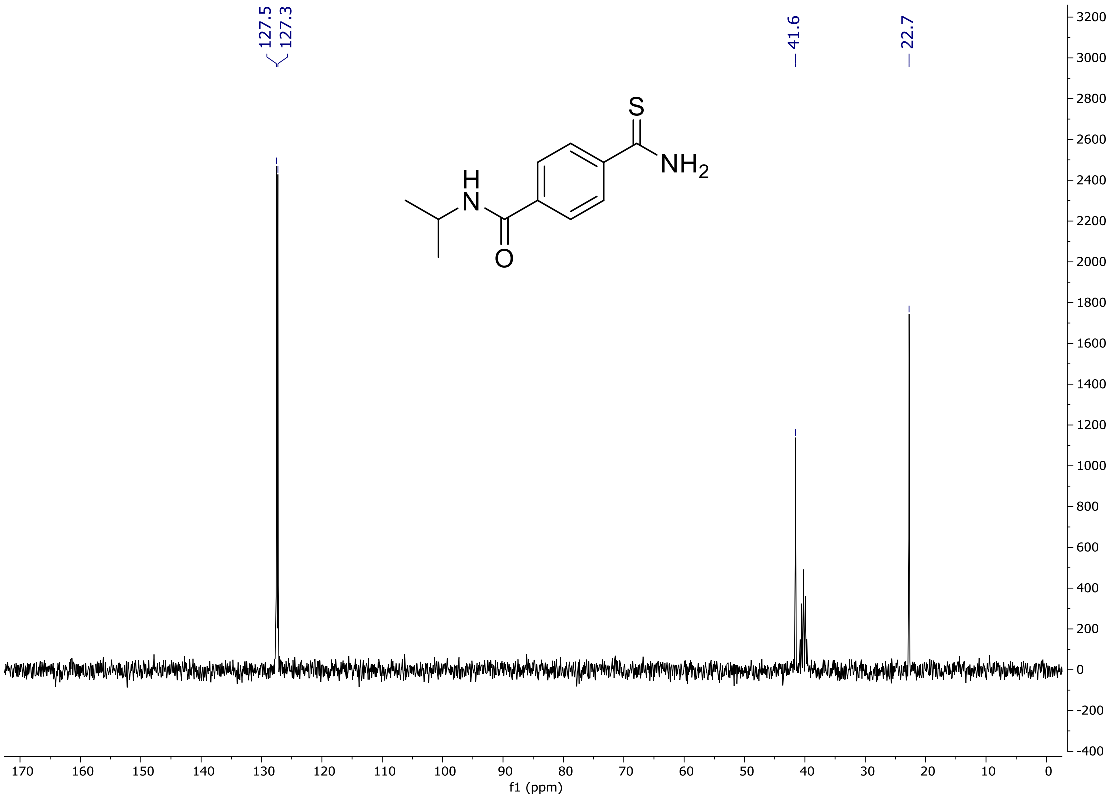

Figure SI_47: DEPT 135-NMR for **3i** in DMSO-d^6 (75 MHz).

Acquisition Parameter

Source Type	ESI	Ion Polarity	Positive	Set Nebulizer	2.0 Bar
Focus	Active	Set Capillary	4000 V	Set Dry Heater	250 °C
Scan Begin	50 m/z	Set End Plate Offset	-500 V	Set Dry Gas	5.0 l/min
Scan End	1500 m/z	Set Charging Voltage	2000 V	Set Divert Valve	Source
		Set Corona	0 nA	Set APCI Heater	0 °C

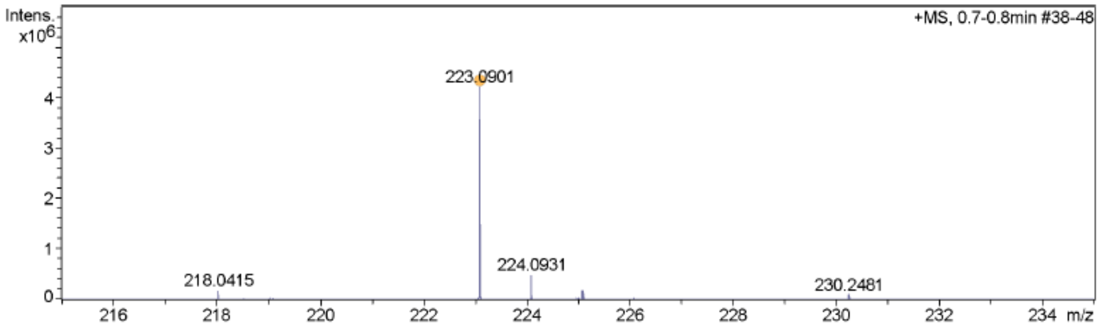

Meas. m/z	#	Ion Formula	m/z	err [ppm]	mSigma	# mSigma	Score	rdb	e⁻ Conf	N-Rule	Adduct
223.0901	1	C11H15N2OS	223.0900	-0.8	12.6	1	100.00	6.0	even	ok	M+H

Figure SI_48: HRMS (ESI$^+$, m/z) analysis of **3i**.

4-(Pyrrolidine-1-carbonyl)benzothioamide (3j)

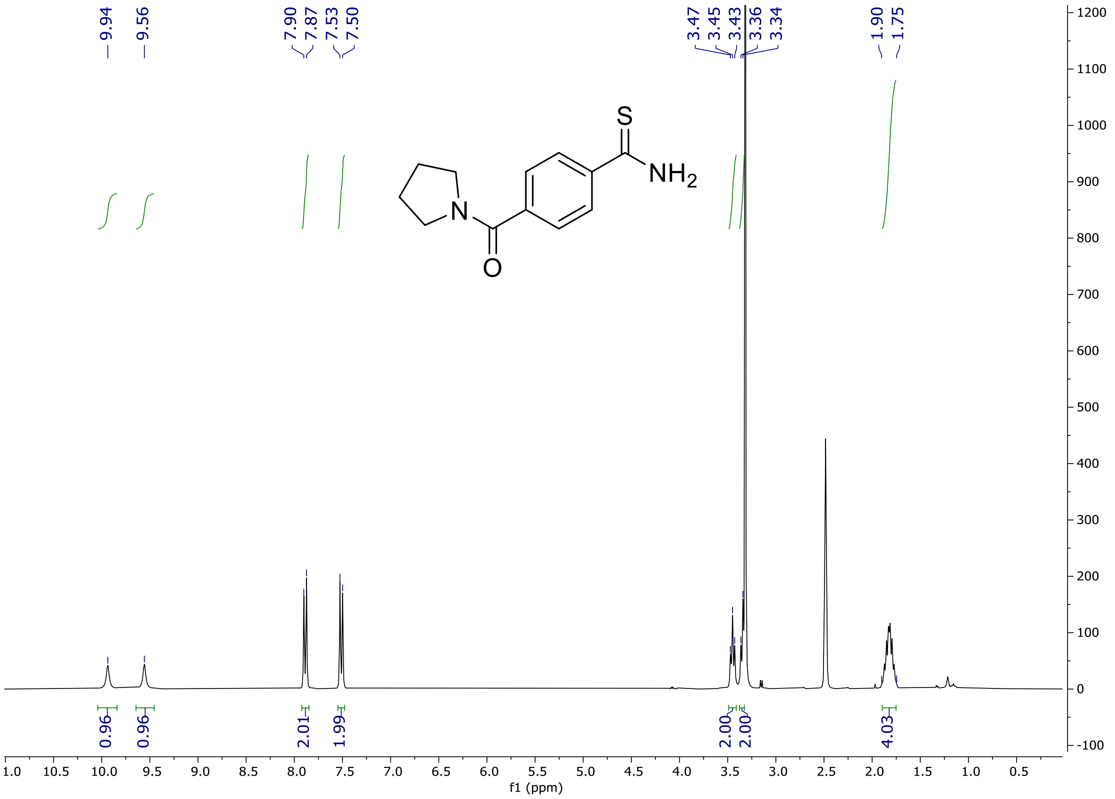

Figure SI_49: ^{1}H-NMR for **3j** in DMSO-*d*^{6} (300 MHz).

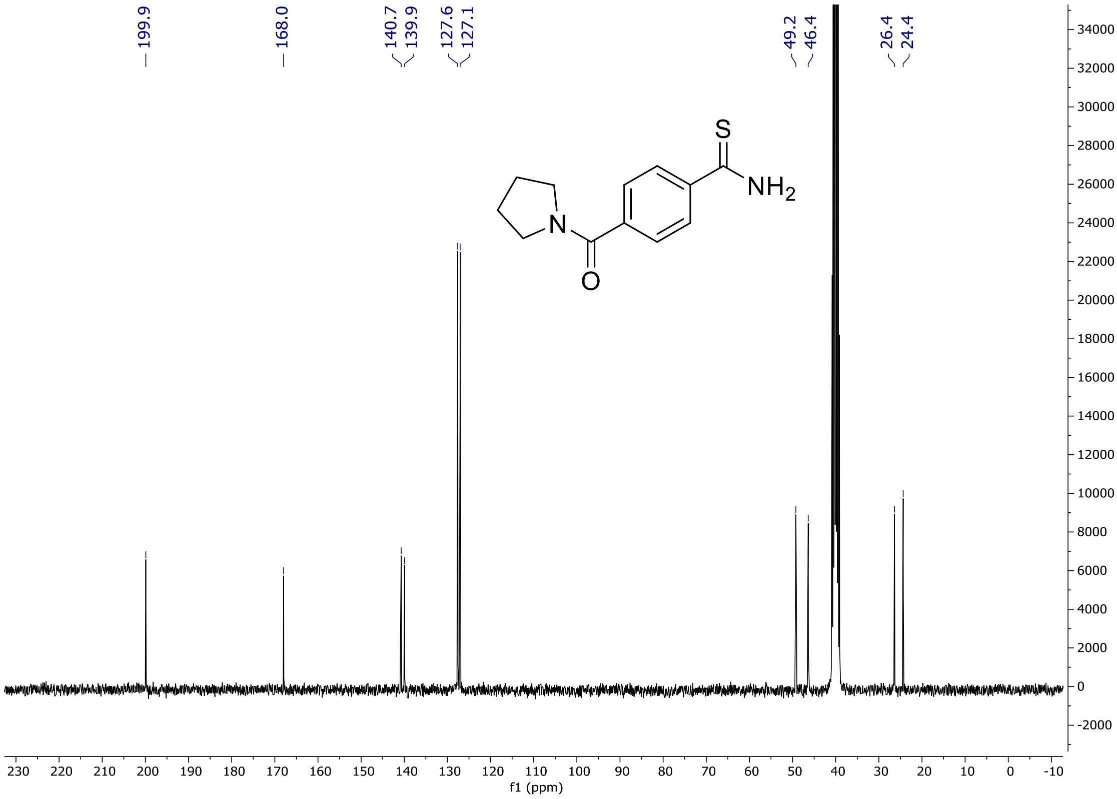

Figure SI_50: ^{13}C-NMR for **3j** in DMSO-*d*^{6} (75 MHz).

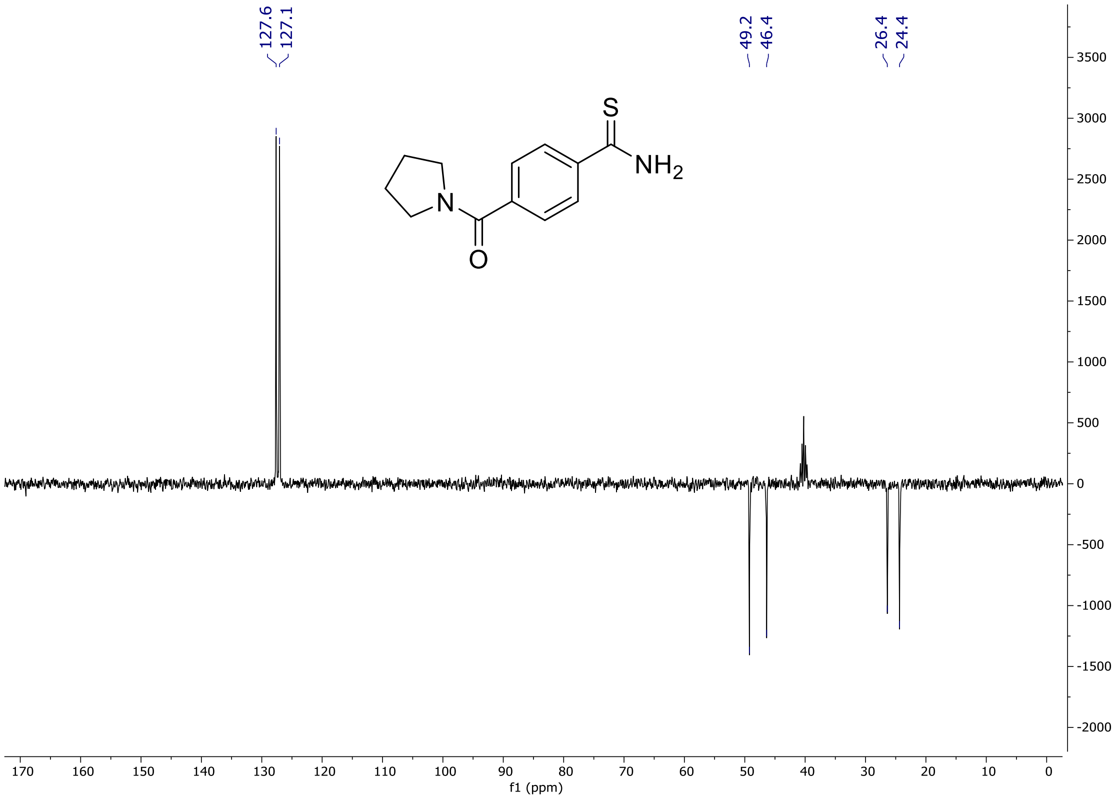

Figure SI_51: DEPT 135-NMR for **3j** in DMSO-d^6 (75 MHz).

Acquisition Parameter

Source Type	ESI	Ion Polarity	Positive	Set Nebulizer	2.0 Bar
Focus	Active	Set Capillary	4000 V	Set Dry Heater	250 °C
Scan Begin	50 m/z	Set End Plate Offset	-500 V	Set Dry Gas	5.0 l/min
Scan End	1500 m/z	Set Charging Voltage	2000 V	Set Divert Valve	Source
		Set Corona	0 nA	Set APCI Heater	0 °C

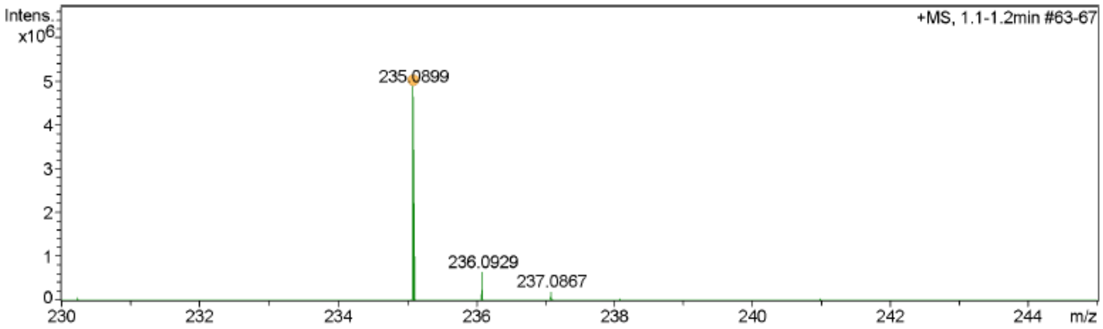

Meas. m/z	#	Ion Formula	m/z	err [ppm]	mSigma	# mSigma	Score	rdb	e⁻ Conf	N-Rule	Adduct
235.0899	1	C12H15N2OS	235.0900	0.1	10.2	1	100.00	7.0	even	ok	M+H

Figure SI_52: HRMS (ESI$^+$, m/z) analysis of **3j**.

4-Acetylbenzothioamide (3k)

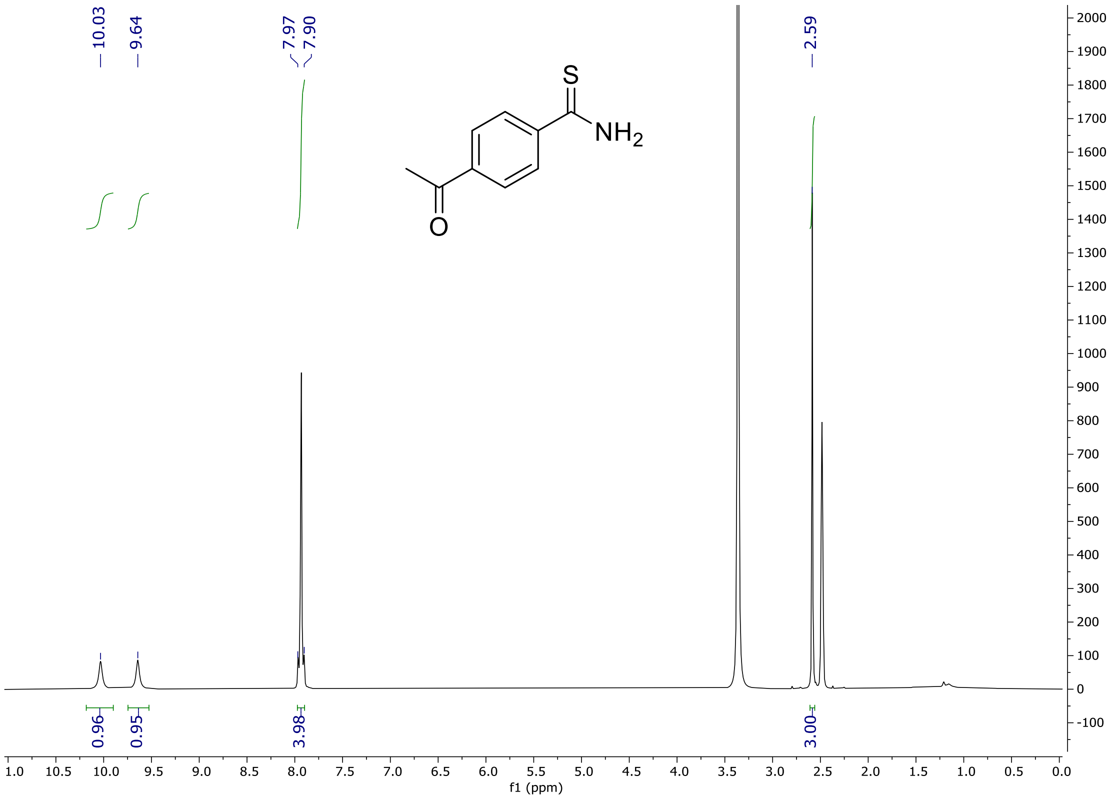

Figure SI_53: ^{1}H-NMR for **3k** in DMSO-d^{6} (300 MHz).

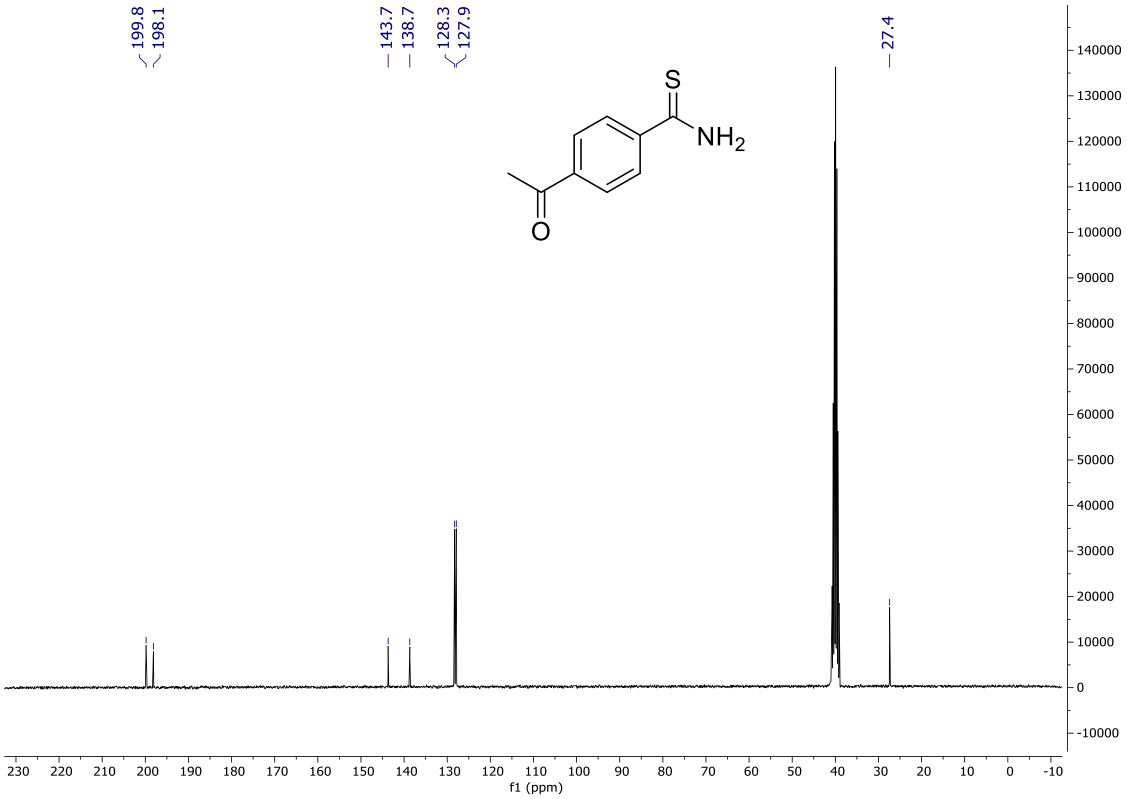

Figure SI_54: ^{13}C-NMR for **3k** in DMSO-d^{6} (75 MHz).

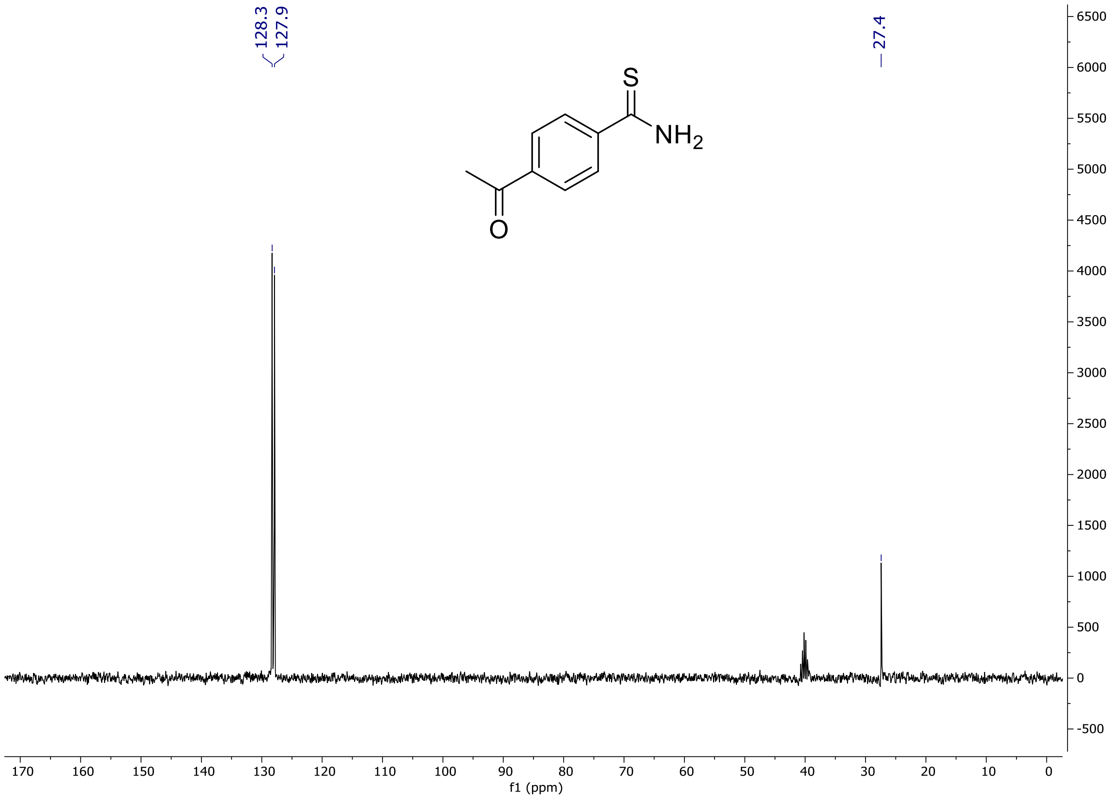

Figure SI_55: DEPT 135-NMR for **3k** in DMSO-d^6 (75 MHz).

Acquisition Parameter

Source Type	ESI	Ion Polarity	Positive	Set Nebulizer	2.0 Bar
Focus	Active	Set Capillary	4000 V	Set Dry Heater	250 °C
Scan Begin	50 m/z	Set End Plate Offset	-500 V	Set Dry Gas	5.0 l/min
Scan End	1500 m/z	Set Charging Voltage	2000 V	Set Divert Valve	Source
		Set Corona	0 nA	Set APCI Heater	0 °C

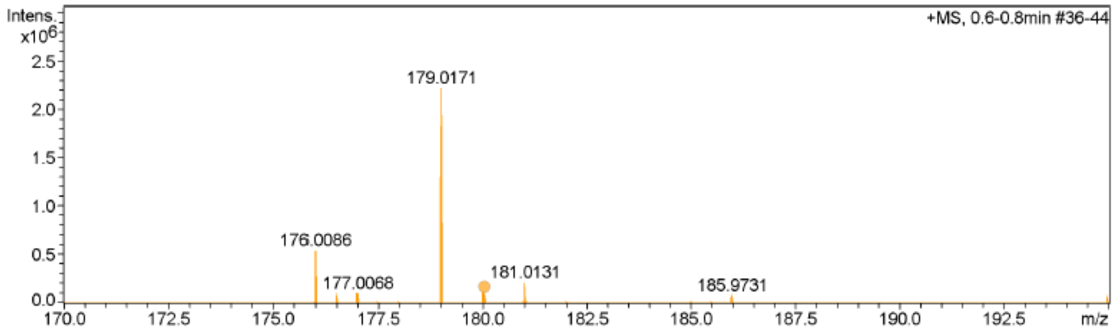

Meas. m/z	#	Ion Formula	m/z	err [ppm]	mSigma	# mSigma	Score	rdb	e⁻ Conf	N-Rule	Adduct
180.0477	1	C9H10NOS	180.0478	0.1	30.3	1	100.00	6.0	even	ok	M+H

Figure SI_56: HRMS (ESI$^+$, m/z) analysis of **3k.**

4-(Methoxycarbonyl)benzothioamide (3l)

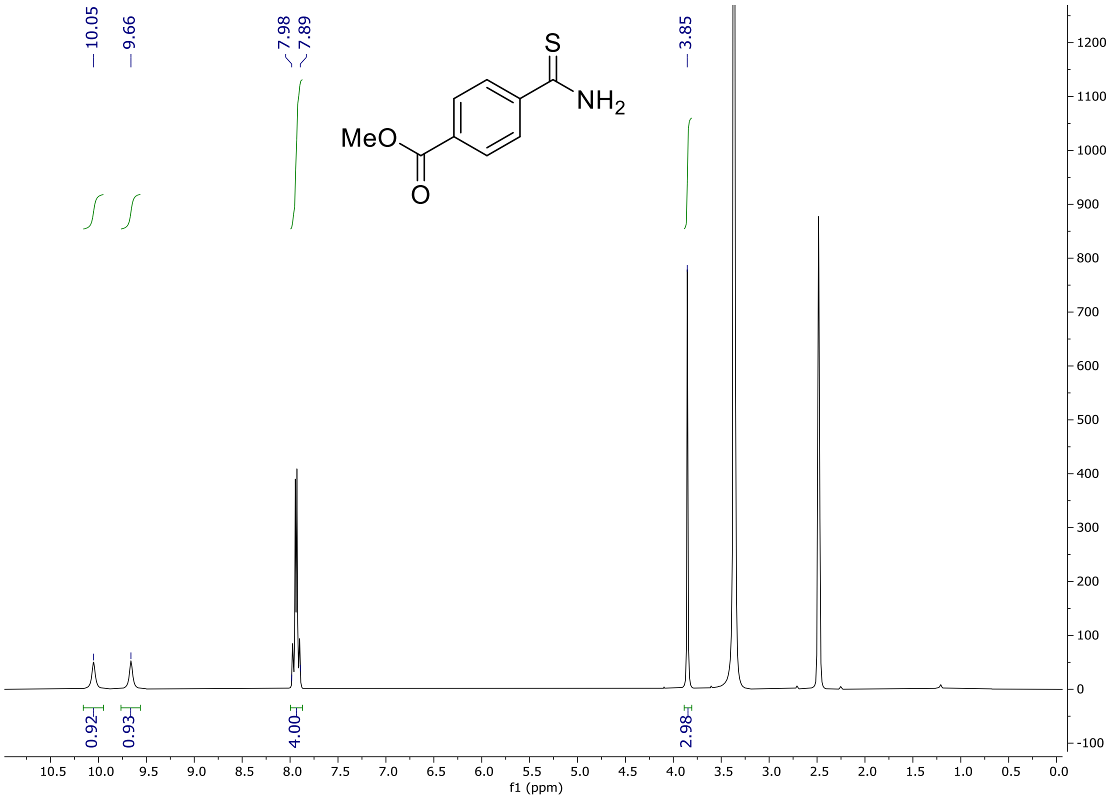

Figure SI_57: ^{1}H-NMR for **3l** in DMSO-d^{6} (300 MHz).

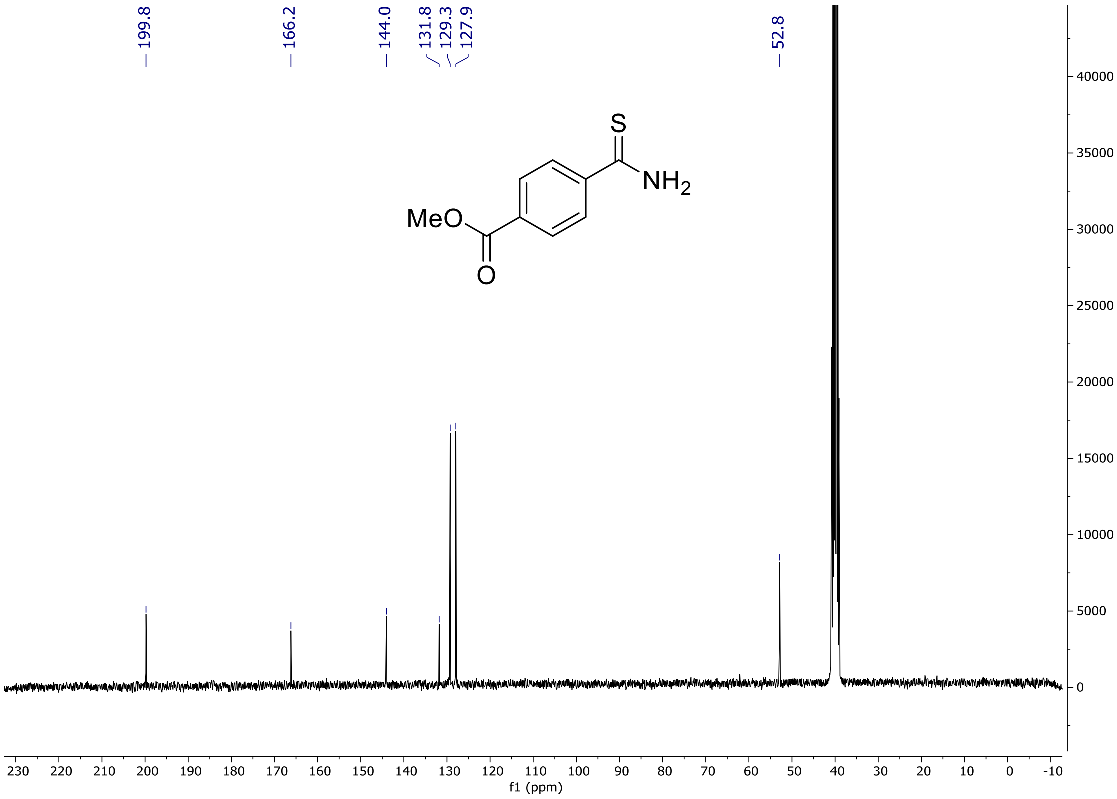

Figure SI_58: ^{13}C-NMR for **3l** in DMSO-d^{6} (75 MHz).

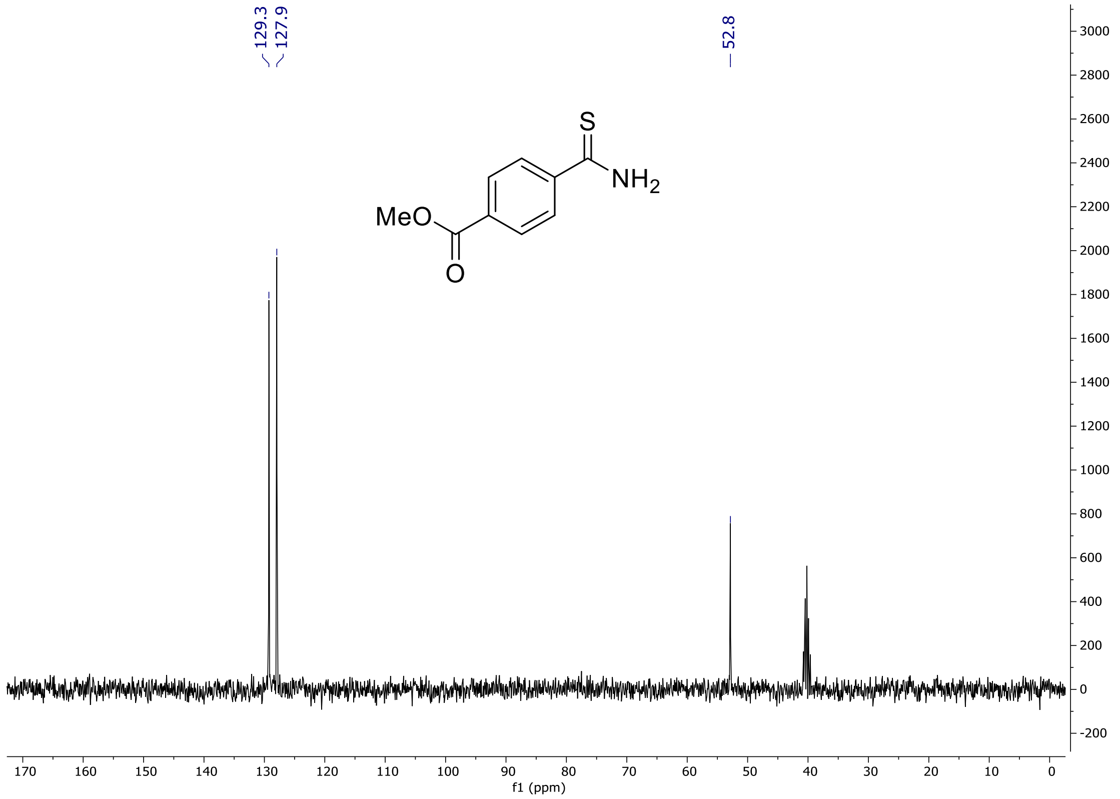

Figure SI_59: DEPT 135-NMR for **3l** in DMSO-d^6 (75 MHz).

4-(Trifluoromethyl)benzothioamide (3m)

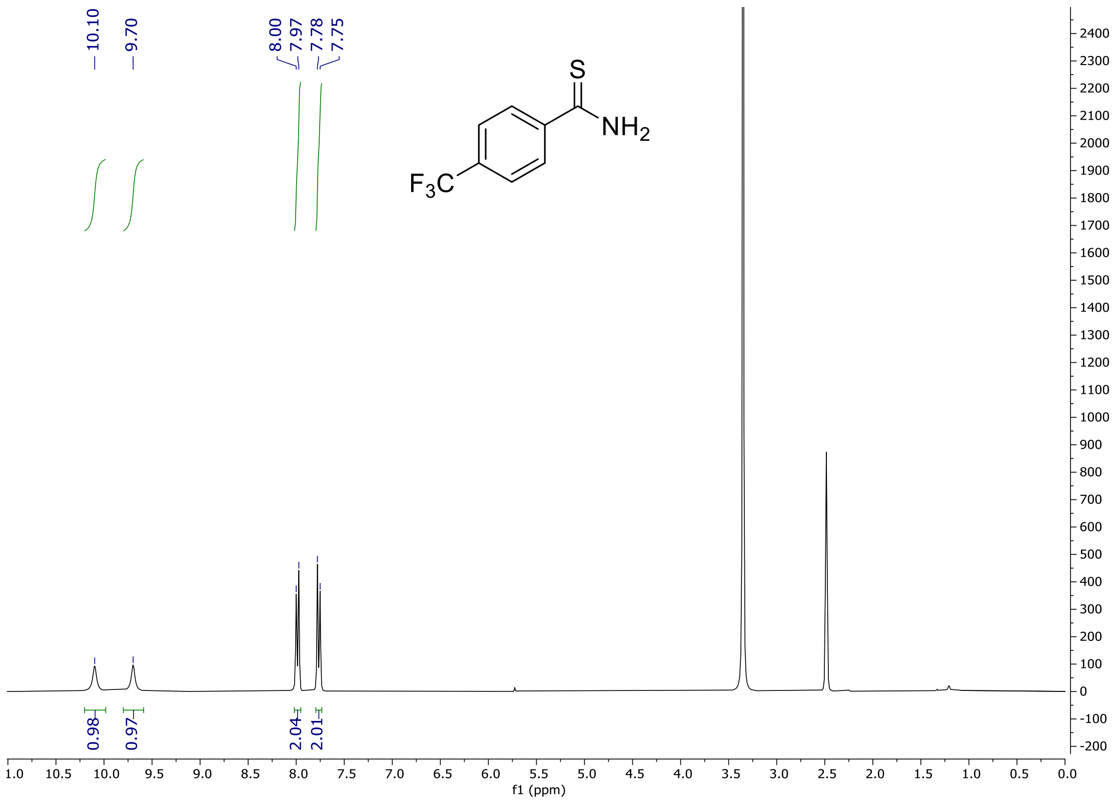

Figure SI_60: ^{1}H-NMR for **3m** in DMSO-d^6 (300 MHz).

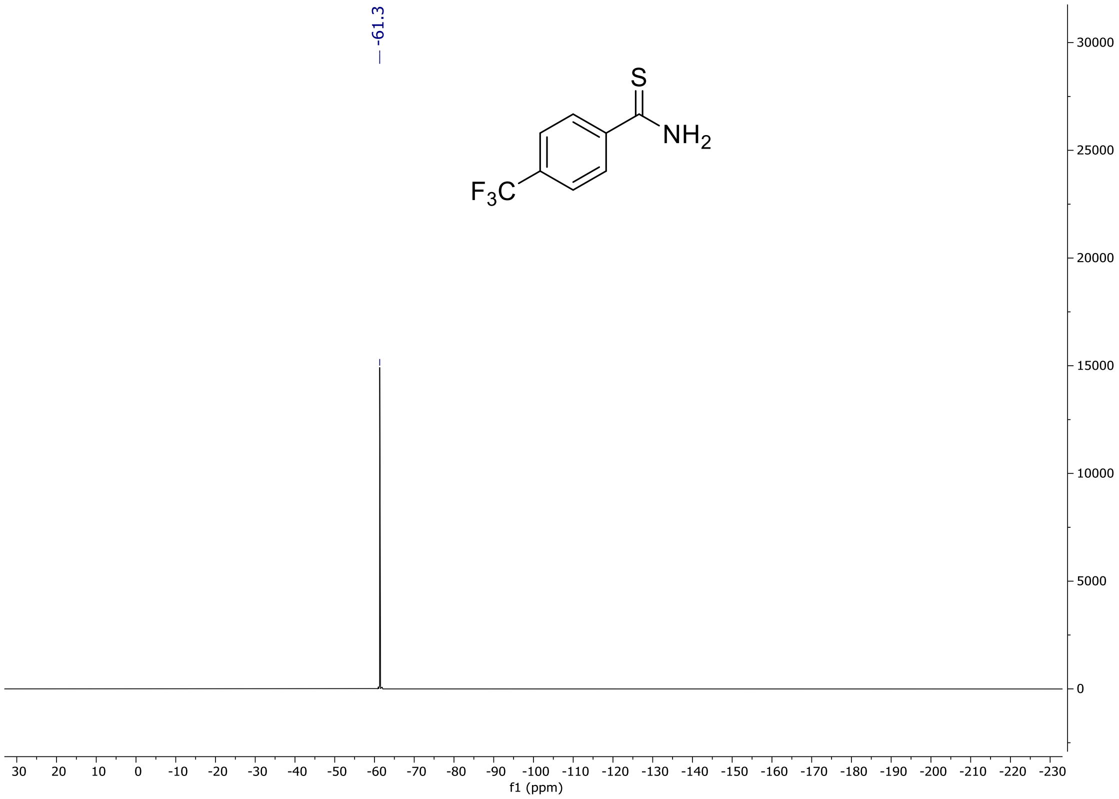

Figure SI_61: ^{19}F-NMR for **3m** in DMSO-d^6 (282 MHz).

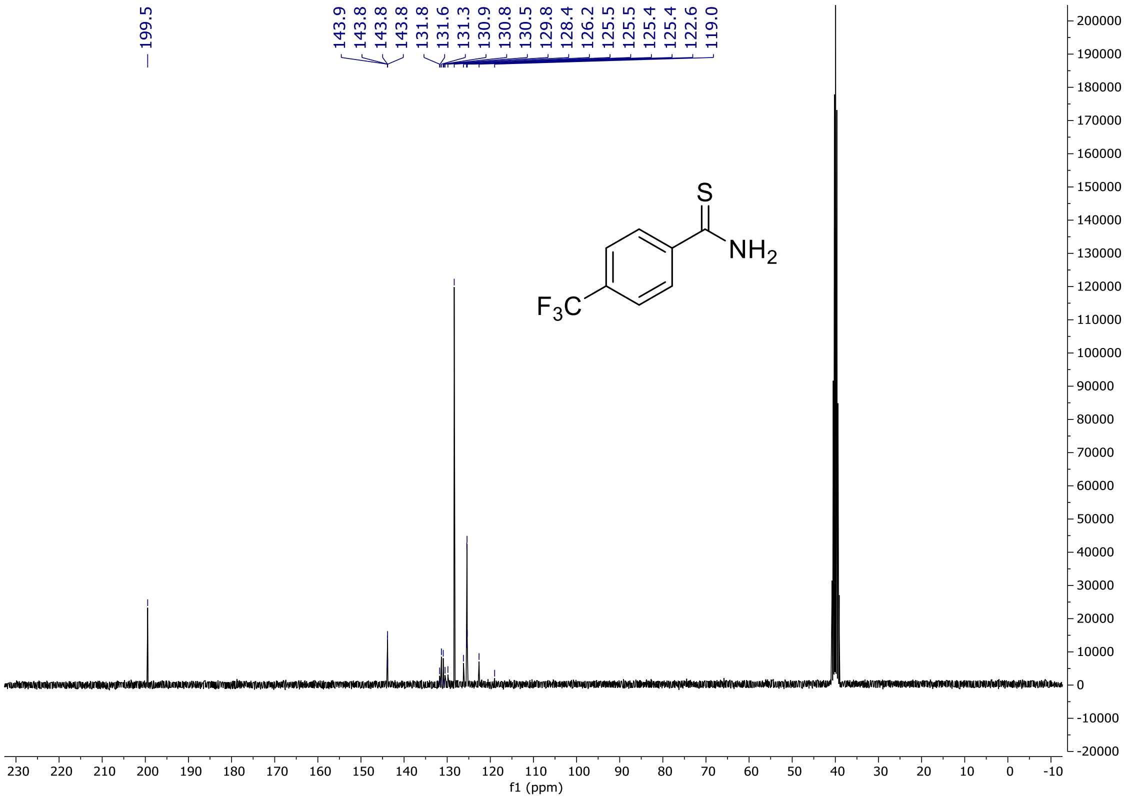

Figure SI_62: ^{13}C-NMR for **3m** in DMSO-d^6 (75 MHz).

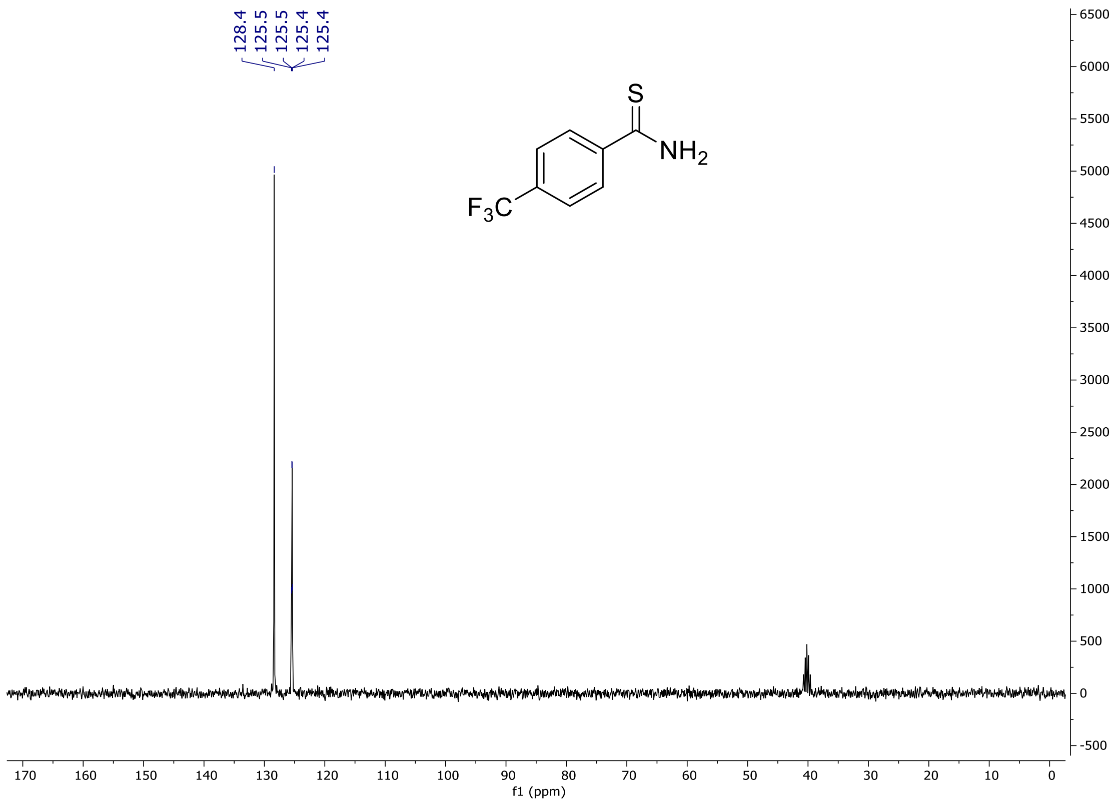

Figure SI_63: DEPT 135-NMR for **3m** in DMSO-d^6 (75 MHz).

Furan-2-carbothioamide (3n)

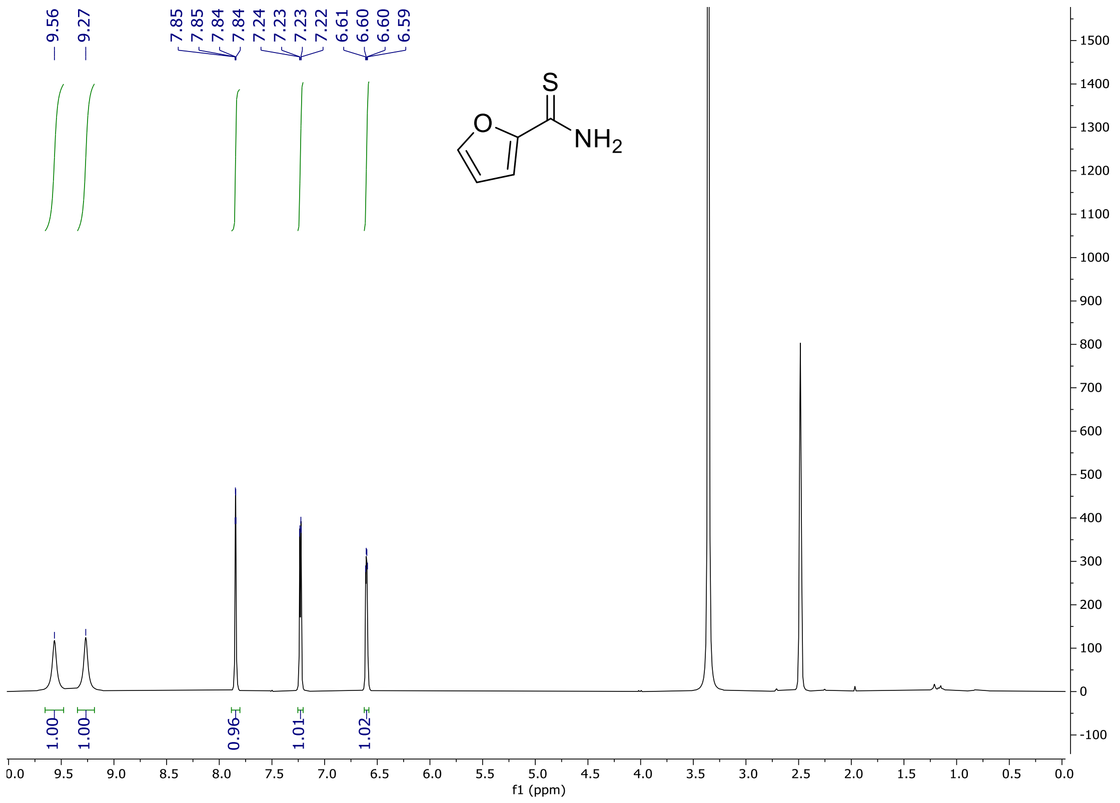

Figure SI_64: ^{1}H-NMR for **3n** in DMSO-d^6 (300 MHz).

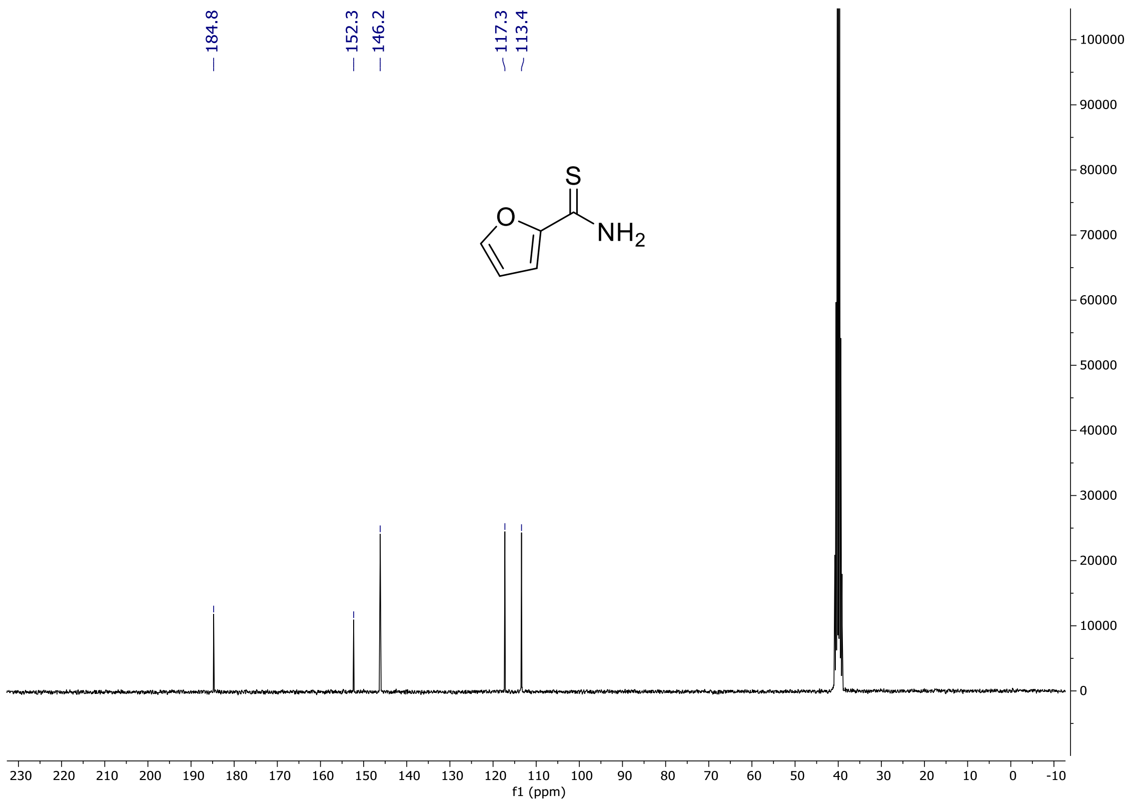

Figure SI_65: ^{13}C-NMR for **3n** in DMSO-d^6 (75 MHz).

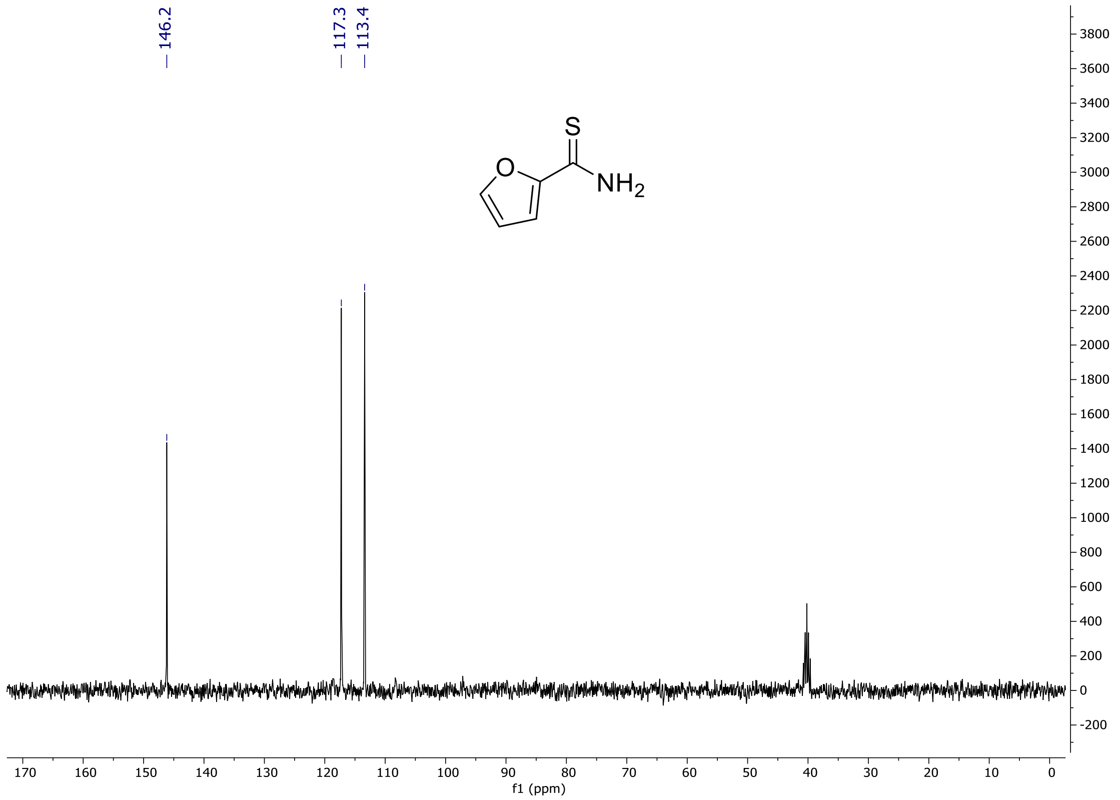

Figure SI_66: DEPT 135-NMR for **3n** in DMSO-d^6 (75 MHz).

Acquisition Parameter

Source Type	ESI	Ion Polarity	Positive	Set Nebulizer	2.0 Bar
Focus	Active	Set Capillary	4000 V	Set Dry Heater	250 °C
Scan Begin	50 m/z	Set End Plate Offset	-500 V	Set Dry Gas	5.0 l/min
Scan End	1500 m/z	Set Charging Voltage	2000 V	Set Divert Valve	Source
		Set Corona	0 nA	Set APCI Heater	0 °C

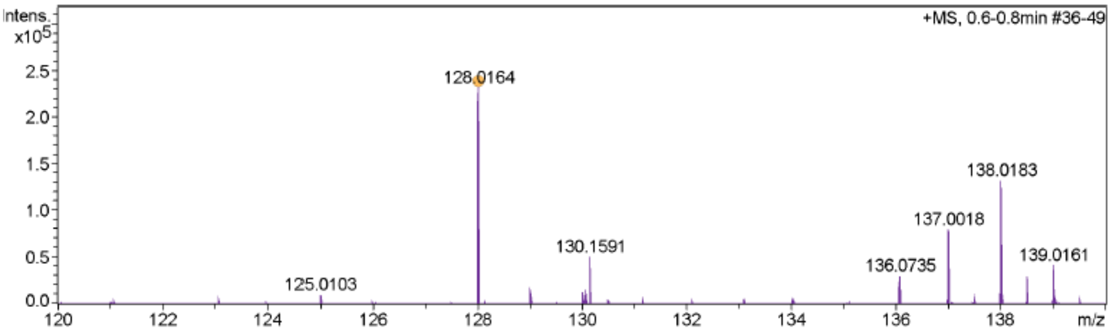

Meas. m/z	#	Ion Formula	m/z	err [ppm]	mSigma	# mSigma	Score	rdb	e⁻ Conf	N-Rule	Adduct
128.0164	1	C5H6NOS	128.0165	0.4	9.6	1	100.00	4.0	even	ok	M+H

Figure SI_67: HRMS (ESI$^+$, m/z) analysis of **3n**.

Thiophene-2-carbothioamide (3o)

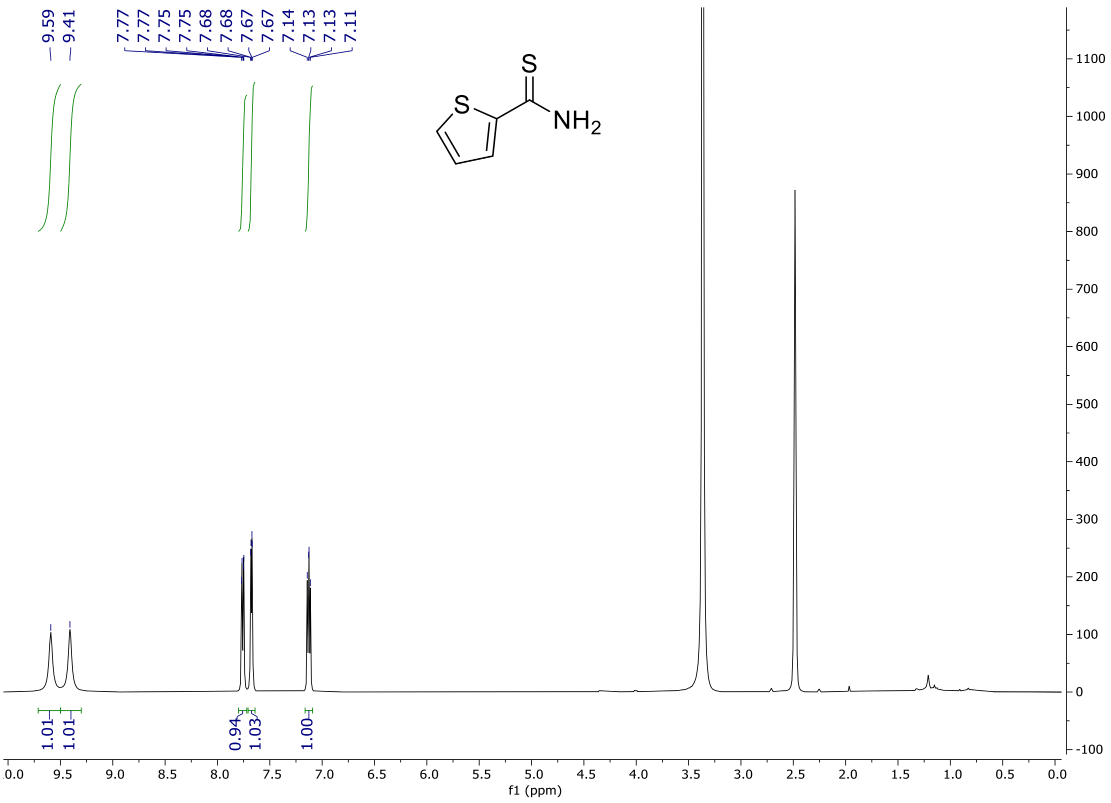

Figure SI_68: ^{1}H-NMR for **3o** in DMSO-d^6 (300 MHz).

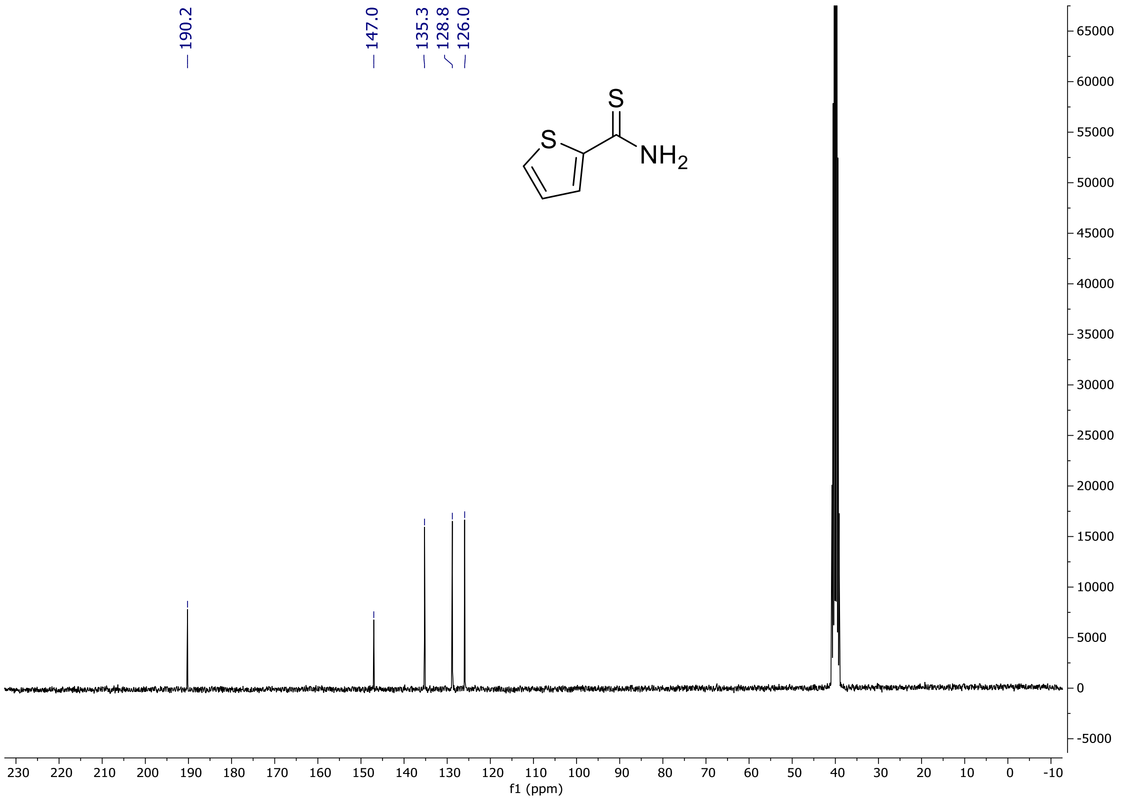

Figure SI_69: ^{13}C-NMR for **3o** in DMSO-d^6 (75 MHz).

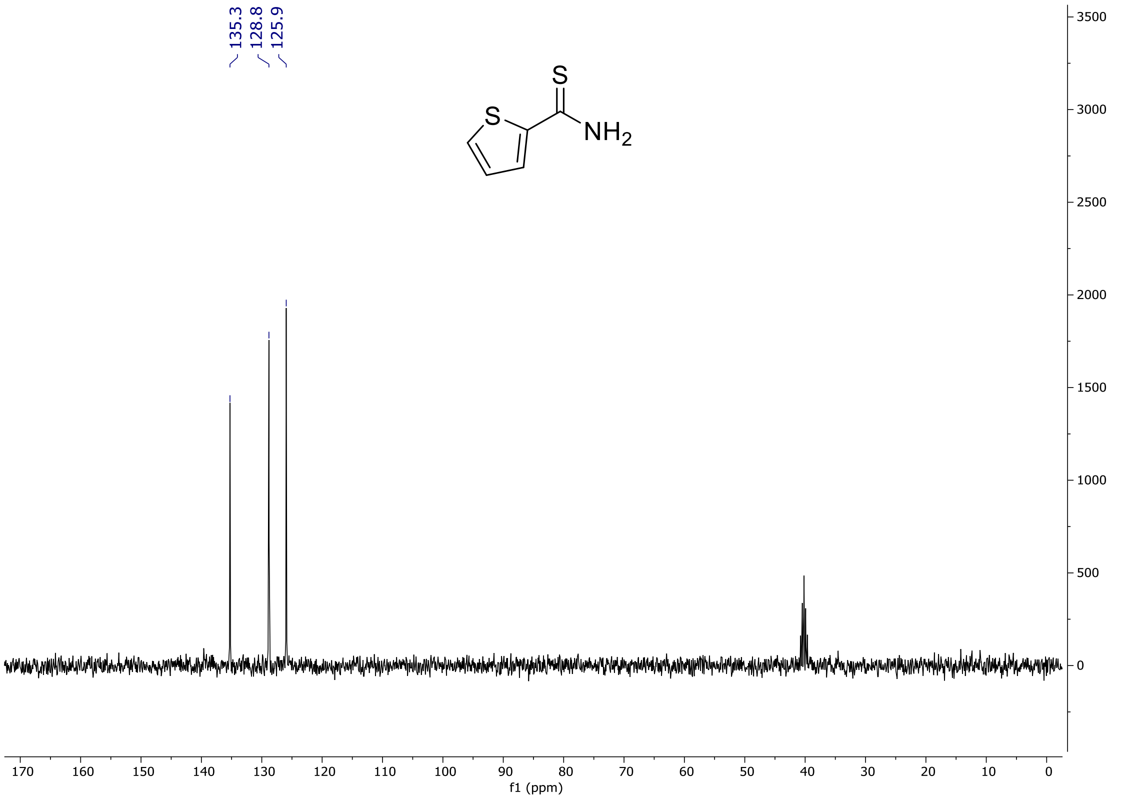

Figure SI_70: DEPT 135-NMR for **3o** in DMSO-d^6 (75 MHz).

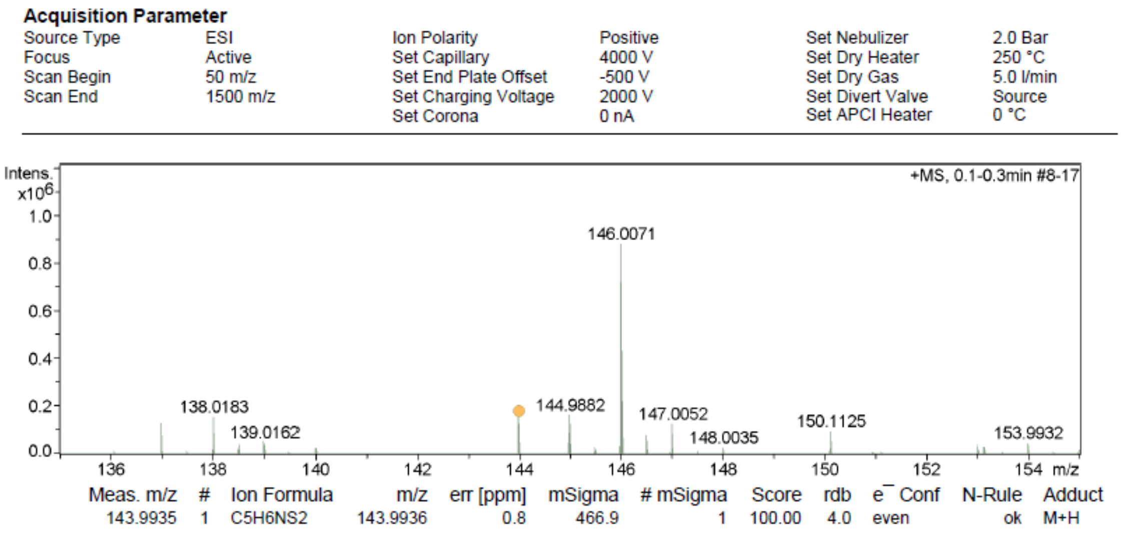

Figure SI_71: HRMS (ESI$^+$, m/z) analysis of **3o**.

Pyridine-3-carbothioamide (3p)

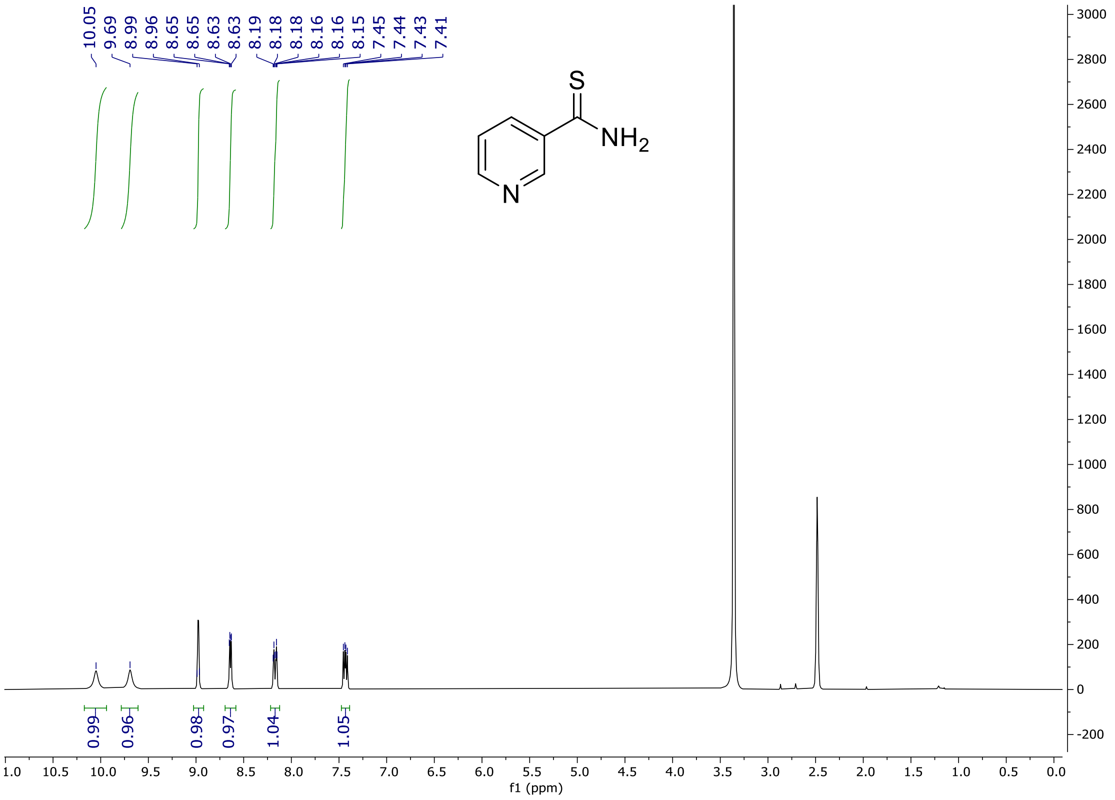

Figure SI_72: ^{1}H-NMR for **3p** in DMSO-d^6 (300 MHz).

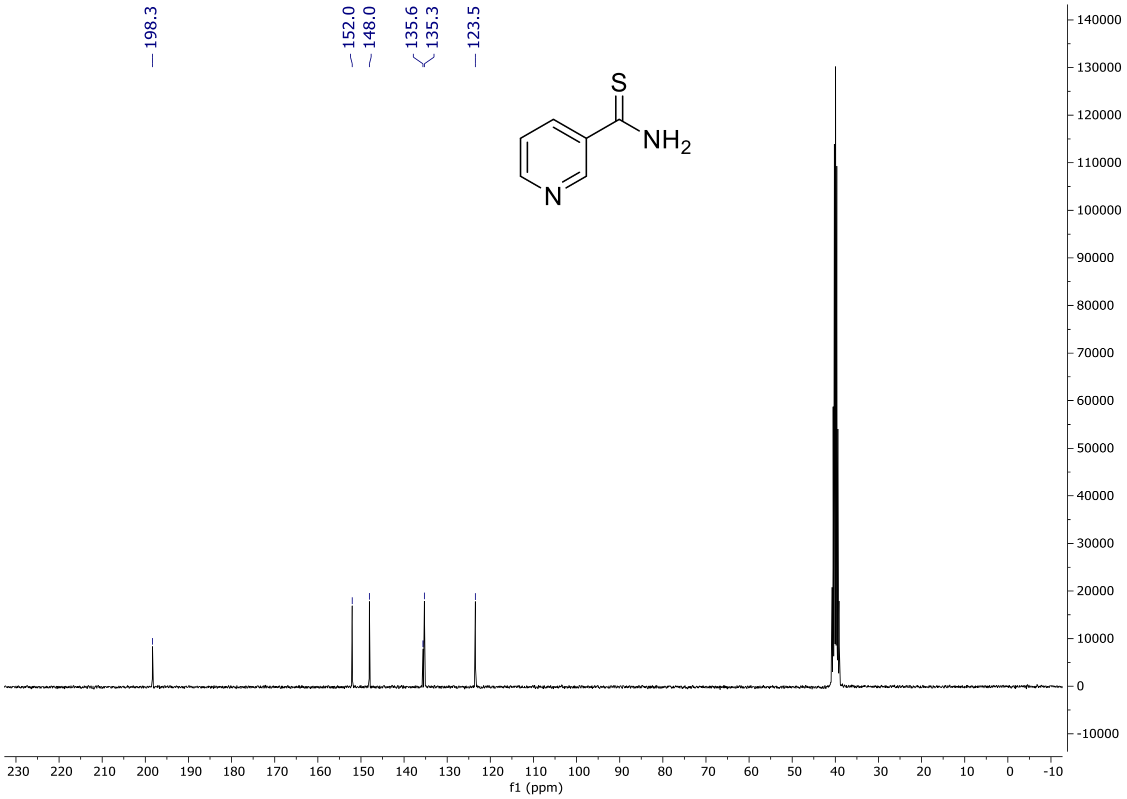

Figure SI_73: ^{13}C-NMR for **3p** in DMSO-*d*^{6} (75 MHz).

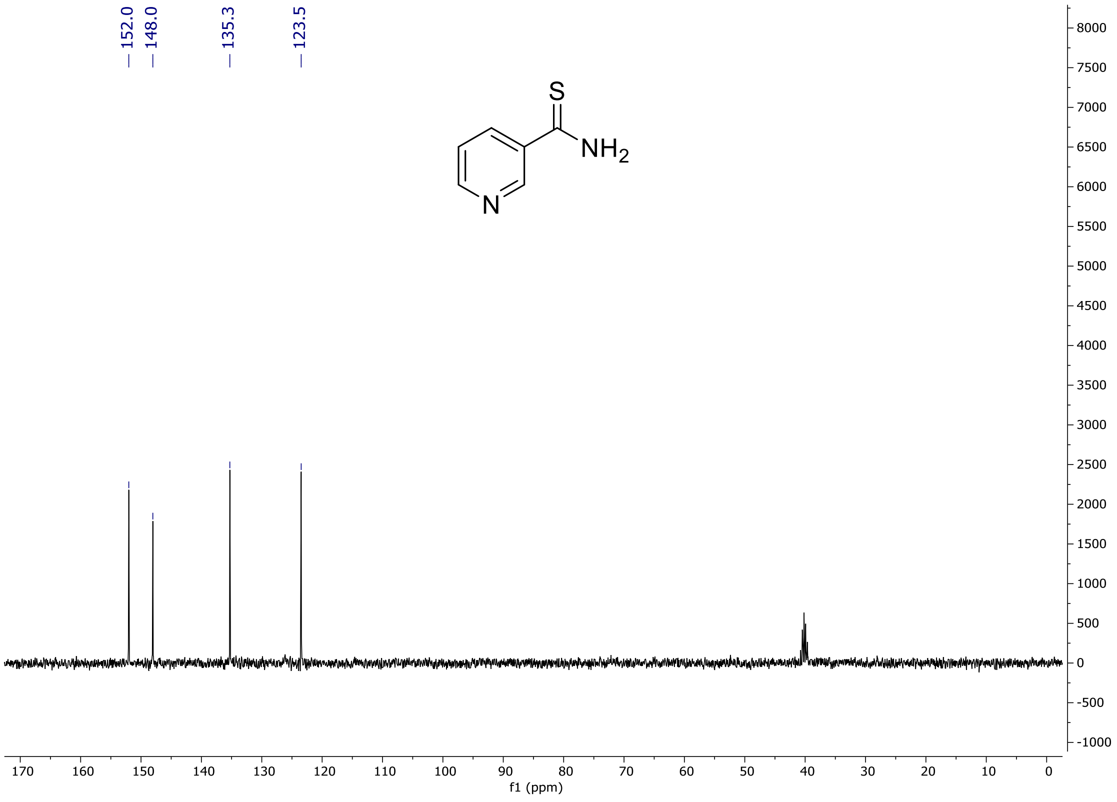

Figure SI_74: DEPT 135-NMR for **3p** in DMSO-*d*^{6} (75 MHz).

Naphthalene-1-carbothioamide (3q)

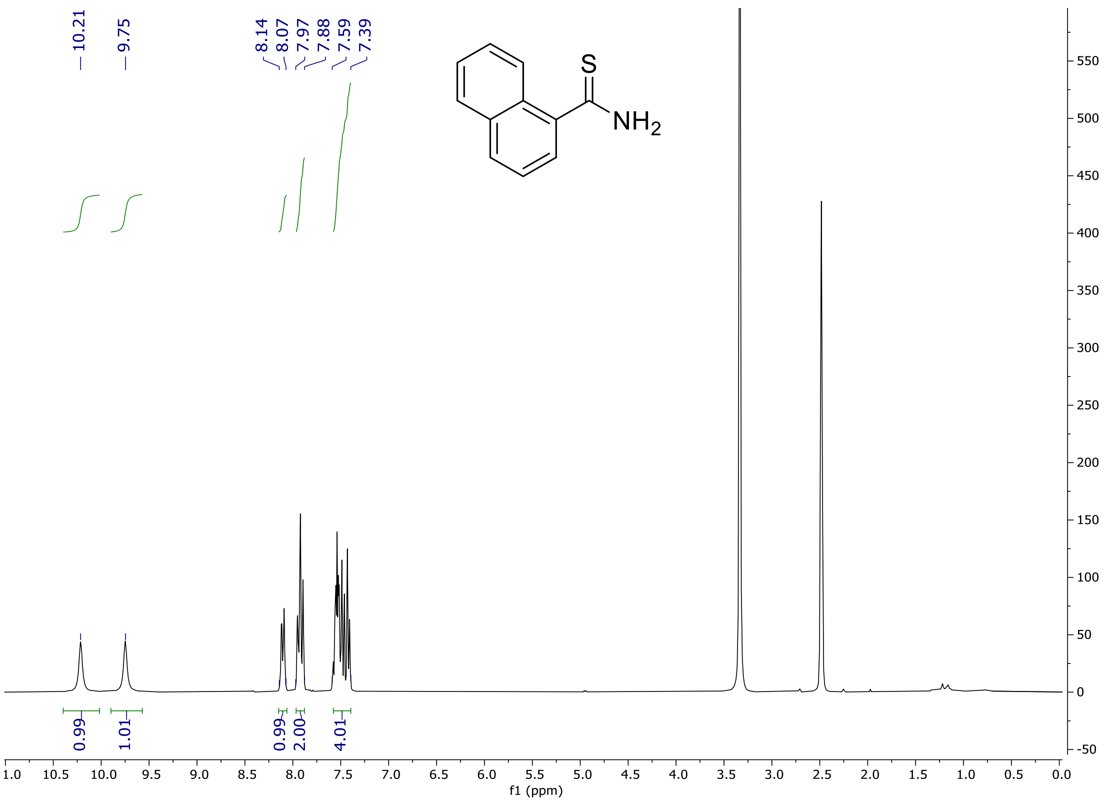

Figure SI_75: ^{1}H-NMR for **3q** in DMSO-d^{6} (300 MHz).

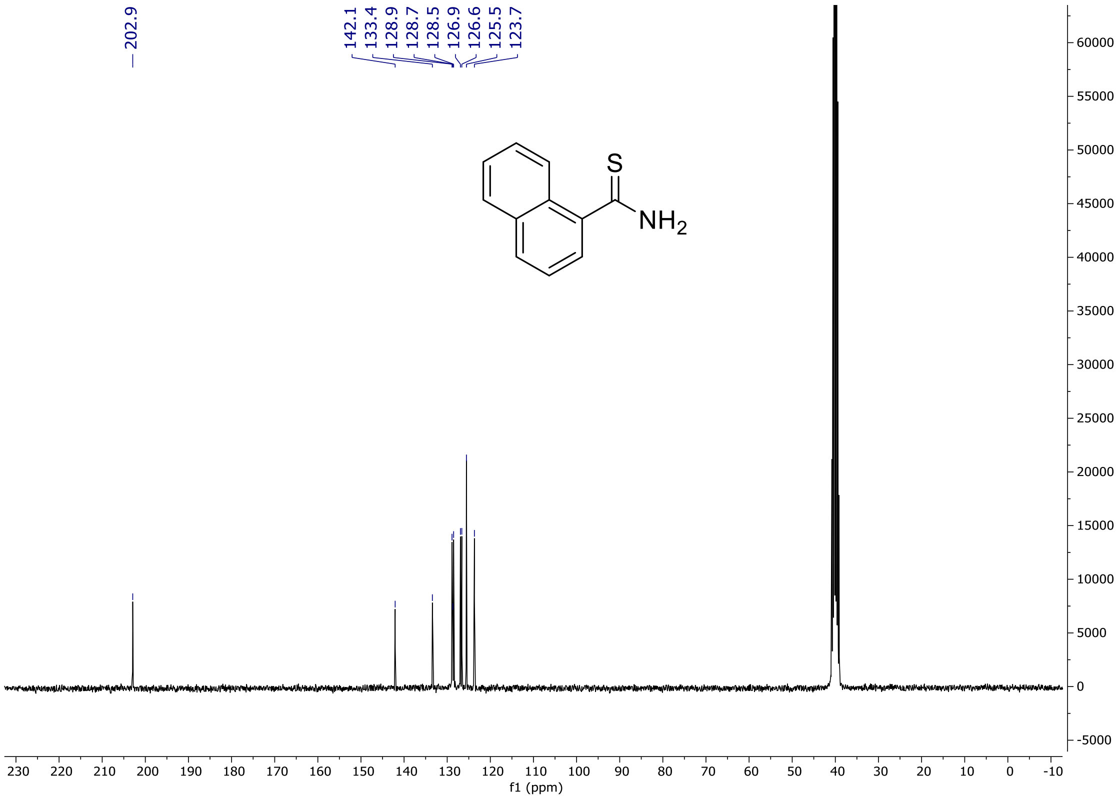

Figure SI_76: ^{13}C-NMR for **3q** in DMSO-d^{6} (75 MHz).

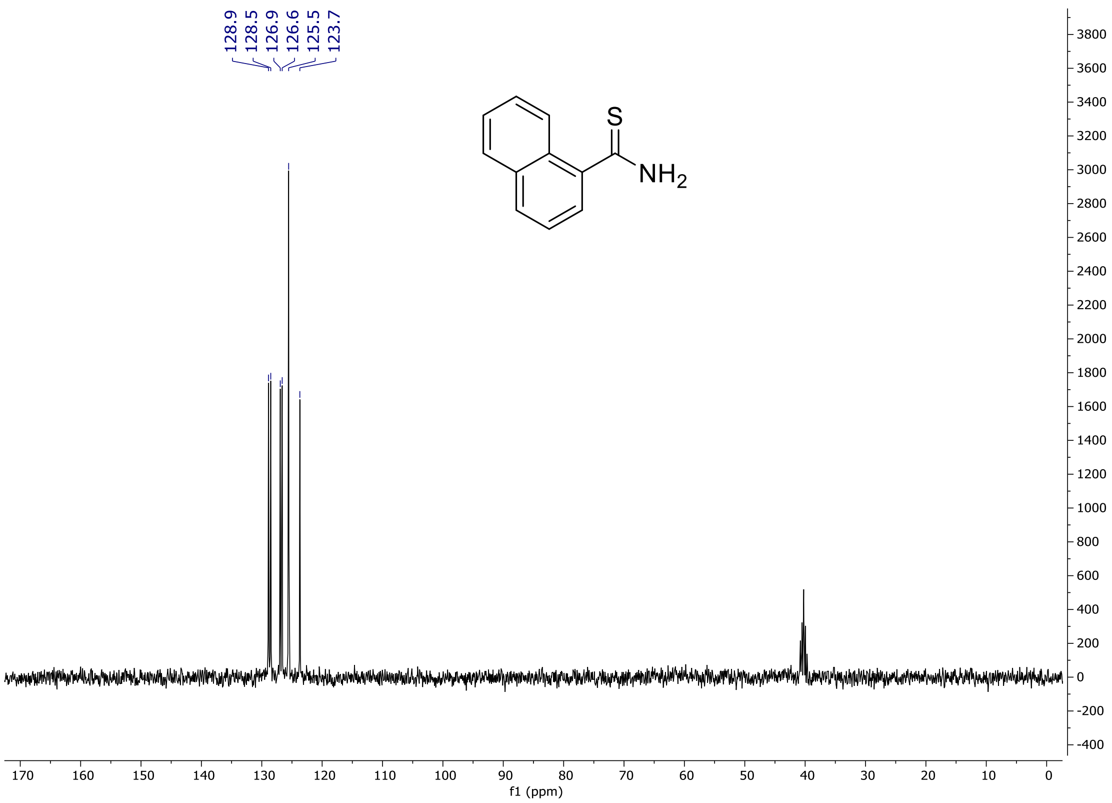

Figure SI_77: DEPT 135-NMR for **3q** in DMSO-d^6 (75 MHz).

Anthracene-9-carbothioamide (3r)

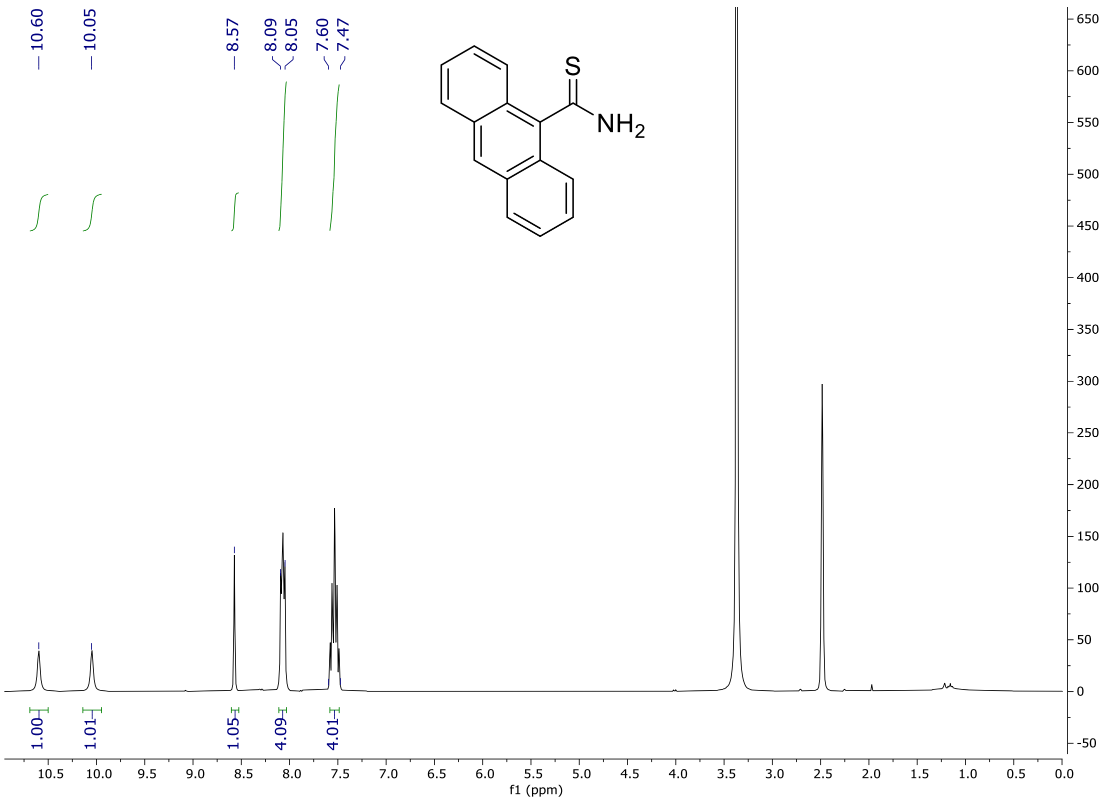

Figure SI_78: ^{1}H-NMR for **3r** in DMSO-d^6 (300 MHz).

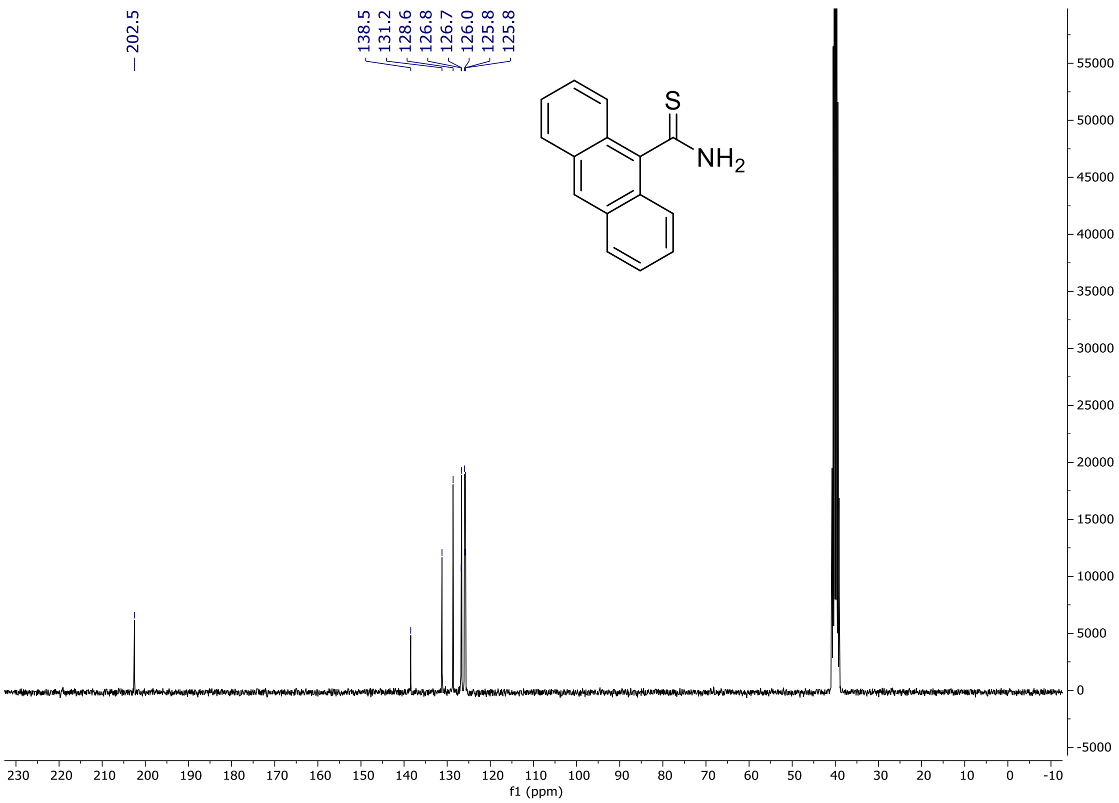

Figure SI_79: ^{13}C-NMR for **3r** in DMSO-d^6 (75 MHz).

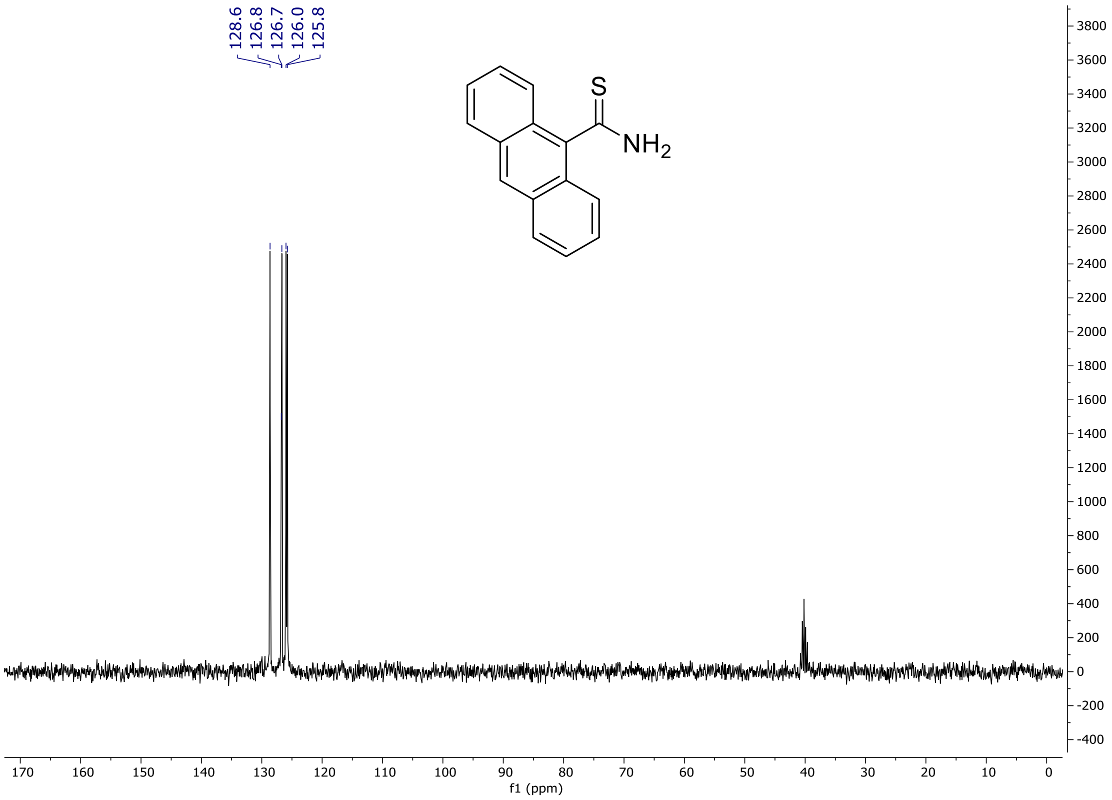

Figure SI_80: DEPT 135-NMR for **3r** in DMSO-d^6 (75 MHz).

Acquisition Parameter

Source Type	ESI	Ion Polarity	Positive	Set Nebulizer	2.0 Bar
Focus	Active	Set Capillary	4000 V	Set Dry Heater	250 °C
Scan Begin	50 m/z	Set End Plate Offset	-500 V	Set Dry Gas	5.0 l/min
Scan End	1500 m/z	Set Charging Voltage	2000 V	Set Divert Valve	Source
		Set Corona	0 nA	Set APCI Heater	0 °C

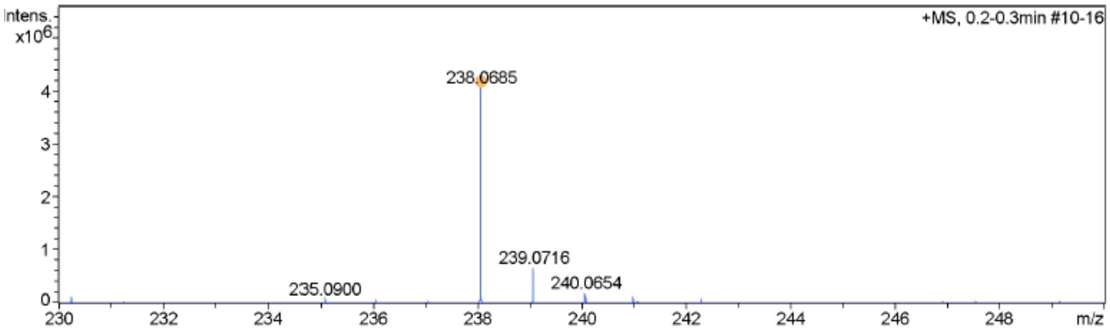

Meas. m/z	#	Ion Formula	m/z	err [ppm]	mSigma	# mSigma	Score	rdb	e⁻ Conf	N-Rule	Adduct
238.0685	1	C15H12NS	238.0685	-0.1	9.0	1	100.00	11.0	even	ok	M+H

Figure SI_81: HRMS (ESI^+, m/z) analysis of **3r**.

3-Phenylpropanethioamide (3s)

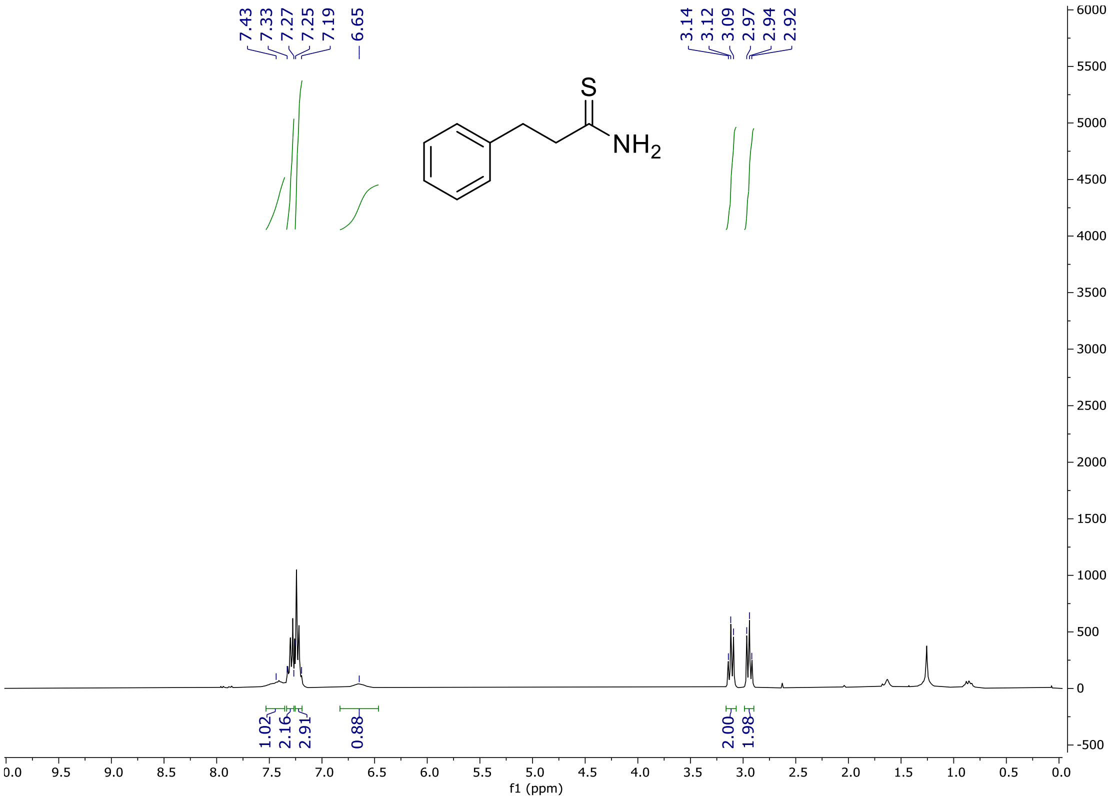

Figure SI_82: ^{1}H-NMR for **3s** in $CDCl_3$ (300 MHz).

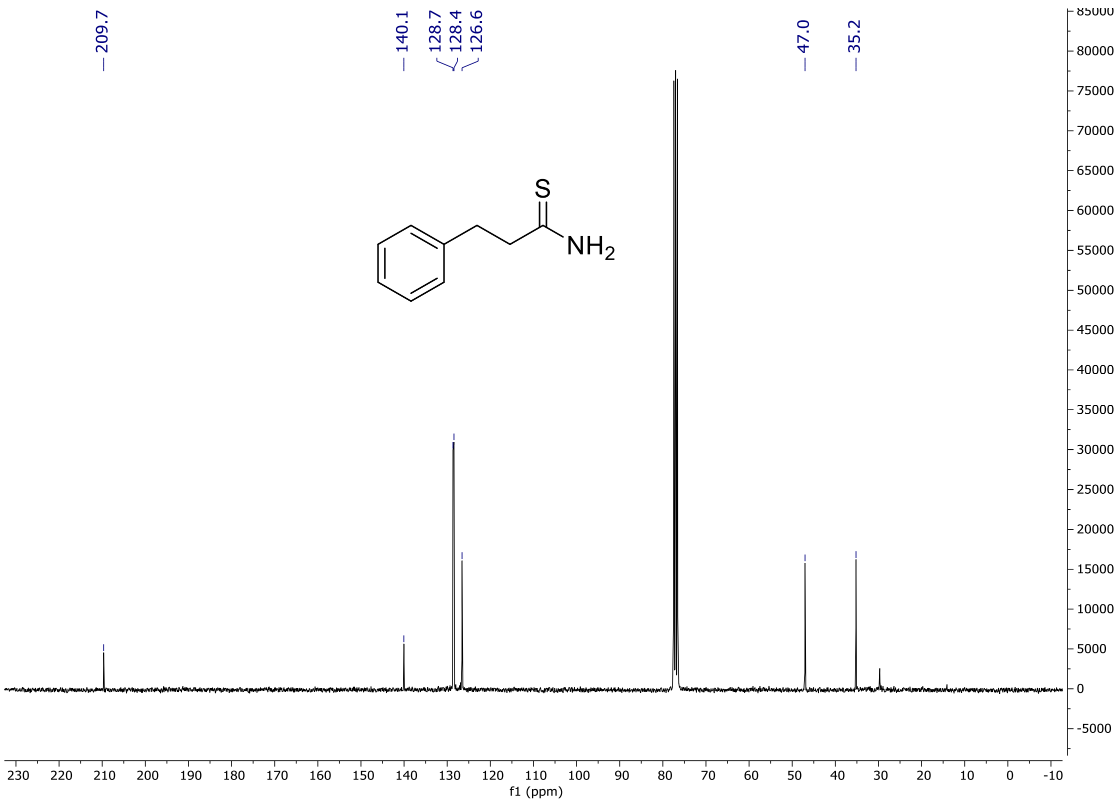

Figure SI_83: ^{13}C-NMR for **3s** in $CDCl_3$ (75 MHz).

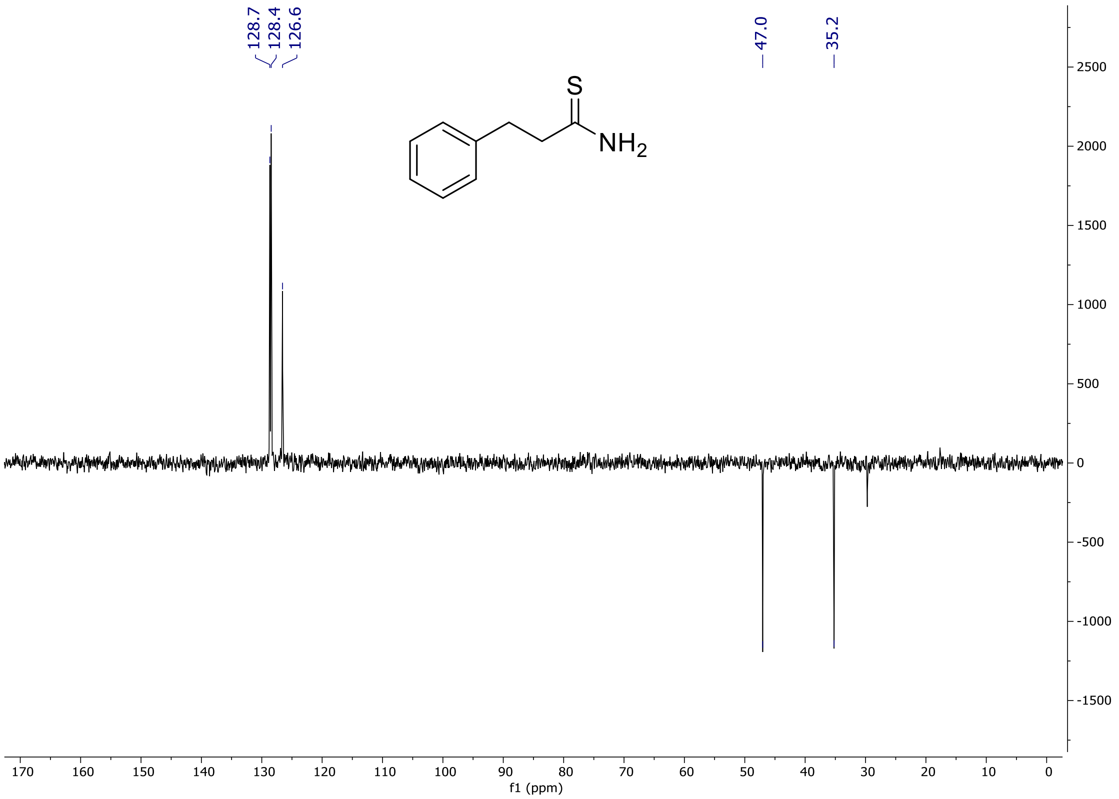

Figure SI_84: DEPT 135-NMR for **3s** in $CDCl_3$ (75 MHz).

Cyclohexanecarbothioamide (3t)

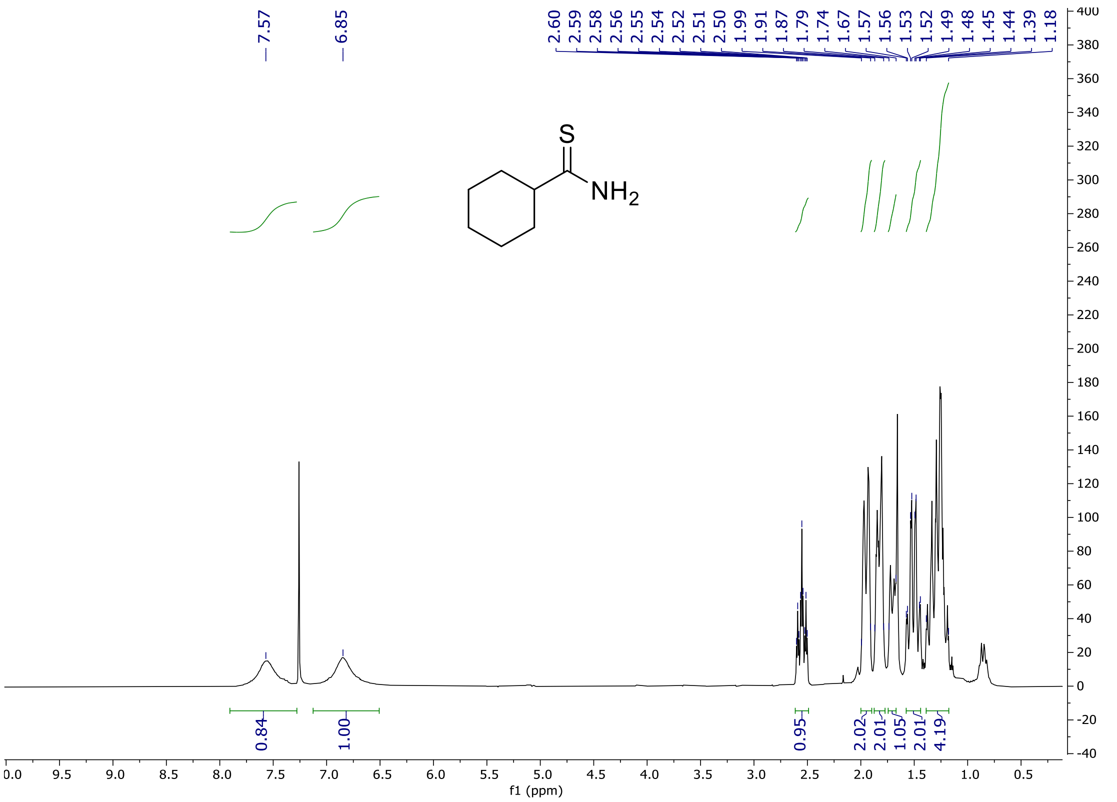

Figure SI_85: ^{1}H-NMR for **3t** in $CDCl_3$ (300 MHz).

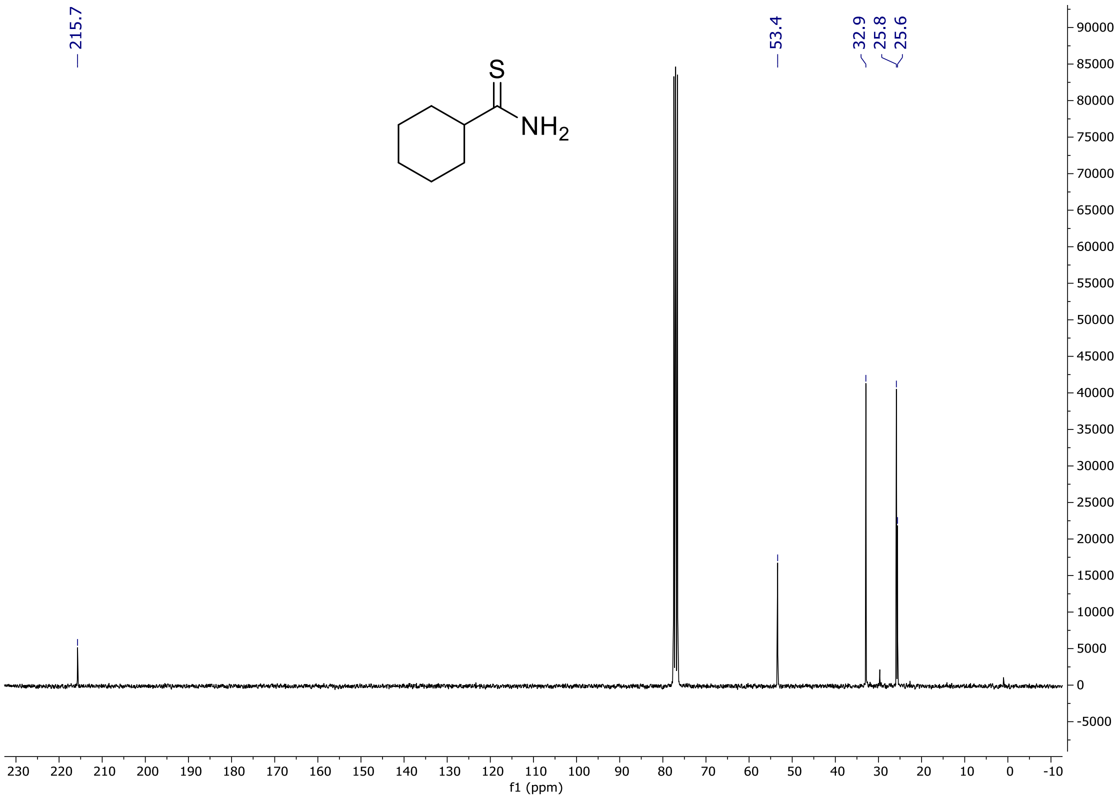

Figure SI_86: ^{13}C-NMR for **3t** in $CDCl_3$ (75 MHz).

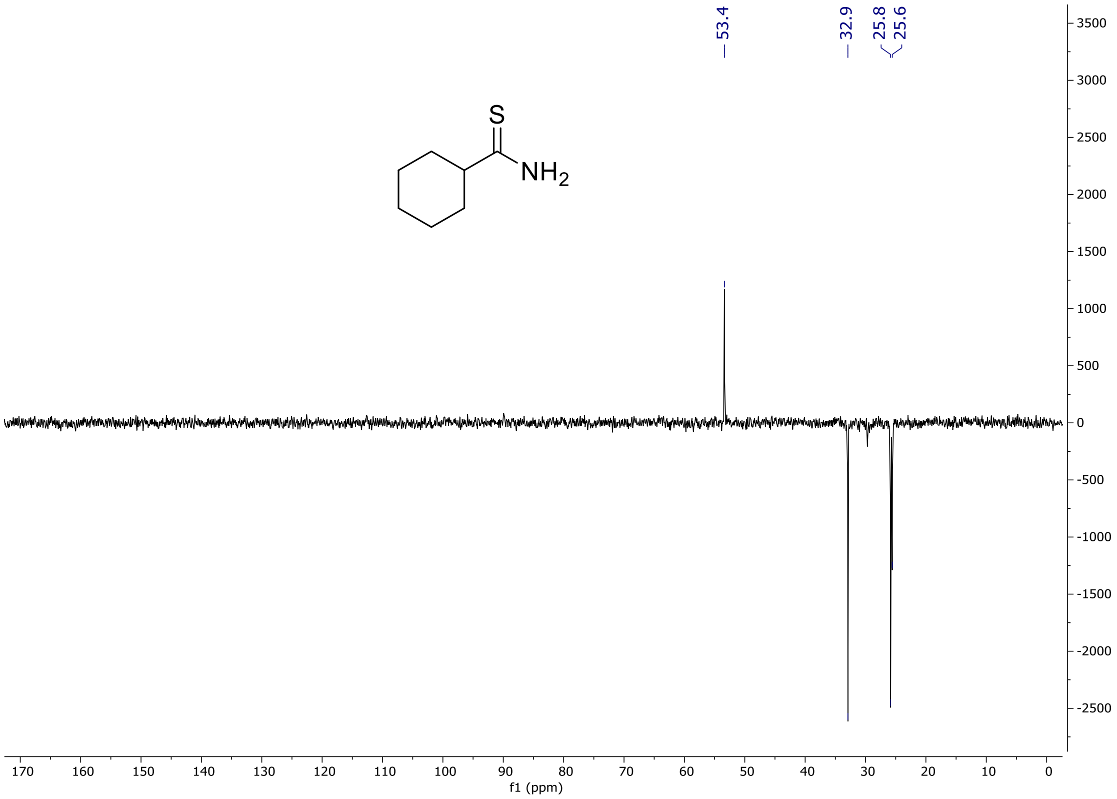

Figure SI_87: DEPT 135-NMR for **3t** in $CDCl_3$ (75 MHz).

4-aminobenzonitrile (3u)

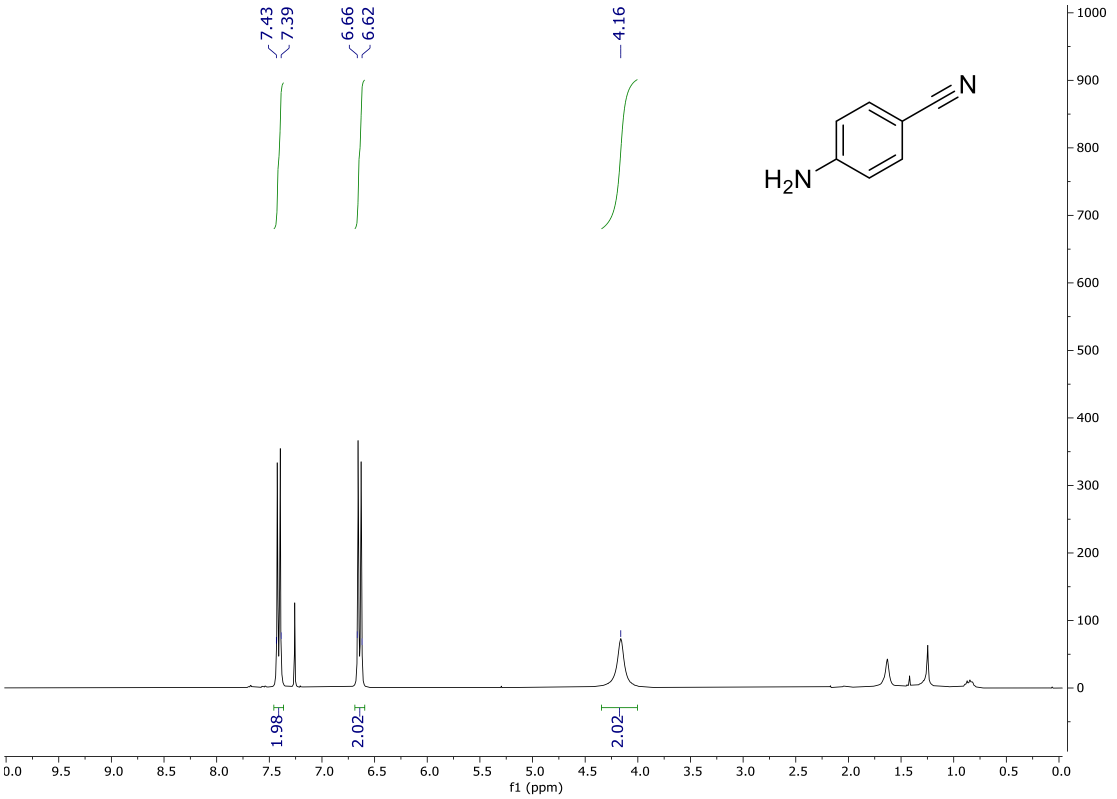

Figure SI_88: ^{1}H-NMR for **3u** in $CDCl_3$ (300 MHz).

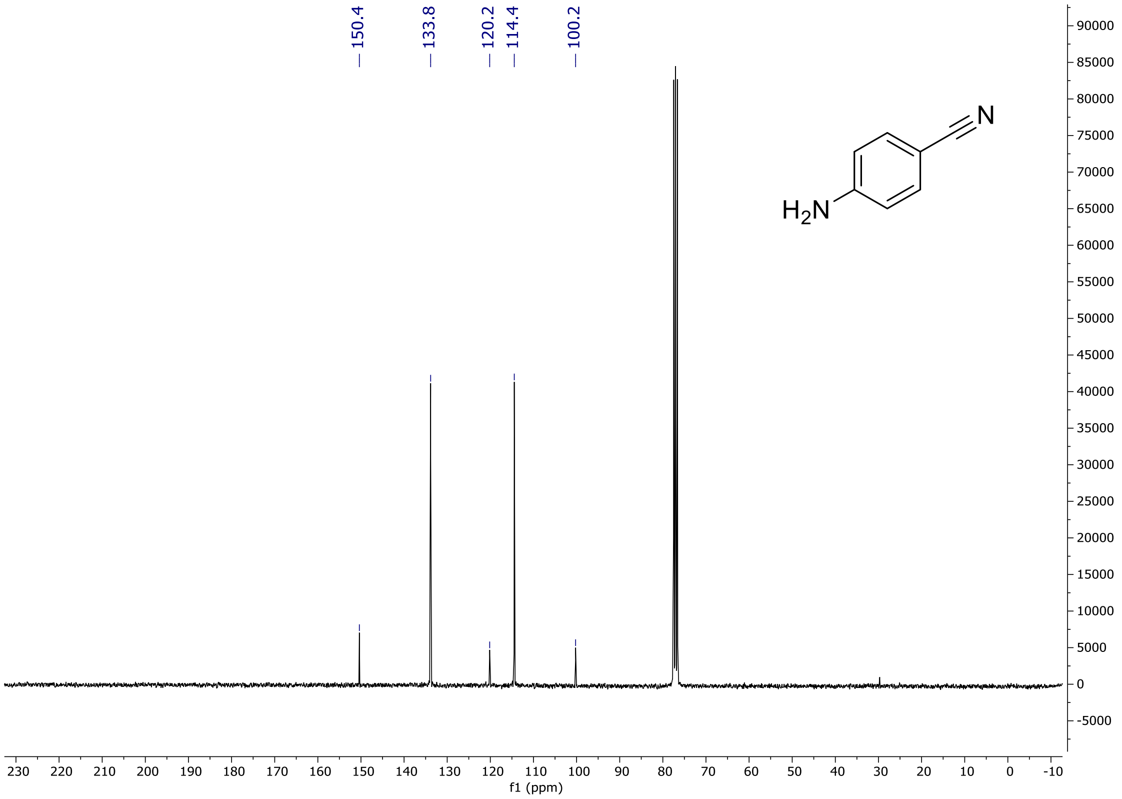

Figure SI_89: ^{13}C-NMR for **3u** in $CDCl_3$ (75 MHz).

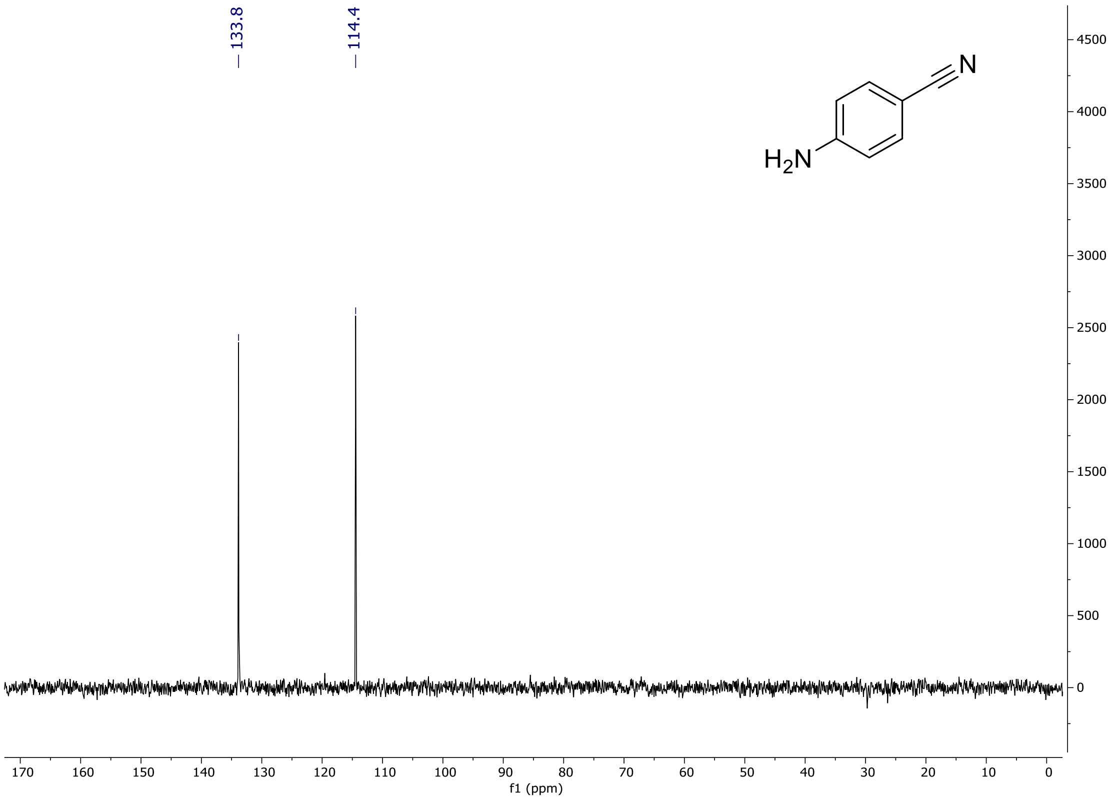

Figure SI_90: DEPT 135-NMR for **3u** in $CDCl_3$ (75 MHz).

Ethyl 4-methyl-2-(pyridin-3-yl)thiazole-5-carboxylate 4

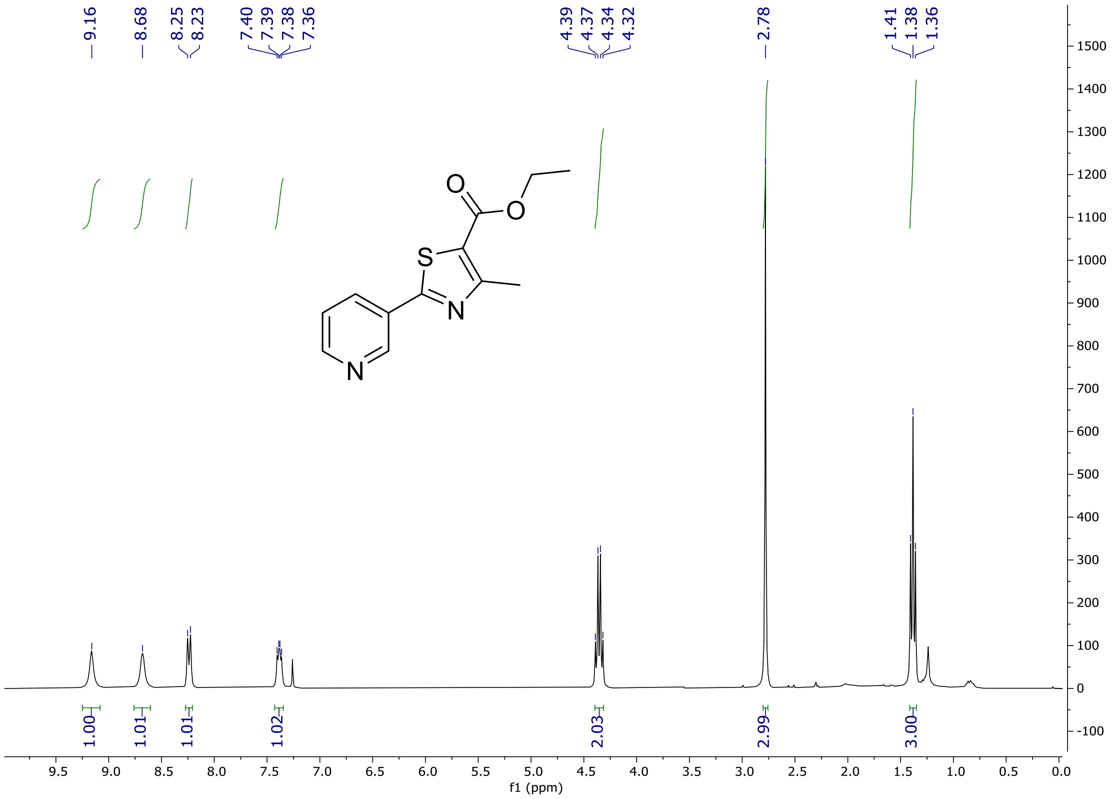

Figure SI_91: ^{1}H-NMR for **4** in $CDCl_3$ (300 MHz).

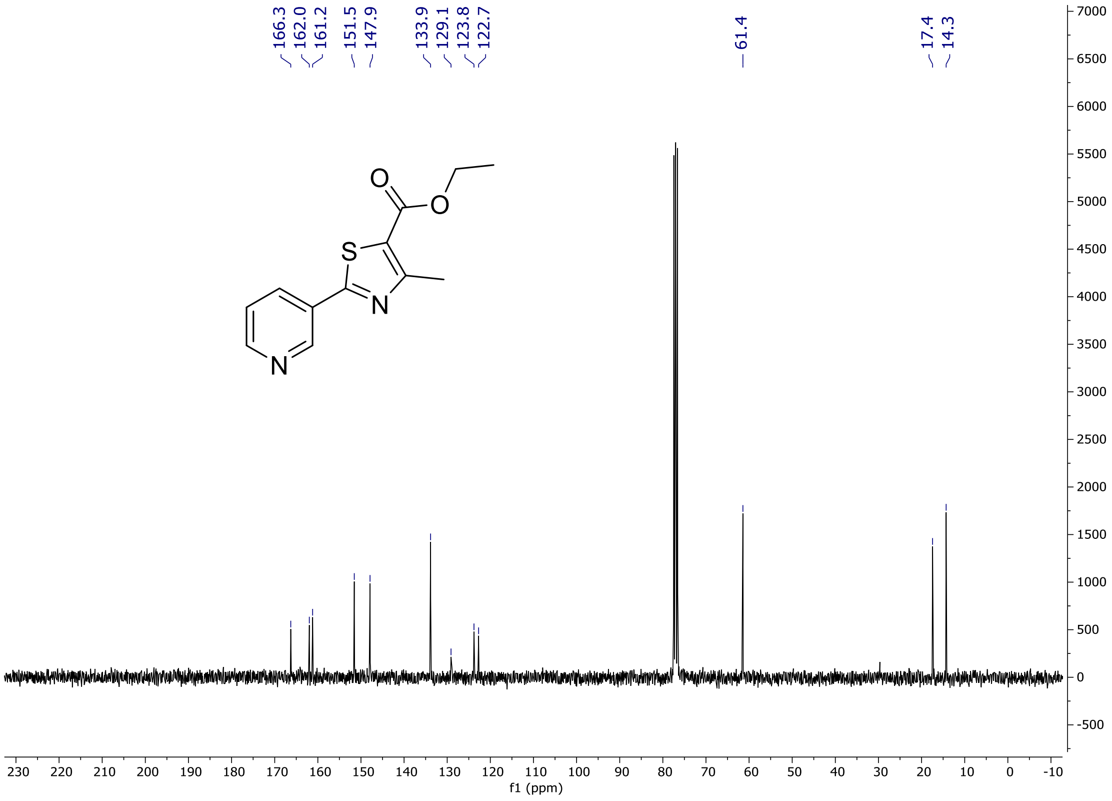

Figure SI_92: ^{13}C-NMR for **4** in $CDCl_3$ (75 MHz).

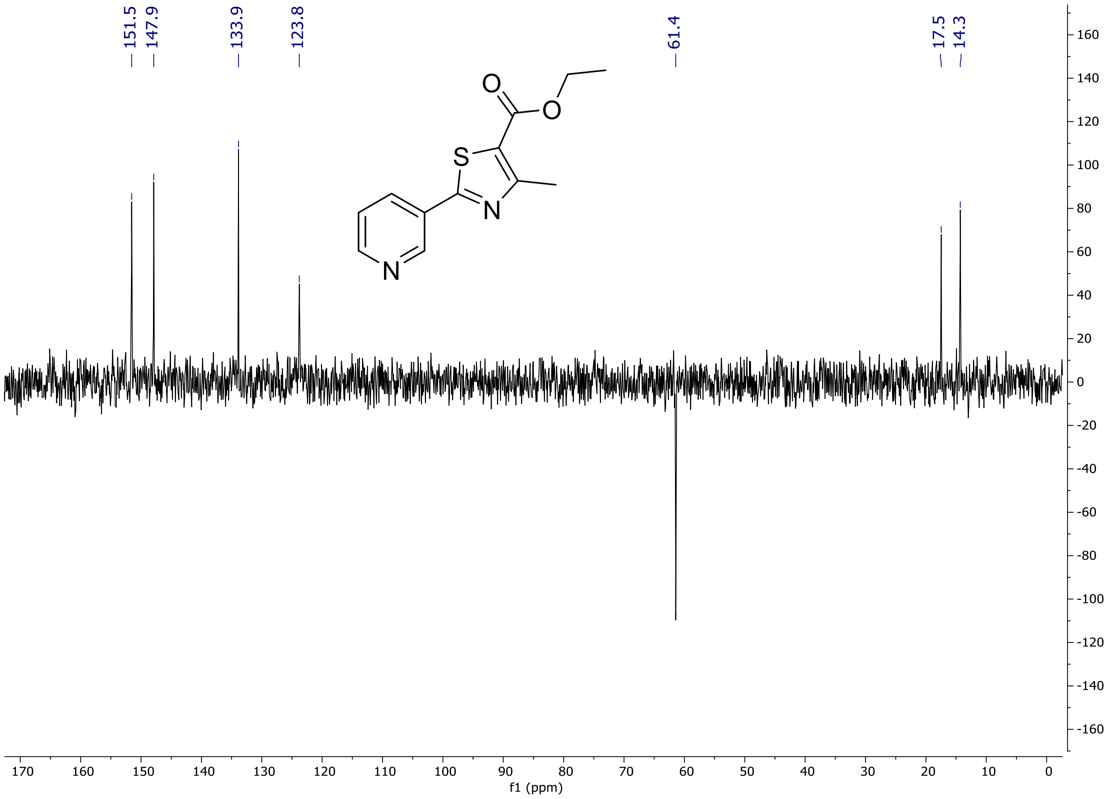

Figure SI_93: DEPT 135-NMR for **4** in $CDCl_3$ (75 MHz).

3-Bromo-4-hydroxybenzaldehyde

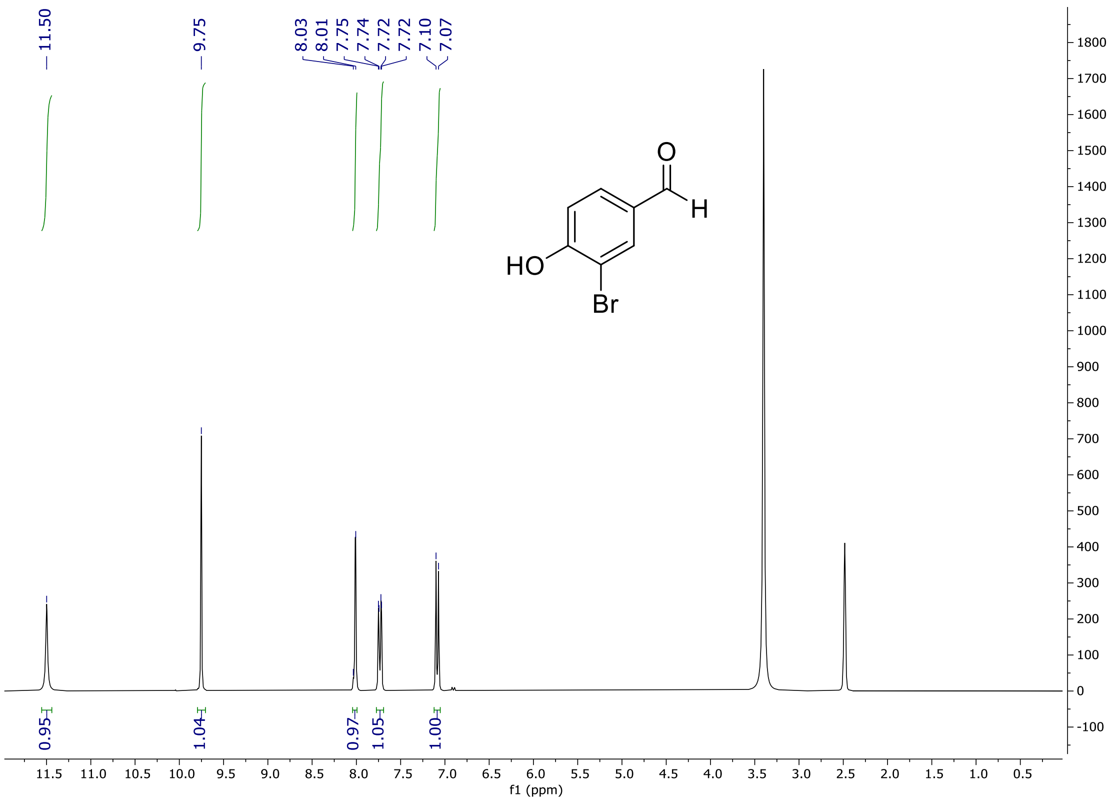

Figure SI_94: ^{1}H-NMR for 3-bromo-4-hydroxybenzaldehyde in DMSO-d^6 (300 MHz).

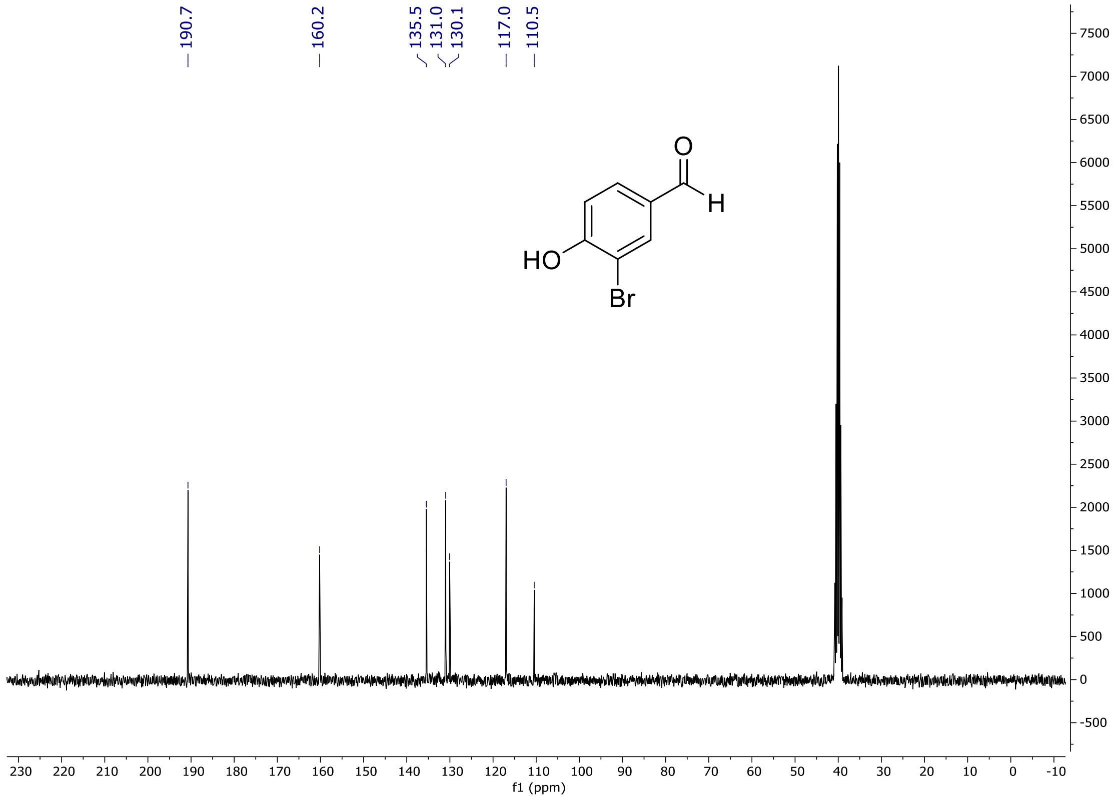

Figure SI_95: ^{13}C-NMR for 3-bromo-4-hydroxybenzaldehyde in DMSO-d^6 (75 MHz).

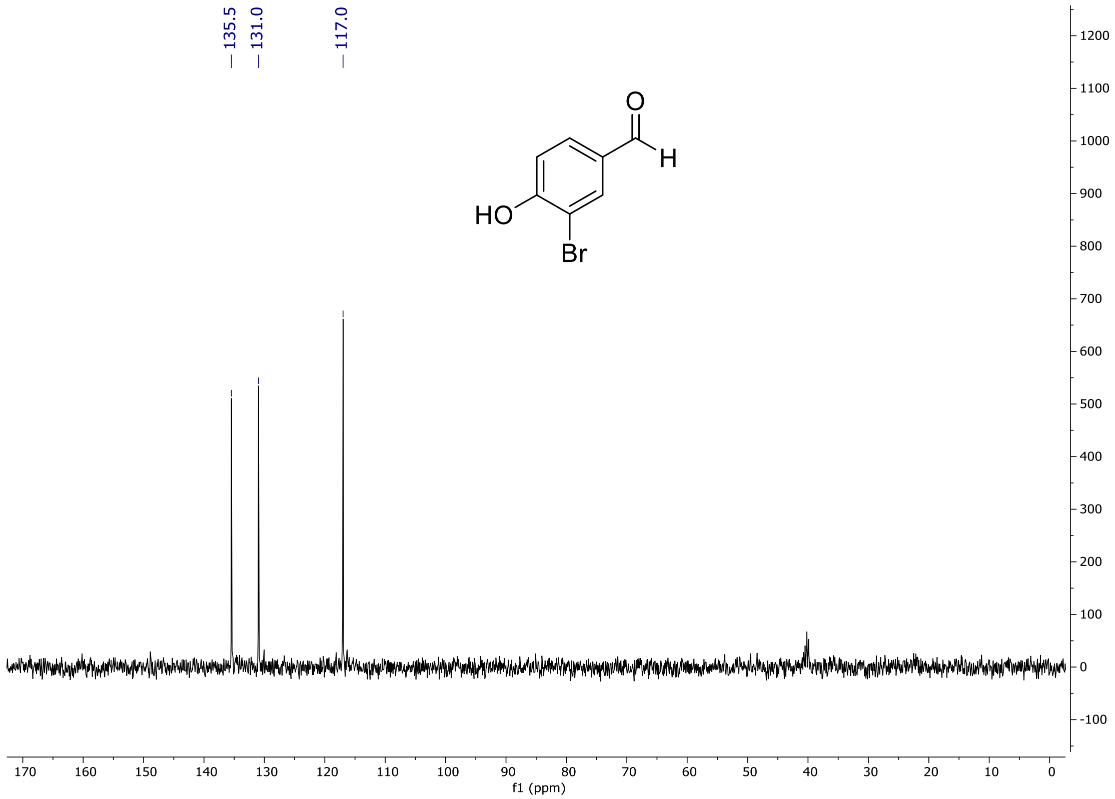

Figure SI_96: DEPT 135-NMR for 3-bromo-4-hydroxybenzaldehyde in DMSO-d^6 (75 MHz).

3-Bromo-4-isobutoxybenzaldehyde

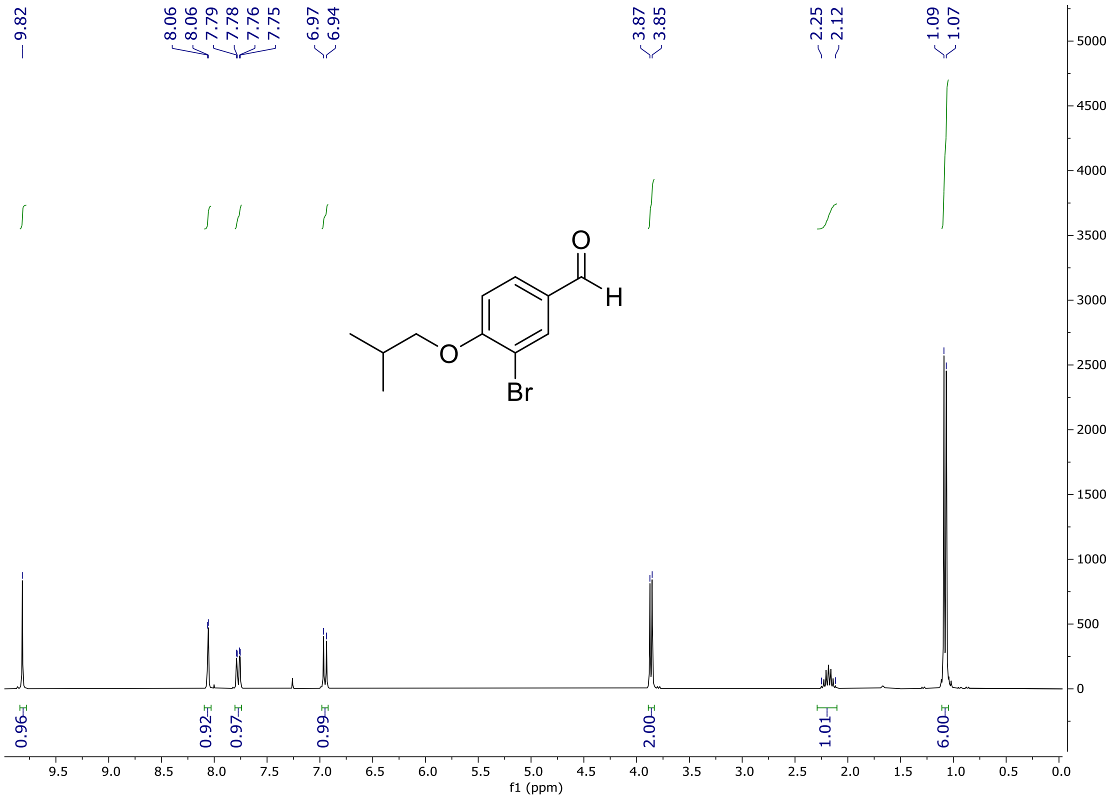

Figure SI_97: ^{1}H-NMR for 3-bromo-4-isobutoxybenzaldehyde in $CDCl_3$ (300 MHz).

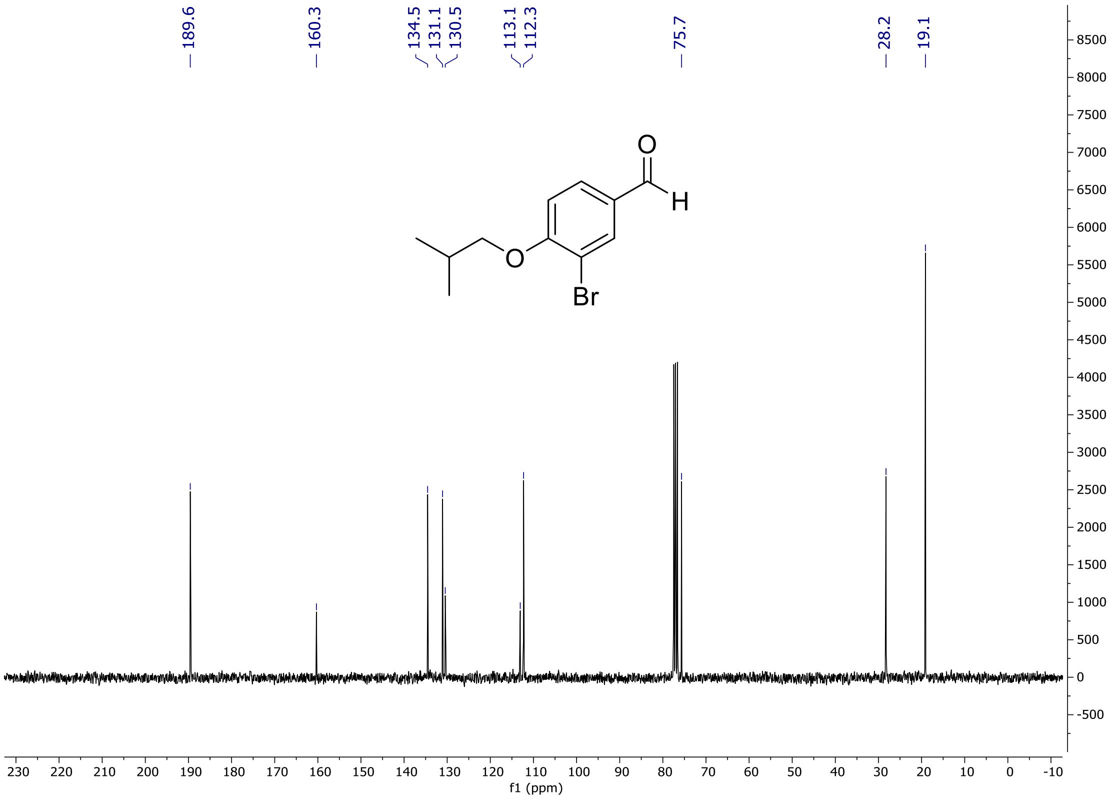

Figure SI_98: ^{13}C-NMR for 3-bromo-4-isobutoxybenzaldehyde in $CDCl_3$ (75 MHz).

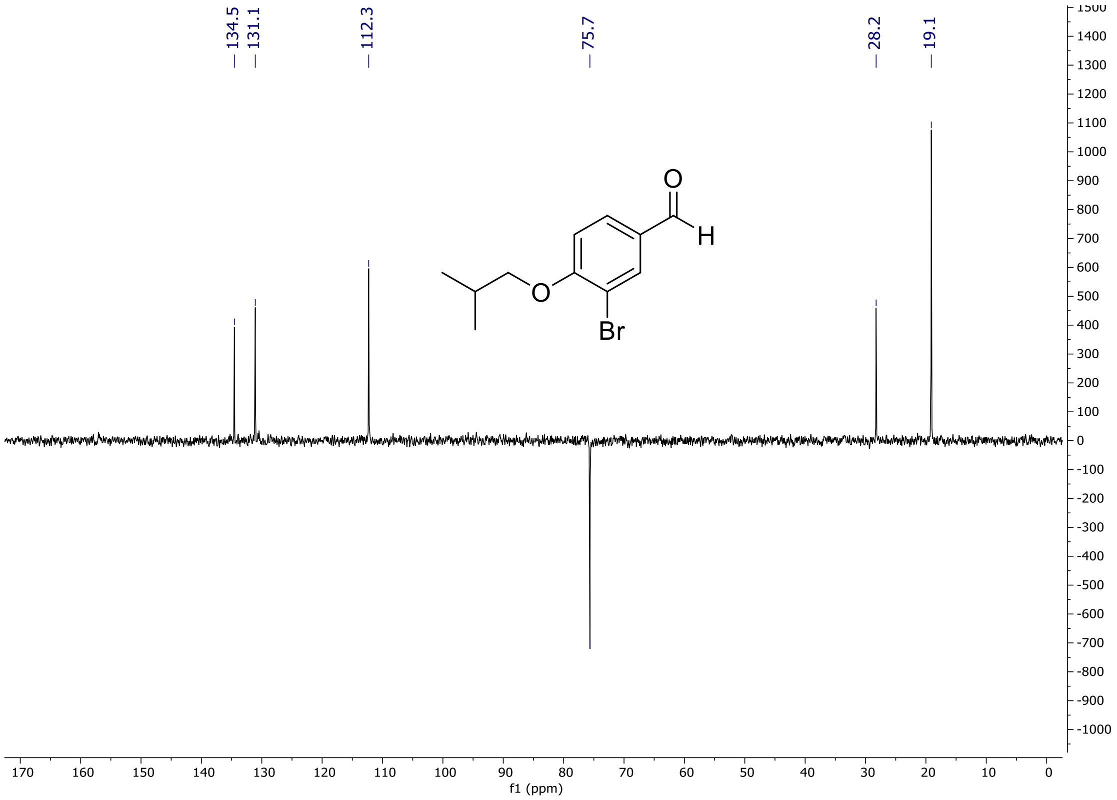

Figure SI_99: DEPT 135-NMR for 3-bromo-4-isobutoxybenzaldehyde in $CDCl_3$ (75 MHz).

3-Cyano-4-isobutoxybenzaldehyde (6)

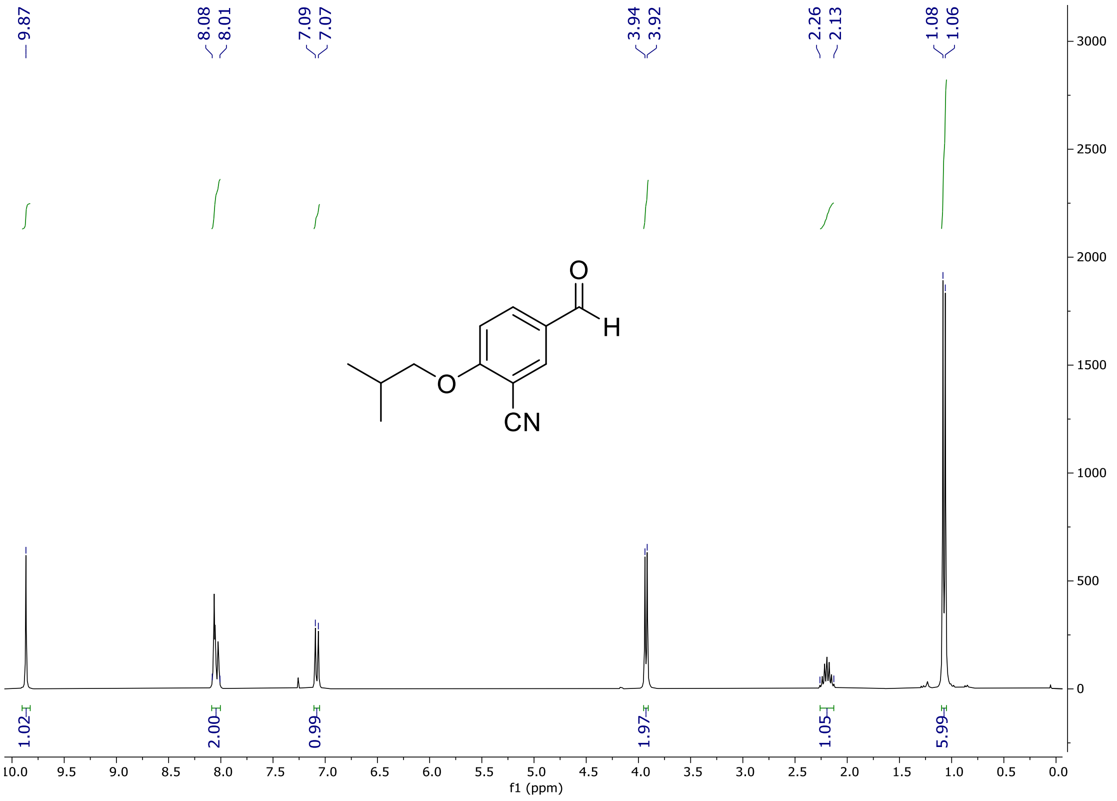

Figure SI_100: ^{1}H-NMR for **6** in $CDCl_3$ (300 MHz).

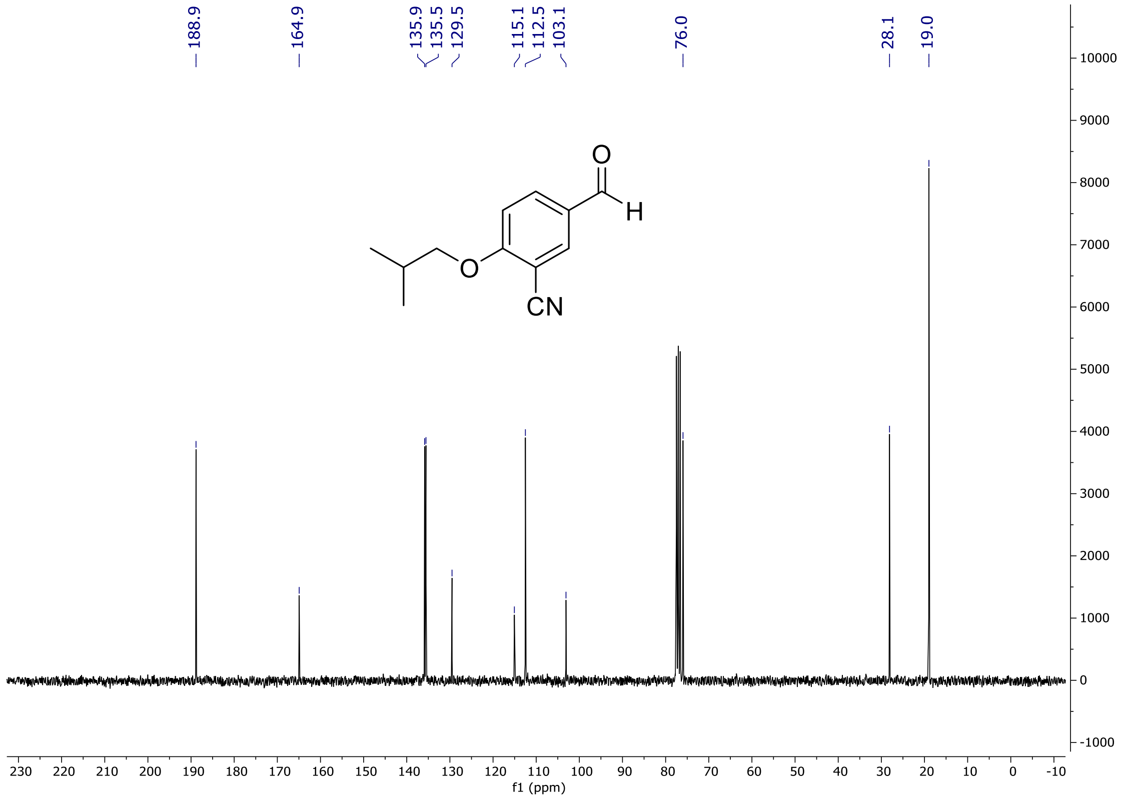

Figure SI_101: ^{13}C-NMR for **6** in $CDCl_3$ (75 MHz).

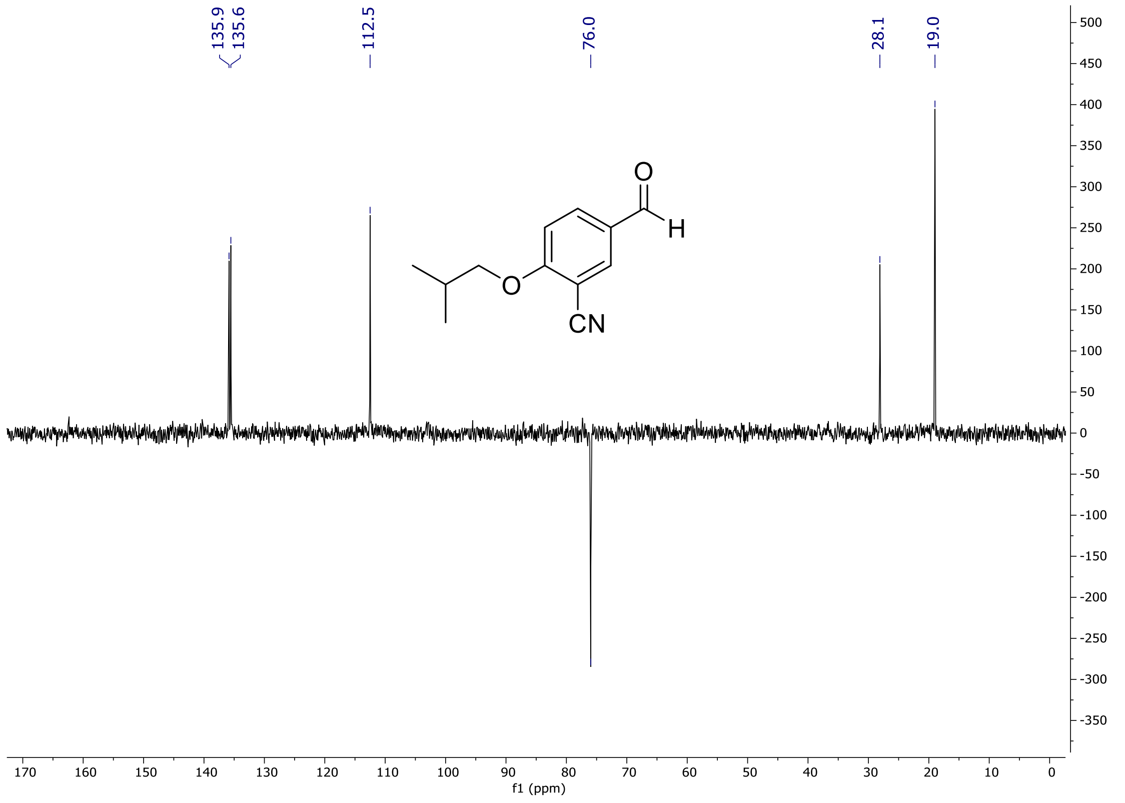

Figure SI_102: DEPT 135-NMR for **6** in $CDCl_3$ (75 MHz).

3-Cyano-4-isobutoxybenzaldoxime (1v)

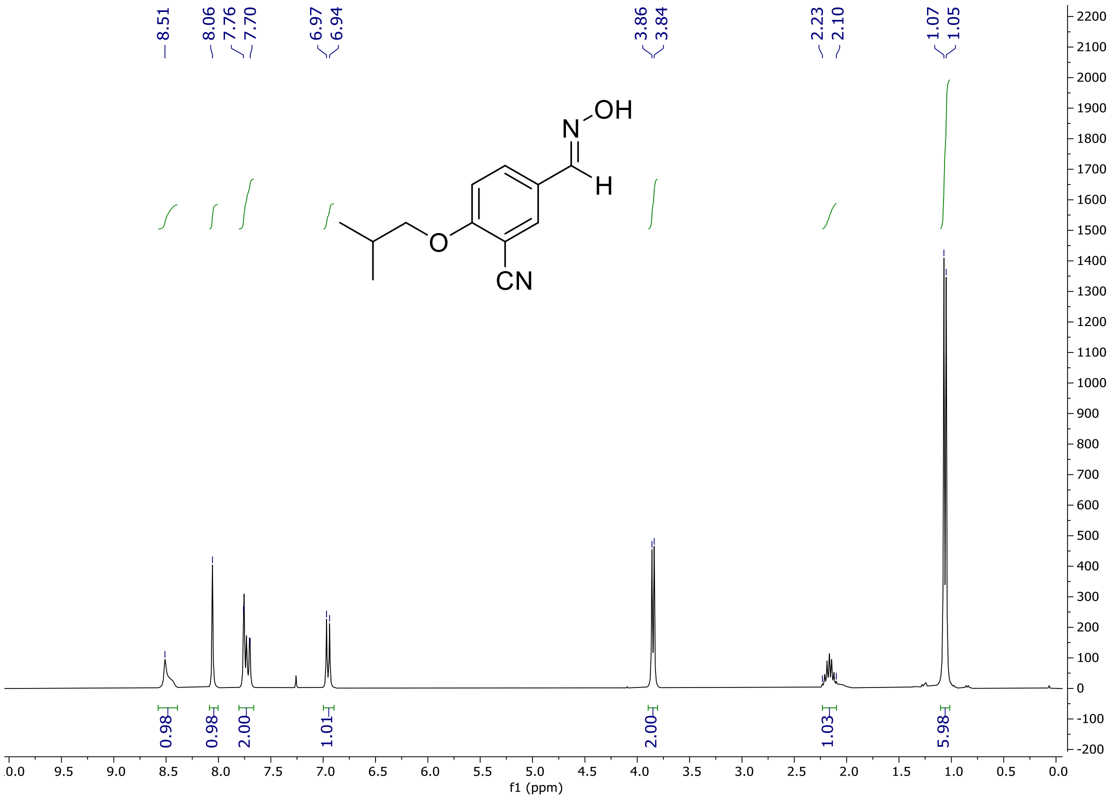

Figure SI_103: ^{1}H-NMR for **1v** in $CDCl_3$ (300 MHz).

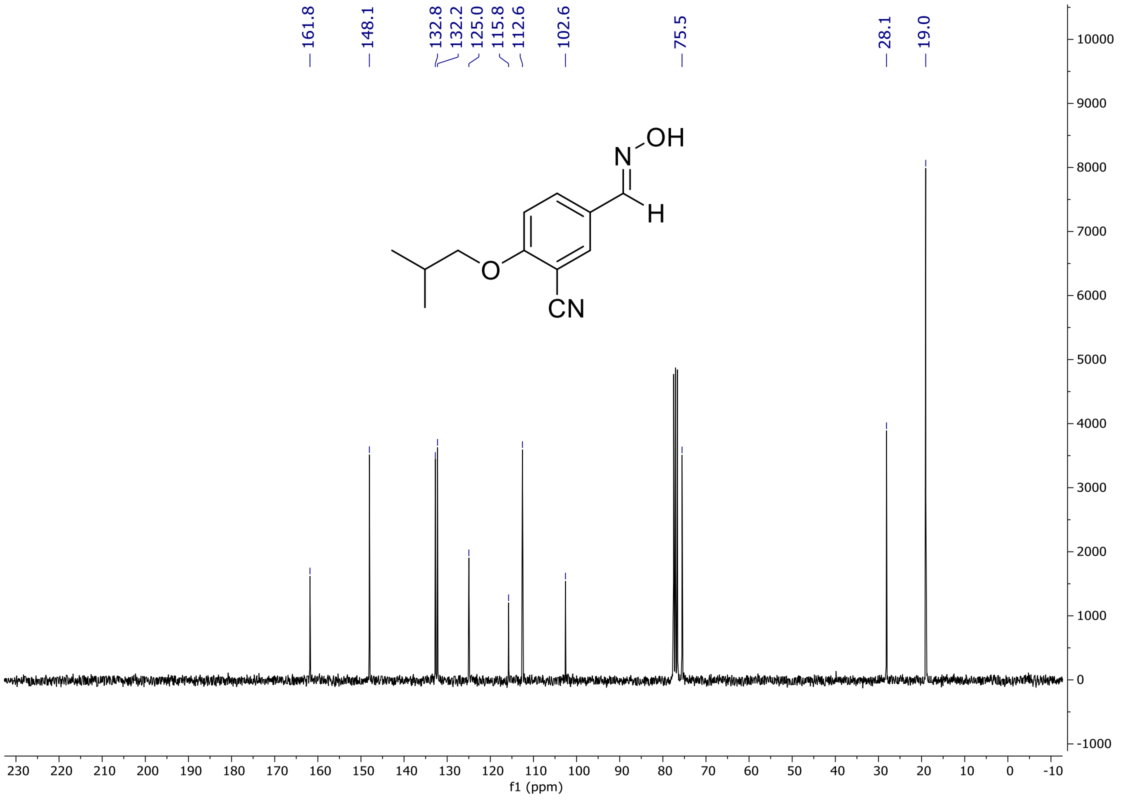

Figure SI_104: ^{13}C-NMR for **1v** in $CDCl_3$ (75 MHz).

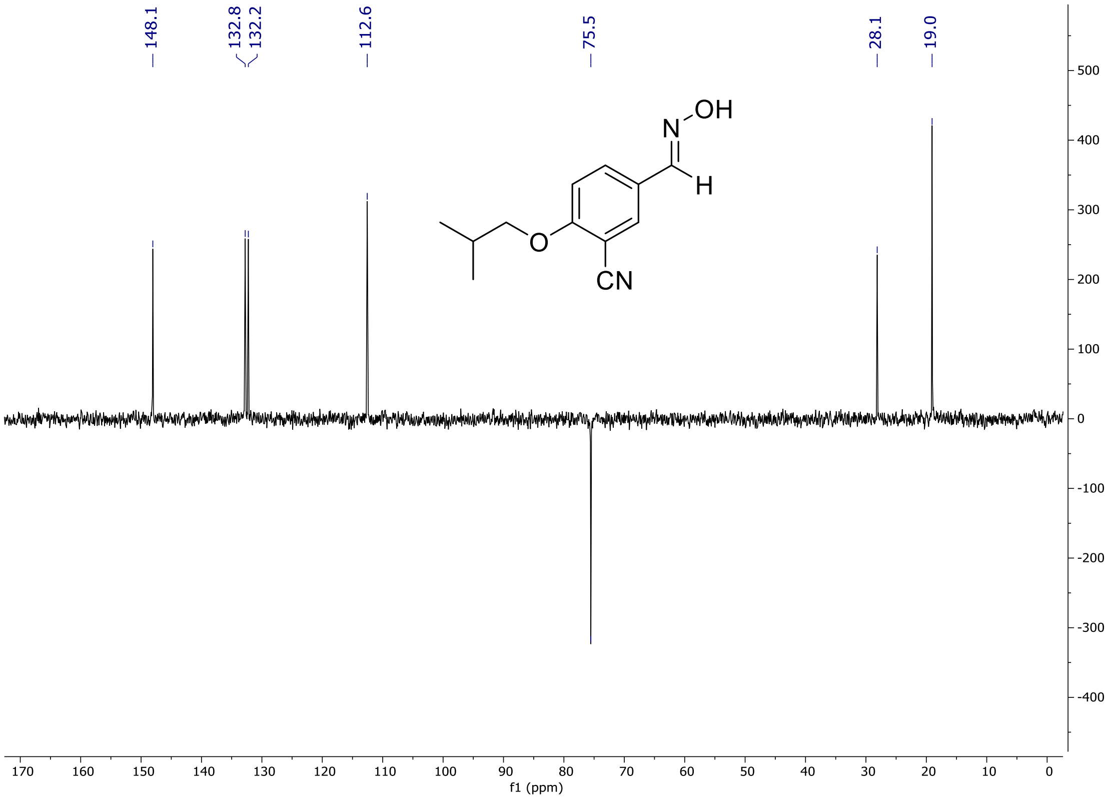

Figure SI_105: DEPT 135-NMR for **1v** in $CDCl_3$ (75 MHz).

Acquisition Parameter

Source Type	ESI	Ion Polarity	Positive	Set Nebulizer	2.0 Bar
Focus	Active	Set Capillary	3500 V	Set Dry Heater	250 °C
Scan Begin	50 m/z	Set End Plate Offset	-500 V	Set Dry Gas	5.0 l/min
Scan End	1500 m/z	Set Charging Voltage	2000 V	Set Divert Valve	Source
		Set Corona	0 nA	Set APCI Heater	0 °C

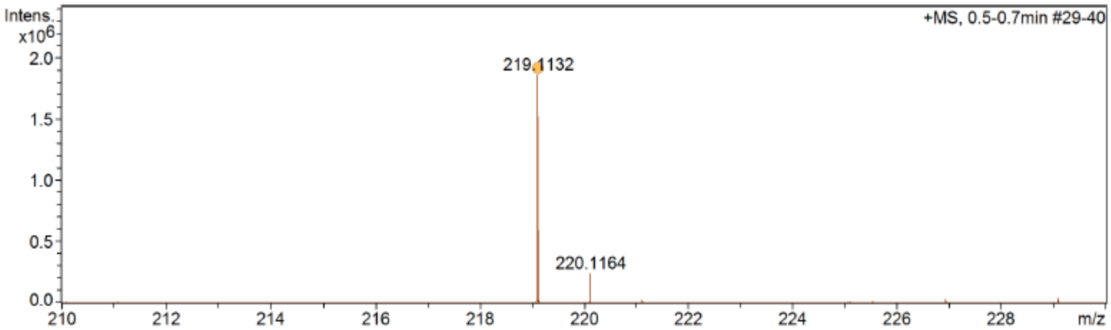

Meas. m/z	#	Ion Formula	m/z	err [ppm]	mSigma	# mSigma	Score	rdb	e⁻ Conf	N-Rule	Adduct
219.1132	1	C12H15N2O2	219.1128	-1.9	5.2	1	100.00	7.0	even	ok	M+H

Figure SI_106: HRMS (ESI^+, m/z) analysis of **1v**.

3-Cyano-4-isobutoxybenzothioamide (3v)

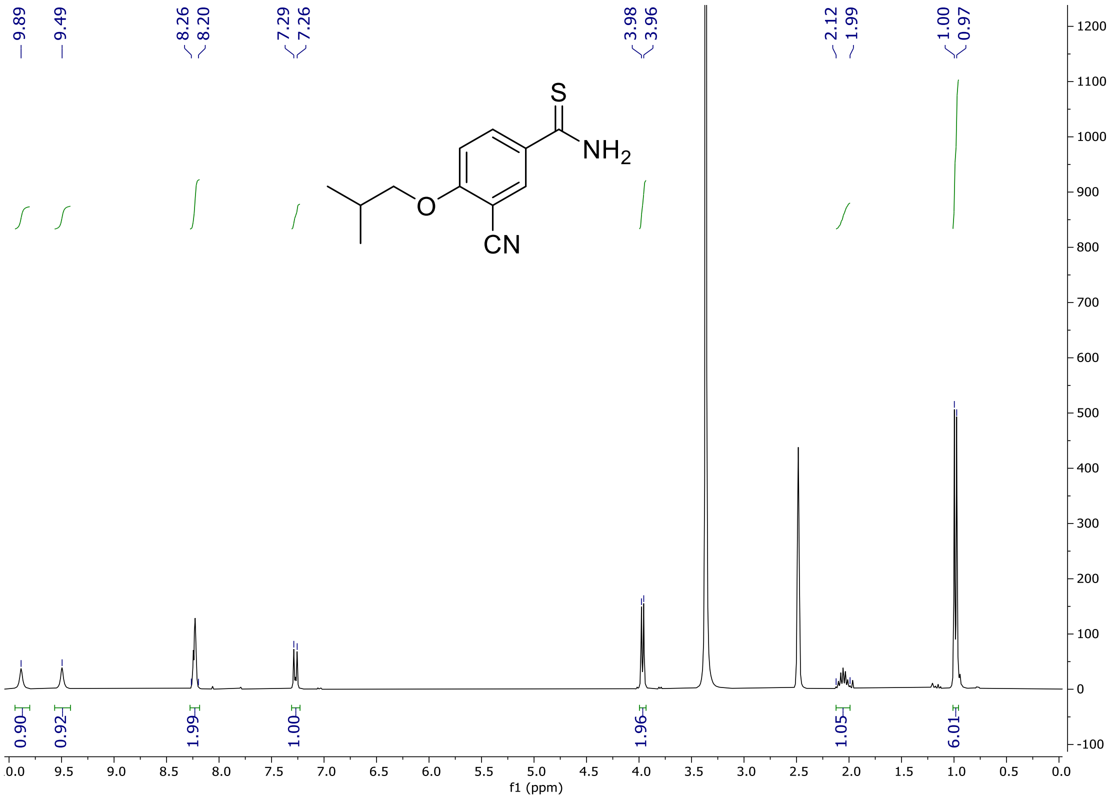

Figure SI_107: ^{1}H-NMR for **3v** in DMSO-d^6 (300 MHz).

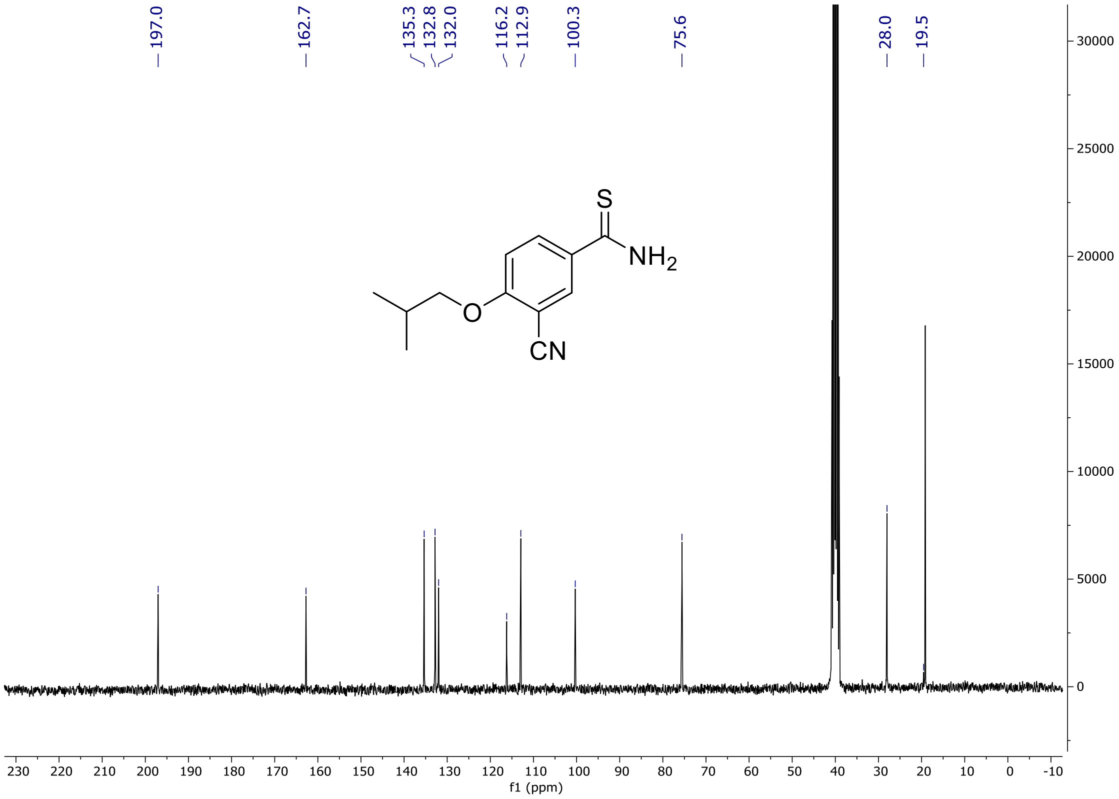

Figure SI_108: ^{13}C-NMR for **3v** in DMSO-d^6 (75 MHz).

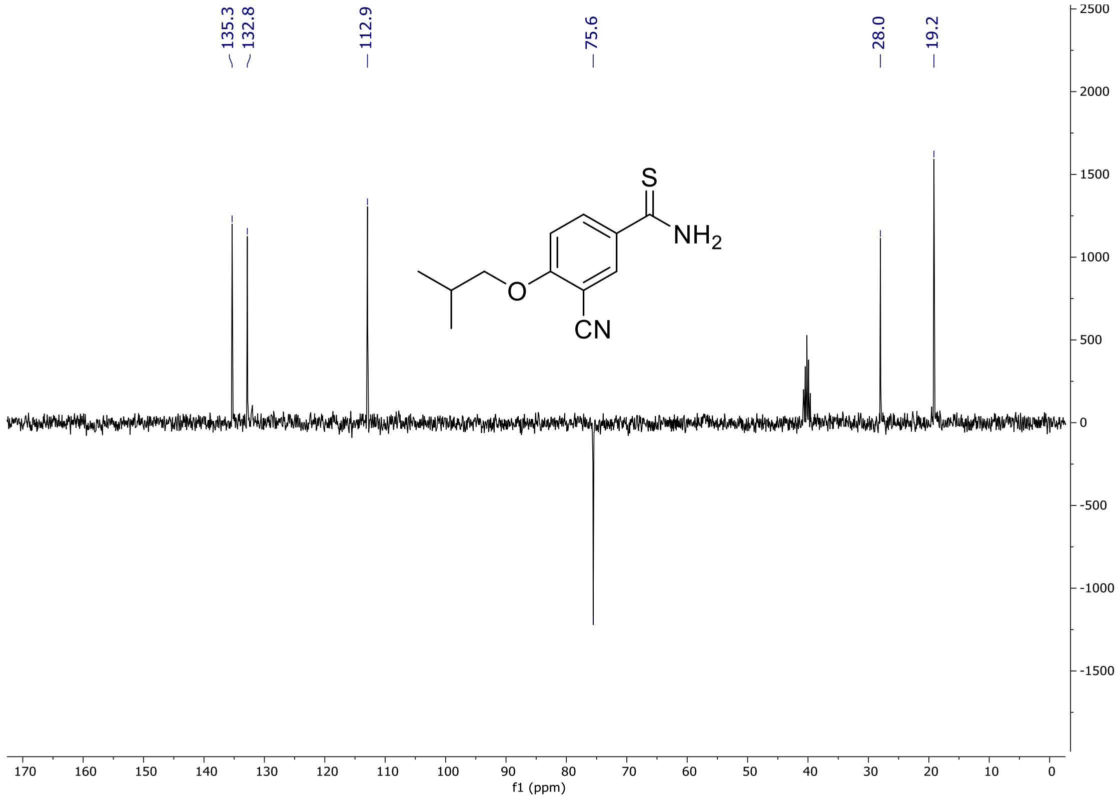

Figure SI_109: DEPT 135-NMR for **3v** in DMSO-d^6 (75 MHz).

Acquisition Parameter

Source Type	ESI	Ion Polarity	Positive	Set Nebulizer	2.0 Bar
Focus	Active	Set Capillary	4000 V	Set Dry Heater	250 °C
Scan Begin	50 m/z	Set End Plate Offset	-500 V	Set Dry Gas	5.0 l/min
Scan End	1500 m/z	Set Charging Voltage	2000 V	Set Divert Valve	Source
		Set Corona	0 nA	Set APCI Heater	0 °C

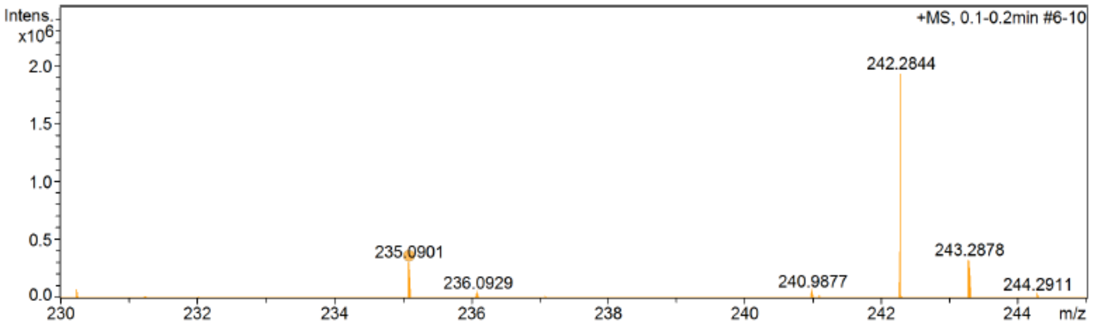

Meas. m/z	#	Ion Formula	m/z	err [ppm]	mSigma	# mSigma	Score	rdb	e¯ Conf	N-Rule	Adduct	Adduct
235.0901	1	C12H15N2OS	235.0900	-0.7	11.8	1	100.00	7.0	even	ok	M+H	M+H

Figure SI_110: HRMS (ESI$^+$, m/z) analysis of **3v**.

Ethyl 2-(3-cyano-4-isobutoxyphenyl)-4-methylthiazole-5-carboxylate (7)

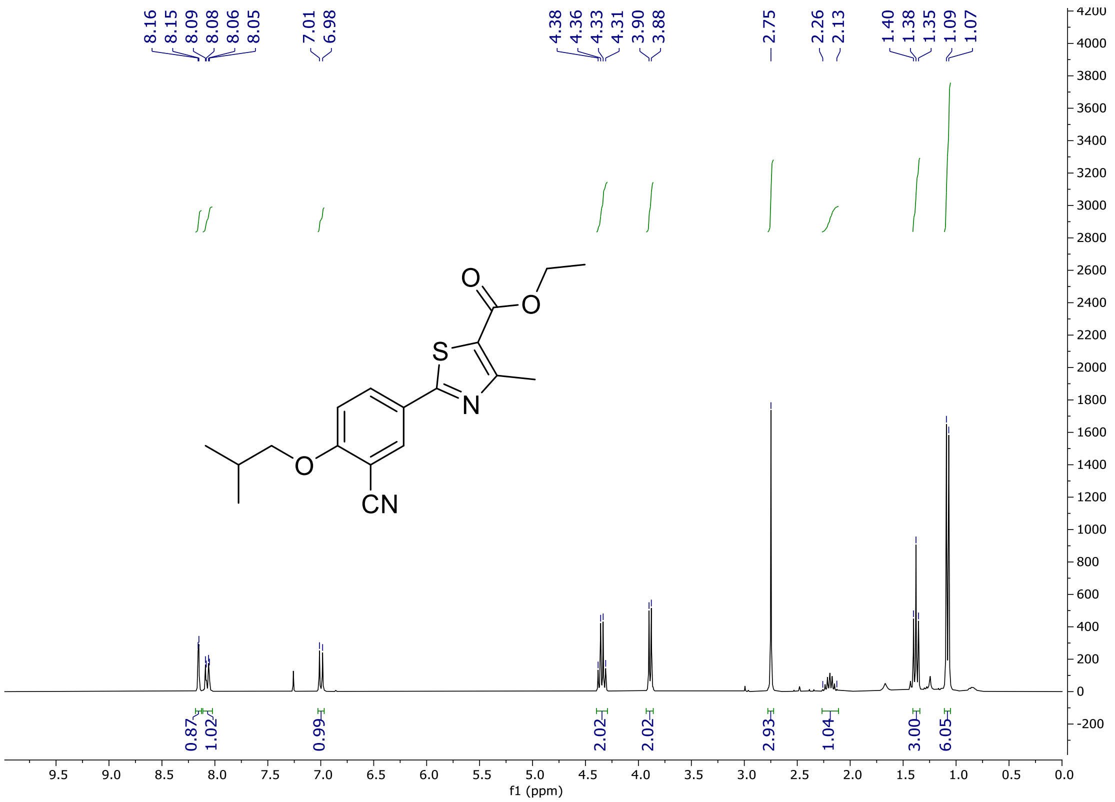

Figure SI_111: ^{1}H-NMR for **7** in $CDCl_3$ (300 MHz).

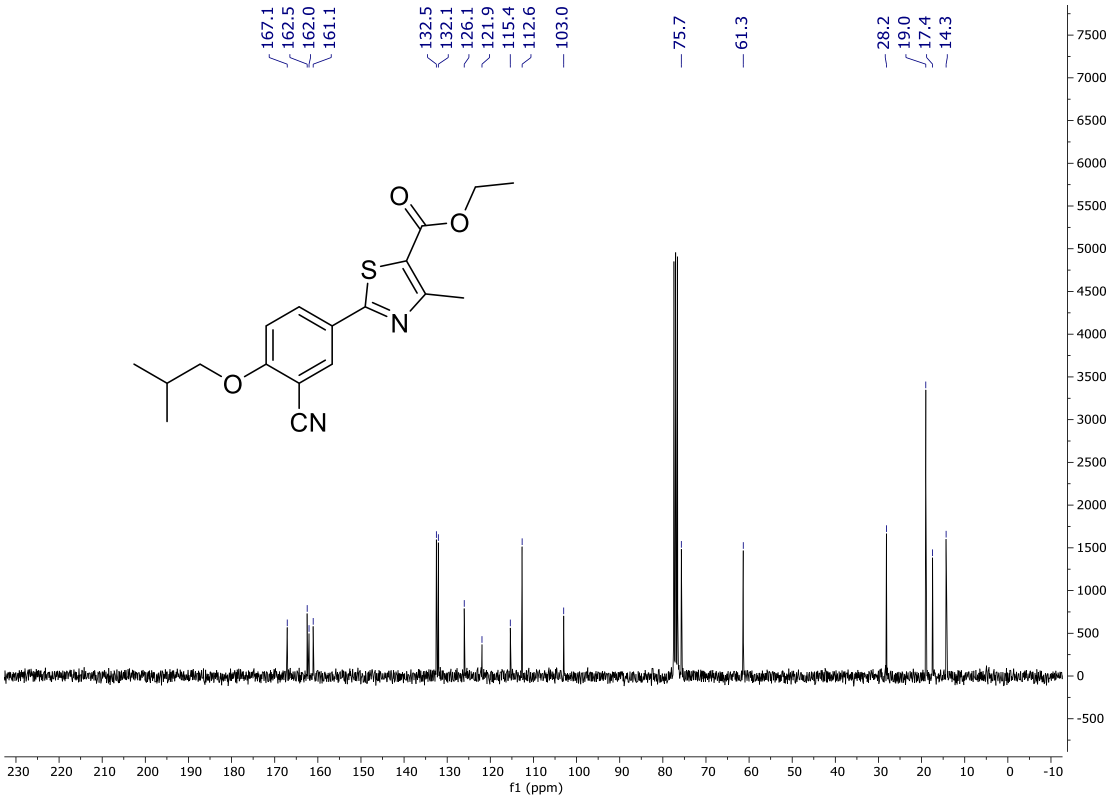

Figure SI_112: ^{13}C-NMR for **7** in $CDCl_3$ (75 MHz).

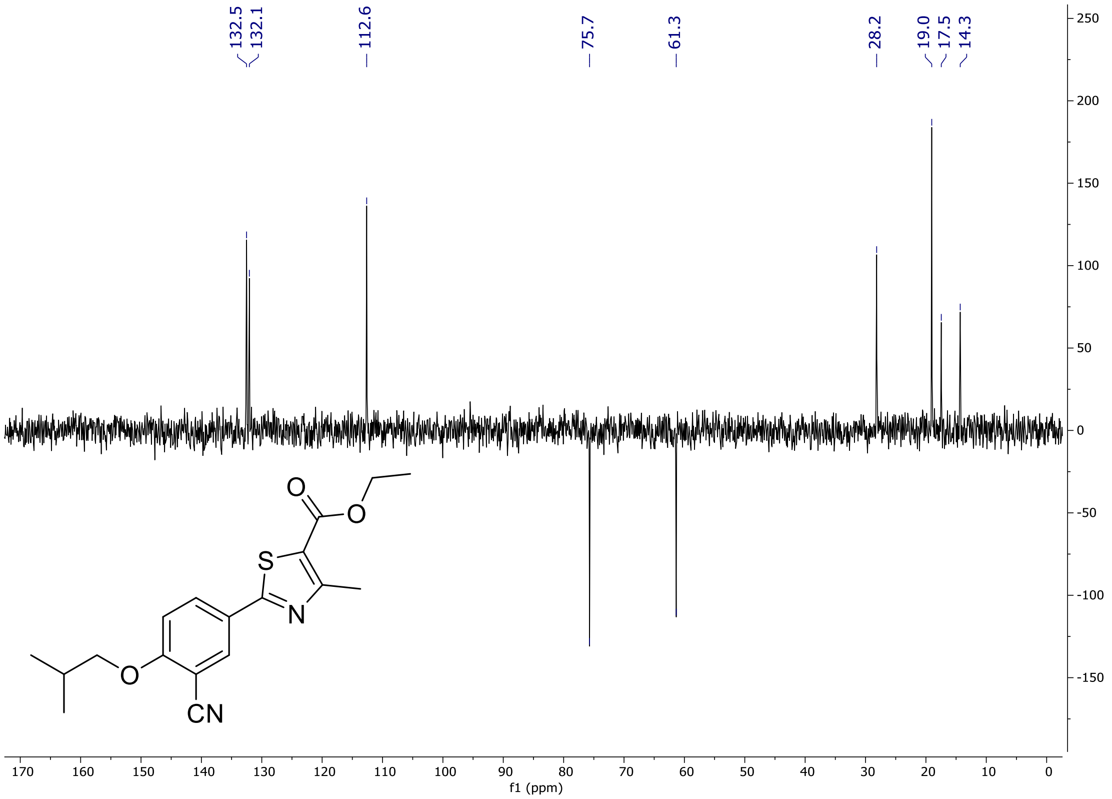

Figure SI_113: DEPT 135-NMR for **7** in $CDCl_3$ (75 MHz).

Febuxostat (8)

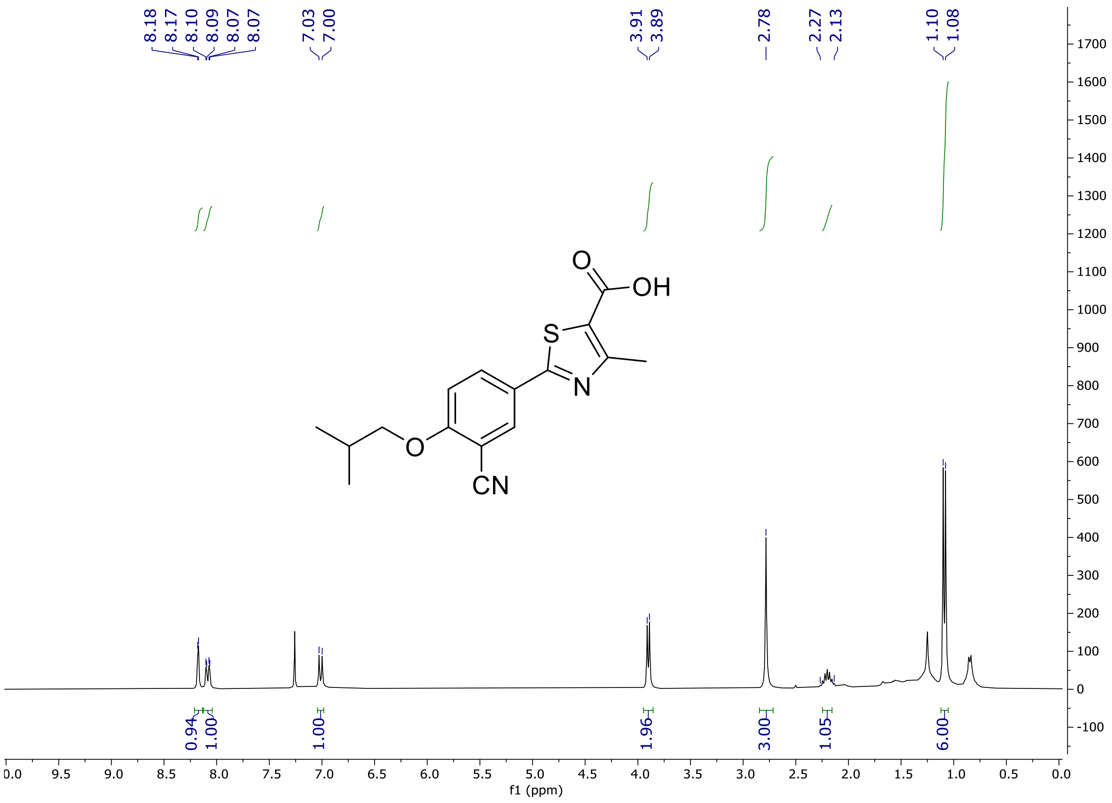

Figure SI_114: ^{1}H-NMR for **8** in $CDCl_3$ (300 MHz).

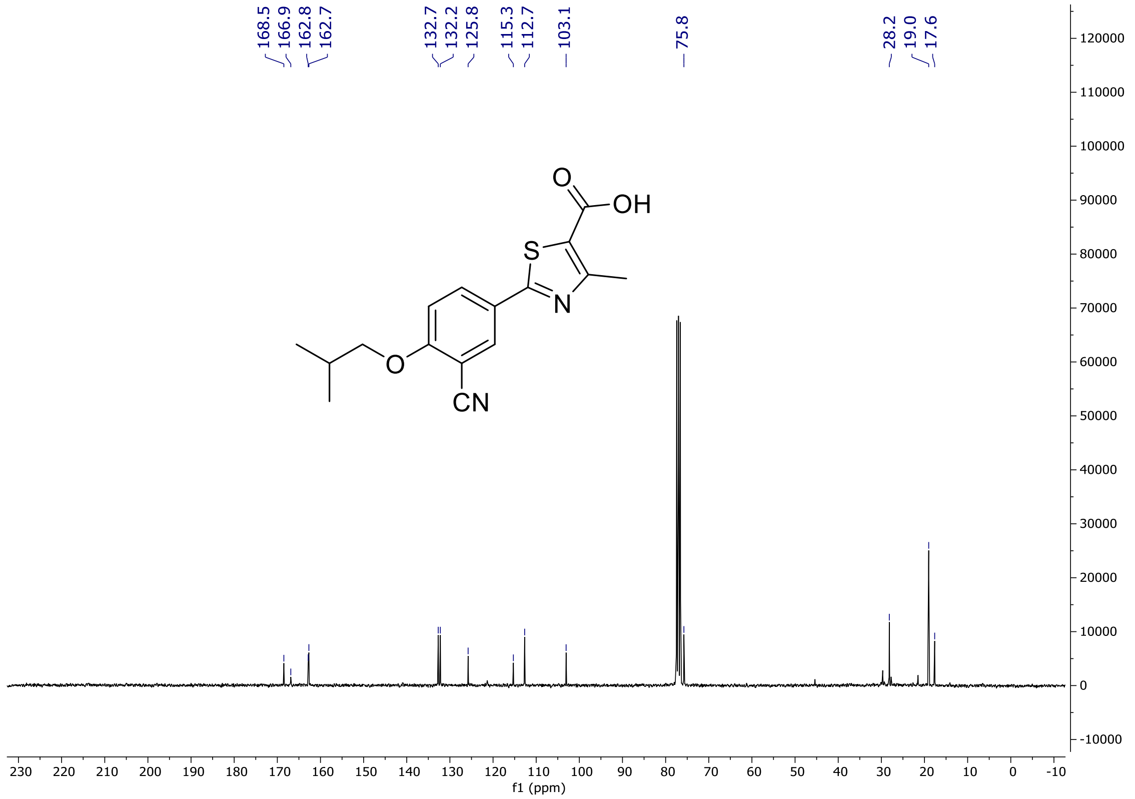

Figure SI_115: ^{13}C-NMR for **8** in $CDCl_3$ (75 MHz).

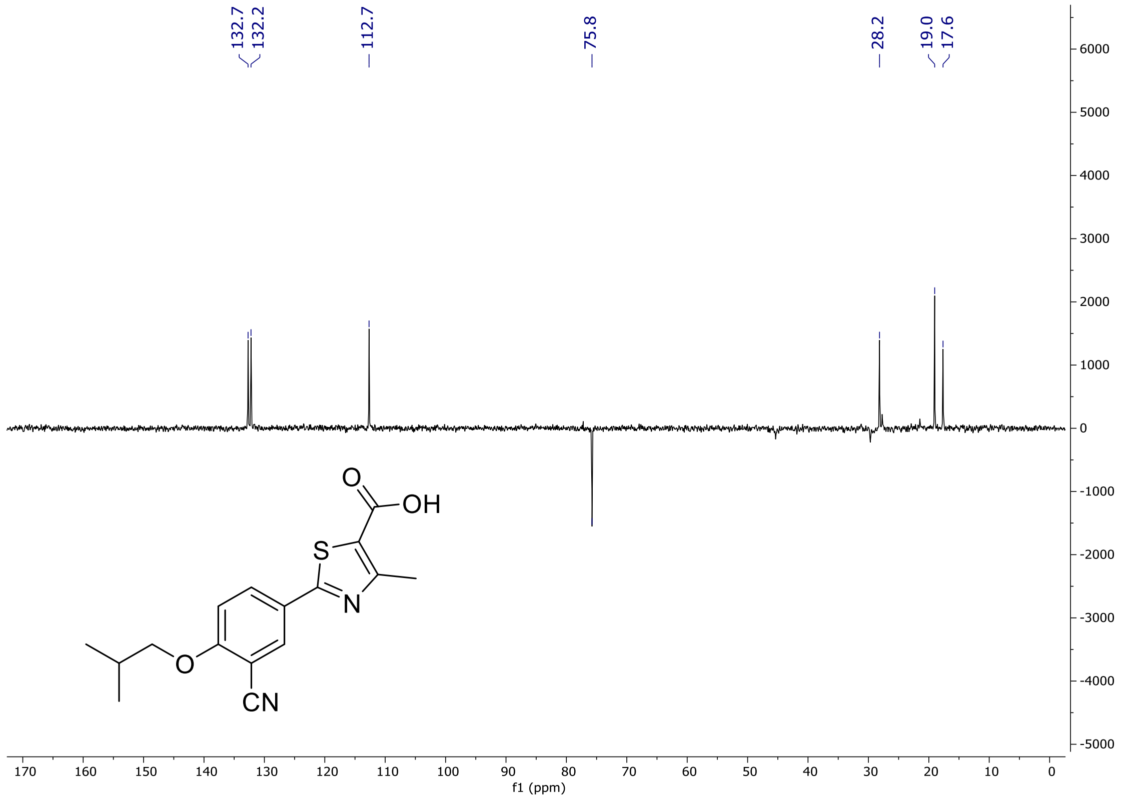

Figure SI_116: DEPT 135-NMR for **8** in $CDCl_3$ (75 MHz).

XI. Computational Supporting Information.

Computational details

All geometries considered have been optimized at DFT level employing the GGA hybrid M06-2X functional along with the def2-SVP basis set. Energies were further refined at M06-2X/def2-TZVP level. In both optimization and energy refinement, the implicit solvent scheme by means of SMD scheme was applied using THF as solvent for cavity size and ε considerations. Calculations were carried out in Gaussian16.

The given Gibbs energies correspond to those corrected according to the Morokuma scheme including the translational entropy to the total Gibbs energies.

Tautomerism of the oxime substrate

The tautomerization of the oxime becomes an essential stage necessary for the reaction to proceed (see energy profile in Figure CS1). Beginning with the oxime **1a**, a single molecule of DBU is required to deprotonate the -OH group in **1a**. DBU molecule faces the -OH from the N8 (according to the atom labels displayed in Figure CS2). The deprotonation step imposes an energy barrier of 11.6 kcal/mol, yielding the intermediate **1-τ**. Through **TS-2-τ**, which may be considered spontaneous, the proton returns to the oxime in the N position, finally yielding the active species **II** when DBU is released, and completing the tautomerization.

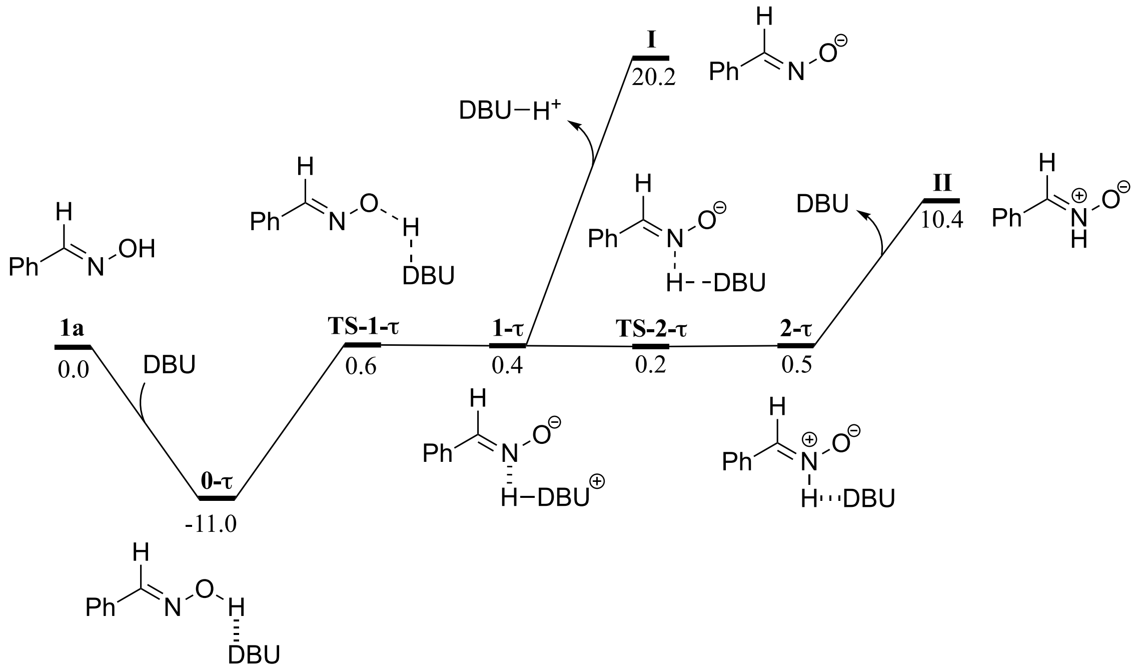

Figure CS1. Energy profile for the tautomerization of oxime **1a**. Gibbs energies are given in kcal/mol.

Alternatively, the computed direct (unassisted) H-migration pathway from **1a** to intermediate **II** indicates that this direct tautomerization proceeds through a transition state with an associated Gibbs energy barrier of 52.0 kcal/mol, rendering this pathway kinetically inaccessible under the employed reaction conditions. These findings provide compelling computational evidence that DBU-mediated proton transfer is indispensable for the activation of the aldoxime substrate, thereby rationalizing the essential role of DBU observed experimentally (Figure CS2).

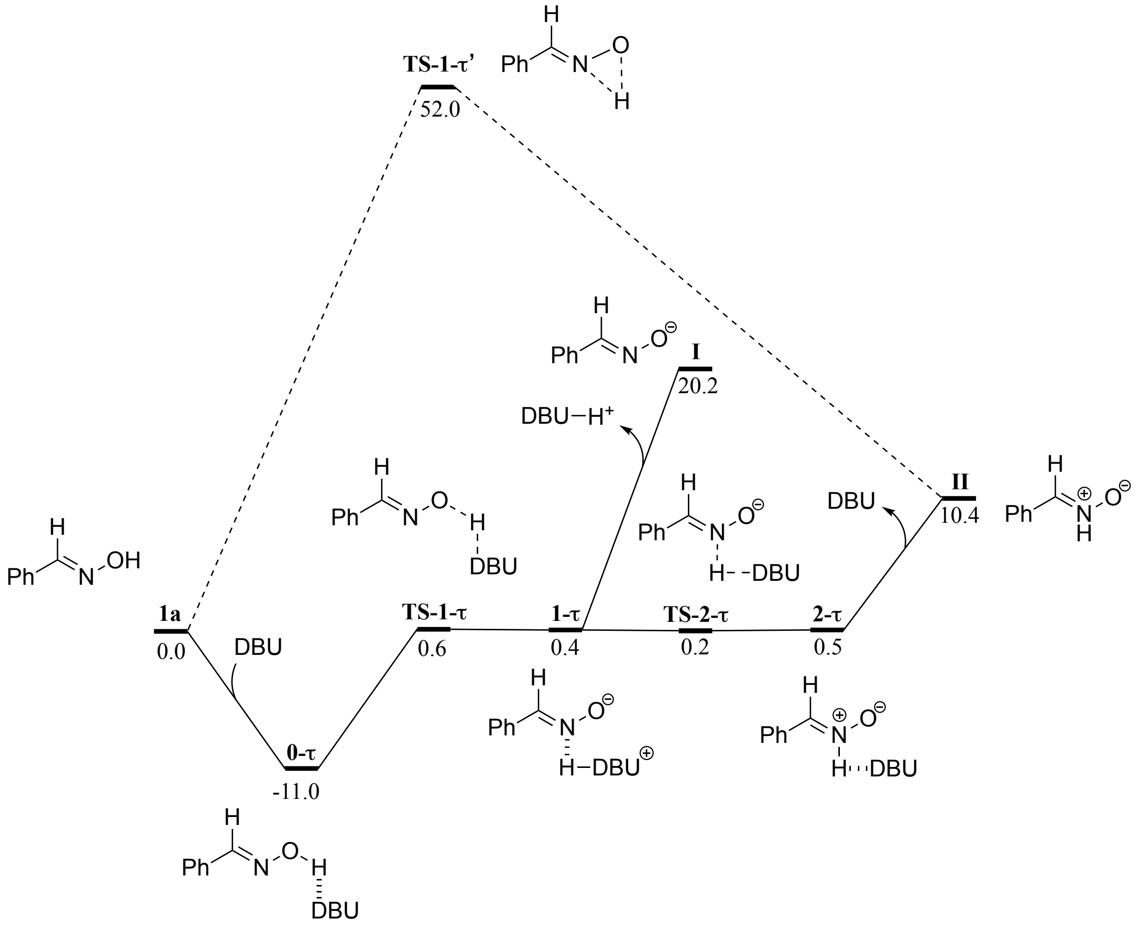

Figure CS2. Energy profile for the DBU-mediated tautomerization of oxime **1a** (solid-lines route) and the direct H-migration (dashed-lines route). Gibbs energies are given in kcal/mol.

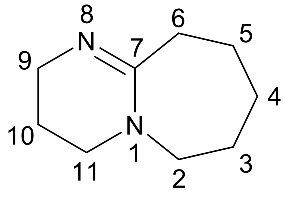

Figure CS3. Atom labels for the DBU geometry.

[CS_2·DBU] adduct formation

The formation of the $[CS_2Cl]^-$ adduct was studied in depth in previous works.[1] Therefore, only the DBU-derivate is addressed herein. The process is similar to the observed with halogen atoms. The DBU molecule plays the role of a nucleophile that can attack the carbon atom in CS_2, provoking the bending of the S-C-S angle. The reactivity of the newly formed adduct follows the similar trend as CS_2-halogenated derivatives.

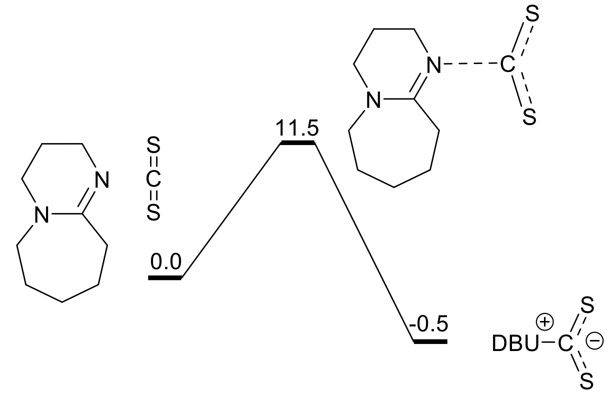

Figure CS4. Gibbs energy profile of the CS_2 activation and [CS2·DBU] adduct formation (in kcal/mol).

---

[1] (a) López-Aguilar, M.; Ríos-Lombardía, N.; Barrena-Espés, D.; Gallegos, M.; García-Álvarez, J.; Concellón, C.; del Amo, V. Unexpected Reactivity of Nitrones: Catalytic Insertion of CS_2. *Org. Lett.* **2025**, *27*, 8338-8343. (b) López-Aguilar, M.; Ríos-Lombardía, N.; Gallegos, M.; Barrena-Espés, D.; García-Álvarez, J.; Concellón, C.; del Amo, V. Organocatalytic CS_2 Insertion into Epoxides in Neat Conditions: a Straigtforward Approach for the Efficient Synthesis of Di- and Tri-thiocarbonates. *Chem. Commun.* **2025**, *61*, 3488-3491.

Reaction intermediate 2a

The five-membered heterocyclic intermediate **2a** is not experimentally detected, however, it takes part during the reaction in combination with Cl^- or DBU, depending on the path followed. Nonetheless, when the [CS2·DBU] adduct is inserted into the active oxime, there is a point where, from **B'** (see Figure 1 in the main text), the DBU molecule can be released (see Figure CS5). This step is dominated by the **TS-III**, which only requires 0.6 kcal/mol, yielding to intermediate **III**. This intermediate still shows the freed DBU interacting with the hydrogen atom in the α-carbon. The complete release of DBU finally yields the **2a** species.

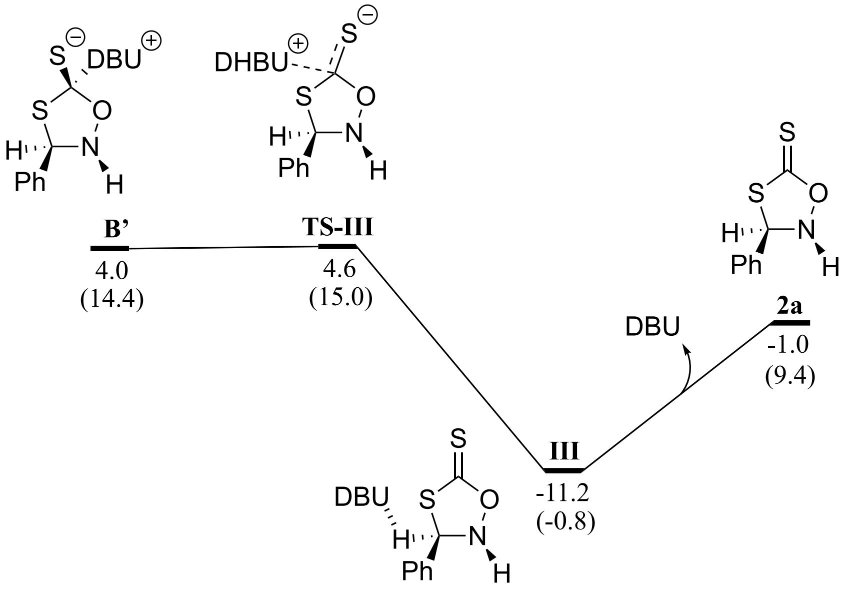

Figure CS5. Energy profile for the formation of intermediate **2a** through the DBU release from intermediate **2'**. Energies given belong to the Morokuma-corrected Gibbs energies relative to the intermediate **II** (in parentheses relative to the **1a** oxime) (in kcal/mol).

The **2a** structure can further evolve following two possible pathways: the first would comprise the reverse route of the one displayed in Figure CS5: the new insertion of DBU directly to the heterocycle. The second would consist of inserting a Cl$^-$ to the same carbon that released the DBU. Figure CS6 shows the energy profile followed by the latter. The **TS-IV** that dominates the insertion of chloride induces an immediate ring opening and requires 33.7 kcal/mol, yielding to intermediate **C**. The relative energy of the latter intermediate becomes higher due to the different routes followed. In this case, the CS_2 insertion has been initially mediated by DBU, while for the original reaction, the $[CS_2Cl]^-$ adduct is considered for reaching **C**. In the same way, all the intermediates from that point on acquire higher relative energies and may prevent this pathway in a greater extent. Hindered the pathway that follows the **2a** intermediate, the mechanism shown in the main text becomes the more feasible reaction path.

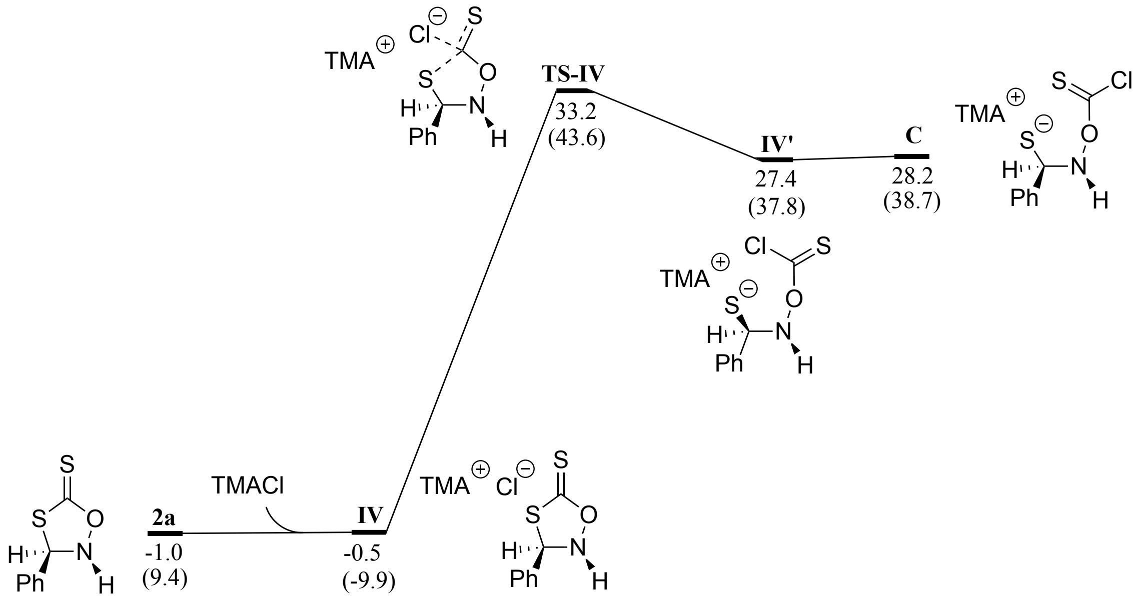

Figure CS6. Energy profile for the insertion of chlorine (from TMACl in the reaction medium) to **2a** intermediate. Morokuma-corrected Gibbs energies relative to **II** (in parentheses relative to **1a**) are given in kcal/mol.

According to computation, a DBU-mediated H-migration from intermediate **C** is also energetically viable. This pathway proceeds in two steps: (i) proton abstraction by DBU, with an associated Gibbs energy barrier of 11.9 kcal/mol, affording a significantly stabilized intermediate (**VI**), and (ii) spontaneous subsequent proton transfer from the protonated DBU to the nitrogen atom. Notably, the unassisted (intramolecular) pathway exhibits a barrier of 16.0 kcal/mol (with $[CS_2{\cdot}Cl]^-$, 15.2 kcal/mol with $[CS_2{\cdot}DBU]$). Although this value is higher than the DBU-mediated route, we consider that both mechanisms are plausible and may operate concurrently under the reaction conditions. This consideration arises from the different nature of both processes: while the intramolecular pathway benefits from entropic factors (no requirement for bimolecular encounter), the intermolecular DBU-mediated route is enthalpically more favourable but depends on effective collision with the base in solution.

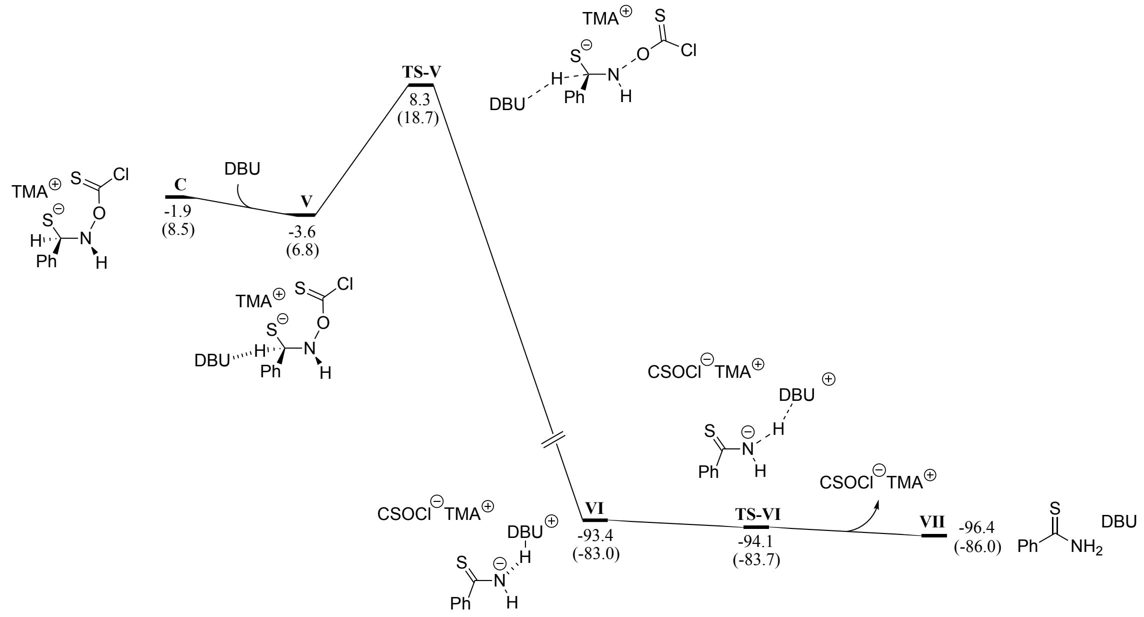

Figure CS7. Energy profile for the DBU-promoted H migration from intermediate C. Morokuma-corrected Gibbs energies relative to **II** (in parentheses relative to **1a**) are given in kcal/mol.

XII. Cartesian coordinates of optimized structures.

All structures were optimized at the DFT level. Cartesian coordinates are given in Ångström.

1 Substrate Activation and Tautomerization

1.1 1a – Aldoxime Substrate

```
16
Generated from Gaussian log
H          4.133845       0.735525       0.001704
O          3.582698      -0.058575       0.000302
N          2.302452       0.419614       0.000677
C          1.437430      -0.513732      -0.000883
H          1.758643      -1.565027      -0.002203
C         -0.004838      -0.220211      -0.000478
C         -0.491290       1.096836      -0.000546
C         -0.913028      -1.285956      -0.000017
C         -1.861346       1.335094      -0.000098
H          0.217932       1.926053      -0.001001
C         -2.286546      -1.043662       0.000441
H         -0.536730      -2.311452      -0.000013
C         -2.763178       0.266334       0.000412
H         -2.232440       2.361508      -0.000193
H         -2.985625      -1.881683       0.000819
H         -3.837597       0.458164       0.000743
```

1.2 u

ndec-7-ene]DBU – 1,8-Diazabicyclo[5.4.0]undec-7-ene

```
27
Generated from Gaussian log
C          0.851039       1.460502      -0.265651
C         -0.367906      -0.725053       0.121887
C          2.099433       1.132223       0.548144
C          0.926509      -1.480290       0.377345
C          2.947104       0.007524      -0.046243
C          2.090289      -1.147977      -0.561019
H          1.092092       1.443162      -1.347036
H          1.236720      -1.327863       1.424724
H          1.784967       0.888154       1.574592
H          3.645721      -0.360314       0.722180
H          0.556900       2.494160      -0.037047
H          2.707200       2.046025       0.622678
H          0.642251      -2.534138       0.284845
H          3.565937       0.401575      -0.868047
H          2.717231      -2.041850      -0.692786
H          1.691744      -0.909082      -1.560377
N         -0.337403       0.653916       0.013031
N         -1.428740      -1.450439       0.065612
C         -2.724367      -0.834443      -0.144920
C         -1.582622       1.341483      -0.311472
```

H	-1.707373	1.418442	-1.409021
H	-1.513482	2.368345	0.076625
C	-2.768670	0.619092	0.297573
H	-3.483417	-1.425334	0.391943
H	-2.989849	-0.905129	-1.215728
H	-2.702424	0.676048	1.395878
H	-3.706066	1.105098	-0.007789

1.3 H-DBU$^+$ – Protonated DBU

28
Generated from Gaussian log

C	0.904912	1.498812	-0.226516
C	-0.307361	-0.666297	0.054577
C	2.078475	1.093447	0.654495
C	0.935343	-1.500404	0.252688
C	2.963755	0.009356	0.044085
C	2.138314	-1.099957	-0.601891
H	1.209625	1.572974	-1.283662
H	1.192672	-1.472366	1.323898
H	1.687972	0.781733	1.635845
H	3.607581	-0.413646	0.830381
H	0.561881	2.494096	0.077777
H	2.675122	1.997675	0.839620
H	0.643150	-2.534661	0.034193
H	3.632757	0.451395	-0.709605
H	2.761724	-1.989866	-0.762119
H	1.787390	-0.789012	-1.598357
N	-0.297603	0.643609	-0.139725
N	-1.449526	-1.339673	0.085612
C	-2.788229	-0.769601	-0.015448
C	-1.565344	1.335755	-0.431836
H	-1.743051	1.304840	-1.518917
H	-1.440445	2.384214	-0.140229
C	-2.716962	0.703566	0.326111
H	-3.441193	-1.314469	0.677509
H	-3.170451	-0.920909	-1.036097
H	-2.560582	0.835854	1.406925
H	-3.655680	1.201753	0.054625
H	-1.375988	-2.345209	0.209417

1.4 I – Aldoxime-DBU Complex

15
Generated from Gaussian log

C	-1.775301	-1.338890	-0.000058
C	-0.411266	-1.075464	0.000144
C	0.083280	0.252454	0.000309
C	-0.877212	1.289535	0.000065
C	-2.243631	1.018796	-0.000070
C	-2.711143	-0.297244	-0.000133
H	-2.118610	-2.376623	-0.000024
H	0.313514	-1.890331	0.000218

H	-0.532158	2.327039	0.000095
H	-2.954127	1.849241	-0.000213
H	-3.781628	-0.509369	-0.000321
C	1.499552	0.553760	0.000108
H	1.832969	1.603358	-0.000328
N	2.406853	-0.403196	-0.000042
O	3.625799	-0.074829	-0.000165

1.5 II – Deprotonated Aldoxime (Nitrone-like Intermediate)

16
Generated from Gaussian log

H	-2.137317	1.279657	-0.000809
O	-3.642165	0.048733	-0.000087
N	-2.410313	0.282116	-0.000216
C	-1.440309	-0.585045	0.000292
H	-1.750175	-1.631623	0.000885
C	-0.022234	-0.233249	0.000113
C	0.463730	1.087645	0.000175
C	0.907265	-1.287297	-0.000052
C	1.832758	1.335741	0.000048
H	-0.217377	1.941156	0.000375
C	2.276304	-1.033563	-0.000133
H	0.543037	-2.316784	-0.000066
C	2.746394	0.279513	-0.000098
H	2.189631	2.367059	0.000127
H	2.979622	-1.868066	-0.000227
H	3.818640	0.481457	-0.000156

1.6 0-tau – Tautomerization Reactant Complex

43
Generated from Gaussian log

C	-4.146442	-0.575395	1.100542
C	-2.246427	0.242246	-0.350104
C	-4.583515	-1.706779	0.176176
C	-2.035025	-1.118340	-0.991603
C	-3.568052	-2.843808	0.072616
C	-2.138310	-2.321260	-0.051369
H	-3.672410	-0.990960	2.009887
H	-2.744399	-1.231918	-1.828011
H	-4.799298	-1.279127	-0.815132
H	-3.814353	-3.467757	-0.801152
H	-5.044664	-0.041912	1.438708
H	-5.538185	-2.100957	0.554024
H	-1.035646	-1.065936	-1.438322
H	-3.646617	-3.496166	0.956579
H	-1.480213	-3.121309	-0.419278
H	-1.747341	-2.040570	0.939775
N	-3.288755	0.455433	0.507921
N	-1.394720	1.152813	-0.699029
C	-1.470531	2.505508	-0.179302
C	-3.387064	1.753477	1.174345

H	-2.820690	1.735821	2.123593
H	-4.442650	1.921611	1.428317
C	-2.873645	2.862470	0.275561
H	-1.122613	3.195941	-0.962613
H	-0.760097	2.607884	0.660044
H	-3.537095	2.958084	-0.598327
H	-2.885900	3.819802	0.814261
H	-0.136292	0.810691	-1.551968
O	0.753385	0.567459	-2.016392
N	1.617182	0.428411	-0.995492
C	2.805006	0.124847	-1.348480
H	3.050638	-0.001338	-2.412917
C	3.874595	-0.063610	-0.354654
C	3.638206	0.074967	1.023245
C	5.165322	-0.389984	-0.791073
C	4.671858	-0.108826	1.935577
H	2.633026	0.328601	1.363312
C	6.200424	-0.573980	0.125848
H	5.356003	-0.499396	-1.861326
C	5.957388	-0.433886	1.491461
H	4.476339	0.001696	3.003933
H	7.200894	-0.827704	-0.229320
H	6.765820	-0.577215	2.210555

1.7 TS-1-tau – Transition State for DBU-Mediated Tautomerization

43
Generated from Gaussian log

C	4.363641	0.819135	-0.375440
C	2.018284	0.101596	0.129122
C	5.010125	-0.447653	0.168512
C	2.307411	-1.376155	0.049278
C	4.725133	-1.697707	-0.661254
C	3.257999	-1.783893	-1.076905
H	4.468816	0.869960	-1.472986
H	2.708371	-1.690893	1.026826
H	4.689888	-0.583757	1.212791
H	4.998658	-2.586747	-0.072035
H	4.897303	1.685441	0.034035
H	6.094268	-0.268115	0.204492
H	1.327859	-1.868186	-0.027618
H	5.363511	-1.704649	-1.558320
H	3.014770	-2.809290	-1.387763
H	3.072652	-1.148365	-1.957566
N	2.949621	1.044164	-0.022281
N	0.760239	0.421232	0.363052
C	0.246324	1.772028	0.531009
C	2.546285	2.458346	-0.029858
H	2.282419	2.752013	-1.059500
H	3.417736	3.050310	0.273081
C	1.382494	2.696767	0.913794
H	-0.534761	1.738288	1.302230
H	-0.225486	2.102570	-0.407979

```
H         1.701951       2.497332       1.947586
H         1.062193       3.744266       0.852344
H         0.050631      -0.332174       0.418789
O        -0.582839      -2.274712       0.869400
N        -1.429358      -1.390496       0.488138
C        -2.700194      -1.675988       0.507275
H        -3.011711      -2.671270       0.856413
C        -3.714176      -0.717572       0.084112
C        -3.387969       0.552499      -0.438112
C        -5.079931      -1.046784       0.185348
C        -4.380503       1.444872      -0.828769
H        -2.335199       0.821103      -0.535528
C        -6.071923      -0.151052      -0.209310
H        -5.359901      -2.025315       0.583684
C        -5.732372       1.103413      -0.718107
H        -4.097775       2.420953      -1.229926
H        -7.122212      -0.436617      -0.117519
H        -6.508551       1.805420      -1.027273
```

1.8 1-tau – Tautomerization Intermediate

```
43
Generated from Gaussian log
C        -4.044756       1.037321       0.254196
C        -1.741508       0.132141      -0.135995
C        -4.785896      -0.204552      -0.224149
C        -2.159363      -1.312781      -0.024703
C        -4.599395      -1.427527       0.672583
C        -3.145641      -1.605156       1.108671
H        -4.146825       1.150884       1.347643
H        -2.598327      -1.601103      -0.993838
H        -4.478242      -0.419768      -1.258623
H        -4.935950      -2.322683       0.126810
H        -4.514594       1.918549      -0.199322
H        -5.853382       0.054520      -0.273255
H        -1.241649      -1.913372       0.050294
H        -5.242834      -1.340073       1.561800
H        -2.983004      -2.632363       1.463145
H        -2.921484      -0.950243       1.965860
N        -2.618272       1.141511      -0.103788
N        -0.451562       0.345060      -0.290679
C         0.152038       1.651531      -0.510487
C        -2.130707       2.524052      -0.203270
H        -1.928534       2.912821       0.809088
H        -2.939133       3.125959      -0.635538
C        -0.887348       2.601011      -1.068329
H         0.998927       1.517208      -1.197253
H         0.556733       2.037570       0.438689
H        -1.140262       2.321357      -2.102137
H        -0.504331       3.629194      -1.077823
H         0.213380      -0.500035      -0.270441
O         0.502768      -2.850077      -0.714134
N         1.210409      -1.847050      -0.376736
```

```
C          2.512241      -1.911267      -0.465252
H          2.963711      -2.823502      -0.878897
C          3.367640      -0.810839      -0.036503
C          2.909134       0.211901       0.820778
C          4.709838      -0.750200      -0.460349
C          3.741721       1.261733       1.197799
H          1.890521       0.156871       1.210006
C          5.544261       0.295362      -0.071622
H          5.094206      -1.537524      -1.113549
C          5.065714       1.315452       0.753161
H          3.358694       2.039719       1.862540
H          6.579271       0.315884      -0.419718
H          5.719105       2.135004       1.056838
```

1.9 TS-2-tau – Transition State for Second Tautomerization Step

```
43
Generated from Gaussian log
C         -3.945483       0.991570       0.375956
C         -1.654273       0.095060      -0.143630
C         -4.704306      -0.297421       0.080927
C         -2.036755      -1.336829       0.168798
C         -4.412223      -1.424779       1.069964
C         -2.924393      -1.523537       1.401767
H         -3.936773       1.190669       1.462876
H         -2.526212      -1.759193      -0.723527
H         -4.475684      -0.607011      -0.950278
H         -4.762219      -2.377062       0.641654
H         -4.486891       1.825304      -0.088025
H         -5.778484      -0.062680       0.098647
H         -1.098211      -1.892159       0.262235
H         -4.985911      -1.266545       1.996596
H         -2.707436      -2.502706       1.851233
H         -2.653528      -0.774722       2.163373
N         -2.576839       1.083430      -0.153619
N         -0.396581       0.294906      -0.429006
C          0.122376       1.597218      -0.812298
C         -2.159299       2.451539      -0.476398
H         -1.915603       2.995258       0.453199
H         -3.016598       2.958563      -0.938422
C         -0.971973       2.451900      -1.420030
H          0.951831       1.434156      -1.517254
H          0.551291       2.101862       0.070302
H         -1.274868       2.037219      -2.393718
H         -0.625033       3.480883      -1.581963
H          0.438542      -0.673454      -0.528699
O          0.496140      -2.595892      -1.374757
N          1.189801      -1.687326      -0.827847
C          2.487965      -1.758654      -0.728917
H          2.980886      -2.626341      -1.181930
C          3.282363      -0.733410      -0.057754
C          2.723192       0.192664       0.845646
C          4.667260      -0.664489      -0.297606
```

C	3.513265	1.167907	1.448451
H	1.661123	0.129974	1.091158
C	5.456323	0.305412	0.316179
H	5.122777	-1.382066	-0.984083
C	4.883919	1.234418	1.186826
H	3.056100	1.875041	2.144059
H	6.528047	0.338189	0.109836
H	5.501308	1.995476	1.666796

1.10 2-tau – Tautomerization Product

43

Generated from Gaussian log

C	-3.952707	1.019040	0.370325
C	-1.673341	0.097491	-0.197688
C	-4.756617	-0.244149	0.078771
C	-2.108924	-1.333635	0.053620
C	-4.457203	-1.402776	1.029872
C	-2.961502	-1.548517	1.308138
H	-3.902291	1.190702	1.462443
H	-2.655093	-1.690571	-0.835117
H	-4.576319	-0.534915	-0.967345
H	-4.846596	-2.335216	0.591422
H	-4.497116	1.875062	-0.049664
H	-5.823525	0.015017	0.144783
H	-1.190528	-1.929199	0.085306
H	-4.995659	-1.254373	1.979259
H	-2.756609	-2.548256	1.717064
H	-2.649074	-0.834190	2.087064
N	-2.609858	1.096423	-0.214734
N	-0.411463	0.275563	-0.431515
C	0.105181	1.598759	-0.738104
C	-2.179760	2.467290	-0.485695
H	-1.973180	2.996100	0.463250
H	-3.015431	2.990110	-0.972530
C	-0.951526	2.483218	-1.373769
H	0.978596	1.482968	-1.399184
H	0.481161	2.075364	0.185221
H	-1.212686	2.094522	-2.370571
H	-0.589413	3.513057	-1.496639
H	0.711388	-0.981550	-0.512981
O	0.619526	-2.834202	-1.175729
N	1.298592	-1.868091	-0.731574
C	2.591357	-1.864058	-0.583972
H	3.117606	-2.760351	-0.921807
C	3.326567	-0.740878	-0.005511
C	2.717627	0.218223	0.824506
C	4.703207	-0.625941	-0.264397
C	3.459280	1.276056	1.344266
H	1.659181	0.124122	1.076220
C	5.442635	0.428271	0.265863
H	5.190651	-1.372331	-0.895518
C	4.823312	1.388740	1.067198

H	2.969657	2.011523	1.985788
H	6.510241	0.501171	0.050417
H	5.402413	2.214788	1.483176

1.11 TS-1-taup – Transition State for Direct (Unassisted) Tautomerization

16
Generated from Gaussian log

H	-3.057300	1.161110	-0.000407
O	-3.677531	0.026785	-0.000110
N	-2.338144	0.290847	-0.000558
C	-1.417739	-0.589164	0.000416
H	-1.745579	-1.637927	0.001417
C	0.009287	-0.254735	0.000299
C	0.455771	1.076707	0.000321
C	0.947641	-1.295178	0.000003
C	1.817887	1.355176	0.000084
H	-0.274234	1.888151	0.000509
C	2.312336	-1.010957	-0.000250
H	0.600846	-2.330730	-0.000079
C	2.749969	0.312963	-0.000193
H	2.158335	2.391963	0.000088
H	3.036039	-1.827673	-0.000457
H	3.818236	0.536022	-0.000370

2 Key Reaction Intermediates

2.1 CS_2 – Carbon Disulfide

```
3
Generated from Gaussian log
S          0.000000     -0.000000      1.553382
C         -0.000000      0.000000     -0.000020
S         -0.000000      0.000000     -1.553374
```

2.2 CS_2-DBU – Carbon Disulfide-DBU Adduct

```
30
Generated from Gaussian log
C          2.803910      0.709744     -0.068398
C          0.369667      0.096743     -0.108613
C          3.160273     -0.490772      0.796547
C          0.595134     -1.398746     -0.157236
C          3.095296     -1.816382      0.044634
C          1.866284     -1.874221     -0.854314
H          3.269809      0.629943     -1.065007
H          0.516523     -1.790478      0.869563
H          2.491808     -0.501736      1.671943
H          3.072969     -2.646794      0.767017
H          3.213959      1.611520      0.403082
H          4.174447     -0.328702      1.188600
H         -0.274046     -1.799947     -0.693571
H          4.002583     -1.948371     -0.564897
H          1.699239     -2.904291     -1.198108
H          2.026591     -1.272491     -1.762782
N          1.362323      0.986322     -0.220277
N         -0.885240      0.504875      0.002940
C         -1.309574      1.905132      0.118278
C          1.019717      2.391357     -0.471197
H          0.749220      2.512919     -1.532649
H          1.912389      2.994840     -0.278079
C         -0.132749      2.803720      0.422347
H         -2.062199      1.941248      0.917565
H         -1.799874      2.180743     -0.827492
H          0.168777      2.704809      1.475666
H         -0.408493      3.850249      0.240484
C         -2.028984     -0.438248      0.064944
S         -2.359945     -1.022101      1.590955
S         -2.810501     -0.665511     -1.391701
```

2.3 CS_2Cl^- – Chlorodithioformate Anion

```
4
Generated from Gaussian log
C         -0.001075     -0.187365     -0.000135
S         -1.519134     -0.849258      0.000019
Cl         0.008752      1.679220      0.000013
S          1.510238     -0.864651      0.000019
```

2.4 TMA^+ – Tetrabutylammonium Cation (truncated as TMA)

```
17
Generated from Gaussian log
N        -0.000151     -0.000107      0.000244
C        -0.683475      0.760644     -1.083750
H        -1.325752      0.070377     -1.641797
H        -1.283207      1.555563     -0.626318
H         0.080355      1.189019     -1.742156
C         0.819761     -1.091095     -0.598617
H         1.564389     -0.641225     -1.264754
H         1.313665     -1.639440      0.211405
H         0.156354     -1.757054     -1.161488
C        -1.021187     -0.588656      0.912064
H        -0.503693     -1.140176      1.704831
H        -1.616697      0.225737      1.339655
H        -1.658280     -1.264064      0.330021
C         0.884745      0.919193      0.770188
H         0.268155      1.714626      1.203740
H         1.379272      0.344762      1.561513
H         1.627434      1.342109      0.084325
```

2.5 Cl_TMA – Chloride-TMA Ion Pair

```
18
Generated from Gaussian log
Cl        2.645707      0.000043      0.000093
N        -1.057610     -0.000294      0.000195
C        -0.544436      0.978244      1.003466
H         0.554377      0.939296      0.960297
H        -0.920122      1.973018      0.736571
H        -0.915936      0.685043      1.992406
C        -0.544395     -1.358595      0.345092
H        -0.919497     -1.626875      1.339780
H        -0.916029     -2.067215     -0.404438
H         0.554450     -1.301813      0.332725
C        -0.543986      0.380166     -1.348614
H        -0.918608     -0.347526     -2.078221
H        -0.915037      1.383674     -1.588147
H         0.554852      0.362618     -1.292608
C        -2.542300      0.000194     -0.000166
H        -2.893476      1.006678     -0.255746
H        -2.894277     -0.724300     -0.743549
H        -2.893741     -0.281328      0.999314
```

2.6 CS_2Cl_TMA – Chlorodithioformate-TMA Ion Pair

```
21
Generated from Gaussian log
C        -1.906601      0.019793     -0.064287
S        -1.128304     -1.451345     -0.183311
Cl       -3.712333     -0.122662      0.206687
N         2.656555      0.001848      0.050807
```

C	4.041383	0.535277	0.145850
H	4.495154	0.172320	1.075061
H	4.612350	0.181818	-0.720431
H	3.993894	1.630178	0.147923
C	1.854059	0.466267	1.219549
H	1.831984	1.561600	1.212807
H	0.837016	0.067673	1.114008
H	2.333025	0.096293	2.133374
C	2.692342	-1.487843	0.046816
H	1.659568	-1.848280	-0.027175
H	3.282217	-1.820870	-0.814628
H	3.155293	-1.826354	0.981035
C	2.016759	0.482323	-1.208786
H	2.604572	0.111431	-2.056367
H	0.988594	0.099310	-1.235858
H	2.009085	1.578092	-1.195863
S	-1.338986	1.574733	-0.149068

3 CS_2-Cl^- Mediated Pathway

3.1 1 – Reactant Complex (Nitrone + CS_2Cl^-)

```
37
Generated from Gaussian log
H        -1.439342     -1.559107      0.784619
O        -2.347696     -3.266286      0.608693
N        -2.337651     -2.017652      0.519728
C        -3.318216     -1.253478      0.131470
H        -4.233865     -1.773064     -0.157124
C        -3.238477      0.201173      0.061967
C        -2.125588      0.945710      0.500376
C        -4.339919      0.894264     -0.469093
C        -2.128849      2.334179      0.404077
H        -1.251693      0.442471      0.922667
C        -4.334222      2.283504     -0.566374
H        -5.209010      0.327662     -0.810508
C        -3.227606      3.011875     -0.130474
H        -1.262866      2.896936      0.757807
H        -5.200783      2.798904     -0.984493
H        -3.221553      4.100782     -0.201361
C         1.058585     -1.721466     -0.196107
S         0.857763     -1.525191      1.456489
S         0.179706     -1.124055     -1.460033
Cl        2.501094     -2.738941     -0.653625
N         2.972218      1.905404      0.088183
C         3.521693      3.025361     -0.723028
H         3.134215      3.969200     -0.323276
H         4.615415      3.002801     -0.656531
H         3.200316      2.892449     -1.762044
C         1.480127      1.948108      0.052568
H         1.095797      1.074963      0.597674
H         1.157108      2.882488      0.526522
H         1.153787      1.913037     -0.992695
C         3.449518      0.603547     -0.461795
H         4.542497      0.638352     -0.535297
H         3.140880     -0.190847      0.226075
H         2.997489      0.447704     -1.448247
C         3.433243      2.031463      1.498918
H         2.975719      1.223586      2.081864
H         4.525494      1.944211      1.517755
H         3.120157      3.007997      1.884966
```

3.2 TS-1 – Transition State for C-S Bond Formation

```
37
Generated from Gaussian log
H         1.831680      1.302759      1.547595
O         0.850156      2.590819      0.430238
N         1.824780      1.762232      0.626008
C         2.363980      1.148886     -0.402805
H         2.399466      1.712440     -1.334072
C         3.191425     -0.044747     -0.198306
```

```
C          2.819820     -0.999157      0.762110
C          4.343195     -0.245286     -0.969502
C          3.617953     -2.119958      0.971432
H          1.879656     -0.869453      1.307327
C          5.136300     -1.371244     -0.758039
H          4.618622      0.490246     -1.728078
C          4.778653     -2.304707      0.215984
H          3.326256     -2.862072      1.716366
H          6.037163     -1.521169     -1.355242
H          5.400148     -3.186934      0.379278
C         -0.579581      1.422464      0.097414
S         -0.865295      0.698907      1.646088
S         -0.024394      0.525003     -1.259747
Cl        -1.713141      2.803252     -0.329154
N         -3.625276     -1.592438     -0.172531
C         -4.586071     -2.477666     -0.880766
H         -4.532359     -3.478878     -0.437884
H         -5.595007     -2.064587     -0.766762
H         -4.308802     -2.516034     -1.940394
C         -2.246307     -2.152101     -0.285163
H         -1.553421     -1.463156      0.219364
H         -2.240879     -3.141223      0.188330
H         -1.993262     -2.234533     -1.348117
C         -3.658124     -0.227476     -0.772807
H         -4.692812      0.133875     -0.747103
H         -3.007506      0.424264     -0.178721
H         -3.296360     -0.288452     -1.805237
C         -3.994337     -1.493079      1.267560
H         -3.259926     -0.839433      1.755570
H         -5.001649     -1.067320      1.340742
H         -3.971800     -2.498882      1.702286
```

3.3 2 – Thiocarbonyl Intermediate

```
37
Generated from Gaussian log
N         -1.125230     -1.755577     -1.190106
C         -0.488742     -1.966519      0.095147
O         -2.433007     -1.377275     -0.908843
S         -2.076655      1.277896     -1.057089
C         -2.397903     -0.180358     -0.127388
S         -1.109171     -0.588411      1.189765
Cl        -4.045116     -0.192575      0.686069
N          1.499303      2.671495      0.102705
C          2.816134      3.180332      0.566157
H          3.168553      2.539750      1.382600
H          2.689991      4.210719      0.917748
H          3.520154      3.147432     -0.273341
C          0.988745      3.522729     -1.008107
H          0.024950      3.109049     -1.328897
H          1.714669      3.498023     -1.828750
H          0.867900      4.546531     -0.636108
C          1.644259      1.268944     -0.388216
```

```
H          2.342564       1.271109      -1.233586
H          0.647734       0.918856      -0.691933
H          2.033803       0.651881       0.430785
C          0.519499       2.701811       1.227429
H          0.437258       3.736230       1.581359
H          0.890441       2.050308       2.026799
H         -0.443026       2.333904       0.844434
H         -0.701325      -0.936800      -1.650313
C          1.015089      -2.000940       0.014328
C          1.687644      -1.957510      -1.210850
C          1.763498      -2.071968       1.197531
C          3.084895      -1.955224      -1.248800
H          1.126534      -1.933733      -2.146369
C          3.154803      -2.072562       1.158600
H          1.246606      -2.117626       2.159718
C          3.821798      -2.006083      -0.067938
H          3.596225      -1.917550      -2.212474
H          3.722953      -2.125248       2.089113
H          4.912728      -2.003777      -0.099951
H         -0.871301      -2.906169       0.518364
```

3.4 TS-2 – Transition State for COS Elimination

```
37
Generated from Gaussian log
N         -1.629147      -1.718470       0.145956
C         -1.784822      -0.628341      -0.865134
O         -0.417560      -2.375161      -0.132983
S          0.861323      -1.228855       1.901620
C          0.676390      -1.853179       0.389910
S         -0.287636       0.356640      -1.127518
Cl         2.050900      -2.451150      -0.534262
N          3.516245       1.552716      -0.097179
C          4.520171       2.639864      -0.249497
H          4.078901       3.439610      -0.854770
H          5.406569       2.230530      -0.747212
H          4.781663       3.016576       0.745820
C          4.099956       0.447574       0.713324
H          3.338521      -0.330439       0.844436
H          4.396543       0.851533       1.688293
H          4.973099       0.049927       0.183193
C          2.303044       2.082589       0.590239
H          2.591815       2.411251       1.595408
H          1.543283       1.289876       0.626977
H          1.918340       2.926931       0.006390
C          3.133905       1.034898      -1.442592
H          4.034705       0.645088      -1.930850
H          2.711692       1.863052      -2.023813
H          2.376976       0.249388      -1.318558
H         -1.493414      -1.283404       1.067485
C         -2.983779       0.167383      -0.384187
C         -4.225729       0.037461      -1.011960
C         -2.873860       1.013944       0.727069
```

C	-5.335719	0.748394	-0.549138
H	-4.322596	-0.625918	-1.874844
C	-3.982241	1.711010	1.200820
H	-1.893991	1.134698	1.195598
C	-5.218176	1.583647	0.560338
H	-6.296692	0.644239	-1.056783
H	-3.882549	2.364979	2.069523
H	-6.085192	2.137344	0.925545
H	-2.066026	-1.140170	-1.797740

3.5 3 – Thioamide Precursor

37
Generated from Gaussian log

N	-0.873852	-1.593533	-0.681402
C	-0.064122	-1.769751	0.552050
O	-2.226927	-1.581132	-0.291687
S	-2.527399	0.898290	-1.190878
C	-2.911538	-0.481759	-0.403371
S	-0.396519	-0.539320	1.848627
Cl	-4.475376	-0.744291	0.314391
N	1.169534	2.906520	0.051575
C	2.406255	3.601308	0.499569
H	2.856255	3.017289	1.310244
H	2.138362	4.602971	0.854865
H	3.096472	3.669449	-0.348931
C	0.568845	3.639452	-1.095512
H	-0.360457	3.130763	-1.377787
H	1.278321	3.627272	-1.930490
H	0.360918	4.669814	-0.784700
C	1.508070	1.517141	-0.375076
H	2.285967	1.571102	-1.146368
H	0.593258	1.057392	-0.764686
H	1.835383	0.947444	0.501299
C	0.187323	2.857682	1.174080
H	-0.105562	3.885856	1.417122
H	0.663397	2.364891	2.027987
H	-0.668794	2.246965	0.863337
H	-0.694565	-0.651111	-1.049801
H	-0.337354	-2.775065	0.917016
C	1.392249	-1.832136	0.100967
C	1.768662	-1.946244	-1.244107
C	2.407124	-1.777384	1.069072
C	3.118762	-1.977631	-1.610689
H	1.005382	-2.017669	-2.019500
C	3.749794	-1.830311	0.706880
H	2.110778	-1.661106	2.112421
C	4.114793	-1.920702	-0.639874
H	3.386713	-2.054852	-2.666581
H	4.520062	-1.791720	1.480137
H	5.167878	-1.950112	-0.925871

3.6 TS-3 – Transition State for Final H-Migration

```
37
Generated from Gaussian log
N          0.504348       0.177257      -1.405955
C          1.239768      -0.932568      -0.989355
O         -0.270021       0.902871       0.096025
S         -1.991039       2.714523       0.881819
C         -0.710592       2.063226       0.066150
S          0.401020      -2.456666      -0.654577
Cl         0.250309       3.191961      -1.014066
N         -3.298275      -1.285736       0.281046
C         -2.836577      -0.551269      -0.933162
H         -2.829309       0.519977      -0.699853
H         -3.541767      -0.760906      -1.746296
H         -1.827182      -0.903904      -1.183355
C         -3.356201      -2.740847      -0.026770
H         -3.663850      -3.278713       0.877359
H         -2.354144      -3.054513      -0.344798
H         -4.082593      -2.900122      -0.832269
C         -2.335866      -1.056785       1.400067
H         -1.350714      -1.423012       1.079314
H         -2.699473      -1.605322       2.277149
H         -2.293734       0.020600       1.599616
C         -4.643234      -0.792452       0.676658
H         -5.336938      -0.954789      -0.156463
H         -4.562937       0.277268       0.903604
H         -4.974291      -1.345855       1.562887
H          1.139970       0.979415      -1.531257
C          2.533243      -0.671764      -0.231735
C          3.428668      -1.727915      -0.018958
C          2.863690       0.599834       0.250424
C          4.630138      -1.515951       0.650748
H          3.155339      -2.721417      -0.376872
C          4.061965       0.808225       0.937285
H          2.186413       1.444771       0.114788
C          4.950544      -0.245629       1.135779
H          5.319167      -2.349014       0.801905
H          4.295319       1.803724       1.319033
H          5.888041      -0.081017       1.669895
H          1.482122      -0.936466      -2.127758
```

3.7 3a – Primary Thioamide Product

```
37
Generated from Gaussian log
N         -0.789067       0.593854      -2.088057
C         -1.378184       1.475552      -1.286039
O          2.079173      -1.209076       1.061020
S          1.662033      -1.665968      -1.527911
C          2.350287      -1.084264      -0.089512
S         -2.964117       1.950441      -1.530388
Cl         3.911026      -0.010937      -0.402870
C         -0.547096       1.988423      -0.151741
```

C	0.850342	1.858480	-0.154620
C	-1.169871	2.571708	0.961768
C	1.601467	2.280233	0.941549
H	1.381563	1.433884	-1.009542
C	-0.417761	2.997010	2.052320
H	-2.255157	2.678759	0.956529
C	0.970644	2.847352	2.047525
H	2.683648	2.144839	0.923592
H	-0.918081	3.443726	2.913186
H	1.560590	3.173806	2.905835
N	-1.668476	-2.369410	0.796184
C	-2.854135	-2.670978	1.641506
H	-2.574936	-2.536704	2.692812
H	-3.160318	-3.707074	1.457637
H	-3.661285	-1.980646	1.371407
C	-0.549706	-3.292093	1.145018
H	-0.882465	-4.321201	0.967928
H	-0.298918	-3.146251	2.201857
H	0.308895	-3.041513	0.509664
C	-2.017533	-2.539076	-0.643379
H	-2.821227	-1.835720	-0.892581
H	-2.348273	-3.571893	-0.801708
H	-1.117930	-2.325673	-1.234211
C	-1.236002	-0.960486	1.022870
H	-0.971016	-0.836764	2.079422
H	-2.070303	-0.300553	0.754386
H	-0.361581	-0.770572	0.389602
H	-1.316198	0.230438	-2.876225
H	0.123588	0.173791	-1.896659

4 CS_2-DBU Mediated Pathway

4.1 1p – Reactant Complex (Nitrone + CS_2-DBU)

```
46
Generated from Gaussian log
H          2.137827       0.931594       0.720617
O          1.791185       2.730170       0.051258
N          2.516479       1.704553       0.132358
C          3.676337       1.525187      -0.423252
H          4.056276       2.364795      -1.008778
C          4.455370       0.297087      -0.288360
C          3.952475      -0.879029       0.300149
C          5.771208       0.295970      -0.781270
C          4.759091      -2.008756       0.403044
H          2.923860      -0.926191       0.667094
C          6.570387      -0.839732      -0.678457
H          6.166359       1.201933      -1.246150
C          6.068715      -1.997028      -0.082471
H          4.355023      -2.912782       0.862191
H          7.590627      -0.819845      -1.065727
H          6.692709      -2.888551      -0.000513
N         -1.541951       0.605570       0.306069
C         -2.672489      -0.053323       0.087602
C         -1.443109       2.069168       0.410553
C         -2.584259      -1.566572       0.102478
N         -3.818518       0.604156      -0.111188
C         -2.553146       2.698004      -0.395216
H         -0.443926       2.352636       0.052192
H         -1.508382       2.342026       1.475220
C         -3.796099      -2.315456       0.657298
H         -2.335189      -1.905655      -0.915768
H         -1.712486      -1.807186       0.720587
C         -5.135511      -0.015210      -0.363130
C         -3.867909       2.070018       0.012600
H         -2.375152       2.532857      -1.468488
H         -2.586019       3.779867      -0.214526
C         -4.879784      -2.545812      -0.389060
H         -3.443721      -3.278240       1.052191
H         -4.201611      -1.765146       1.520539
C         -5.124945      -1.282477      -1.208183
H         -5.650735      -0.184332       0.597142
H         -5.707106       0.752914      -0.895519
H         -4.120269       2.331188       1.053251
H         -4.681729       2.426906      -0.628407
H         -4.585676      -3.367108      -1.060504
H         -5.810209      -2.859077       0.109138
H         -4.364833      -1.168937      -1.996789
H         -6.093528      -1.349121      -1.723633
C         -0.233454      -0.059172       0.391836
S          0.406008      -0.136052       1.944154
S          0.437684      -0.512889      -1.059346
```

4.2 TS-1p – Transition State for C-S Bond Formation (DBU pathway)

```
46
Generated from Gaussian log
H          2.425816       1.463127       1.249448
O          0.974154       2.174085       0.139318
N          2.175585       1.717642       0.281790
C          2.645488       0.937764      -0.700737
H          2.470536       1.321676      -1.706883
C          3.860252       0.135305      -0.446988
C          3.953037      -0.642081       0.716865
C          4.917604       0.141031      -1.363284
C          5.107896      -1.376323       0.972685
H          3.092421      -0.688894       1.392170
C          6.070270      -0.600972      -1.106062
H          4.837905       0.735133      -2.276196
C          6.169096      -1.354234       0.063929
H          5.176149      -1.981874       1.878091
H          6.894403      -0.589940      -1.821456
H          7.071023      -1.935548       0.264080
N         -1.381486       0.814493       0.062710
C         -2.387510      -0.059009       0.055473
C         -1.554235       2.256964      -0.193031
C         -2.079922      -1.539665       0.122617
N         -3.650176       0.384787       0.062655
C         -2.984866       2.624076      -0.518536
H         -0.868720       2.517151      -1.005709
H         -1.199827       2.778497       0.705016
C         -3.199783      -2.426125       0.660707
H         -1.742446      -1.869739      -0.873004
H         -1.206143      -1.628133       0.778771
C         -4.847909      -0.337847      -0.407554
C         -3.878192       1.795704       0.379118
H         -3.229286       2.404230      -1.568857
H         -3.129248       3.699698      -0.352556
C         -4.222116      -2.801366      -0.406321
H         -2.745614      -3.333357       1.082442
H         -3.692811      -1.911916       1.500948
C         -4.605135      -1.584597      -1.241345
H         -5.487705      -0.566504       0.461265
H         -5.393347       0.390625      -1.023938
H         -3.628800       1.969790       1.437947
H         -4.942050       2.011435       0.236395
H         -3.810420      -3.581845      -1.064740
H         -5.117942      -3.227175       0.071673
H         -3.830483      -1.363504      -1.992748
H         -5.527783      -1.785507      -1.804583
C         -0.004305       0.369801       0.391491
S          0.311600       0.088929       2.050638
S          0.815003      -0.455749      -0.888535
```

4.3 2p – Thiocarbonyl Intermediate (DBU pathway)

```
46
```

```
Generated from Gaussian log
H         -2.633343       1.506898       1.199064
O         -0.829985       2.107529       0.937997
N         -1.727333       1.037899       1.258643
C         -1.686972      -0.004792       0.220860
H         -1.043291      -0.825692       0.567755
C         -3.065015      -0.545097      -0.069505
C         -4.058875       0.287524      -0.601129
C         -3.369593      -1.878836       0.213673
C         -5.336572      -0.209766      -0.843126
H         -3.819765       1.327639      -0.838340
C         -4.649560      -2.378589      -0.033044
H         -2.598262      -2.529547       0.631937
C         -5.634599      -1.545093      -0.559817
H         -6.103292       0.445695      -1.259752
H         -4.876668      -3.422327       0.190846
H         -6.636057      -1.934238      -0.751726
N          1.074776       0.771569       0.726704
C          1.903878      -0.081446       0.123640
C          1.201197       0.934566       2.180363
C          2.151992       0.050510      -1.365385
N          2.540618      -1.010039       0.849071
C          1.117661      -0.434777       2.814012
H          0.410000       1.600203       2.524386
H          2.173822       1.407424       2.393204
C          3.624514      -0.028456      -1.787351
H          1.555094      -0.705547      -1.895086
H          1.765722       1.033843      -1.649215
C          3.654254      -1.862961       0.380413
C          2.271504      -1.251656       2.279766
H          0.147040      -0.885408       2.559145
H          1.186784      -0.374733       3.907832
C          4.067226      -1.455920      -2.090306
H          3.753474       0.604019      -2.676683
H          4.259731       0.419909      -1.007832
C          3.547067      -2.423820      -1.031286
H          4.600313      -1.311299       0.509749
H          3.680039      -2.698733       1.088618
H          3.196311      -1.016083       2.829675
H          2.084788      -2.328635       2.394604
H          3.696320      -1.761738      -3.080772
H          5.166558      -1.501549      -2.133746
H          2.498911      -2.699560      -1.223585
H          4.121129      -3.360934      -1.061002
C          0.144498       1.751304       0.003813
S          0.975839       3.171468      -0.621480
S         -0.879435       0.767156      -1.227115
```

4.4 TS-2p – Transition State for COS Elimination (DBU pathway)

```
46
Generated from Gaussian log
H         -2.474295       1.464144       1.073111
```

O	-0.720803	2.120687	0.905526
N	-1.570725	0.991377	1.018852
C	-1.580167	0.110453	-0.206498
H	-0.964181	-0.764776	0.054583
C	-3.011874	-0.383449	-0.318426
C	-4.011569	0.431499	-0.867559
C	-3.363186	-1.643182	0.173879
C	-5.331361	-0.007276	-0.922016
H	-3.723256	1.403194	-1.275272
C	-4.685705	-2.090724	0.110907
H	-2.589829	-2.283477	0.606471
C	-5.672880	-1.272718	-0.434300
H	-6.099861	0.635843	-1.355491
H	-4.943149	-3.081154	0.491254
H	-6.706873	-1.618936	-0.484674
N	1.057057	0.645116	0.930512
C	1.844430	-0.152030	0.186555
C	0.909013	0.384500	2.378426
C	2.271089	0.279795	-1.193602
N	2.247655	-1.315177	0.687749
C	0.630550	-1.083917	2.571154
H	0.093828	1.004552	2.756454
H	1.847712	0.689117	2.868897
C	3.786248	0.200852	-1.425401
H	1.718253	-0.341212	-1.911333
H	1.905668	1.291008	-1.354152
C	3.321273	-2.173047	0.126881
C	1.786376	-1.856718	1.981890
H	-0.318802	-1.333147	2.075970
H	0.535640	-1.326964	3.636998
C	4.191966	-1.139271	-2.034090
H	4.078133	1.026533	-2.088583
H	4.321206	0.372858	-0.478111
C	3.431012	-2.293778	-1.386408
H	4.277401	-1.834670	0.559756
H	3.120240	-3.167206	0.540735
H	2.650097	-1.850638	2.665073
H	1.511129	-2.904851	1.804814
H	3.988653	-1.135420	-3.115818
H	5.275973	-1.289236	-1.913645
H	2.422241	-2.399822	-1.811507
H	3.947369	-3.242606	-1.590850
C	0.529452	1.926921	0.530549
S	1.505098	3.226121	0.230201
S	-0.912147	0.901673	-1.673346

4.5 3p – Thioamide Precursor (DBU pathway)

46

Generated from Gaussian log

H	-1.446171	2.251977	-0.635681
O	0.432682	2.091914	-0.842103
N	-0.778502	1.585997	-0.237338

```
C         -1.157024      0.240546     -0.756581
H         -0.539581     -0.475127     -0.193512
C         -2.575670      0.046822     -0.248555
C         -3.637813      0.774807     -0.803030
C         -2.847015     -0.857353      0.782945
C         -4.935119      0.612186     -0.324825
H         -3.433417      1.445124     -1.641790
C         -4.150761     -1.035752      1.253209
H         -2.026938     -1.438637      1.214636
C         -5.196976     -0.296138      0.705597
H         -5.752011      1.187176     -0.765422
H         -4.346917     -1.754607      2.051211
H         -6.215980     -0.430317      1.073126
N          1.547707      0.962409      0.840049
C          1.746422     -0.296263      0.399404
C          1.211646      1.321580      2.228433
C          2.180660     -0.422521     -1.036816
N          1.568292     -1.322377      1.215852
C          0.474510      0.163208      2.855545
H          0.581137      2.217765      2.194147
H          2.141312      1.562073      2.765929
C          3.011150     -1.648772     -1.413859
H          1.273269     -0.335221     -1.684191
H          2.779382      0.473884     -1.240585
C          1.609874     -2.745353      0.801474
C          1.273417     -1.105310      2.644047
H         -0.518788      0.068740      2.391442
H          0.340030      0.339758      3.930001
C          2.152426     -2.878034     -1.681698
H          3.589553     -1.389865     -2.311244
H          3.750992     -1.854364     -0.624318
C          1.086186     -3.033812     -0.601800
H          2.631954     -3.133564      0.935795
H          0.975381     -3.260860      1.530653
H          2.225845     -1.068588      3.195872
H          0.713590     -1.976806      2.998644
H          1.659120     -2.784447     -2.660838
H          2.791897     -3.773446     -1.722807
H          0.232609     -2.371221     -0.824346
H          0.688203     -4.058344     -0.603876
C          1.509032      2.048524     -0.089859
S          2.701354      3.149277     -0.196574
S         -0.979358     -0.029826     -2.531411
```

4.6 TS-3p – Transition State for Final H-Migration (DBU pathway)

```
46
Generated from Gaussian log
H         -2.317395     -2.122014     -0.563615
O         -1.036241     -1.295337      0.895997
N         -1.580739     -1.495449     -0.918902
C         -2.174625     -0.278041     -1.265595
H         -2.358617     -0.988195     -2.195047
```

C	-3.495260	0.036376	-0.605881
C	-4.710466	-0.268690	-1.224514
C	-3.494630	0.638860	0.659850
C	-5.918916	0.029915	-0.590435
H	-4.712946	-0.732881	-2.214234
C	-4.701331	0.924779	1.292646
H	-2.541488	0.873657	1.140312
C	-5.916480	0.625483	0.669178
H	-6.863239	-0.203191	-1.086024
H	-4.695033	1.388138	2.281156
H	-6.859397	0.859100	1.166983
N	1.155911	-0.991434	0.342036
C	2.167884	-0.132903	0.076515
C	1.228200	-2.446516	0.069429
C	1.865499	1.325028	-0.134625
N	3.395719	-0.605494	-0.035925
C	2.584608	-2.837742	-0.471295
H	0.420368	-2.684964	-0.630442
H	1.030799	-2.965645	1.018182
C	2.843453	2.268440	0.567877
H	1.871698	1.476467	-1.226343
H	0.831496	1.504992	0.157683
C	4.628703	0.133569	-0.392938
C	3.623332	-2.024101	0.272646
H	2.666235	-2.628134	-1.547820
H	2.743516	-3.912921	-0.319357
C	4.033092	2.627806	-0.323055
H	2.305065	3.177338	0.869671
H	3.187711	1.797294	1.501645
C	4.498300	1.438971	-1.159959
H	5.192696	0.285299	0.542599
H	5.207217	-0.568913	-1.005875
H	3.529124	-2.170587	1.360581
H	4.645178	-2.276335	-0.025606
H	3.757843	3.448791	-1.002710
H	4.863958	2.996404	0.297960
H	3.841847	1.279619	-2.027837
H	5.495897	1.646071	-1.573981
C	-0.004113	-0.623575	1.125788
S	0.172241	0.429252	2.426634
S	-1.178012	1.064748	-1.751017

4.7 3ap – Primary Thioamide Product (DBU pathway)

46
Generated from Gaussian log

H	-0.020348	1.036349	-1.685521
O	-1.020030	2.294206	-0.810985
N	0.336163	0.178340	-2.128389
C	1.477019	-0.405980	-1.776076
H	-0.279281	-0.322619	-2.762239
C	2.364183	0.402105	-0.881826
C	2.248065	1.800240	-0.829785

```
C          3.314070     -0.231833     -0.068751
C          3.053033      2.542039      0.033003
H          1.536190      2.323881     -1.469159
C          4.111805      0.511214      0.797202
H          3.411719     -1.316800     -0.126277
C          3.980265      1.900175      0.853598
H          2.955013      3.628379      0.059941
H          4.840190      0.003432      1.431715
H          4.606162      2.482894      1.531772
N         -2.133267      0.370399     -0.221775
C         -1.410846     -0.327729      0.643282
C         -3.037548     -0.204100     -1.221768
C         -0.497865      0.472528      1.548230
N         -1.537565     -1.653893      0.718962
C         -2.776455     -1.685599     -1.384638
H         -2.875718      0.340217     -2.163942
H         -4.070120     -0.007350     -0.897669
C         -0.155186     -0.154795      2.896162
H          0.411818      0.732411      0.981087
H         -1.026306      1.418277      1.727072
C         -0.651603     -2.577872      1.454569
C         -2.644301     -2.294929     -0.003379
H         -1.846141     -1.864896     -1.945432
H         -3.604096     -2.151821     -1.933918
C          0.982609     -1.163530      2.806793
H          0.113163      0.655677      3.587634
H         -1.057582     -0.627539      3.314755
C          0.785321     -2.110240      1.627773
H         -1.110890     -2.830500      2.424962
H         -0.646660     -3.494942      0.852906
H         -3.574977     -2.160327      0.571147
H         -2.432097     -3.367258     -0.059331
H          1.942974     -0.635739      2.692664
H          1.041196     -1.735255      3.745922
H          1.106810     -1.642693      0.683673
H          1.414883     -3.003690      1.749137
C         -2.042632      1.874533     -0.288772
S         -3.383595      2.680094      0.325655
S          1.887808     -1.939783     -2.303050
```

4.8 V – DBU-Mediated H-Migration Reactant Complex

```
64
Generated from Gaussian log
N          1.343082     -0.770922     -1.179347
C          0.002117     -0.611777     -0.575470
O          2.221285     -1.270760     -0.159897
S          4.571058     -1.857279      0.726807
C          3.488359     -1.418967     -0.407382
S         -0.074446      0.723843      0.642445
Cl         3.954308     -1.152633     -2.079288
N          3.433459      2.559661      0.950733
C          3.479116      1.865874     -0.367917
```

H	4.337996	1.184287	-0.358964
H	3.606853	2.619114	-1.154569
H	2.530715	1.324411	-0.502444
C	2.305723	3.534755	0.948949
H	2.250368	4.002181	1.938884
H	1.385826	2.978642	0.721922
H	2.503222	4.290585	0.179959
C	3.209467	1.545244	2.022760
H	2.255548	1.037512	1.811514
H	3.175779	2.065933	2.986989
H	4.042686	0.832687	2.001157
C	4.716813	3.270668	1.188359
H	4.873425	3.991901	0.377819
H	5.527592	2.532995	1.202426
H	4.656678	3.788256	2.152485
H	1.293165	-1.546054	-1.852888
C	-0.552490	-1.948216	-0.101335
C	-0.276528	-2.455997	1.174255
C	-1.331371	-2.713543	-0.976268
C	-0.770000	-3.699143	1.563727
H	0.308448	-1.841516	1.860780
C	-1.836693	-3.956375	-0.585456
H	-1.561840	-2.316599	-1.969174
C	-1.555405	-4.453320	0.686318
H	-0.547824	-4.082950	2.561719
H	-2.455518	-4.533490	-1.275768
H	-1.949057	-5.423015	0.997312
H	-0.587131	-0.328074	-1.462660
N	-2.736350	0.538895	-2.075857
C	-3.366828	0.660591	-0.958296
C	-2.003832	1.674983	-2.599886
C	-4.067402	-0.586441	-0.448390
N	-3.501102	1.831521	-0.235101
C	-2.627132	3.003243	-2.202291
H	-1.942100	1.583065	-3.695968
H	-0.966168	1.637700	-2.214752
C	-3.680164	-0.991420	0.979666
H	-5.160091	-0.452882	-0.521481
H	-3.794860	-1.371427	-1.161998
C	-3.761773	1.884571	1.204488
C	-2.782041	3.013880	-0.694789
H	-3.614518	3.109355	-2.680571
H	-2.006474	3.852474	-2.522982
C	-4.614379	-0.387534	2.026487
H	-3.674724	-2.089665	1.064289
H	-2.644638	-0.662738	1.165750
C	-4.949911	1.072363	1.715082
H	-2.840870	1.613416	1.756685
H	-3.958243	2.944451	1.419883
H	-1.785528	3.042481	-0.215675
H	-3.344773	3.901593	-0.365849
H	-5.549488	-0.968661	2.083020
H	-4.142532	-0.456417	3.019810

H	-5.757471	1.140333	0.969558
H	-5.331261	1.561191	2.624174

4.9 TS-V – Transition State for DBU-Mediated Proton Abstraction

64

Generated from Gaussian log

N	0.998941	-0.286431	-0.758366
C	0.294103	0.494642	0.156982
O	2.330937	-1.049912	0.070987
S	4.458797	-2.525742	-0.275252
C	2.887799	-2.054075	-0.429094
S	1.025924	2.038049	0.716149
Cl	1.778353	-3.125684	-1.389335
N	4.889913	1.704542	-0.015079
C	4.136124	1.165238	-1.184833
H	4.393629	0.105209	-1.295377
H	4.439220	1.728811	-2.075436
H	3.062951	1.288060	-0.983401
C	4.576435	3.152334	0.133856
H	5.099713	3.534652	1.018055
H	3.489352	3.245771	0.250591
H	4.914060	3.677610	-0.767230
C	4.471500	0.979648	1.222546
H	3.391128	1.138935	1.356192
H	5.044368	1.387982	2.063824
H	4.685473	-0.086678	1.081848
C	6.347996	1.518146	-0.230289
H	6.639930	2.043351	-1.146990
H	6.548615	0.444479	-0.326079
H	6.886154	1.929583	0.631357
H	0.477327	-1.160123	-0.919504
C	-0.544868	-0.235633	1.189818
C	-1.539717	0.475198	1.879463
C	-0.390962	-1.598920	1.464565
C	-2.355536	-0.157117	2.812761
H	-1.643125	1.543856	1.680124
C	-1.194868	-2.231027	2.418771
H	0.365698	-2.188030	0.943931
C	-2.181535	-1.516926	3.092919
H	-3.126084	0.414353	3.335134
H	-1.044699	-3.291784	2.628909
H	-2.810660	-2.011736	3.835083
H	-0.457874	0.816652	-0.682504
N	-2.047306	1.489969	-1.345194
C	-3.015326	0.672225	-1.087381
C	-2.152507	2.904026	-1.035028
C	-2.778381	-0.763873	-1.527867
N	-4.195092	1.027748	-0.480902
C	-3.587632	3.366617	-0.848559
H	-1.665318	3.469989	-1.845394
H	-1.550303	3.108396	-0.131741
C	-3.237446	-1.863828	-0.570354

H	-3.242014	-0.906546	-2.519113
H	-1.695609	-0.825063	-1.697349
C	-5.249496	0.117516	-0.031431
C	-4.297320	2.379701	0.059961
H	-4.108130	3.395366	-1.819164
H	-3.625319	4.376889	-0.417358
C	-4.696025	-2.258224	-0.785155
H	-2.589839	-2.744650	-0.695700
H	-3.084682	-1.521605	0.464971
C	-5.587223	-1.031882	-0.976373
H	-5.017800	-0.277906	0.976869
H	-6.143907	0.744674	0.082290
H	-3.865417	2.419798	1.078017
H	-5.363483	2.629647	0.148405
H	-4.784476	-2.913260	-1.666704
H	-5.047265	-2.844077	0.079153
H	-5.523706	-0.656482	-2.009660
H	-6.638806	-1.310628	-0.814485

4.10 VI – Stabilized Intermediate after Proton Abstraction

64

Generated from Gaussian log

N	0.479692	-0.699060	-0.686932
C	1.078089	-1.687310	-0.100393
O	2.803872	2.009959	-0.587676
S	1.110056	4.055356	-0.532057
C	1.919405	2.682775	-1.023438
S	0.421193	-2.483741	1.291010
Cl	1.275878	1.961424	-2.749009
N	1.840390	1.133857	2.769215
C	0.559301	1.126141	2.002053
H	0.760182	1.513834	0.997274
H	-0.154565	1.772781	2.526590
H	0.197659	0.089302	1.942201
C	1.587861	0.654514	4.153929
H	2.536886	0.650008	4.702271
H	1.175834	-0.359985	4.096477
H	0.872535	1.331137	4.635365
C	2.821717	0.223707	2.106384
H	2.402599	-0.790747	2.110753
H	3.760415	0.263655	2.671525
H	2.973015	0.577153	1.080301
C	2.389010	2.517350	2.797819
H	1.659276	3.172172	3.288081
H	2.552058	2.845551	1.763473
H	3.331144	2.508127	3.358238
H	0.988637	-0.345503	-1.501616
C	2.416506	-2.156302	-0.620477
C	2.817326	-3.491804	-0.482596
C	3.283424	-1.254327	-1.256628
C	4.043816	-3.922158	-0.984733
H	2.147520	-4.183395	0.029646

C	4.519409	-1.682619	-1.741983
H	3.005483	-0.202058	-1.360393
C	4.901761	-3.017809	-1.613417
H	4.333857	-4.969576	-0.881081
H	5.187032	-0.964651	-2.222200
H	5.866941	-3.352582	-1.998295
H	-0.992505	-0.074041	-0.423599
N	-1.934052	0.460517	-0.356875
C	-3.049759	-0.225425	-0.255975
C	-1.857104	1.900485	-0.548398
C	-2.874416	-1.723128	-0.178825
N	-4.245352	0.376144	-0.240008
C	-3.167292	2.411008	-1.110589
H	-1.011423	2.105811	-1.219873
H	-1.630484	2.391420	0.411847
C	-3.784619	-2.445337	0.814206
H	-3.002780	-2.134117	-1.193669
H	-1.824930	-1.889802	0.104098
C	-5.538355	-0.288278	-0.003188
C	-4.310050	1.843772	-0.288652
H	-3.277107	2.092803	-2.158369
H	-3.195528	3.507743	-1.084953
C	-5.142775	-2.804366	0.216864
H	-3.267724	-3.353453	1.155929
H	-3.913561	-1.816706	1.709824
C	-5.708752	-1.666670	-0.631148
H	-5.734849	-0.329244	1.082275
H	-6.286967	0.387949	-0.432627
H	-4.289162	2.244847	0.738779
H	-5.275681	2.114716	-0.732498
H	-5.048906	-3.705617	-0.408760
H	-5.846519	-3.053937	1.026217
H	-5.246742	-1.652295	-1.630268
H	-6.784285	-1.826546	-0.794338

4.11 TS-VI – Transition State for Proton Transfer to Nitrogen

64

Generated from Gaussian log

N	0.364515	-0.664897	-0.358888
C	0.990662	-1.638966	0.236429
O	2.340501	1.675899	-1.397196
S	1.136069	4.025510	-1.107342
C	1.455895	2.458580	-1.583319
S	0.585435	-2.144207	1.825921
Cl	0.039541	1.676320	-2.702431
N	2.730244	1.475896	2.221076
C	1.264824	1.504914	1.937386
H	1.136268	1.690488	0.866688
H	0.817404	2.314345	2.526396
H	0.835976	0.527999	2.203523
C	2.950184	1.270328	3.677031
H	4.029039	1.222575	3.865611

H	2.468888	0.328941	3.967374
H	2.504286	2.110064	4.221542
C	3.355533	0.354236	1.460489
H	2.923508	-0.587598	1.821256
H	4.437966	0.380638	1.631712
H	3.125691	0.500653	0.398753
C	3.338597	2.764952	1.789085
H	2.858293	3.579148	2.344237
H	3.158644	2.889671	0.713686
H	4.412482	2.734229	2.007045
H	0.660274	-0.453359	-1.313066
C	2.117391	-2.324072	-0.493498
C	2.467015	-3.646380	-0.189879
C	2.834103	-1.647505	-1.492846
C	3.494698	-4.285353	-0.880013
H	1.915762	-4.161514	0.597471
C	3.873764	-2.283404	-2.171117
H	2.600317	-0.606259	-1.729354
C	4.203611	-3.605210	-1.871744
H	3.745371	-5.320452	-0.640574
H	4.429398	-1.739408	-2.937150
H	5.014127	-4.103397	-2.406916
H	-0.738946	-0.071666	0.065034
N	-1.816668	0.513949	0.295736
C	-2.931723	-0.158816	0.200092
C	-1.792396	1.964609	0.325760
C	-2.769504	-1.665810	0.175223
N	-4.141508	0.433274	0.104418
C	-2.963390	2.525868	-0.454370
H	-0.842217	2.305478	-0.105557
H	-1.826842	2.313296	1.373180
C	-3.778906	-2.467935	0.999102
H	-2.775502	-2.002104	-0.874744
H	-1.762855	-1.853034	0.569832
C	-5.438331	-0.252180	0.193356
C	-4.239731	1.897793	0.069717
H	-2.829199	2.285043	-1.520261
H	-3.017790	3.618385	-0.358605
C	-5.064946	-2.775890	0.236612
H	-3.296761	-3.404236	1.314956
H	-4.006537	-1.919228	1.927040
C	-5.549328	-1.570441	-0.566670
H	-5.716934	-0.394200	1.252645
H	-6.163558	0.455859	-0.224993
H	-4.469828	2.275422	1.081081
H	-5.088957	2.154858	-0.578168
H	-4.902823	-3.625160	-0.445515
H	-5.846339	-3.087765	0.947479
H	-4.990035	-1.474027	-1.510039
H	-6.603067	-1.712641	-0.847317

4.12 VII – Final Thioamide Product (DBU-mediated route)

```
64
Generated from Gaussian log
N         -0.443484      0.776894     -0.331569
C         -1.132508      1.732167      0.257371
O         -2.350177     -1.751428     -1.245894
S         -0.996507     -4.039666     -1.218381
C         -1.442171     -2.471997     -1.533961
S         -0.773425      2.278537      1.815747
Cl        -0.129117     -1.492643     -2.677769
N         -2.506406     -1.602519      2.332382
C         -1.069432     -1.486537      1.946537
H         -1.003556     -1.602343      0.860067
H         -0.508699     -2.280929      2.452733
H         -0.702435     -0.494166      2.241765
C         -2.639988     -1.434007      3.804090
H         -3.699746     -1.517540      4.071066
H         -2.253907     -0.444404      4.074995
H         -2.058282     -2.219333      4.299998
C         -3.291605     -0.540853      1.637083
H         -2.909866      0.436187      1.957871
H         -4.345950     -0.654670      1.914402
H         -3.159949     -0.673688      0.556955
C         -3.016866     -2.941246      1.923949
H         -2.439861     -3.708985      2.452221
H         -2.882154     -3.049205      0.840198
H         -4.077559     -3.006214      2.192621
H         -0.650937      0.483720     -1.285740
C         -2.286000      2.298026     -0.512531
C         -2.724433      3.606527     -0.269231
C         -2.946708      1.523813     -1.478803
C         -3.791793      4.137425     -0.988153
H         -2.212808      4.198400      0.490400
C         -4.024920      2.054626     -2.186050
H         -2.641808      0.491566     -1.668113
C         -4.446837      3.362123     -1.946923
H         -4.115564      5.162140     -0.798121
H         -4.537867      1.438750     -2.926610
H         -5.287913      3.776987     -2.505376
N          1.896215     -0.527553      0.482447
C          2.964023      0.174698      0.275176
C          1.926928     -1.969457      0.317923
C          2.794870      1.675088      0.445179
N          4.170432     -0.341533     -0.120522
C          2.973433     -2.407672     -0.688852
H          0.932679     -2.309641     -0.005359
H          2.122796     -2.446433      1.296657
C          3.890755      2.396971      1.233806
H          2.683280      2.135872     -0.551184
H          1.832095      1.794914      0.957702
C          5.452099      0.367885     -0.104670
C          4.301661     -1.786437     -0.305613
H          2.668985     -2.063675     -1.689614
```

H	3.062113	-3.502509	-0.722469
C	5.052960	2.851263	0.353473
H	3.447791	3.266281	1.741394
H	4.257063	1.734529	2.034697
C	5.443945	1.785649	-0.669853
H	5.882543	0.368997	0.914729
H	6.125323	-0.243218	-0.719851
H	4.691994	-2.253944	0.617501
H	5.049171	-1.955419	-1.094160
H	4.779305	3.777309	-0.176712
H	5.920226	3.097223	0.986750
H	4.765444	1.804977	-1.536618
H	6.448817	2.003183	-1.060747
H	0.392774	0.314319	0.118478

5 Secondary Substrate (2a) Pathway

5.1 2a – Secondary Aldoxime Substrate

```
19
Generated from Gaussian log
N          0.577143       1.447649       0.050428
C          0.012447       0.256973       0.635154
O          1.979694       1.325374       0.119820
H          0.151722       0.293025       1.726437
S          4.010146      -0.254082      -0.264240
C          2.432059       0.065462      -0.049030
S          1.128199      -1.102243       0.021148
C         -1.439298       0.082324       0.272175
C         -2.232692       1.212267       0.039997
C         -2.018329      -1.189453       0.206616
C         -3.585560       1.066023      -0.265647
H         -1.794057       2.209422       0.104213
C         -3.371668      -1.330460      -0.093619
H         -1.416111      -2.082411       0.391812
C         -4.157946      -0.203297      -0.333302
H         -4.194160       1.952868      -0.449724
H         -3.811070      -2.327875      -0.143782
H         -5.216677      -0.315136      -0.572691
H          0.355213       1.475721      -0.952411
```

5.2 III – Reaction Intermediate from 2a

```
46
Generated from Gaussian log
N         -1.968816       1.787653      -0.152843
C         -1.979290       0.367938       0.120969
O         -0.741461       2.102114      -0.776713
H         -1.305712       0.161668       0.963037
S          1.112160       1.359740      -2.432394
C         -0.213693       1.111141      -1.513011
S         -1.125929      -0.369258      -1.361345
C         -3.372992      -0.146549       0.344389
C         -4.335310      -0.085301      -0.671089
C         -3.718827      -0.667493       1.593954
C         -5.632257      -0.533067      -0.432946
H         -4.065568       0.302355      -1.657298
C         -5.017267      -1.120292       1.829106
H         -2.965983      -0.714607       2.383475
C         -5.974617      -1.051307       0.817878
H         -6.377575      -0.483447      -1.228388
H         -5.280645      -1.527033       2.806764
H         -6.990355      -1.405660       1.001670
H         -2.686444       2.015319      -0.850909
N          1.042532       0.516779       1.185881
C          1.981316      -0.349416       1.014646
C          1.401803       1.900487       1.439383
C          1.573325      -1.769612       0.686095
N          3.327431      -0.085058       1.062703
```

```
C          2.766361       2.051375       2.095140
H          0.615966       2.359291       2.060699
H          1.392258       2.453463       0.482490
C          1.881894      -2.180083      -0.762651
H          2.046971      -2.479812       1.384250
H          0.490563      -1.811920       0.855942
C          4.338668      -1.090110       0.762925
C          3.785066       1.283486       1.271402
H          2.739044       1.638126       3.116318
H          3.057817       3.108679       2.167456
C          3.320935      -2.624779      -1.006905
H          1.206909      -3.006088      -1.035679
H          1.631046      -1.339092      -1.430751
C          4.369139      -1.562047      -0.692661
H          4.220686      -1.961924       1.428914
H          5.304991      -0.636452       1.018175
H          4.754280       1.247397       1.789441
H          3.948569       1.790651       0.302522
H          3.529051      -3.514920      -0.386819
H          3.426602      -2.944184      -2.055521
H          5.369033      -1.973022      -0.903055
H          4.236943      -0.685261      -1.349156
```

5.3 TS-III – Transition State from 2a

```
46
Generated from Gaussian log
N         -1.996897       0.332961       1.593205
C         -1.999819      -0.083827       0.197791
O         -0.816529       1.073931       1.865919
H         -1.372919      -0.977895       0.080625
S          0.933660       2.784289       0.940635
C         -0.115782       1.482566       0.773553
S         -1.157388       1.292200      -0.683418
C         -3.402852      -0.356869      -0.276464
C         -4.340127       0.678604      -0.379717
C         -3.785980      -1.664904      -0.584286
C         -5.644698       0.404748      -0.783462
H         -4.040705       1.706182      -0.158738
C         -5.091955      -1.937512      -0.992243
H         -3.055030      -2.472171      -0.502202
C         -6.022671      -0.903992      -1.090468
H         -6.368481       1.217400      -0.864540
H         -5.382213      -2.961603      -1.232622
H         -7.044418      -1.116481      -1.409782
H         -2.747751       1.017270       1.729613
N          1.001281      -0.219278       0.655848
C          1.979270      -0.421812      -0.177664
C          0.854307      -1.059651       1.834223
C          2.193828       0.540364      -1.322476
N          2.876539      -1.437240      -0.060302
C          1.416484      -2.452046       1.607917
H         -0.207704      -1.122563       2.102504
```

H	1.360964	-0.572022	2.685291
C	3.469379	1.387244	-1.164796
H	2.224290	-0.019934	-2.270699
H	1.329579	1.203768	-1.365646
C	3.978133	-1.629981	-1.003870
C	2.834032	-2.341141	1.086104
H	0.795687	-2.985052	0.870169
H	1.399555	-3.030000	2.541761
C	4.748214	0.713061	-1.650080
H	3.320099	2.319109	-1.730339
H	3.565142	1.686571	-0.109462
C	5.086397	-0.581000	-0.918331
H	3.588882	-1.679939	-2.033291
H	4.393545	-2.620182	-0.781137
H	3.204159	-3.322383	0.760221
H	3.510386	-1.978792	1.879898
H	4.645589	0.490378	-2.726740
H	5.588405	1.418381	-1.556386
H	6.001563	-1.013091	-1.351844
H	5.299839	-0.380422	0.144886

5.4 IV – Thioamide Precursor from 2a

37
Generated from Gaussian log

N	1.393508	0.741439	-1.638623
C	1.644497	-0.484420	-0.868249
O	1.012169	1.767651	-0.724967
S	-0.460306	2.325811	1.344673
C	0.268867	1.327775	0.271106
S	0.290429	-0.422839	0.364206
Cl	-2.164264	0.812356	-2.123785
N	-4.038591	-0.945594	0.523436
C	-3.153396	-1.909742	-0.192533
H	-2.611578	-1.339390	-0.962936
H	-3.782964	-2.689899	-0.637122
H	-2.463711	-2.349908	0.537668
C	-4.783504	-1.640201	1.602479
H	-5.430947	-0.913335	2.106500
H	-4.062253	-2.061070	2.312647
H	-5.386504	-2.439327	1.155714
C	-3.193871	0.145086	1.097470
H	-2.483882	-0.301447	1.804472
H	-3.850806	0.851441	1.619715
H	-2.674031	0.630290	0.256293
C	-4.993158	-0.349320	-0.456419
H	-5.603443	-1.156967	-0.877862
H	-4.392608	0.147401	-1.233141
H	-5.627818	0.369770	0.074972
H	2.277687	1.097295	-2.012856
C	3.020773	-0.595280	-0.252851
C	3.421248	0.211409	0.821714
C	3.938129	-1.487050	-0.817125

C	4.722873	0.130154	1.310309
H	2.714508	0.897595	1.295036
C	5.239729	-1.573196	-0.321210
H	3.627638	-2.120226	-1.651735
C	5.634519	-0.762200	0.741118
H	5.025692	0.762203	2.146776
H	5.944794	-2.277205	-0.766386
H	6.651496	-0.827328	1.131714
H	1.457226	-1.327693	-1.546006

5.5 TS-IV – Transition State for H-Migration from 2a

```
37
Generated from Gaussian log
N          -1.476199       1.645045       0.628190
C          -1.315073       0.178736       0.324414
O          -0.503934       2.461246      -0.001866
S           1.919072       2.850630      -0.836468
C           0.757506       2.109298       0.063345
S          -0.192516      -0.132788      -1.054893
Cl          1.204333       1.353932       1.635284
N           3.270357      -1.802766      -0.096885
C           2.352472      -1.929017       1.071678
H           2.791820      -1.389794       1.918552
H           2.242957      -2.993539       1.310288
H           1.389216      -1.485569       0.784036
C           2.687175      -2.549215      -1.247407
H           3.336238      -2.408350      -2.119225
H           1.684113      -2.145646      -1.436351
H           2.631729      -3.610703      -0.980108
C           3.401602      -0.361143      -0.464087
H           2.401566       0.017747      -0.712492
H           4.077378      -0.284860      -1.324152
H           3.810336       0.181970       0.395545
C           4.604941      -2.360490       0.243692
H           4.484524      -3.412148       0.528584
H           5.019933      -1.784725       1.078790
H           5.254552      -2.276404      -0.635074
H          -2.312495       1.976893       0.145329
C          -2.715765      -0.369686       0.132681
C          -3.438674      -0.095471      -1.037126
C          -3.321376      -1.125708       1.139352
C          -4.738169      -0.568644      -1.193003
H          -2.952116       0.475116      -1.831999
C          -4.622491      -1.610660       0.980768
H          -2.766641      -1.337115       2.056798
C          -5.334408      -1.331150      -0.183593
H          -5.289516      -0.349673      -2.109561
H          -5.080558      -2.204966       1.773759
H          -6.351654      -1.706754      -0.308740
H          -0.904028      -0.270835       1.239460
```

5.6 IVp – Thioamide Product from 2a

```
37
Generated from Gaussian log
N         -0.881245       1.408354      -0.933124
C         -0.943356      -0.038043      -0.584287
O          0.477377       1.760406      -1.205715
S          2.733283       2.785079      -0.514454
C          1.201194       2.300503      -0.265995
S         -0.332385      -1.146099      -1.874924
Cl         0.429913       2.460788       1.301326
N          2.591098      -2.095704       0.574968
C          1.253430      -2.470258       1.119816
H          1.054725      -1.853511       2.004425
H          1.278813      -3.531320       1.395107
H          0.509582      -2.290823       0.328347
C          2.845468      -2.883464      -0.664924
H          3.823713      -2.594516      -1.065773
H          2.044584      -2.642728      -1.376238
H          2.836184      -3.949243      -0.409003
C          2.597603      -0.642380       0.230483
H          1.838611      -0.483275      -0.553086
H          3.595735      -0.382064      -0.141034
H          2.372714      -0.071208       1.139942
C          3.645846      -2.377488       1.583521
H          3.628290      -3.447676       1.819790
H          3.435470      -1.786396       2.482430
H          4.617952      -2.096412       1.162466
H         -1.279098       1.487845      -1.874068
C         -2.391064      -0.242740      -0.127039
C         -3.206644      -1.245802      -0.653104
C         -2.915259       0.599726       0.866007
C         -4.517091      -1.409966      -0.196611
H         -2.784539      -1.892939      -1.423317
C         -4.220524       0.436740       1.324252
H         -2.285035       1.393413       1.273218
C         -5.028575      -0.571296       0.791825
H         -5.141547      -2.199889      -0.619233
H         -4.611358       1.099983       2.098613
H         -6.052486      -0.700012       1.148161
H         -0.319353      -0.121287       0.325910
```
